# Twenty years of participation of racialised groups in type 2 diabetes randomised clinical trials: a meta-epidemiological review

**DOI:** 10.1007/s00125-023-06052-w

**Published:** 2024-01-04

**Authors:** Rabeeyah Ahmed, Russell J. de Souza, Vincent Li, Laura Banfield, Sonia S. Anand

**Affiliations:** 1 Department of Medicine, Faculty of Health Sciences, McMaster University, Hamilton, ON Canada; 2 Chanchlani Research Centre, McMaster University, Hamilton, ON Canada; 3 Department of Health Research Methods, Evidence and Impact, McMaster University, Hamilton, ON Canada; 4 Health Sciences Library, McMaster University, Hamilton, ON Canada

**Keywords:** Ethnicity, Meta-analysis, Race, RCT, Type 2 diabetes

## Abstract

**Aims/hypothesis:**

Type 2 diabetes mellitus prevalence is increasing globally and the greatest burden is borne by racialised people. However, there are concerns that the enrolment of racialised people into RCTs is limited, resulting in a lack of ethnic and racial diversity. This may differ depending whether an RCT is government funded or industry funded. The aim of this study was to review the proportions of racialised and white participants included in large RCTs of type 2 diabetes pharmacotherapies relative to the disease burden of type 2 diabetes in these groups.

**Methods:**

The Ovid MEDLINE database was searched from 1 January 2000 to 31 December 2020. English language reports of RCTs of type 2 diabetes pharmacotherapies published in select medical journals were included. Studies were included in this review if they had a sample size of at least 100 participants and all participants were adults with type 2 diabetes. Industry-funded trials must have recruited participants from at least two countries. Government-funded trials were not held to the same standard because they are typically conducted in a single country. Data including the numbers and proportions of participants by ethnicity and race were extracted from trial reports. The participation-to-prevalence ratio (PPR) was calculated for each trial by dividing the percentage of white and racialised participants in each trial by the percentage of white and racialised participants with type 2 diabetes, respectively, for the regions of recruitment. A random-effects meta-analysis was used to generate the pooled PPRs and 95% CIs across study types. A PPR <0.80 indicates under-representation and a PPR >1.20 indicates over-representation. Risk of bias assessments were not conducted for this study as the objective was to examine recruitment of racialised and white participants rather than evaluate the trustworthiness of clinical trial outcomes.

**Results:**

A total of 83 trials were included, involving 283,122 participants, of which 15 were government-funded and 68 were industry-funded trials. In government-funded trials, the PPR for white participants was 1.11 (95% CI 0.99, 1.24) and the PPR for racialised participants was 0.72 (95% CI 0.60, 0.86). In industry-funded trials, the PPR for white participants was 1.95 (95% CI 1.74, 2.18) and the PPR for racialised participants was 0.36 (95% CI 0.32, 0.42). The limitations of this study include the reliance on investigator-reported ethnicity and race to classify participants as ‘white’ or ‘racialised’, the use of estimates for type 2 diabetes prevalence and demographic data, and the high levels of heterogeneity of pooled estimates. However, despite these limitations, the results were consistent with respect to direction.

**Conclusions/interpretation:**

Racialised participants are under-represented in government- and industry-funded type 2 diabetes trials. Strategies to improve recruitment and enrolment of racialised participants into RCTs should be developed.

**Registration:**

Open Science Framework registration no. f59mk (https://osf.io/f59mk)

**Funding:**

The authors received no financial support for this research or authorship of the article.

**Graphical Abstract:**

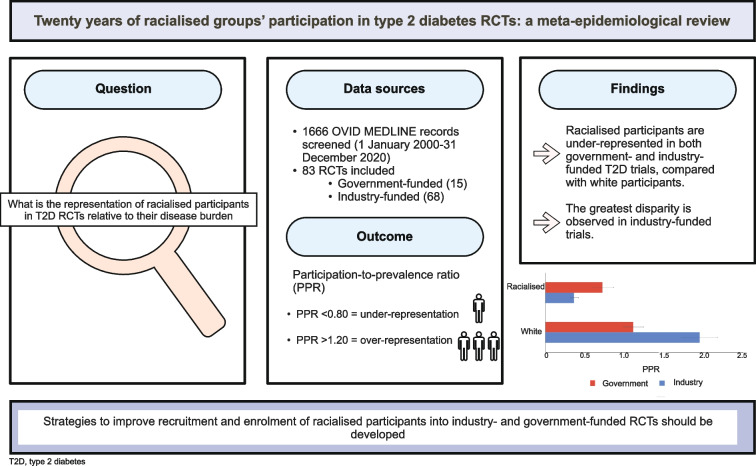

**Supplementary Information:**

The online version contains peer-reviewed but unedited supplementary material available at 10.1007/s00125-023-06052-w.


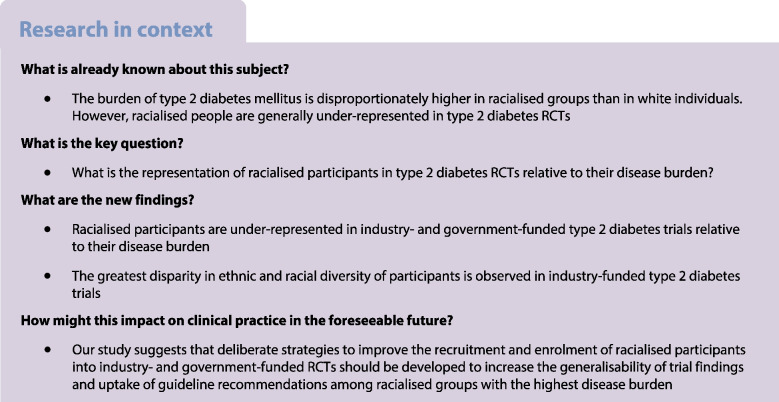


## Introduction

The burden of type 2 diabetes is disproportionately higher in non-white ethnic and racial groups than in white individuals [1]. However, individuals from non-white ethnic or racial groups, herein referred to as racialised people, are generally under-represented in RCTs, which by design produce the most reliable evidence regarding the efficacy and safety of medical therapies [1, 2]. As such, RCTs inform treatment recommendations in guidelines. Under-representation of racialised people in RCTs can limit the generalisability of the trial findings and uptake of guideline recommendations among racialised groups with the highest disease burden [3].

In the USA, the National Institutes of Health (NIH) provides guidelines for the inclusion of racialised groups in the clinical research they fund, a measure taken to improve the generalisability of research findings [4]. Industry-funded trials do not have the same requirements as the NIH, nor do many other government-established funding agencies [5–8]. For example, Canada and Australia do not have guidelines for the recruitment of racialised populations into clinical trials. The ‘Guidance for industry: standards for clinical trials in type 2 diabetes in Canada’ (2007) document does not mention the terms ‘race’ or ‘ethnicity,’ nor does it provide guidelines on participant recruitment [7]. Similarly, the Government of Australia’s guidelines for clinical trials do not include any regulations pertaining to recruitment of racialised groups [8]. In the UK, there is also no requirement to record and report ethnicity or race in research studies [5].

The NIH requirements are guided by the Public Health Service Act sec. 492B, 42 U.S.C. sec. 289a-2 and are designed to enhance the inclusion of minority groups in NIH-funded research [4]. Efforts to increase representation of racialised groups in RCTs have been made internationally as well. For example, some research institutions in the UK are taking measures to address the lack of ethnic participant recruitment guidelines for clinical trials. In 2018, the UK’s National Institute for Health and Care Research (NIHR) launched the INCLUDE project, designed to enhance the diversity of research participants in clinical studies [5].

In this study we conducted a meta-epidemiological review of Phase II–IV RCTs published between 1 January 2000 and 31 December 2020 that tested at least one type 2 diabetes pharmacotherapy and investigated (1) the participation of racialised individuals relative to the disease burden of type 2 diabetes in large RCTs in which type 2 diabetes therapies were evaluated and (2) the differences in participation of racialised and white individuals between industry- and government-funded trials.

## Methods

This meta-epidemiological review was developed in accordance with the Preferred Reporting Items for Systematic Reviews and Meta-analyses (PRISMA) reporting guidelines. This study was registered on Open Science Framework (registration no. f59mk; https://osf.io/f59mk).

### Data sources and searches

Guided by a clinical expert, a broad search strategy with keywords related to type 2 diabetes RCTs was developed (electronic supplementary material [ESM] Table 1). Initially, the search was limited to trials published in the *New England Journal of Medicine*, *The Lancet*, *JAMA*, *The BMJ* and *Annals of Internal Medicine*. These journals were selected because they have a history of being targeted for publication of large, high-impact, multicountry trials of type 2 diabetes drugs. This criterion was kept for industry-funded trials; however, it was expanded for government-funded trials to include publications from five specialty journals (*Diabetes Care*, *Circulation*, *Lancet Diabetes and Endocrinology*, *JAMA Internal Medicine*, *JAMA Ophthalmology*), as we recognise that discipline-specific journals are more likely to publish smaller-scale government-funded trials that recruit fewer participants and/or are conducted in a single country. A complete list of study selection criteria can be found in ESM Table 2. The Ovid MEDLINE database was searched from 1 January 2000 to 31 December 2020 to identify relevant studies. The year 2000 was selected as a starting point because most of the oral hypoglycaemic agents for diabetes (metformin, glimepiride, rosiglitazone) were approved in the mid to late 1990s. As such, their use in clinical trials became more widespread in 2000 and beyond. The endpoint of our time frame was selected to capture trends before the COVID-19 pandemic. The decade-by-decade analysis presents an opportunity to revisit the data and examine trends in 10-year increments. Ovid MEDLINE was used because the identified journals are all indexed in the database, which eliminated the need for multiple database searching. Full-text screening was completed by two reviewers (RA and RJdS) independently and in duplicate based on predetermined study selection criteria (ESM Fig. 1). Covidence (https://www.covidence.org/) was used for data management.

### Data extraction

Three reviewers (authors RA and VL, with JW) independently extracted the following information from published studies: title, year of publication, journal, primary funding source, pharmacotherapy intervention, total number of participants, country or region of greatest participant recruitment, and numbers of white and racialised participants. For this review, participants were categorised as ‘racialised’ if they belonged to any race or ethnicity that was not specifically described as ‘white’ or ‘Caucasian’ by the investigators. Discrepancies were cross-checked and, where necessary, were resolved by discussion with the senior author (SSA). The resource ClinicalTrials.gov and other publicly available web resources were consulted to obtain any missing information that was not in the main articles or supplementary materials.

### Outcome measures

There were two outcomes of interest for this meta-analysis: the proportion of white participants in government- and industry-funded trials relative to the type 2 diabetes disease burden in the population, and the proportion of racialised participants in government- and industry-funded trials relative to the type 2 diabetes disease burden in the population.

### Statistical analysis

The participation-to-prevalence ratio (PPR) metric was used to estimate the representation of white participants and the representation of racialised participants compared with their respective disease burden, separately in industry- and government-funded trials. The PPR for white participants and racialised participants in each trial was calculated using the respective formulas below.

$$\mathrm{PPR}=\frac{\mathrm{Percentage\;of\;white\;participants\;in\;the\;trial\;}(\mathrm{\%})}{\mathrm{Percentage\;of\;white\;people\;among\;the\;diseased\;population\;}(\mathrm{\%})}$$

$$\mathrm{PPR}=\frac{\mathrm{Percentage\;of\;racialised\;participants\;in\;the\;trial\;}(\mathrm{\%})}{\mathrm{Percentage\;of\;racialised\;people\;among\;the\;diseased\;population\;}(\mathrm{\%})}$$

A PPR of 1 suggests that racialised or white people make up the same proportion of participants in the trial as the proportion of racialised or white people, respectively, among the diseased population in the countries from which a trial recruited. For example, if 80% of participants in a trial are racialised and 80% of the cases of diabetes in the country or region occur in racialised groups, the PPR would be 1. The underlying goal for equity should be for the proportion of participants recruited by ethnicity or race to be similar to the disease burden faced by those ethnic or racial groups in that country or region. For this review, a group was considered to be under-represented when the PPR was <0.80 and over-represented when the PPR was >1.20. These decision points are consistent with a 2020 study evaluating the participation of women in cardiovascular RCTs [9].

The numerator for the PPR was known for each trial. The denominator, however, was calculated for each trial using prevalence and demographic data. A detailed explanation of PPR calculations is provided in ESM Methods. A list of estimates used for the PPR calculations is provided in ESM Table 3.

Once the PPRs for white and racialised people were calculated for each trial, a random-effects meta-analysis was used to pool the individual-study PPRs and compute the overall 95% CIs for the pooled PPRs. A random-effects model was used for this analysis because it provides appropriately wider CIs and study weights in the presence of heterogeneity, which we expected to see across varying recruitment approaches, trial conditions and countries of conduct. Under this model, it can be assumed that the true PPR may differ according to the setting, country or type of trial.

We conducted a sensitivity analysis by varying our worldwide population proportion estimates (11.7% white and 88.3% racialised). These estimates were used when a trial that recruited from three or more regions did not provide data on how many participants were recruited from each country/region. We systematically altered the proportion of racialised people to values from 80% to 90% in increments of 2.5%, and of white participants from 10% to 20% in increments of 2.5%.

All study-specific PPR estimates were calculated in Microsoft Excel and the pooled PPRs across studies and 95% CIs were calculated using Review Manager version 5.4 (RevMan; The Cochrane Collaboration, London, UK).

## Results

Of the 512 records that were assessed for eligibility, 83 RCTs with either industry funding or government funding with a total of 283,122 participants were included (ESM Fig. 1). Three other studies with dual funding were included in a sensitivity analysis. The RCTs that were excluded after full-text review are listed in ESM Table 4. Of the 83 trials included in the review, 15 (18.1%) were government-funded [10–24], 68 (81.9%) were sponsored by industry [25–92] and 49 (59.0%) were published between the years 2011 and 2020. The proportion of racialised participants in this set of trials increased significantly from 10.7% in 2000–2005 to 23.6% in 2006–2010 and remained relatively constant between 2006 and 2020. Over one-third of the studies in the review (42.2%) recruited the greatest number of participants from the USA. The most common pharmacotherapy interventions were from the glucagon-like peptide-1 (GLP-1) receptor agonist, sodium–glucose co-transporter 2 (SGLT-2) inhibitor and dipeptidyl peptidase-4 (DPP-4) inhibitor classes. The percentage of racialised participants across all trials was 23.8%. Government-funded trials had a higher overall percentage of racialised participants (26.0%) than industry-funded studies (23.4%) (Table 1). The list of included RCTs is provided in Table 2.

**Table 1:** Baseline characteristics of the 83 RCTs included in the meta-epidemiological review

Category	Number of trials, *N* (%)	White participants, *N* (%)	Racialised participants, *N* (%)
Overall	83	215,840	67,282
Sponsor			
Government	15 (18.1)	25,721 (74.0)	9059 (26.0)
Industry	68 (81.9)	190,119 (76.6)	58,223 (23.4)
Publication years			
2000–2005	11 (13.3)	21,323 (89.3)	2562 (10.7)
2006–2010	23 (27.7)	28,210 (76.4)	8738 (23.6)
2011–2015	26 (31.3)	61,157 (72.9)	22,763 (27.1)
2016–2020	23 (27.7)	105,150 (76.0)	33,219 (24.0)
Trial size			
Quartile 1 (100–500)	20 (24.1)	3987 (69.5)	1747 (30.5)
Quartile 2 (501–1500)	24 (28.9)	14,802 (81.7)	3313 (18.3)
Quartile 3 (1501–5000)	18 (21.7)	39,433 (77.8)	11,251 (22.2)
Quartile 4 (>5000)	21 (25.3)	157,618 (75.6)	50,971 (24.4)
Region of greatest recruitment			
Global	12 (14.5)	7887 (80.8)	1872 (19.2)
Europe	13 (15.7)	45,004 (80.4)	10,976 (19.6)
North America	5 (6.0)	5333 (75.4)	1743 (24.6)
North America and Europe	4 (4.8)	2057 (88.5)	266 (11.5)
North America and South America	1 (1.2)	480 (87.9)	66 (12.1)
USA	35 (42.2)	129,911 (73.5)	46,769 (26.5)
UK	2 (2.4)	3329 (90.9)	335 (9.1)
Australia	1 (1.2)	9093 (92.8)	702 (7.2)
China	1 (1.2)	0 (0)	304 (100)
Argentina	1 (1.2)	7498 (75.7)	2403 (24.3)
Qatar	1 (1.2)	16 (6.9)	215 (93.1)
Greece	1 (1.2)	100 (100)	0 (0)
Canada	1 (1.2)	152 (30.3)	350 (69.7)
Europe and USA	1 (1.2)	426 (91.8)	38 (8.2)
Bulgaria	1 (1.2)	691 (84.2)	130 (15.8)
Russian Federation	1 (1.2)	527 (94.6)	30 (5.4)
Serbia	1 (1.2)	2119 (87.6)	299 (12.4)
Slovakia	1 (1.2)	1217 (60.8)	784 (39.2)
Pharmacotherapy intervention			
GLP-1 receptor agonists	16 (19.3)	45,137 (20.9)	12,834 (19.1)
SGLT-2 inhibitors	7 (8.4)	38,089 (17.7)	10,226 (15.2)
DPP-4 inhibitors	6 (7.2)	32,371 (15.0)	11,670 (17.3)
Aliskiren	1 (1.2)	4850 (2.2)	3711 (5.5)
Bardoxolone methyl	1 (1.2)	1694 (0.8)	491 (0.7)
Ticagrelor	1 (1.2)	13,696 (6.4)	5524 (8.2)
Aleglitazar	1 (1.2)	4818 (2.2)	2408 (3.6)
Atrasentan	1 (1.2)	2110 (1.0)	1558 (2.3)
Apabetalone	1 (1.2)	2119 (1.0)	299 (0.4)
Finerenone	1 (1.2)	691 (0.3)	130 (0.2)
Darbepoetin alfa	1 (1.2)	2570 (1.2)	1468 (2.2)
Rimonabant	1 (1.2)	925 (0.4)	120 (0.2)
Angiotensin receptor blockers	4 (4.8)	7587 (3.5)	868 (1.3)
Fibrates	2 (2.4)	9493 (4.4)	720 (1.1)
Atorvastatin	1 (1.2)	2676 (1.2)	162 (0.2)
Salicylates	2 (2.4)	206 (0.1)	188 (0.3)
Metformin and glipizide	1 (1.2)	0 (0)	304 (0.5)
Insulin	7 (8.4)	8555 (4.0)	2559 (3.8)
Thiazolidinediones	2 (2.4)	5264 (2.4)	74 (0.1)
Metformin	1 (1.2)	152 (0.1)	350 (0.5)
Two or more classes of pharmacotherapies	25 (30.1)	32,837 (15.2)	11,618 (17.3)

**Table 2:** List of RCTs included in the meta-epidemiological review

Publication	Year	Journal	Pharmacotherapy intervention	Number of participants	White participants *N* (%)	Racialised participants *N* (%)	Country/region of greatest participant recruitment
ACCORD [10]	2008	*NEJM*	Intense (HbA_1c_ <6%, <42 mmol/mol) vs standard therapy	10,251	6604 (64.4)	3647 (35.6)	USA
BARI 2D [11]	2009	*NEJM*	Insulin-providing drugs vs insulin-sensitising drugs	2368	1561 (65.9)	807 (34.1)	USA
CARDS [12]	2004	*Lancet*	Atorvastatin	2838	2676 (94.3)	162 (5.7)	UK
FIELD [13]	2005	*Lancet*	Fenofibrate	9795	9093 (92.8)	702 (7.2)	Australia
GRADE [14]	2019	*Diabetes Care*	Glimepiride, sitagliptin, liraglutide, insulin glargine	5047	3314 (65.7)	1733 (34.3)	USA
Kadoglou et al [15]	2007	*Diabetes Care*	Rosiglitazone	100	100 (100)	0 (0)	Greece
Levin et al [16]	2000	*Diabetes Care*	Intensive vs standard treatment (insulin)	153	99 (64.7)	54 (35.3)	USA
Meyer et al [17]	2010	*Diabetes Care*	Gliusine vs insulin	180	136 (75.6)	44 (24.4)	USA
MiTy [18]	2020	*Lancet Diabetes and Endocrinology*	Metformin	502	152 (30.3)	350 (69.7)	Canada
SPREAD-DIMCAD [19]	2013	*Diabetes Care*	Glizipide plus metformin placebo or metformin plus glipizide placebo	304	0 (0)	304 (100)	China
The Qatar Study [20]	2017	*Diabetes Care*	Exenatide, pioglitazone, insulin therapy	231^a^	16 (6.9)^b^	215 (93.1)	Qatar
TINSAL-T2D [21]	2010	*Annals of Internal Medicine*	Salsalate	108	55 (50.9)	53 (49.1)	USA
TINSAL-T2D II [22]	2013	*Annals of Internal Medicine*	Salsalate	286	151 (52.8)	135 (47.2)	USA
UKPDS 57 [23]	2002	*Diabetes Care*	Insulin vs diet control	826	653 (79.1)	173 (20.9)	UK
VADT [24]	2009	*NEJM*	Insulin, glimepiride, rosiglitazone, metformin	1791	1111 (62)	680 (38)	USA
1860-LIRA-DPP-4 [25]	2010	*Lancet*	Liraglutide vs sitagliptin	665	576 (86.6)	89 (13.4)	North America and Europe
4-T Study Group [26]	2007	*NEJM*	Biphasic vs prandial vs basal insulin	708	653 (92.2)	55 (7.8)	Europe
ADOPT [27]	2006	*NEJM*	Rosiglitazone vs metformin vs glyburide (glibenclamide)	4351^c^	3847 (88.4)	504 (11.6)	North America
AleCardio [28]	2014	*JAMA*	Aleglitazar	7226	4818 (66.7)	2408 (33.3)	USA
ALTITUDE [29]	2012	*NEJM*	Aliskiren	8561	4850 (56.7)	3711 (43.3)	Europe
ARTS-DN [30]	2015	*JAMA*	Finerenone	821	691 (84.2)	130 (15.8)	Bulgaria
AVOID [31]	2008	*NEJM*	Aliskiren, losartan	599	520 (86.8)	79 (13.2)	North America and Europe
AWARD-4 [32]	2015	*Lancet*	Dutaglutide, glargine	884	697 (78.8)	187 (21.2)	USA
AWARD-6 [33]	2014	*Lancet*	Dulaglutide vs liraglutide	599	515 (86)	84 (14)	USA
Bailey et al [34]	2010	*Lancet*	Dapagliflozin	546	480 (87.9)	66 (12.1)	North America and South America
Barnett et al [35]	2013	*Lancet*	Linagliptin	241	233 (96.7)	8 (3.3)	Worldwide
BEACON [36]	2013	*NEJM*	Bardoxolone methyl	2185	1694 (77.5)	491 (22.5)	USA
BEGIN Basal-Bolus Type 2 [37]	2012	*Lancet*	Insulin degludec vs insulin glargine	992	822 (82.9)	170 (17.1)	Worldwide
Burant et al [38]	2012	*Lancet*	TAK-875, glimepiride, placebo	426	352 (82.6)	74 (17.4)	North America
Buse et al [39]	2011	*Annals of Internal Medicine*	Exenatide	259	201 (77.6)	58 (22.4)	North America and Europe
CANTATA-SU [40]	2013	*Lancet*	Canagliflozin, glimepiride	1450	978 (67.4)	472 (32.6)	USA
CANVAS [41]	2017	*NEJM*	Canagliflozin	10,142	7944 (78.3)	2198 (21.7)	USA
CARMELINA [42]	2019	*JAMA*	Linagliptin	6979	5596 (80.2)	1383 (19.8)	Europe
CREDENCE [43]	2019	*NEJM*	Canagliflozin	4401	2931 (66.6)	1470 (33.4)	USA
DAIS [44]	2001	*Lancet*	Fenofibrate	418	400 (95.7)	18 (4.3)	Europe
Dapagliflozin 006 [45]	2012	*Annals of Internal Medicine*	Dapagliflozin	800^d^	760 (95)	40 (5)	North America and Europe
Davies et al [46]	2017	*JAMA*	Semaglutide	630	523 (83)	107 (17)	USA
DECLARE-TIMI 58 [47]	2019	*NEJM*	Dapagliflozin, placebo	17,160	13,653 (79.6)	3507 (20.4)	USA
DETAIL [48]	2004	*NEJM*	Telmisartan vs enalapril	250	246 (98.4)	4 (1.6)	Europe
DEVOTE [49]	2017	*NEJM*	Insulin degludec, insulin glargine	7637	5775 (75.6)	1862 (24.4)	USA
DIRECT-Protect 2 [50]	2008	*Lancet*	Candesartan	1905	1830 (96.1)	75 (3.9)	Europe
DUAL V [51]	2016	*JAMA*	Insulin glargine, liraglutide	557	527 (94.6)	30 (5.4)	Russian Federation
DURATION-1 [52]	2008	*Lancet*	Exenatide	295	230 (78)	65 (22)	North America
DURATION-2 [53]	2010	*Lancet*	Exenatide, sitagliptin, pioglitazone	491^e^	168 (34.2)	323 (65.8)	North America
DURATION-3 [54]	2010	*Lancet*	Exenatide vs insulin glargine	456	379 (83.1)	77 (16.9)	Worldwide
DURATION-6 [55]	2013	*Lancet*	Exenatide vs liraglutide	911	753 (82.7)	158 (17.3)	Worldwide
ELIXA [56]	2015	*NEJM*	Lixisenatide	6068	4576 (75.4)	1492 (24.6)	USA
EMPA-REG Outcome [57]	2015	*NEJM*	Empagliflozin	7020	5081 (72.4)	1939 (27.6)	Europe
EUREXA [58]	2012	*Lancet*	Exenatide vs glimepiride	977	894 (91.5)	83 (8.5)	Europe
EXAMINE [59]	2013	*NEJM*	Alogliptin	5380	3909 (72.7)	1471 (27.3)	USA
EXSCEL [60]	2017	*NEJM*	Exenatide	14,752	11,175 (75.8)	3577 (24.2)	Europe
Frias et al [61]	2018	*Lancet*	LY3298176	316^f^	253 (80.1)	63 (19.9)	USA
Gallwitz et al [62]	2012	*Lancet*	Linagliptin, glimepiride	1551^g^	1319 (85)	232 (15)	Worldwide
Harmony Outcomes [63]	2018	*Lancet*	Albiglutide	9463	8030 (84.9)	1433 (15.1)	USA
Heine et al [64]	2005	*Annals of Internal Medicine*	Exenatide, insulin glargine	549^h^	440 (80.1)	109 (19.9)	Worldwide
INTERVAL [65]	2013	*Lancet*	Vildagliptin	278	269 (96.8)	9 (3.2)	Europe
IRMA-2 [66]	2001	*NEJM*	Irbesartan	590^i^	574 (97.3)	16 (2.7)	Worldwide
LEAD-3 Mono [67]	2009	*Lancet*	Liraglutide vs glimepiride	746	583 (78.2)	163 (21.8)	USA
LEAD-6 [68]	2009	*Lancet*	Liraglutide vs exenatide	464	426 (91.8)	38 (8.2)	Europe and USA
LEADER [69]	2016	*NEJM*	Liraglutide	9340	7238 (77.5)	2102 (22.5)	USA
Lewis et al [70]	2001	*NEJM*	Irbesartan, amlodipine	1715	1242 (72.4)	473 (27.6)	Worldwide
PERISCOPE [71]	2008	*JAMA*	Pioglitazone, glimepiride	543	445 (82)	98 (18)	USA
PIONEER 3 [72]	2019	*JAMA*	Semaglutide, sitagliptin	1864	1324 (71)	540 (29)	USA
PIONEER 4 [73]	2019	*Lancet*	Semaglutide, liraglutide	711	519 (73)	192 (27)	USA
PIONEER 6 [74]	2019	*NEJM*	Semaglutide	3183	2300 (72.3)	883 (27.7)	USA
PROactive [75]	2005	*Lancet*	Pioglitazone	5238	5164 (98.6)	74 (1.4)	Europe
Ray et al [76]	2020	*JAMA*	Apabetalone	2418^j^	2119 (87.6)	299 (12.4)	Serbia
RECORD [77]	2009	*Lancet*	Rosiglitazone vs metformin plus sulfonylurea	4447	4399 (98.9)	48 (1.1)	Europe
RENAAL [78]	2001	*NEJM*	Losartan	1513	736 (48.6)	777 (51.4)	North America
REWIND [79]	2019	*Lancet*	Dulaglutide	9901	7498 (75.7)	2403 (24.3)	Argentina
RIO-Diabetes [80]	2006	*Lancet*	Rimonabant	1045	925 (88.5)	120 (11.5)	Worldwide
ROADMAP [81]	2011	*NEJM*	Olmesartan	4447	4447 (100)	0 (0)	Europe
Rosenstock et al [82]	2010	*Lancet*	Inhaled insulin, glargine vs biaspart insulin	618^k^	417 (67.5)	201 (32.5)	Worldwide
SAVOR-TIMI 53 [83]	2013	*NEJM*	Saxagliptin	16,492	12,407 (75.2)	4085 (24.8)	USA
SCALE [84]	2015	*JAMA*	Liraglutide	846	705 (83.3)	141 (16.7)	Worldwide
SONAR [85]	2019	*Lancet*	Atrasentan	3668	2110 (57.5)	1558 (42.5)	USA
TECOS [86]	2015	*NEJM*	Sitagliptin	14,671	9957 (67.9)	4714 (32.1)	USA
THEMIS [87]	2019	*NEJM*	Ticagrelor	19,220	13,696 (71.3)	5524 (28.7)	USA
TREAT [88]	2009	*NEJM*	Darbepoetin alfa	4038	2570 (63.6)	1468 (36.4)	USA
VERIFY [89]	2019	*Lancet*	Vildagliptin, metformin	2001	1217 (60.8)	784 (39.2)	Slovakia
VERTIS CV [90]	2020	*NEJM*	Ertugliflozin	8246	7240 (87.8)	1006 (12.2)	USA
Zinman et al [91]	2007	*Annals of Internal Medicine*	Exenatide	233	195 (83.7)	38 (16.3)	USA
Zinman et al [92]	2011	*Lancet*	Insulin degludec, insulin glargine, metformin	245	78 (31.8)	167 (68.2)	Worldwide
Fayfman et al [93]^l^	2019	*Diabetes Care*	Exenatide	150	41 (27.3)	109 (72.7)	USA
Dailey et al [94]^l^	2004	*Diabetes Care*	Insulin glulisine	876	748 (85.4)	128 (14.6)	North America and Australia
Zhu et al [95]^l^	2018	*Lancet Diabetes and Endocrinology*	Dorzagliatin	255	0 (0)	255 (100)	China

^a^251 participants were randomised but baseline characteristics are available for only 231 participants

^b^Ethnic breakdown was categorised as Qatari, Non-Qatari Arab, Asian Indian and Other. As a conservative assumption, it was assumed that the participants categorised as ‘Other’ were white, resulting in 16 white and 215 racialised participants

^c^4360 participants were randomised but nine of these participants did not receive the study medication

^d^808 participants were randomised but baseline characteristics are available for only 800 participants

^e^514 participants were randomised but baseline characteristics are available for only 491 participants who were included in the final analysis

^f^318 participants were randomised but demographic information is provided for only 316 participants

^g^1552 participants were randomised. One participant was untreated but demographic information for the untreated participant is not provided

^h^551 participants were randomised. Two participants were lost to follow-up after receiving the study drug and it is not known if they took at least one dose of the drug. For the purposes of data analysis, these participants were classified as untreated

^i^611 participants were randomised but 21 participants were excluded

^j^2425 participants were randomised but efficacy analyses and baseline characteristics are available for only 2418 participants

^k^677 participants were randomised but baseline characteristics are available for only 618 participants

^l^These trials were included only in the sensitivity analysis (ESM Appendix 2)

*NEJM*, *New England Journal of Medicine*

**Fig. 1:**
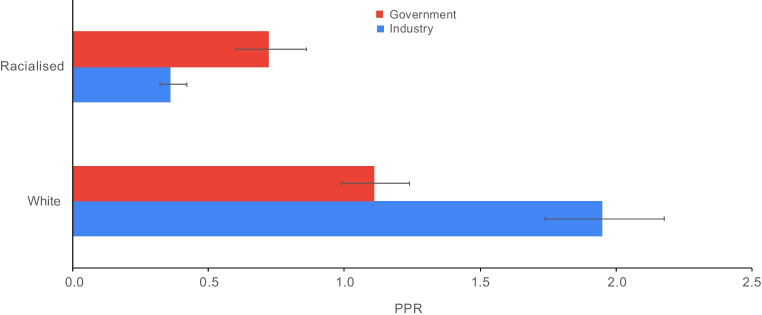
Representation of racialised and white participants in industry- and government-funded trials. Error bars represent 95% CIs

**Fig. 2:**
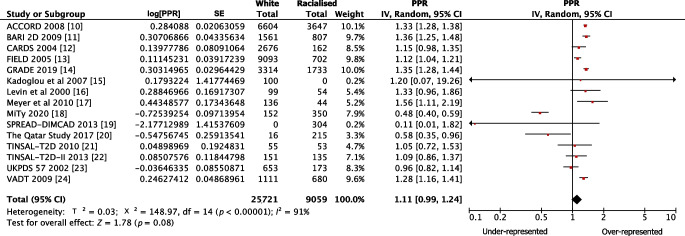
Forest plot showing PPRs for white participants in government-funded trials. Individual PPRs for 15 government-funded trials are shown along with a pooled PPR for the white population. Horizontal lines represent 95% CIs

**Fig. 3:**
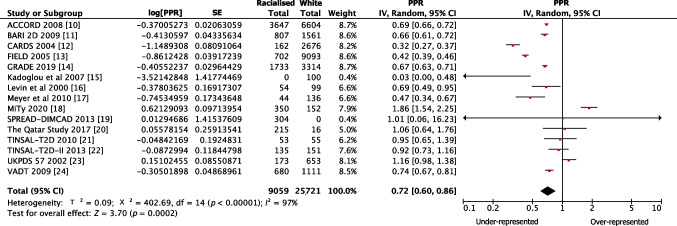
Forest plot showing PPRs for racialised participants in government-funded trials. Individual PPRs for 15 government-funded trials are shown along with a pooled PPR for the racialised population. Horizontal lines represent 95% CIs

**Fig. 4:**
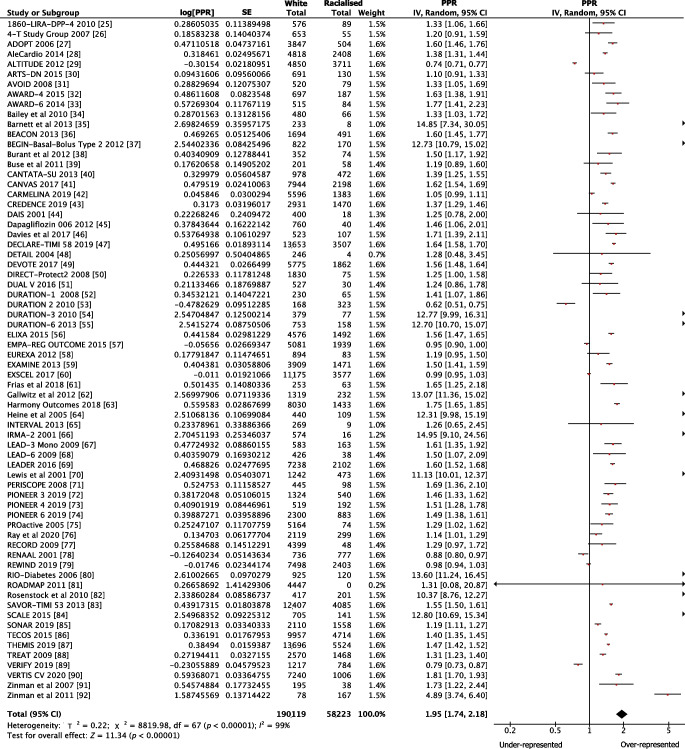
Forest plot showing PPRs for white participants in industry-funded trials. Individual PPRs for 68 industry-funded trials are shown along with a pooled PPR for the white population. Horizontal lines represent 95% CIs

**Fig. 5:**
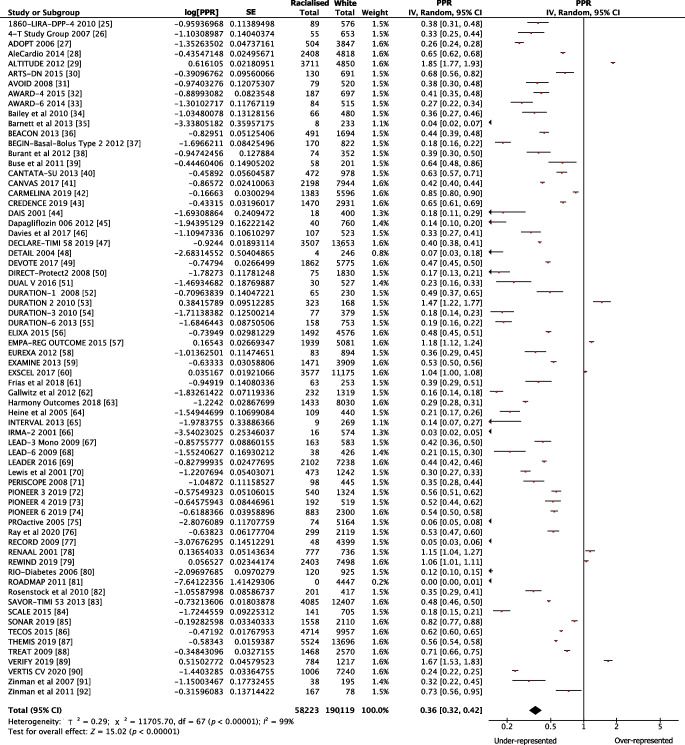
Forest plot showing PPRs for racialised participants in industry-funded trials. Individual PPRs for 68 industry-funded trials are shown along with a pooled PPR for the racialised population. Horizontal lines represent 95% CIs

The pooled PPR for white participants was 1.11 (95% CI 0.99, 1.24) for government-funded trials, consistent with proportional representation, and 1.95 (95% CI 1.74, 2.18) for industry-funded trials, consistent with over-representation. The PPR for racialised people was 0.72 (95% CI 0.60, 0.86) for government-funded trials and 0.36 (95% CI 0.32, 0.42) for industry-funded trials, both of which are consistent with under-representation (Fig. 1). Figures 2, 3, 4 and 5 show detailed breakdowns of the pooled PPRs.

The pooled meta-analytic estimates had high levels of heterogeneity (*I*^2^>90%). However, despite this, the results were directionally consistent. Only seven of 68 industry-funded trials had a PPR <1 for white people and a PPR >1 for racialised people [29, 53, 57, 60, 78, 79, 89], and these carried approximately 11.1% of the weight in the pooled estimates. Similarly, only four of 15 government-funded trials had a PPR <1 for white people and a PPR >1 for racialised people [18–20, 23], and these carried approximately 20.6% of the weight in the pooled estimates.

### Sensitivity analysis

Twelve industry-funded trials that recruited from three or more regions did not provide data on how many participants were recruited from each country or region (indicated by ‘Worldwide’ in Table 2). For these trials, worldwide estimates of the proportions of white and racialised people were used (11.7% and 88.3%, respectively) (ESM Methods). A series of sensitivity analyses were conducted in which the estimated proportions of white and racialised people were varied for these trials. The proportion of white participants was varied from 80% to 90% and of racialised participants from 10% to 20%. This did not result in appreciably different estimates from the main analyses. The full data for these sensitivity analyses are provided in ESM Appendix 1 (ESM Figs 2–5).

In addition to the 83 trials that were included in the main analysis, three trials that were funded by both government and industry sources were included in a separate sensitivity analysis [93–95]. Because a primary source of funding for these trials could not be determined with confidence, a sensitivity analysis was conducted to determine the effect that the trials would have on the pooled PPRs had they been included and categorised as industry or government funded. In one analysis both trials were included as industry-funded studies and in a second analysis both were included as government-funded trials. The results were not appreciably different from the main analyses (see ESM Appendix 2, ESM Figs 6 and 7).

## Discussion

Type 2 diabetes disproportionately affects racialised people worldwide [96]. This meta-epidemiological review shows that white individuals are over-enrolled and racialised individuals are under-enrolled in type 2 diabetes RCTs. Government-funded type 2 diabetes trials tend to have better representation of racialised participants than industry-funded trials.

The finding that government-funded trials recruit more racialised participants may reflect adherence to the more stringent conditions associated with funding from government bodies. For example, the NIH emphasises the inclusion and appropriate representation of minority groups in clinical research [4]. This measure attempts to ensure that racialised populations are proportionately represented in NIH-funded clinical research and that research findings are generalisable across all ethnic groups. Industry-funded trials do not have the same requirements as some government bodies and therefore decisions regarding who to enrol and in what proportion (i.e. by ethnicity, race and sex) are influenced entirely by the trial sponsors, steering committees and research staff. The under-representation of racialised participants limits the ability to generalise efficacy and safety outcomes to racialised people and may limit the uptake of this evidence by racialised communities, which adds to the disadvantages they may face.

For industry-funded trials, white participants were over-represented relative to their disease burden (Fig. 4). However, the pooled PPR estimate was heterogeneous. For example, some studies had PPR values that were notably higher than the others [35, 37, 54, 55, 62, 64, 66, 70, 80, 82, 84, 92]. This is because worldwide estimates were used for both type 2 diabetes prevalence and demographic data for these trials. Prevalence estimates were obtained from Saeedi et al [97] and are listed in ESM Table 3. Although the PPRs for white participants in industry-funded trials may be overestimates, the results of our sensitivity analyses do not suggest any substantial overestimation (ESM Appendix 1). This is because the proportion of racialised participants in these trials was relatively small and therefore varying the worldwide proportion estimates for racialised participants did not greatly affect the pooled PPR. Greater variance was observed among the different proportions of white participants in the trials. Overall, the effects of varying worldwide proportions of both white and racialised people on the PPRs were limited. This is because worldwide estimates were needed for only 12 of the 68 industry-funded trials and the participants in all 12 trials comprised only 3.8% of the total participants across the industry-funded trials in this review.

Racialised participants were under-represented in industry-funded trials relative to their disease burden (Fig. 5). Seven trials [29, 53, 57, 60, 78, 79, 89], however, had a PPR >1, with racialised PPRs of 1.85, 1.47, 1.18, 1.04, 1.15, 1.06, 1.67, respectively. Six of these trials recruited over 1500 participants from at least 24 countries including regions of North America, Europe, South America, Asia and Africa. Future industry-funded trials should consider enrolling participants from diverse communities and regions of the world, especially when the disease burden is higher than in white European-origin individuals.

There are several potential explanations for the patterns of over-representation of white participants and under-representation of racialised participants in government- and industry-funded RCTs. First, inclusion and exclusion criteria for RCTs may favour enrolling white over racialised participants. For example, in the USA, the ability to read and speak English is often an inclusion criterion for clinical trials, which can disproportionately impact the participation of racialised groups. Second, recruitment processes can affect the overall diversity of participants in RCTs. For instance, specialty clinics and hospitals where research is taking place may be located in areas with lower proportions of racialised people. Third, limited screening of racialised people for enrolment may occur because of implicit biases and/or social or medical reasons that make participation difficult for these groups. Fourth, mistrust and fear of medical institutions because of historical mistreatment of racialised groups may result in a lack of willingness of racialised people to participate in clinical trials. Fifth, racialised groups may not enrol because of language barriers, cultural practices or related contextual factors that limit their participation, including socioeconomic disadvantages. Finally, logistical barriers may exist such as inflexible work schedules and additional costs associated with participating in research studies, such as transport costs for attending study visits [98].

Furthermore, a lack of diversity among principal investigators, local investigators and study staff in some RCTs may also be related to lower enrolment rates for racialised participants [98]. For example, in an NIH study on the diversity of the NIH-funded workforce, it was found that 71.9% of principal investigators on NIH-funded research project grants were white [99]. Ethnically or racially diverse representation among study staff might increase the trust of participants from racialised communities and improve communication [98]. Additionally, industry-funded trials might benefit from having racialised participant recruitment guidelines similar to those that exist for the NIH. Regulatory bodies could indicate that proportional representation of participants affected by the disease of interest by ethnicity is strongly recommended or mandatory so that industry trial leaders more carefully consider who and from where to recruit within a given country or region. In future work, reviews of the recruitment of racialised groups should be carried out for trials conducted within single countries to assess country-specific trends. These reviews could guide clinical practice and be used to establish recruitment standards that are reasonable given the prevalence of type 2 diabetes and demographics in a given country. Future studies should also be conducted to analyse racialised participant recruitment trends in RCTs on other health conditions such as hypertension and stroke, which are known to be common in racialised communities.

Our study has several strengths. The meta-analysis included trials that were published in top-tier medical journals and that are likely to be highly cited and used to inform clinical guidelines. Analysing racialised participant recruitment in these studies allows for a finer assessment of the generalisability of the results to certain practice settings. The calculation and presentation of PPRs improves the summary of the findings of over-representation of white participants and under-representation of racialised participants (Figs 2, 3, 4 and 5).

Our study also has certain limitations. First, this analysis relied on investigator-reported ethnicity or race. Participants belonging to any ethnicity or race that was not explicitly defined by trial investigators as ‘white’ were categorised as ‘racialised’. We recognise, however, that the understanding of the terms ‘white’ and ‘racialised’ may vary between countries and trial investigators. Inconsistent interpretations of the term ‘white’ could influence the overall PPR estimates. Second, the PPR denominator calculations were based on prevalence and demographic data for white and racialised participants in different countries and regions of the world. While type 2 diabetes prevalence and demographic data are typically documented for specific countries, limited data exist for type 2 diabetes prevalence and demographics in larger regions of the world. When specific data were not available, these values were estimated (ESM Methods and ESM Table 3). Third, this meta-epidemiological review included studies that were published between 1 January 2000 and 31 December 2020. However, our estimates of type 2 diabetes prevalence and demographic data did not correspond exactly to the prevalence of type 2 diabetes in white and racialised people and the ethnic breakdown of countries or regions at the time that each trial was conducted, although we attempted to match them as closely as possible. Fourth, our pooled meta-analytic estimates had high levels of heterogeneity (*I*^2^ >90%). This heterogeneity may stem from many factors, including the study design used, country or region of conduct, study size and period of enrolment. Despite the high levels of heterogeneity, our results were consistent with respect to direction. Thus, statistical heterogeneity influenced the precision of our estimates but not the direction or magnitude to an extent that would cause concern. Finally, we did not conduct risk of bias assessments for the trials used in this review. This is because our goal was to examine recruitment of white and racialised participants as opposed to evaluating the clinical outcomes of included studies. Furthermore, our selection criteria were such that we only included large (*n*>100) RCTs (lowest risk of bias design) published in top-tier journals, which helped to ensure a comparable and low risk of bias across included studies.

### Conclusion

Racialised participants appear to be under-represented in both government- and industry-funded type 2 diabetes RCTs relative to their disease burden, while white participants appear to be over-represented in industry-funded trials. This meta-epidemiological review shows that the greatest disparity in ethnic and racial diversity in RCTs occurs in industry-funded trials. Strategies to improve the recruitment and enrolment of racialised participants into industry- and government-funded RCTs should be developed.

### Supplementary Information

Below is the link to the electronic supplementary material.

Supplementary file1 (PDF 1.28 MB)

## Data Availability

The authors confirm that the data supporting the findings of this study are available within the article and its supplementary materials. Demographic and prevalence data sourced from resources in the public domain are referenced in the ESM.

## Abbreviations

DPP-4: Dipeptidyl peptidase-4
GLP-1: Glucagon-like peptide-1
NIH: National Institutes of Health
PPR: Participation-to-prevalence ratio
SGLT-2: Sodium–glucose co-transporter 2

## Authors’ relationships and activities

SSA holds a Canada Research Chair in Ethnic Diversity and Cardiovascular Disease and the Heart and Stroke Michael G DeGroote Chair in Population Health. SSA also has received honoraria from Bayer, Janssen Pharmaceuticals, Novartis, and Daiichi Sankyo (consulting and speaker fees). RJdS has served as an external resource person to the WHO’s Nutrition Guidelines Advisory Group on trans fats, saturated fats and polyunsaturated fats. The WHO paid for his travel and accommodation to attend meetings from 2012 to 2017 to present and discuss this work. He presented updates of this work to the WHO in 2022. He has also carried out contract research for the Canadian Institutes of Health Research’s Institute of Nutrition, Metabolism and Diabetes, Health Canada and the WHO for which he received remuneration. He has received speaker’s fees from the University of Toronto and McMaster Children’s Hospital. He has held grants from the Canadian Institutes of Health Research, Canadian Foundation for Dietetic Research, Population Health Research Institute and Hamilton Health Sciences Corporation as a principal investigator and is a co-investigator on several funded team grants from the Canadian Institutes of Health Research. He has served as an independent director of the Helderleigh Foundation (Canada) and as a member of the Nutrition Science Advisory Committee to Health Canada (Government of Canada). He is a co-opted member of the Scientific Advisory Committee on Nutrition (SACN) Subgroup on the Framework for the Evaluation of Evidence (Public Health England).
