# Supplementary material for: Twenty years of participation of racialised groups in type 2 diabetes randomised clinical trials: a meta-epidemiological review
Source: Diabetologia. 2024 Jan 4;67(3):443–58. doi: 10.1007/s00125-023-06052-w (PMC10844363; doi:10.1007/s00125-023-06052-w)

**Electronic Supplementary Material (ESM)**

## ESM Methods

The participation to prevalence ratio (PPR) metric illustrates the representation of trial participants compared to the prevalence of type 2 diabetes in countries/regions stratified by ethnicity and race. The following approach was used to determine what prevalence and population demographic data should be used for PPRs:

- If the number of participants broken down by country is reported, the country of greatest recruitment is whichever country recruited the most participants. Country-specific prevalence and population data should be used where available.
- If the number of participants by country is not available, but reported by region, region-specific prevalence and population data may be used.
- If 2 regions are reported with no accompanying numbers of how many were recruited from each region, assume 50% recruitment from each and average prevalence and population data.
- If over 3 regions are reported with no accompanying numbers of how many were recruited from each region, use worldwide estimates.

When a study recruited predominantly from the United States (US) (35) or United Kingdom (UK) (2), country-specific prevalence and demographic data was used to determine the denominator for the participation-to-prevalence ratio. Country-specific demographic data was also used for trials with Argentina (1), Australia (1), Qatar (1), Canada (1), and China (1) as the country with the greatest participant recruitment.

To determine the expected number of diabetes cases for white participants in a study, prevalence and demographic data was used. For studies that recruited the most participants from the US, an estimate for white diabetes prevalence for the US from the Centre of Disease Control and Prevention website was used. This value was multiplied by the percentage of the US that is comprised of white people (retrieved from the US Census website). Then, the white prevalence percentage was multiplied by the total number of white people in the US to get an estimate of how many white people have diabetes in the US. The expected number of white cases was

determined by dividing the number of white people with diabetes in the US by the total number of people with diabetes in the US (white and racialised).

Similarly, to determine the expected number of diabetes cases among racialised people in the US, the racialised diabetes prevalence value was multiplied by the percentage of the US that is comprised of racialised people. Then, the racialised prevalence percentage was multiplied by the total number of racialised people in the US to get an estimate of how many racialised people have diabetes in the US. The expected number of racialised cases was found by dividing the number of racialised people with diabetes in the US by the total number of people with diabetes in the US (racialised and white).

For trials that specified the region of greatest recruitment, regional prevalence estimates were used. Diabetes prevalence data for the remaining trials was estimated using Table 1 from Saeedi et al [97]. The study defines confidence intervals for the prevalence of diabetes in IDF regions. For the white and racialised diabetes prevalence estimates, the lower bound of the confidence interval was taken to represent the white prevalence, and the upper bound of the confidence interval represented the racialised prevalence. This was a reasonable approach for estimation given that the greatest prevalence of T2DM is observed in racialised ethnic groups. For trials that provided no indication of how many participants were recruited from each region (12), worldwide prevalence estimates from Saeedi et al. were used (ESM Table 3).

When demographic data about ethnicity was unavailable for regions (Europe, North America, Worldwide), it was estimated. For trials that recruited primarily from Europe (13), demographic data from the UK was used. For trials that recruited the most participants from Bulgaria (1), Serbia (1), Slovakia (1), Greece (1), and the Russian Federation (1), UK demographic data was used for estimates. For North American trials (5), demographic data from the US and Canada was combined and averaged. For trials with no participant recruitment data by region/country, the proportion of white people worldwide (11.7%) was estimated by multiplying the white population proportions for North America, Europe, and Australia by the population in those regions in 2020 to match the timeframe of our data collection. This calculation made it possible to determine a worldwide racialised population proportion estimate (88.3%).

For trials that recruited only from North America and Europe (4), South America and North America (1), and Europe and USA (1), it was assumed that 50% of the recruitment was

from each respective region. As such, the prevalence values for each region were averaged and the total population of each region was added to calculate the PPR.

For trials that had 0 white participants or 0 racialised participants, a 0.5 correction was used in place of “0” for calculation purposes.

**ESM Table 1. Ovid Medline Search Strategy**

| N | Search Terms |
|---|---|
| 1 | jama.jn |
| 2 | lancet.jn |
| 3 | british medical journal.jn |
| 4 | “new england journal of medicine”.jn |
| 5 | or/1-4 |
| 6 | circulation.jn |
| 7 | “annals of internal medicine”.jn |
| 8 | diabetes care.jn |
| 9 | or/6-8 |
| 10 | lancet diabetes & endocrinology.jn |
| 11 | jama cardiology.jn |
| 12 | jama internal medicine.jn |
| 13 | jama ophthalmology.jn |
| 14 | or/10-13 |
| 15 | exp Diabetes Mellitus, Type 2/ |
| 16 | ((mature onset or slow onset or non-insulin dependent or ketosis resistant) adj2 diabet*).ti,ab,kf,kw. |
| 17 | (NIDDM or NIDD or MODY or T2DM or T2D).ti,ab,kf,kw. |
| 18 | or/15-17 |
| 19 | random*.ti,ab,kf,kw. |
| 20 | rct.ti,ab,kf,kw. |
| 21 | random Allocation/ |
| 22 | randomized Controlled Trial/ |
| 23 | ((single or double or triple or treble) adj2 (blind* or mask* or conceal*)).ti,ab,kf,kw. |
| 24 | randomized controlled trial.pt. |
| 25 | or/19-24 |
| 26 | 18 and 25 |
| 27 | 14 and 26 |
| 28 | limit 27 to yr=”2000 – 2020” |
| 29 | 9 and 26 |
| 30 | limit 29 to yr=”2000-2020” |
| 31 | 5 and 26 |
| 32 | limit 31 to yr=”2000-2020” |
| 33 | 28 or 30 or 32 |
| 34 | remove duplicates from 33 |
| 35 | systematic review.pt. |
| 36 | comment.pt. |
| 37 | editorial.pt. |
| 38 | meta analysis.pt. |
| 39 | or/35-38 |
| 40 | 34 not 39 |

**ESM Table 2. Study Selection Criteria**

| Criterion | Industry | Government |
|---|---|---|
| Described the results of an RCT of a T2DM pharmacotherapy | ✓ | ✓ |
| Published in *NEJM, JAMA, The Lancet, BMJ, or Annals of Internal Medicine* | ✓ | ✓ |
| Published in a specialty journal (*Diabetes Care*, *Circulation*, *Lancet Diabetes and Endocrinology*, *JAMA Internal Medicine*, *JAMA Ophthalmology*) | | ✓ |
| Sample size of at least 100 participants[a] | ✓ | ✓ |
| All participants were adults with T2DM | ✓ | ✓ |
| Participants enrolled from at least two countries[b] | ✓ | |
| Published between 1 January 2000-31 December 2020 | ✓ | ✓ |
| English language | ✓ | ✓ |

a) A sample size of at least 100 participants was required to help ensure the selection of the most robust findings and avoid small study bias.
b) Government trials were not held to the same multi-country standard as industry trials because they are typically conducted within a single country.

**ESM Table 3. Estimates used for PPR Calculations**

| Variable | Estimate (%) of Population | Source |
|---|---|---|
| T2DM Prevalence in US | White: 7.5<br>Racialised: 12.0 | Centers for Disease Control and Prevention. National Diabetes Statistics Report, 2020. Atlanta, GA: Centers for Disease Control and Prevention, U.S. Dept of Health and Human Services; 2020. |
| Prevalence in UK | White: 5.0<br>Racialised: 6.8 | Pham TM, Carpenter JR, Morris TP, Sharma M, Petersen I. Ethnic Differences in the Prevalence of Type 2 Diabetes Diagnoses in the UK: Cross-Sectional Analysis of the Health Improvement Network Primary Care Database. *Clin Epidemiol*. 2019;11:1081-1088. doi:10.2147/CLEP.S227621 |
| Prevalence in Australia | White: 8.3<br>Racialised: 15.6 | Saeedi et al. (2019) – used estimates for WP |
| Prevalence in Argentina | White: 6.7<br>Racialised: 11.3 | Saeedi et al. (2019) – used estimates for SACA |
| Global Prevalence | White: 6.2<br>Racialised: 11.8 | Saeedi et al. (2019) – used estimates for world |
| North America Prevalence | White: 9.0<br>Racialised: 14.5 | Saeedi et al. (2019) – used estimates for NAC |
| Canada Prevalence | White: 9.0<br>Racialised: 14.5 | Saeedi et al. (2019) – used estimates for NAC |
| Europe Prevalence | White: 4.9<br>Racialised: 9.2 | Saeedi et al. (2019) – used estimates for EUR |
| Prevalence in Qatar | White: 5.0 (UK estimate)<br>Racialised: 7.0 | Awad SF, A Toumi A, A Al-Mutawaa K*, et al*<br>Type 2 diabetes epidemic and key risk factors in Qatar: a mathematical modeling analysis<br>*BMJ Open Diabetes Research and Care* 2022;10:e002704. doi: 10.1136/bmjdrc-2021-002704 |
| Prevalence in China | White: 8.3<br>Racialised: 8.6 | Saeedi et al. (2019) – used estimate for white population in the Western Pacific region<br>Yuan, H., Li, X., Wan, G., Sun, L., Zhu, X., Che, F., & Yang, Z. (2017). Type 2 diabetes epidemic in East Asia: a 35-year systematic trend analysis. *Oncotarget*, *9*(6), 6718–6727. https://doi.org/10.18632/oncotarget.22961 - used estimate for racialised population |

| | | |
|---|---|---|
| Prevalence in Greece | White: 7.4<br>Racialised: 9.2 (Europe estimate) | Lalagkas, P.-N., Polyzois, S., Papanas, N., Nena, E., Vourli, N., Kontogiorgis, C., & Constantinides, T. (2022). Prevalence of pharmacologically treated type 2 diabetes mellitus in 2012–2016 in Greece: Real-World Data. *Primary Care Diabetes*, *16*(5), 714–716. https://doi.org/10.1016/j.pcd.2022.07.001 |
| Prevalence in Bulgaria, Serbia, Russian Federation, Slovakia | White: 4.9<br>Racialised: 9.2 | Saeedi et al. (2019) – used estimates for EUR |
| Proportion of the population (US) | White: 60.1<br>Racialised: 39.9 | US Census Bureau. (2019). https://www.census.gov/quickfacts/fact/table/US/PST045219 |
| Proportion of the population (UK) | White: 86.0<br>Racialised: 14.0 | UK Census data, Government of the United Kingdom. (2011). https://www.ons.gov.uk/census/2011census |
| Proportion of the population (Australia) | White: 90.2<br>Racialised: 9.8 | People of Australia. Britannica. (2007). https://www.britannica.com/place/Australia |
| Proportion of the population (Argentina) | White: 85.0<br>Racialised: 15.0 | Fernandez FL. Composición Étnica de las Tres Áreas Culturales del Continente Americano al Comienzo del Siglo XXI" (PDF) (in Spanish). Centro de Investigación en Ciencias Sociales y Humanidades, UAEM. 2005. |
| Proportion of the population (Canada) | White: 72.9<br>Racialised: 27.1 | Statistics Canada 2016 |
| Proportion of the population (North America) | White: 66.5<br>Racialised: 33.5 | Average of the percentage of white people in Canada, 72.9 (Statistics Canada 2016), and in the US, 60.1 (US Census Bureau 2019) |
| Proportion of the population (Europe) | White: 86.0<br>Racialised: 14.0 | UK census data estimates were used for Europe estimates. |
| Proportion of the population (Qatar) | White: 16.0<br>Racialised: 84.0 | https://worldpopulationreview.com/countries/qatar-population |
| Proportion of the population (China) | White: 98.5<br>Racialised: 1.5 | Conservative estimate given that the 2020 Chinese Census showed that 8.89% of the country was comprised of minorities, of which the majority are racialised groups. |
| Proportion of the population (Greece) | White: 86.0<br>Racialised: 14.0 | UK census data was used as an estimate because data on ethnicity collected by Greece represents citizenship. |
| Proportion of the population (Global) | White: 11.7%<br>Racialised: 88.3% | White population proportions:<br>• North America: 66.5%<br>• Europe: 86.0% |

| | | |
|---|---|---|
| | | • Australia: 90.2%<br><br>Proportions were multiplied by the population in those regions in 2020 (source: Population Reference Bureau https://www.prb.org/wp-content/uploads/2020/07/letter-booklet-2020-world-population.pdf) |

## ESM Table 4. List of Excluded Studies After Full Text Review

| **Ancillary Publication** | **48** |
|---|---|
| Steinberg 2017 Amylase, Lipase, and Acute Pancreatitis in People With Type 2 Diabetes Treated With Liraglutide: Results From the LEADER Randomized Trial. Steinberg, William M; Buse, John B; Ghorbani, Marie Louise Muus; Orsted, David D; Nauck, Michael A; LEADER Steering Committee; LEADER Trial Investigators Diabetes care / 2017;40(7):966-972 | Ancillary Publication |
| Scirica 2014 Heart failure, saxagliptin, and diabetes mellitus: observations from the SAVOR-TIMI 53 randomized trial. Scirica, Benjamin M; Braunwald, Eugene; Raz, Itamar; Cavender, Matthew A; Morrow, David A; Jarolim, Petr; Udell, Jacob A; Mosenzon, Ofri; Im, KyungAh; Umez-Eronini, Amarachi A; Pollack, Pia S; Hirshberg, Boaz; Frederich, Robert; Lewis, Basil S; McGuire, Darren K; Davidson, Jaime; Steg, Ph Gabriel; Bhatt, Deepak L; SAVOR-TIMI 53 Steering Committee and Investigators* Circulation / 2014;130(18):1579-88 | Ancillary Publication |
| Mosenzon 2017 Effect of Saxagliptin on Renal Outcomes in the SAVOR-TIMI 53 Trial. Mosenzon, Ofri; Leibowitz, Gil; Bhatt, Deepak L; Cahn, Avivit; Hirshberg, Boaz; Wei, Cheryl; Im, KyungAh; Rozenberg, Aliza; Yanuv, Ilan; Stahre, Christina; Ray, Kausik K; Iqbal, Nayyar; Braunwald, Eugene; Scirica, Benjamin M; Raz, Itamar Diabetes care / 2017;40(1):69-76 | Ancillary Publication |
| Ting 2012 Benefits and safety of long-term fenofibrate therapy in people with type 2 diabetes and renal impairment: the FIELD Study. Ting, Ru-Dee; Keech, Anthony C; Drury, Paul L; Donoghoe, Mark W; Hedley, John; Jenkins, Alicia J; Davis, Timothy M E; Lehto, Seppo; Celermajer, David; Simes, R John; Rajamani, Kushwin; Stanton, Kim; FIELD Study Investigators Diabetes care / 2012;35(2):218-25 | Ancillary Publication |
| Gerstein 2014 Effects of intensive glycaemic control on ischaemic heart disease: analysis of data from the randomised, controlled ACCORD trial. Gerstein, Hertzel C; Miller, Michael E; Ismail-Beigi, Faramarz; Largay, Joe; McDonald, Charlotte; Lochnan, Heather A; Booth, Gillian L; ACCORD Study Group Lancet (London, England) / 2014;384(9958):1936-41 England 2014 / | Ancillary Publication |
| Anderson 2014 Blood pressure and pulse pressure effects on renal outcomes in the Veterans Affairs Diabetes Trial (VADT). Anderson, Robert J; Bahn, Gideon D; Emanuele, Nicholas V; Marks, Jennifer B; Duckworth, William C; VADT Study Group Diabetes care / 2014;37(10):2782-8 | Ancillary Publication |
| Siegelaar 2011 A decrease in glucose variability does not reduce cardiovascular event rates in type 2 diabetic patients after acute myocardial infarction: a reanalysis of the HEART2D study. Siegelaar, Sarah E; Kerr, Lisa; Jacober, Scott J; Devries, J Hans Diabetes care / 2011;34(4):855-7 | Ancillary Publication |
| Althouse 2014 Risk factors for incident peripheral arterial disease in type 2 diabetes: results from the Bypass Angioplasty Revascularization Investigation in type 2 Diabetes (BARI 2D) Trial. Althouse, Andrew D; Abbott, J Dawn; Forker, Alan D; Bertolet, Marnie; Barinas-Mitchell, Emma; Thurston, Rebecca C; Mulukutla, Suresh; Aboyans, Victor; Brooks, Maria Mori; BARI 2D Study Group Diabetes care / 2014;37(5):1346-52 | Ancillary Publication |
| Tian 2020 Effects of Intensive Glycemic Control on Clinical Outcomes Among Patients With Type 2 Diabetes With Different Levels of Cardiovascular Risk and Hemoglobin A1c in the ADVANCE Trial. Tian, Jingyan; Ohkuma, Toshiaki; Cooper, Mark; Harrap, Stephen; Mancia, Giuseppe; Poulter, Neil; Wang, Ji-Guang; Zoungas, Sophia; Woodward, Mark; Chalmers, John Diabetes care / 2020;43(6):1293-1299 | Ancillary Publication |
| Furtado 2019 Dapagliflozin and Cardiovascular Outcomes in Patients With Type 2 Diabetes Mellitus and Previous Myocardial Infarction. Furtado, Remo H M; Bonaca, Marc P; Raz, Itamar; Zelniker, Thomas A; Mosenzon, Ofri; Cahn, Avivit; Kuder, Julia; Murphy, Sabina A; Bhatt, Deepak L; Leiter, Lawrence A; McGuire, Darren K; Wilding, John P H; Ruff, Christian T; Nicolau, Jose C; Gause-Nilsson, Ingrid A M; Fredriksson, Martin; Langkilde, Anna Maria; Sabatine, Marc S; Wiviott, Stephen D Circulation / 2019;139(22):2516-2527 | Ancillary Publication |
| Cornel 2016 Effect of Sitagliptin on Kidney Function and Respective Cardiovascular Outcomes in Type 2 Diabetes: Outcomes From TECOS. Cornel, Jan H; Bakris, George L; Stevens, Susanna R; Alvarsson, Michael; Bax, Willem A; Chuang, Lee-Ming; Engel, Samuel S; Lopes, Renato D; McGuire, Darren K; Riefflin, Axel; Rodbard, Helena Wachslicht; Sinay, Isaac; Tankova, Tsvetalina; Wainstein, Julio; Peterson, Eric D; Holman, Rury R; TECOS Study Group Diabetes care / 2016;39(12):2304-2310 | Ancillary Publication |

| | |
|---|---|
| Zhou 2018 Glycemic Variation and Cardiovascular Risk in the Veterans Affairs Diabetes Trial. Zhou, Jin J; Schwenke, Dawn C; Bahn, Gideon; Reaven, Peter; VADT Investigators Diabetes care / 2018;41(10):2187-2194 | Ancillary Publication |
| Kato 2019 Effect of Dapagliflozin on Heart Failure and Mortality in Type 2 Diabetes Mellitus. Kato, Eri T; Silverman, Michael G; Mosenzon, Ofri; Zelniker, Thomas A; Cahn, Avivit; Furtado, Remo H M; Kuder, Julia; Murphy, Sabina A; Bhatt, Deepak L; Leiter, Lawrence A; McGuire, Darren K; Wilding, John P H; Bonaca, Marc P; Ruff, Christian T; Desai, Akshay S; Goto, Shinya; Johansson, Peter A; Gause-Nilsson, Ingrid; Johanson, Per; Langkilde, Anna Maria; Raz, Itamar; Sabatine, Marc S; Wiviott, Stephen D Circulation / 2019;139(22):2528-2536 | Ancillary Publication |
| Docherty 2020 Effect of Dapagliflozin in DAPA-HF According to Background Glucose-Lowering Therapy. Docherty, Kieran F; Jhund, Pardeep S; Bengtsson, Olof; DeMets, David L; Inzucchi, Silvio E; Kober, Lars; Kosiborod, Mikhail N; Langkilde, Anna Maria; Martinez, Felipe A; Sabatine, Marc S; Sjostrand, Mikaela; Solomon, Scott D; McMurray, John J V; DAPA-HF Investigators and Committees Diabetes care / 2020;43(11):2878-2881 | Ancillary Publication |
| Bach 2013 Rosiglitazone and outcomes for patients with diabetes mellitus and coronary artery disease in the Bypass Angioplasty Revascularization Investigation 2 Diabetes (BARI 2D) trial. Bach, Richard G; Brooks, Maria Mori; Lombardero, Manuel; Genuth, Saul; Donner, Thomas W; Garber, Alan; Kennedy, Laurence; Monrad, E Scott; Pop-Busui, Rodica; Kelsey, Sheryl F; Frye, Robert L; BARI 2D Investigators Circulation / 2013;128(8):785-94 | Ancillary Publication |
| Tanamas 2016 Long-term Effect of Losartan on Kidney Disease in American Indians With Type 2 Diabetes: A Follow-up Analysis of a Randomized Clinical Trial. Tanamas, Stephanie K; Saulnier, Pierre-Jean; Fufaa, Gudeta D; Wheelock, Kevin M; Weil, E Jennifer; Hanson, Robert L; Knowler, William C; Bennett, Peter H; Nelson, Robert G Diabetes care / 2016;39(11):2004-2010 | Ancillary Publication |
| Verma 2018 Cardiovascular Outcomes and Safety of Empagliflozin in Patients With Type 2 Diabetes Mellitus and Peripheral Artery Disease: A Subanalysis of EMPA-REG OUTCOME. Verma, Subodh; Mazer, C David; Al-Omran, Mohammed; Inzucchi, Silvio E; Fitchett, David; Hehnke, Uwe; George, Jyothis T; Zinman, Bernard Circulation / 2018;137(4):405-407 | Ancillary Publication |
| McGuire 2019 Linagliptin Effects on Heart Failure and Related Outcomes in Individuals With Type 2 Diabetes Mellitus at High Cardiovascular and Renal Risk in CARMELINA. McGuire, Darren K; Alexander, John H; Johansen, Odd Erik; Perkovic, Vlado; Rosenstock, Julio; Cooper, Mark E; Wanner, Christoph; Kahn, Steven E; Toto, Robert D; Zinman, Bernard; Baanstra, David; Pfarr, Egon; Schnaidt, Sven; Meinicke, Thomas; George, Jyothis T; von Eynatten, Maximilian; Marx, Nikolaus; CARMELINA Investigators Circulation / 2019;139(3):351-361 | Ancillary Publication |
| Holman 2009 Three-year efficacy of complex insulin regimens in type 2 diabetes. Holman, Rury R; Farmer, Andrew J; Davies, Melanie J; Levy, Jonathan C; Darbyshire, Julie L; Keenan, Joanne F; Paul, Sanjoy K; 4-T Study Group The New England journal of medicine / 2009;361(18):1736-47 | Ancillary Publication |
| Bonaca 2020 Dapagliflozin and Cardiac, Kidney, and Limb Outcomes in Patients With and Without Peripheral Artery Disease in DECLARE-TIMI 58. Bonaca, Marc P; Wiviott, Stephen D; Zelniker, Thomas A; Mosenzon, Ofri; Bhatt, Deepak L; Leiter, Lawrence A; McGuire, Darren K; Goodrich, Erica L; De Mendonca Furtado, Remo Holanda; Wilding, John P H; Cahn, Avivit; Gause-Nilsson, Ingrid A M; Johanson, Per; Fredriksson, Martin; Johansson, Peter A; Langkilde, Anna Maria; Raz, Itamar; Sabatine, Marc S Circulation / 2020;142(8):734-747 | Ancillary Publication |
| Blevins 2020 Randomized Double-Blind Clinical Trial Comparing Ultra Rapid Lispro With Lispro in a Basal-Bolus Regimen in Patients With Type 2 Diabetes: PRONTO-T2D. Blevins, Thomas; Zhang, Qianyi; Frias, Juan P; Jinnouchi, Hideaki; Chang, Annette M; PRONTO-T2D Investigators Diabetes care / 2020;43(12):2991-2998 | Ancillary Publication |
| Tripathy 2014 Baseline adiponectin levels do not influence the response to pioglitazone in ACT NOW. Tripathy, Devjit; Clement, Stephen C; Schwenke, Dawn C; Banerji, MaryAnn; Bray, George A; Buchanan, Thomas A; Gastaldelli, Amalia; Henry, Robert R; Kitabchi, Abbas E; Mudaliar, Sunder; Ratner, Robert E; Stentz, Frankie B; Musi, Nicolas; Reaven, Peter D; DeFronzo, Ralph A Diabetes care / 2014;37(6):1706-11 | Ancillary Publication |
| Keech 2007 Effect of fenofibrate on the need for laser treatment for diabetic retinopathy (FIELD study): a randomised controlled trial. Keech, A C; Mitchell, P; Summanen, P A; O'Day, J; Davis, | Ancillary Publication |

| | |
|---|---|
| T M E; Moffitt, M S; Taskinen, M-R; Simes, R J; Tse, D; Williamson, E; Merrifield, A; Laatikainen, L T; d'Emden, M C; Crimet, D C; O'Connell, R L; Colman, P G; FIELD study investigators Lancet (London, England) / 2007;370(9600):1687-97 England 2007 / | |
| Cannon 2020 Evaluating the Effects of Canagliflozin on Cardiovascular and Renal Events in Patients With Type 2 Diabetes Mellitus and Chronic Kidney Disease According to Baseline HbA1c, Including Those With HbA1c <7%: Results From the CREDENCE Trial. Cannon, Christopher P; Perkovic, Vlado; Agarwal, Rajiv; Baldassarre, James; Bakris, George; Charytan, David M; de Zeeuw, Dick; Edwards, Robert; Greene, Tom; Heerspink, Hiddo J L; Jardine, Meg J; Levin, Adeera; Li, Jing-Wei; Neal, Bruce; Pollock, Carol; Wheeler, David C; Zhang, Hong; Zinman, Bernard; Mahaffey, Kenneth W Circulation / 2020;141(5):407-410 | Ancillary Publication |
| Cahn 2016 Predisposing Factors for Any and Major Hypoglycemia With Saxagliptin Versus Placebo and Overall: Analysis From the SAVOR-TIMI 53 Trial. Cahn, Avivit; Raz, Itamar; Mosenzon, Ofri; Leibowitz, Gil; Yanuv, Ilan; Rozenberg, Aliza; Iqbal, Nayyar; Hirshberg, Boaz; Sjostrand, Mikaela; Stahre, Christina; Im, KyungAh; Kanevsky, Estella; Scirica, Benjamin M; Bhatt, Deepak L; Braunwald, Eugene Diabetes care / 2016;39(8):1329-37 | Ancillary Publication |
| Biessels 2019 Effect of Linagliptin on Cognitive Performance in Patients With Type 2 Diabetes and Cardiorenal Comorbidities: The CARMELINA Randomized Trial. Biessels, Geert Jan; Verhagen, Chloe; Janssen, Jolien; van den Berg, Esther; Zinman, Bernard; Rosenstock, Julio; George, Jyothis T; Passera, Anna; Schnaidt, Sven; Johansen, Odd Erik; CARMELINA Investigators Diabetes care / 2019;42(10):1930-1938 | Ancillary Publication |
| Skov 2014 Metformin, but not rosiglitazone, attenuates the increasing plasma levels of a new cardiovascular marker, fibulin-1, in patients with type 2 diabetes. Skov, Vibe; Cangemi, Claudia; Gram, Jeppe; Christensen, Mette M; Grodum, Ellen; Sorensen, Ditte; Argraves, W Scott; Henriksen, Jan E; Rasmussen, Lars M Diabetes care / 2014;37(3):760-6 | Ancillary Publication |
| Erdmann 2007 Pioglitazone use and heart failure in patients with type 2 diabetes and preexisting cardiovascular disease: data from the PROactive study (PROactive 08). Erdmann, Erland; Charbonnel, Bernard; Wilcox, Robert G; Skene, Allan M; Massi-Benedetti, Massimo; Yates, John; Tan, Meng; Spanheimer, Robert; Standl, Eberhard; Dormandy, John A; PROactive Investigators Diabetes care / 2007;30(11):2773-8 | Ancillary Publication |
| Zannad 2015 Heart failure and mortality outcomes in patients with type 2 diabetes taking alogliptin versus placebo in EXAMINE: a multicentre, randomised, double-blind trial. Zannad, Faiez; Cannon, Christopher P; Cushman, William C; Bakris, George L; Menon, Venu; Perez, Alfonso T; Fleck, Penny R; Mehta, Cyrus R; Kupfer, Stuart; Wilson, Craig; Lam, Hung; White, William B; EXAMINE Investigators Lancet (London, England) / 2015;385(9982):2067-76 England 2015 / | Ancillary Publication |
| Pratley 2012 Efficacy and safety of switching from the DPP-4 inhibitor sitagliptin to the human GLP-1 analog liraglutide after 52 weeks in metformin-treated patients with type 2 diabetes: a randomized, open-label trial. Pratley, Richard E; Nauck, Michael A; Bailey, Timothy; Montanya, Eduard; Filetti, Sebastiano; Garber, Alan J; Thomsen, Anne B; Furber, Sabina; Davies, Melanie; 1860-LIRA-DPP-4 Study Group Diabetes care / 2012;35(10):1986-93 | Ancillary Publication |
| Wanner 2016 Empagliflozin and Progression of Kidney Disease in Type 2 Diabetes. Wanner, Christoph; Inzucchi, Silvio E; Lachin, John M; Fitchett, David; von Eynatten, Maximilian; Mattheus, Michaela; Johansen, Odd Erik; Woerle, Hans J; Broedl, Uli C; Zinman, Bernard; EMPA-REG OUTCOME Investigators The New England journal of medicine / 2016;375(4):323-34 | Ancillary Publication |
| Verma 2018 Effect of Liraglutide on Cardiovascular Events in Patients With Type 2 Diabetes Mellitus and Polyvascular Disease: Results of the LEADER Trial. Verma, Subodh; Bhatt, Deepak L; Bain, Stephen C; Buse, John B; Mann, Johannes F E; Marso, Steven P; Nauck, Michael A; Poulter, Neil R; Pratley, Richard E; Zinman, Bernard; Michelsen, Marie M; Monk Fries, Tea; Rasmussen, Soren; Leiter, Lawrence A; LEADER Publication Committee on behalf of the LEADER Trial Investigators Circulation / 2018;137(20):2179-2183 | Ancillary Publication |
| Sivitz 2020 Optimization of Metformin in the GRADE Cohort: Effect on Glycemia and Body Weight. Sivitz, William I; Phillips, Lawrence S; Wexler, Deborah J; Fortmann, Stephen P; Camp, Anne W; Tiktin, Margaret; Perez, Magalys; Craig, Jacqueline; Hollander, Priscilla A; | Ancillary Publication |

| | |
|---|---|
| Cherrington, Andrea; Aroda, Vanita R; Tan, Meng Hee; Krakoff, Jonathan; Rasouli, Neda; Butera, Nicole M; Younes, Naji; GRADE Research Group Diabetes care / 2020;43(5):940-947 | |
| Mahaffey 2018 Canagliflozin for Primary and Secondary Prevention of Cardiovascular Events: Results From the CANVAS Program (Canagliflozin Cardiovascular Assessment Study). Mahaffey, Kenneth W; Neal, Bruce; Perkovic, Vlado; de Zeeuw, Dick; Fulcher, Greg; Erondu, Ngozi; Shaw, Wayne; Fabbrini, Elisa; Sun, Tao; Li, Qiang; Desai, Mehul; Matthews, David R; CANVAS Program Collaborative Group Circulation / 2018;137(4):323-334 | Ancillary Publication |
| Ismail-Beigi 2010 Effect of intensive treatment of hyperglycaemia on microvascular outcomes in type 2 diabetes: an analysis of the ACCORD randomised trial. Ismail-Beigi, Faramarz; Craven, Timothy; Banerji, Mary Ann; Basile, Jan; Calles, Jorge; Cohen, Robert M; Cuddihy, Robert; Cushman, William C; Genuth, Saul; Grimm, Richard H Jr; Hamilton, Bruce P; Hoogwerf, Byron; Karl, Diane; Katz, Lois; Krikorian, Armand; O'Connor, Patrick; Pop-Busui, Rodica; Schubart, Ulrich; Simmons, Debra; Taylor, Harris; Thomas, Abraham; Weiss, Daniel; Hramiak, Irene; ACCORD trial group Lancet (London, England) / 2010;376(9739):419-30 England 2010 / | Ancillary Publication |
| Pitale 2000 Two years of intensive glycemic control and left ventricular function in the Veterans Affairs Cooperative Study in Type 2 Diabetes Mellitus (VA CSDM). Pitale, S U; Abraira, C; Emanuele, N V; McCarren, M; Henderson, W G; Pacold, I; Bushnell, D; Colwell, J A; Nuttall, F Q; Levin, S R; Sawin, C T; Comstock, J P; Silbert, C K Diabetes care / 2000;23(9):1316-20 | Ancillary Publication |
| Vakkilainen 2002 Fenofibrate lowers plasma triglycerides and increases LDL particle diameter in subjects with type 2 diabetes. Vakkilainen, Juha; Steiner, George; Ansquer, Jean-Claude; Perttunen-Nio, Helina; Taskinen, Marja-Riitta Diabetes care / 2002;25(3):627-8 | Ancillary Publication |
| Chan 2004 Renin angiotensin aldosterone system blockade and renal disease in patients with type 2 diabetes. An Asian perspective from the RENAAL Study. Chan, Juliana C N; Wat, Nelson M S; So, Wing-Yee; Lam, Karen S L; Chua, Chin-Teong; Wong, Kok-Seng; Morad, Zaki; Dickson, Tania Z; Hille, Darcy; Zhang, Zhongxin; Cooper, Mark E; Shahinfar, Shahnaz; Brenner, Barry M; Kurokawa, Kiyoshi; Asian RENAAL Study Investigators Diabetes care / 2004;27(4):874-9 | Ancillary Publication |
| Bhatt 2019 Ticagrelor in patients with diabetes and stable coronary artery disease with a history of previous percutaneous coronary intervention (THEMIS-PCI): a phase 3, placebo-controlled, randomised trial. Bhatt, Deepak L; Steg, Philippe Gabriel; Mehta, Shamir R; Leiter, Lawrence A; Simon, Tabassome; Fox, Kim; Held, Claes; Andersson, Marielle; Himmelmann, Anders; Ridderstrale, Wilhelm; Chen, Jersey; Song, Yang; Diaz, Rafael; Goto, Shinya; James, Stefan K; Ray, Kausik K; Parkhomenko, Alexander N; Kosiborod, Mikhail N; McGuire, Darren K; Harrington, Robert A; THEMIS Steering Committee and Investigators Lancet (London, England) / 2019;394(10204):1169-1180 England 2019 / | Ancillary Publication |
| Azad 2016 Association of Blood Glucose Control and Lipids With Diabetic Retinopathy in the Veterans Affairs Diabetes Trial (VADT). Azad, Nasrin; Bahn, Gideon D; Emanuele, Nicholas V; Agrawal, Lily; Ge, Ling; Reda, Dominic; Klein, Ronald; Reaven, Peter D; Hayward, Rodney; VADT Study Group Diabetes care / 2016;39(5):816-22 1 | Ancillary Publication |
| Hempe 2015 The hemoglobin glycation index identifies subpopulations with harms or benefits from intensive treatment in the ACCORD trial. Hempe, James M; Liu, Shuqian; Myers, Leann; McCarter, Robert J; Buse, John B; Fonseca, Vivian Diabetes care / 2015;38(6):1067-74 1 | Ancillary Publication |
| Radholm 2018 Canagliflozin and Heart Failure in Type 2 Diabetes Mellitus: Results From the CANVAS Program. Radholm, Karin; Figtree, Gemma; Perkovic, Vlado; Solomon, Scott D; Mahaffey, Kenneth W; de Zeeuw, Dick; Fulcher, Greg; Barrett, Terrance D; Shaw, Wayne; Desai, Mehul; Matthews, David R; Neal, Bruce Circulation / 2018;138(5):458-468 | Ancillary Publication |
| Althouse 2013 Favorable effects of insulin sensitizers pertinent to peripheral arterial disease in type 2 diabetes: results from the Bypass Angioplasty Revascularization Investigation 2 Diabetes (BARI 2D) trial. Althouse, Andrew D; Abbott, J Dawn; Sutton-Tyrrell, Kim; Forker, Alan D; Lombardero, Manuel S; Buitron, L Virginia; Pena-Sing, Ivan; Tardif, Jean-Claude; Brooks, Maria Mori; BARI 2D Study Group Diabetes care / 2013;36(10):3269-75 1 | Ancillary Publication |
| Davis 2019 Effects of Severe Hypoglycemia on Cardiovascular Outcomes and Death in the Veterans Affairs Diabetes Trial. Davis, Stephen N; Duckworth, William; Emanuele, Nicholas; Hayward, Rodney A; Wiitala, Wyndy L; Thottapurathu, Lizy; Reda, Domenic J; Reaven, Peter D; Investigators of the Veterans Affairs Diabetes Trial Diabetes care / 2019;42(1):157-163 1 | Ancillary Publication |

| | |
|---|---|
| Azad 2014 Association of PAI-1 and fibrinogen with diabetic retinopathy in the Veterans Affairs Diabetes Trial (VADT). Azad, Nasrin; Agrawal, Lily; Emanuele, Nicholas V; Klein, Ronald; Bahn, Gideon D; McCarren, Madeline; Reaven, Peter; Hayward, Rodney; Duckworth, William; VADT Study Group Diabetes care / 2014;37(2):501-6 1 | Ancillary Publication |
| **Industry trial conducted in one country** | **56** |
| Beishuizen 2004 Two-year statin therapy does not alter the progression of intima-media thickness in patients with type 2 diabetes without manifest cardiovascular disease. Beishuizen, Edith D; van de Ree, Marcel A; Jukema, J Wouter; Tamsma, Jouke T; van der Vijver, J Carel M; Meinders, A Edo; Putter, Hein; Huisman, Menno V Diabetes care / 2004;27(12):2887-92 | Industry trial conducted in one country |
| Umpierrez 2011 Randomized study of basal-bolus insulin therapy in the inpatient management of patients with type 2 diabetes undergoing general surgery (RABBIT 2 surgery). Umpierrez, Guillermo E; Smiley, Dawn; Jacobs, Sol; Peng, Limin; Temponi, Angel; Mulligan, Patrick; Umpierrez, Denise; Newton, Christopher; Olson, Darin; Rizzo, Monica Diabetes care / 2011;34(2):256-61 | Industry trial conducted in one country |
| Chiasson 2001 The synergistic effect of miglitol plus metformin combination therapy in the treatment of type 2 diabetes. Chiasson, J L; Naditch, L; Miglitol Canadian University Investigator Group Diabetes care / 2001;24(6):989-94 | Industry trial conducted in one country |
| Schwartz 2006 Efficacy, tolerability, and safety of a novel once-daily extended-release metformin in patients with type 2 diabetes. Schwartz, Sherwyn; Fonseca, Vivian; Berner, Bret; Cramer, Marilou; Chiang, Yu-Kun; Lewin, Andrew Diabetes care / 2006;29(4):759-64 | Industry trial conducted in one country |
| Wysham 2016 Efficacy and Safety of Multiple Doses of Exenatide Once-Monthly Suspension in Patients With Type 2 Diabetes: A Phase II Randomized Clinical Trial. Wysham, Carol H; MacConell, Leigh; Hardy, Elise Diabetes care / 2016;39(10):1768-76 | Industry trial conducted in one country |
| Zandbergen 2003 Effect of losartan on microalbuminuria in normotensive patients with type 2 diabetes mellitus. A randomized clinical trial. Zandbergen, Adrienne A M; Baggen, Marinus G A; Lamberts, Steven W J; Bootsma, Aart H; de Zeeuw, Dick; Ouwendijk, Rob J Th Annals of internal medicine / 2003;139(2):90-6 | Industry trial conducted in one country |
| DeFronzo 2010 Effects of exenatide plus rosiglitazone on beta-cell function and insulin sensitivity in subjects with type 2 diabetes on metformin. DeFronzo, Ralph A; Triplitt, Curtis; Qu, Yongming; Lewis, Michelle S; Maggs, David; Glass, Leonard C Diabetes care / 2010;33(5):951-7 | Industry trial conducted in one country |
| Cryer 2005 Comparative outcomes study of metformin intervention versus conventional approach the COSMIC Approach Study. Cryer, Dennis R; Nicholas, Savian P; Henry, David H; Mills, Donna J; Stadel, Bruce V Diabetes care / 2005;28(3):539-43 | Industry trial conducted in one country |
| Mathieu 2015 Randomized, Double-Blind, Phase 3 Trial of Triple Therapy With Dapagliflozin Add-on to Saxagliptin Plus Metformin in Type 2 Diabetes. Mathieu, Chantal; Ranetti, Aurelian Emil; Li, Danshi; Ekholm, Ella; Cook, William; Hirshberg, Boaz; Chen, Hungta; Hansen, Lars; Iqbal, Nayyar Diabetes care / 2015;38(11):2009-17 | Industry trial conducted in one country |
| Beishuizen 2005 No effect of statin therapy on silent myocardial ischemia in patients with type 2 diabetes without manifest cardiovascular disease. Beishuizen, Edith D; Jukema, J Wouter; Tamsma, Jouke T; van de Ree, Marcel A; van der Vijver, J Carel M; Putter, Hein; Maan, Arie C; Meinders, A Edo; Huisman, Menno V Diabetes care / 2005;28(7):1675-9 | Industry trial conducted in one country |
| Pradhan 2009 Effects of initiating insulin and metformin on glycemic control and inflammatory biomarkers among patients with type 2 diabetes: the LANCET randomized trial. Pradhan, Aruna D; Everett, Brendan M; Cook, Nancy R; Rifai, Nader; Ridker, Paul M JAMA / 2009;302(11):1186-94 | Industry trial conducted in one country |
| Yang 2008 Biphasic insulin aspart 30 three times daily is more effective than a twice-daily regimen, without increasing hypoglycemia, in Chinese subjects with type 2 diabetes inadequately controlled on oral antidiabetes drugs. Yang, Wenying; Ji, Qiuhe; Zhu, Dalong; Yang, Jinkui; Chen, Lulu; Liu, Zhimin; Yu, Demin; Yan, Li Diabetes care / 2008;31(5):852-6 | Industry trial conducted in one country |
| Tuttle 2005 The effect of ruboxistaurin on nephropathy in type 2 diabetes. Tuttle, Katherine R; Bakris, George L; Toto, Robert D; McGill, Janet B; Hu, Kuolung; Anderson, Pamela W Diabetes care / 2005;28(11):2686-90 | Industry trial conducted in one country |

| | |
|---|---|
| Pergola 2011 Bardoxolone methyl and kidney function in CKD with type 2 diabetes. Pergola, Pablo E; Raskin, Philip; Toto, Robert D; Meyer, Colin J; Huff, J Warren; Grossman, Eric B; Krauth, Melissa; Ruiz, Stacey; Audhya, Paul; Christ-Schmidt, Heidi; Wittes, Janet; Warnock, David G; BEAM Study Investigators The New England journal of medicine / 2011;365(4):327-36 | Industry trial conducted in one country |
| Wanner 2005 Atorvastatin in patients with type 2 diabetes mellitus undergoing hemodialysis. Wanner, Christoph; Krane, Vera; Marz, Winfried; Olschewski, Manfred; Mann, Johannes F E; Ruf, Gunther; Ritz, Eberhard; German Diabetes and Dialysis Study Investigators The New England journal of medicine / 2005;353(3):238-48 | Industry trial conducted in one country |
| Henry 2013 Randomized trial of continuous subcutaneous delivery of exenatide by ITCA 650 versus twice-daily exenatide injections in metformin-treated type 2 diabetes. Henry, Robert R; Rosenstock, Julio; Logan, Douglas K; Alessi, Thomas R; Luskey, Kenneth; Baron, Michelle A Diabetes care / 2013;36(9):2559-65 | Industry trial conducted in one country |
| Mita 2016 Alogliptin, a Dipeptidyl Peptidase 4 Inhibitor, Prevents the Progression of Carotid Atherosclerosis in Patients With Type 2 Diabetes: The Study of Preventive Effects of Alogliptin on Diabetic Atherosclerosis (SPEAD-A). Mita, Tomoya; Katakami, Naoto; Yoshii, Hidenori; Onuma, Tomio; Kaneto, Hideaki; Osonoi, Takeshi; Shiraiwa, Toshihiko; Kosugi, Keisuke; Umayahara, Yutaka; Yamamoto, Tsunehiko; Yokoyama, Hiroki; Kuribayashi, Nobuichi; Jinnouchi, Hideaki; Gosho, Masahiko; Shimomura, Iichiro; Watada, Hirotaka; Collaborators on the Study of Preventive Effects of Alogliptin on Diabetic Atherosclerosis (SPEAD-A) Trial Diabetes care / 2016;39(1):139-48 | Industry trial conducted in one country |
| Fonseca 2000 Effect of metformin and rosiglitazone combination therapy in patients with type 2 diabetes mellitus: a randomized controlled trial. Fonseca, V; Rosenstock, J; Patwardhan, R; Salzman, A JAMA / 2000;283(13):1695-702 | Industry trial conducted in one country |
| Buse 2004 Effects of exenatide (exendin-4) on glycemic control over 30 weeks in sulfonylurea-treated patients with type 2 diabetes. Buse, John B; Henry, Robert R; Han, Jenny; Kim, Dennis D; Fineman, Mark S; Baron, Alain D; Exenatide-113 Clinical Study Group Diabetes care / 2004;27(11):2628-35 | Industry trial conducted in one country |
| Umpierrez 2007 Randomized study of basal-bolus insulin therapy in the inpatient management of patients with type 2 diabetes (RABBIT 2 trial). Umpierrez, Guillermo E; Smiley, Dawn; Zisman, Ariel; Prieto, Luz M; Palacio, Andres; Ceron, Miguel; Puig, Alvaro; Mejia, Roberto Diabetes care / 2007;30(9):2181-6 | Industry trial conducted in one country |
| Kennedy 2006 Impact of active versus usual algorithmic titration of basal insulin and point-of-care versus laboratory measurement of HbA1c on glycemic control in patients with type 2 diabetes: the Glycemic Optimization with Algorithms and Labs at Point of Care (GOAL A Kennedy, Laurence; Herman, William H; Strange, Poul; Harris, Anthony; GOAL AIC Team Diabetes care / 2006;29(1):1-8 Ref ID: 16373887 | Industry trial conducted in one country |
| Ruggenenti 2010 Effects of combined ezetimibe and simvastatin therapy as compared with simvastatin alone in patients with type 2 diabetes: a prospective randomized double-blind clinical trial. Ruggenenti, Piero; Cattaneo, Dario; Rota, Stefano; Iliev, Ilian; Parvanova, Aneliya; Diadei, Olimpia; Ene-Iordache, Bogdan; Ferrari, Silvia; Bossi, Antonio C; Trevisan, Roberto; Belviso, Antonio; Remuzzi, Giuseppe; Ezetimibe and Simvastatin in Dyslipidemia of Diabetes (ESD) Study Group Diabetes care / 2010;33(9):1954-6 | Industry trial conducted in one country |
| Wulffele 2002 Combination of insulin and metformin in the treatment of type 2 diabetes. Wulffele, Michiel G; Kooy, Adriaan; Lehert, Philippe; Bets, Daniel; Ogterop, Jeles C; Borger van der Burg, Bob; Donker, Ab J M; Stehouwer, Coen D A Diabetes care / 2002;25(12):2133-40 | Industry trial conducted in one country |
| StJohnSutton 2002 A comparison of the effects of rosiglitazone and glyburide on cardiovascular function and glycemic control in patients with type 2 diabetes. St John Sutton, Martin; Rendell, Marc; Dandona, Paresh; Dole, Jo F; Murphy, Karen; Patwardhan, Rita; Patel, Jai; Freed, Martin Diabetes care / 2002;25(11):2058-64 | Industry trial conducted in one country |
| Phillips 2001 Once- and twice-daily dosing with rosiglitazone improves glycemic control in patients with type 2 diabetes. Phillips, L S; Grunberger, G; Miller, E; Patwardhan, R; Rappaport, E B; Salzman, A; Rosiglitazone Clinical Trials Study Group Diabetes care / 2001;24(2):308-15 | Industry trial conducted in one country |

| | |
|---|---|
| Mita 2016 Sitagliptin Attenuates the Progression of Carotid Intima-Media Thickening in Insulin-Treated Patients With Type 2 Diabetes: The Sitagliptin Preventive Study of Intima-Media Thickness Evaluation (SPIKE): A Randomized Controlled Trial. Mita, Tomoya; Katakami, Naoto; Shiraiwa, Toshihiko; Yoshii, Hidenori; Onuma, Tomio; Kuribayashi, Nobuichi; Osonoi, Takeshi; Kaneto, Hideaki; Kosugi, Keisuke; Umayahara, Yutaka; Yamamoto, Tsunehiko; Matsumoto, Kazunari; Yokoyama, Hiroki; Tsugawa, Mamiko; Gosho, Masahiko; Shimomura, Iichiro; Watada, Hirotaka; Collaborators on the Sitagliptin Preventive Study of Intima-Media Thickness Evaluation (SPIKE) Trial Diabetes care / 2016;39(3):455-64 | Industry trial conducted in one country |
| Yale 2001 The effect of a thiazolidinedione drug, troglitazone, on glycemia in patients with type 2 diabetes mellitus poorly controlled with sulfonylurea and metformin. A multicenter, randomized, double-blind, placebo-controlled trial. Yale, J F; Valiquett, T R; Ghazzi, M N; Owens-Grillo, J K; Whitcomb, R W; Foyt, H L Annals of internal medicine / 2001;134(9 Pt 1):737-45 | Industry trial conducted in one country |
| Bakris 2004 Metabolic effects of carvedilol vs metoprolol in patients with type 2 diabetes mellitus and hypertension: a randomized controlled trial. Bakris, George L; Fonseca, Vivian; Katholi, Richard E; McGill, Janet B; Messerli, Franz H; Phillips, Robert A; Raskin, Philip; Wright, Jackson T Jr; Oakes, Rosemary; Lukas, Mary Ann; Anderson, Karen M; Bell, David S H; GEMINI Investigators JAMA / 2004;292(18):2227-36 | Industry trial conducted in one country |
| Rosenstock 2015 Greater dose-ranging effects on A1C levels than on glucosuria with LX4211, a dual inhibitor of SGLT1 and SGLT2, in patients with type 2 diabetes on metformin monotherapy. Rosenstock, Julio; Cefalu, William T; Lapuerta, Pablo; Zambrowicz, Brian; Ogbaa, Ike; Banks, Phillip; Sands, Arthur Diabetes care / 2015;38(3):431-8 | Industry trial conducted in one country |
| Kelley 2002 Clinical efficacy of orlistat therapy in overweight and obese patients with insulin-treated type 2 diabetes: A 1-year randomized controlled trial. Kelley, David E; Bray, George A; Pi-Sunyer, F Xavier; Klein, Samuel; Hill, James; Miles, John; Hollander, Priscilla Diabetes care / 2002;25(6):1033-41 | Industry trial conducted in one country |
| Riddle 2007 Pramlintide improved glycemic control and reduced weight in patients with type 2 diabetes using basal insulin. Riddle, Matthew; Frias, Juan; Zhang, Bei; Maier, Holly; Brown, Carl; Lutz, Karen; Kolterman, Orville Diabetes care / 2007;30(11):2794-9 | Industry trial conducted in one country |
| Raskin 2003 Continuous subcutaneous insulin infusion and multiple daily injection therapy are equally effective in type 2 diabetes: a randomized, parallel-group, 24-week study. Raskin, Philip; Bode, Bruce W; Marks, Jennifer B; Hirsch, Irl B; Weinstein, Richard L; McGill, Janet B; Peterson, Gregory E; Mudaliar, Sunder R; Reinhardt, Rickey R Diabetes care / 2003;26(9):2598-603 | Industry trial conducted in one country |
| Ryan 2006 Improving metabolic control leads to better working memory in adults with type 2 diabetes. Ryan, Christopher M; Freed, Martin I; Rood, Julie A; Cobitz, Alexander R; Waterhouse, Brian R; Strachan, Mark W J Diabetes care / 2006;29(2):345-51 | Industry trial conducted in one country |
| Fineman 2003 Effect on glycemic control of exenatide (synthetic exendin-4) additive to existing metformin and/or sulfonylurea treatment in patients with type 2 diabetes. Fineman, Mark S; Bicsak, Thomas A; Shen, Larry Z; Taylor, Kristin; Gaines, Eling; Varns, Amanda; Kim, Dennis; Baron, Alain D Diabetes care / 2003;26(8):2370-7 | Industry trial conducted in one country |
| Raskin 2001 A randomized trial of rosiglitazone therapy in patients with inadequately controlled insulin-treated type 2 diabetes. Raskin, P; Rendell, M; Riddle, M C; Dole, J F; Freed, M I; Rosenstock, J; Rosiglitazone Clinical Trials Study Group Diabetes care / 2001;24(7):1226-32 | Industry trial conducted in one country |
| Mazzone 2006 Effect of pioglitazone compared with glimepiride on carotid intima-media thickness in type 2 diabetes: a randomized trial. Mazzone, Theodore; Meyer, Peter M; Feinstein, Steven B; Davidson, Michael H; Kondos, George T; D'Agostino, Ralph B Sr; Perez, Alfonso; Provost, Jean-Claude; Haffner, Steven M JAMA / 2006;296(21):2572-81 | Industry trial conducted in one country |
| Gallwitz 2011 Exenatide twice daily versus premixed insulin aspart 70/30 in metformin-treated patients with type 2 diabetes: a randomized 26-week study on glycemic control and hypoglycemia. Gallwitz, Baptist; Bohmer, Michael; Segiet, Thomas; Molle, Andrea; Milek, Karsten; Becker, Bernd; Helsberg, Karin; Petto, Helmut; Peters, Natalie; Bachmann, Oliver Diabetes care / 2011;34(3):604-6 | Industry trial conducted in one country |

| | |
|---|---|
| Araki 2018 Effects of Pemafibrate, a Novel Selective PPARalpha Modulator, on Lipid and Glucose Metabolism in Patients With Type 2 Diabetes and Hypertriglyceridemia: A Randomized, Double-Blind, Placebo-Controlled, Phase 3 Trial. Araki, Eiichi; Yamashita, Shizuya; Arai, Hidenori; Yokote, Koutaro; Satoh, Jo; Inoguchi, Toyoshi; Nakamura, Jiro; Maegawa, Hiroshi; Yoshioka, Narihito; Tanizawa, Yukio; Watada, Hirotaka; Suganami, Hideki; Ishibashi, Shun Diabetes care / 2018;41(3):538-546 | Industry trial conducted in one country |
| Baldwin 2012 A randomized trial of two weight-based doses of insulin glargine and glulisine in hospitalized subjects with type 2 diabetes and renal insufficiency. Baldwin, David; Zander, Jennifer; Munoz, Christina; Raghu, Preeya; DeLange-Hudec, Susan; Lee, Hong; Emanuele, Mary Ann; Glossop, Valerie; Smallwood, Kimberly; Molitch, Mark Diabetes care / 2012;35(10):1970-4 | Industry trial conducted in one country |
| Watada 2020 Efficacy and Safety of 1:1 Fixed-Ratio Combination of Insulin Glargine and Lixisenatide Versus Lixisenatide in Japanese Patients With Type 2 Diabetes Inadequately Controlled on Oral Antidiabetic Drugs: The LixiLan JP-O1 Randomized Clinical Trial. Watada, Hirotaka; Takami, Akane; Spranger, Robert; Amano, Atsushi; Hashimoto, Yasuhiro; Niemoeller, Elisabeth Diabetes care / 2020;43(6):1249-1257 | Industry trial conducted in one country |
| Langenfeld 2005 Pioglitazone decreases carotid intima-media thickness independently of glycemic control in patients with type 2 diabetes mellitus: results from a controlled randomized study. Langenfeld, M R; Forst, T; Hohberg, C; Kann, P; Lubben, G; Konrad, T; Fullert, S D; Sachara, C; Pfutzner, A Circulation / 2005;111(19):2525-31 | Industry trial conducted in one country |
| Meier 2015 Contrasting Effects of Lixisenatide and Liraglutide on Postprandial Glycemic Control, Gastric Emptying, and Safety Parameters in Patients With Type 2 Diabetes on Optimized Insulin Glargine With or Without Metformin: A Randomized, Open-Label Trial. Meier, Juris J; Rosenstock, Julio; Hincelin-Mery, Agnes; Roy-Duval, Christine; Delfolie, Astrid; Coester, Hans-Veit; Menge, Bjoern A; Forst, Thomas; Kapitza, Christoph Diabetes care / 2015;38(7):1263-73 | Industry trial conducted in one country |
| Rosenstock 2004 Repaglinide versus nateglinide monotherapy: a randomized, multicenter study. Rosenstock, Julio; Hassman, David R; Madder, Robert D; Brazinsky, Shari A; Farrell, James; Khutoryansky, Naum; Hale, Paula M; Repaglinide Versus Nateglinide Comparison Study Group Diabetes care / 2004;27(6):1265-70 | Industry trial conducted in one country |
| DiabetesAtorvastinLipidIntervention(DALI)StudyGroup 2001 The effect of aggressive versus standard lipid lowering by atorvastatin on diabetic dyslipidemia: the DALI study: a double-blind, randomized, placebo-controlled trial in patients with type 2 diabetes and diabetic dyslipidemia. Diabetes Atorvastin Lipid Intervention (DALI) Study Group Diabetes care / 2001;24(8):1335-41 | Industry trial conducted in one country |
| Beishuizen 2005 The effect of statin therapy on endothelial function in type 2 diabetes without manifest cardiovascular disease. Beishuizen, Edith D; Tamsma, Jouke T; Jukema, J Wouter; van de Ree, Marcel A; van der Vijver, J Carel M; Meinders, A Edo; Huisman, Menno V Diabetes care / 2005;28(7):1668-74 | Industry trial conducted in one country |
| Aronoff 2000 Pioglitazone hydrochloride monotherapy improves glycemic control in the treatment of patients with type 2 diabetes: a 6-month randomized placebo-controlled dose-response study. The Pioglitazone 001 Study Group. Aronoff, S; Rosenblatt, S; Braithwaite, S; Egan, J W; Mathisen, A L; Schneider, R L Diabetes care / 2000;23(11):1605-11 | Industry trial conducted in one country |
| DeFronzo 2005 Efficacy of inhaled insulin in patients with type 2 diabetes not controlled with diet and exercise: a 12-week, randomized, comparative trial. DeFronzo, Ralph A; Bergenstal, Richard M; Cefalu, William T; Pullman, John; Lerman, Sam; Bode, Bruce W; Phillips, Lawrence S; Exubera Phase III Study Group Diabetes care / 2005;28(8):1922-8 | Industry trial conducted in one country |
| Sloan-Lancaster 2013 Double-blind, randomized study evaluating the glycemic and anti-inflammatory effects of subcutaneous LY2189102, a neutralizing IL-1beta antibody, in patients with type 2 diabetes. Sloan-Lancaster, Joanne; Abu-Raddad, Eyas; Polzer, John; Miller, Jeffrey W; Scherer, Joel C; De Gaetano, Andrea; Berg, Jolene K; Landschulz, William H Diabetes care / 2013;36(8):2239-46 | Industry trial conducted in one country |
| Arnolds 2010 Further improvement in postprandial glucose control with addition of exenatide or sitagliptin to combination therapy with insulin glargine and metformin: a proof-of-concept study. Arnolds, Sabine; Dellweg, Sibylle; Clair, Janina; Dain, Marie-Paule; Nauck, Michael A; Rave, Klaus; Kapitza, Christoph Diabetes care / 2010;33(7):1509-15 | Industry trial conducted in one country |

| | |
|---|---|
| Ruggenenti 2004 Preventing microalbuminuria in type 2 diabetes. Ruggenenti, Piero; Fassi, Anna; Ilieva, Anelja Parvanova; Bruno, Simona; Iliev, Ilian Petrov; Brusegan, Varusca; Rubis, Nadia; Gherardi, Giulia; Arnoldi, Federica; Ganeva, Maria; Ene-Iordache, Bogdan; Gaspari, Flavio; Perna, Annalisa; Bossi, Antonio; Trevisan, Roberto; Dodesini, Alessandro R; Remuzzi, Giuseppe; Bergamo Nephrologic Diabetes Complications Trial (BENEDICT) Investigators The New England journal of medicine / 2004;351(19):1941-51 | Industry trial conducted in one country |
| DeFronzo 2005 Effects of exenatide (exendin-4) on glycemic control and weight over 30 weeks in metformin-treated patients with type 2 diabetes. DeFronzo, Ralph A; Ratner, Robert E; Han, Jenny; Kim, Dennis D; Fineman, Mark S; Baron, Alain D Diabetes care / 2005;28(5):1092-100 | Industry trial conducted in one country |
| Rosenstock 2010 The 11-beta-hydroxysteroid dehydrogenase type 1 inhibitor INCB13739 improves hyperglycemia in patients with type 2 diabetes inadequately controlled by metformin monotherapy. Rosenstock, Julio; Banarer, Salomon; Fonseca, Vivian A; Inzucchi, Silvio E; Sun, William; Yao, Wenqing; Hollis, Gregory; Flores, Robert; Levy, Richard; Williams, William V; Seckl, Jonathan R; Huber, Reid; INCB13739-202 Principal Investigators Diabetes care / 2010;33(7):1516-22 | Industry trial conducted in one country |
| Kendall 2005 Effects of exenatide (exendin-4) on glycemic control over 30 weeks in patients with type 2 diabetes treated with metformin and a sulfonylurea. Kendall, David M; Riddle, Matthew C; Rosenstock, Julio; Zhuang, Dongliang; Kim, Dennis D; Fineman, Mark S; Baron, Alain D Diabetes care / 2005;28(5):1083-91 | Industry trial conducted in one country |
| Gram 2011 Pharmacological treatment of the pathogenetic defects in type 2 diabetes: the randomized multicenter South Danish Diabetes Study. Gram, Jeppe; Henriksen, Jan Erik; Grodum, Ellen; Juhl, Henning; Hansen, Tony Bill; Christiansen, Christian; Yderstraede, Knud; Gjessing, Hans; Hansen, Henrik M; Vestergaard, Vibe; Hangaard, Jorgen; Beck-Nielsen, Henning Diabetes care / 2011;34(1):27-33 1 | Industry trial conducted in one country |
| Wysham 2017 Effect of Insulin Degludec vs Insulin Glargine U100 on Hypoglycemia in Patients With Type 2 Diabetes: The SWITCH 2 Randomized Clinical Trial. Wysham, Carol; Bhargava, Anuj; Chaykin, Louis; de la Rosa, Raymond; Handelsman, Yehuda; Troelsen, Lone N; Kvist, Kajsa; Norwood, Paul JAMA / 2017;318(1):45-56 | Industry trial conducted in one country |
| **Industry trial in specialty journal (Circulation, Diabetes Care)** | **159** |
| Hollander 2010 Effect of rimonabant on glycemic control in insulin-treated type 2 diabetes: the ARPEGGIO trial. Hollander, Priscilla A; Amod, Aslam; Litwak, Leon E; Chaudhari, Umesh; ARPEGGIO Study Group Diabetes care / 2010;33(3):605-7 | Industry trial in a specialty journal |
| Riddle 2013 Adding once-daily lixisenatide for type 2 diabetes inadequately controlled by established basal insulin: a 24-week, randomized, placebo-controlled comparison (GetGoal-L). Riddle, Matthew C; Aronson, Ronnie; Home, Philip; Marre, Michel; Niemoeller, Elisabeth; Miossec, Patrick; Ping, Lin; Ye, Jenny; Rosenstock, Julio Diabetes care / 2013;36(9):2489-96 | Industry trial in a specialty journal |
| Ahren 2014 HARMONY 3: 104-week randomized, double-blind, placebo- and active-controlled trial assessing the efficacy and safety of albiglutide compared with placebo, sitagliptin, and glimepiride in patients with type 2 diabetes taking metformin. Ahren, Bo; Johnson, Susan L; Stewart, Murray; Cirkel, Deborah T; Yang, Fred; Perry, Caroline; Feinglos, Mark N; HARMONY 3 Study Group Diabetes care / 2014;37(8):2141-8 | Industry trial in a specialty journal |
| Madsbad 2004 Improved glycemic control with no weight increase in patients with type 2 diabetes after once-daily treatment with the long-acting glucagon-like peptide 1 analog liraglutide (NN2211): a 12-week, double-blind, randomized, controlled trial. Madsbad, Sten; Schmitz, Ole; Ranstam, Jonas; Jakobsen, Grethe; Matthews, David R; NN2211-1310 International Study Group Diabetes care / 2004;27(6):1335-42 | Industry trial in a specialty journal |
| Kaku 2013 Randomized, double-blind, dose-ranging study of TAK-875, a novel GPR40 agonist, in Japanese patients with inadequately controlled type 2 diabetes. Kaku, Kohei; Araki, Takahiro; Yoshinaka, Ryoji Diabetes care / 2013;36(2):245-50 | Industry trial in a specialty journal |
| Lane 2020 A Randomized Trial Evaluating the Efficacy and Safety of Fast-Acting Insulin Aspart Compared With Insulin Aspart, Both in Combination With Insulin Degludec With or Without Metformin, in Adults With Type 2 Diabetes (ONSET 9). Lane, Wendy S; Favaro, Elena; Rathor, Naveen; Jang, Hak C; Kjaersgaard, Maiken I S; Oviedo, Alejandra; Rose, Ludger; Senior, Peter; Sesti, Giorgio; Soto Gonzalez, Alfonso; Franek, Edward Diabetes care / 2020;43(8):1710-1716 | Industry trial in a specialty journal |

| | |
|---|---|
| Yki-Jarvinen 2000 Less nocturnal hypoglycemia and better post-dinner glucose control with bedtime insulin glargine compared with bedtime NPH insulin during insulin combination therapy in type 2 diabetes. HOE 901/3002 Study Group. Yki-Jarvinen, H; Dressler, A; Ziemen, M; HOE 901/300s Study Group Diabetes care / 2000;23(8):1130-6 | Industry trial in a specialty journal |
| Tan 2005 Comparison of pioglitazone and gliclazide in sustaining glycemic control over 2 years in patients with type 2 diabetes. Tan, Meng H; Baksi, Arun; Krahulec, Boris; Kubalski, Piotr; Stankiewicz, Andrzej; Urquhart, Richard; Edwards, Gareth; Johns, Don; GLAL Study Group Diabetes care / 2005;28(3):544-50 | Industry trial in a specialty journal |
| Fonseca 2003 Addition of nateglinide to rosiglitazone monotherapy suppresses mealtime hyperglycemia and improves overall glycemic control. Fonseca, Vivian; Grunberger, George; Gupta, Shamita; Shen, Sharon; Foley, James E Diabetes care / 2003;26(6):1685-90 | Industry trial in a specialty journal |
| Rosenstock 2008 Two-year pulmonary safety and efficacy of inhaled human insulin (Exubera) in adult patients with type 2 diabetes. Rosenstock, Julio; Cefalu, William T; Hollander, Priscilla A; Belanger, Andre; Eliaschewitz, Freddy G; Gross, Jorge L; Klioze, Solomon S; St Aubin, Lisa B; Foyt, Howard; Ogawa, Masayo; Duggan, William T Diabetes care / 2008;31(9):1723-8 | Industry trial in a specialty journal |
| Ahmann 2018 Efficacy and Safety of Once-Weekly Semaglutide Versus Exenatide ER in Subjects With Type 2 Diabetes (SUSTAIN 3): A 56-Week, Open-Label, Randomized Clinical Trial. Ahmann, Andrew J; Capehorn, Matthew; Charpentier, Guillaume; Dotta, Francesco; Henkel, Elena; Lingvay, Ildiko; Holst, Anders G; Annett, Miriam P; Aroda, Vanita R Diabetes care / 2018;41(2):258-266 | Industry trial in a specialty journal |
| Lewin 2015 Initial combination of empagliflozin and linagliptin in subjects with type 2 diabetes. Lewin, Andrew; DeFronzo, Ralph A; Patel, Sanjay; Liu, Dacheng; Kaste, Renee; Woerle, Hans J; Broedl, Uli C Diabetes care / 2015;38(3):394-402 | Industry trial in a specialty journal |
| Davies 2005 Improvement of glycemic control in subjects with poorly controlled type 2 diabetes: comparison of two treatment algorithms using insulin glargine. Davies, Melanie; Storms, Fred; Shutler, Simon; Bianchi-Biscay, Monique; Gomis, Ramon; ATLANTUS Study Group Diabetes care / 2005;28(6):1282-8 | Industry trial in a specialty journal |
| Rosenstock 2007 A randomized, double-blind, placebo-controlled, multicenter study to assess the efficacy and safety of topiramate controlled release in the treatment of obese type 2 diabetic patients. Rosenstock, Julio; Hollander, Priscilla; Gadde, Kishore M; Sun, Xiang; Strauss, Richard; Leung, Albert; OBD-202 Study Group Diabetes care / 2007;30(6):1480-6 | Industry trial in a specialty journal |
| Mari 2005 Beta-cell function in mild type 2 diabetic patients: effects of 6-month glucose lowering with nateglinide. Mari, Andrea; Gastaldelli, Amalia; Foley, James E; Pratley, Richard E; Ferrannini, Ele Diabetes care / 2005;28(5):1132-8 | Industry trial in a specialty journal |
| Yki-Jarvinen 2014 New insulin glargine 300 units/mL versus glargine 100 units/mL in people with type 2 diabetes using oral agents and basal insulin: glucose control and hypoglycemia in a 6-month randomized controlled trial (EDITION 2). Yki-Jarvinen, Hannele; Bergenstal, Richard; Ziemen, Monika; Wardecki, Marek; Muehlen-Bartmer, Isabel; Boelle, Emmanuelle; Riddle, Matthew C; EDITION 2 Study Investigators Diabetes care / 2014;37(12):3235-43 | Industry trial in a specialty journal |
| Moulin 2006 Efficacy of benfluorex in combination with sulfonylurea in type 2 diabetic patients: an 18-week, randomized, double-blind study. Moulin, Philippe; Andre, Marie; Alawi, Hasan; dos Santos, Lelita C; Khalid, Abdul K; Koev, Dragomir; Moore, Ray; Serban, Viorel; Picandet, Brigitte; Francillard, Marie Diabetes care / 2006;29(3):515-20 | Industry trial in a specialty journal |
| Hanefeld 2004 One-year glycemic control with a sulfonylurea plus pioglitazone versus a sulfonylurea plus metformin in patients with type 2 diabetes. Hanefeld, Markolf; Brunetti, Paolo; Schernthaner, Guntram H; Matthews, David R; Charbonnel, Bernard H; QUARTET Study Group Diabetes care / 2004;27(1):141-7 | Industry trial in a specialty journal |
| Meneghini 2013 The efficacy and safety of insulin degludec given in variable once-daily dosing intervals compared with insulin glargine and insulin degludec dosed at the same time daily: a 26-week, randomized, open-label, parallel-group, treat-to-target trial in individ Meneghini, Luigi; Atkin, Stephen L; Gough, Stephen C L; Raz, Itamar; Blonde, Lawrence; Shestakova, Marina; Bain, Stephen; Johansen, Thue; Begtrup, Kamilla; Birkeland, Kare I; NN1250-3668 (BEGIN FLEX) Trial Investigators Diabetes care / 2013;36(4):858-64 | Industry trial in a specialty journal |

| | |
|---|---|
| Raz 2009 Effects of prandial versus fasting glycemia on cardiovascular outcomes in type 2 diabetes: the HEART2D trial. Raz, Itamar; Wilson, Peter W F; Strojek, Krzysztof; Kowalska, Irina; Bozikov, Velimir; Gitt, Anselm K; Jermendy, Gyorgy; Campaigne, Barbara N; Kerr, Lisa; Milicevic, Zvonko; Jacober, Scott J Diabetes care / 2009;32(3):381-6 | Industry trial in a specialty journal |
| Rosenstock 2016 Benefits of LixiLan, a Titratable Fixed-Ratio Combination of Insulin Glargine Plus Lixisenatide, Versus Insulin Glargine and Lixisenatide Monocomponents in Type 2 Diabetes Inadequately Controlled on Oral Agents: The LixiLan-O Randomized Trial. Rosenstock, Julio; Aronson, Ronnie; Grunberger, George; Hanefeld, Markolf; Piatti, PierMarco; Serusclat, Pierre; Cheng, Xi; Zhou, Tianyue; Niemoeller, Elisabeth; Souhami, Elisabeth; Davies, Melanie; LixiLan-O Trial Investigators Diabetes care / 2016;39(11):2026-2035 | Industry trial in a specialty journal |
| Wysham 2014 Efficacy and safety of dulaglutide added onto pioglitazone and metformin versus exenatide in type 2 diabetes in a randomized controlled trial (AWARD-1). Wysham, Carol; Blevins, Thomas; Arakaki, Richard; Colon, Gildred; Garcia, Pedro; Atisso, Charles; Kuhstoss, Debra; Lakshmanan, Mark Diabetes care / 2014;37(8):2159-67 | Industry trial in a specialty journal |
| Schernthaner 2013 Canagliflozin compared with sitagliptin for patients with type 2 diabetes who do not have adequate glycemic control with metformin plus sulfonylurea: a 52-week randomized trial. Schernthaner, Guntram; Gross, Jorge L; Rosenstock, Julio; Guarisco, Michael; Fu, Min; Yee, Jacqueline; Kawaguchi, Masato; Canovatchel, William; Meininger, Gary Diabetes care / 2013;36(9):2508-15 | Industry trial in a specialty journal |
| Rosenstock 2013 The fate of taspoglutide, a weekly GLP-1 receptor agonist, versus twice-daily exenatide for type 2 diabetes: the T-emerge 2 trial. Rosenstock, Julio; Balas, Bogdan; Charbonnel, Bernard; Bolli, Geremia B; Boldrin, Mark; Ratner, Robert; Balena, Raffaella; T-emerge 2 Study Group Diabetes care / 2013;36(3):498-504 | Industry trial in a specialty journal |
| Pettus 2020 Efficacy and Safety of the Glucagon Receptor Antagonist RVT-1502 in Type 2 Diabetes Uncontrolled on Metformin Monotherapy: A 12-Week Dose-Ranging Study. Pettus, Jeremy H; D'Alessio, David; Frias, Juan P; Vajda, Eric G; Pipkin, James D; Rosenstock, Julio; Williamson, Gretchen; Zangmeister, Miriam A; Zhi, Lin; Marschke, Keith B Diabetes care / 2020;43(1):161-168 | Industry trial in a specialty journal |
| Charbonnel 2006 Efficacy and safety of the dipeptidyl peptidase-4 inhibitor sitagliptin added to ongoing metformin therapy in patients with type 2 diabetes inadequately controlled with metformin alone. Charbonnel, Bernard; Karasik, Avraham; Liu, Ji; Wu, Mei; Meininger, Gary; Sitagliptin Study 020 Group Diabetes care / 2006;29(12):2638-43 | Industry trial in a specialty journal |
| Bowering 2017 Faster Aspart Versus Insulin Aspart as Part of a Basal-Bolus Regimen in Inadequately Controlled Type 2 Diabetes: The onset 2 Trial. Bowering, Keith; Case, Christopher; Harvey, John; Reeves, Michael; Sampson, Michael; Strzinek, Robert; Bretler, Ditte-Marie; Bang, Rikke Beck; Bode, Bruce W Diabetes care / 2017;40(7):951-957 | Industry trial in a specialty journal |
| Riddle 2013 Adding once-daily lixisenatide for type 2 diabetes inadequately controlled with newly initiated and continuously titrated basal insulin glargine: a 24-week, randomized, placebo-controlled study (GetGoal-Duo 1). Riddle, Matthew C; Forst, Thomas; Aronson, Ronnie; Sauque-Reyna, Leobardo; Souhami, Elisabeth; Silvestre, Louise; Ping, Lin; Rosenstock, Julio Diabetes care / 2013;36(9):2497-503 | Industry trial in a specialty journal |
| Hollander 2001 Importance of early insulin secretion: comparison of nateglinide and glyburide in previously diet-treated patients with type 2 diabetes. Hollander, P A; Schwartz, S L; Gatlin, M R; Haas, S J; Zheng, H; Foley, J E; Dunning, B E Diabetes care / 2001;24(6):983-8 | Industry trial in a specialty journal |
| Gough 2013 Low-volume insulin degludec 200 units/ml once daily improves glycemic control similarly to insulin glargine with a low risk of hypoglycemia in insulin-naive patients with type 2 diabetes: a 26-week, randomized, controlled, multinational, treat-to-target t Gough, Stephen C L; Bhargava, Anuj; Jain, Rajeev; Mersebach, Henriette; Rasmussen, Soren; Bergenstal, Richard M Diabetes care / 2013;36(9):2536-42 | Industry trial in a specialty journal |
| Cefalu 2015 Dapagliflozin's Effects on Glycemia and Cardiovascular Risk Factors in High-Risk Patients With Type 2 Diabetes: A 24-Week, Multicenter, Randomized, Double-Blind, Placebo-Controlled Study With a 28-Week Extension. Cefalu, William T; Leiter, Lawrence A; de Bruin, Tjerk W A; Gause-Nilsson, Ingrid; Sugg, Jennifer; Parikh, Shamik J Diabetes care / 2015;38(7):1218-27 | Industry trial in a specialty journal |

| | |
|---|---|
| Fonseca 2012 Efficacy and safety of the once-daily GLP-1 receptor agonist lixisenatide in monotherapy: a randomized, double-blind, placebo-controlled trial in patients with type 2 diabetes (GetGoal-Mono). Fonseca, Vivian A; Alvarado-Ruiz, Ricardo; Raccah, Denis; Boka, Gabor; Miossec, Patrick; Gerich, John E; EFC6018 GetGoal-Mono Study Investigators Diabetes care / 2012;35(6):1225-31 | Industry trial in a specialty journal |
| Swinnen 2010 A 24-week, randomized, treat-to-target trial comparing initiation of insulin glargine once-daily with insulin detemir twice-daily in patients with type 2 diabetes inadequately controlled on oral glucose-lowering drugs. Swinnen, Sanne G; Dain, Marie-Paule; Aronson, Ronnie; Davies, Melanie; Gerstein, Hertzel C; Pfeiffer, Andreas F; Snoek, Frank J; Devries, J Hans; Hoekstra, Joost B; Holleman, Frits Diabetes care / 2010;33(6):1176-8 | Industry trial in a specialty journal |
| Rosenstock 2007 Comparison of vildagliptin and rosiglitazone monotherapy in patients with type 2 diabetes: a 24-week, double-blind, randomized trial. Rosenstock, Julio; Baron, Michelle A; Dejager, Sylvie; Mills, David; Schweizer, Anja Diabetes care / 2007;30(2):217-23 | Industry trial in a specialty journal |
| Hanefeld 2000 Rapid and short-acting mealtime insulin secretion with nateglinide controls both prandial and mean glycemia. Hanefeld, M; Bouter, K P; Dickinson, S; Guitard, C Diabetes care / 2000;23(2):202-7 | Industry trial in a specialty journal |
| Hermansen 2004 Intensive therapy with inhaled insulin via the AERx insulin diabetes management system: a 12-week proof-of-concept trial in patients with type 2 diabetes. Hermansen, Kjeld; Ronnemaa, Tapani; Petersen, Astrid Heide; Bellaire, Shannon; Adamson, Ulf Diabetes care / 2004;27(1):162-7 | Industry trial in a specialty journal |
| Rosenstock 2014 Improved glucose control with weight loss, lower insulin doses, and no increased hypoglycemia with empagliflozin added to titrated multiple daily injections of insulin in obese inadequately controlled type 2 diabetes. Rosenstock, Julio; Jelaska, Ante; Frappin, Guillaume; Salsali, Afshin; Kim, Gabriel; Woerle, Hans J; Broedl, Uli C; EMPA-REG MDI Trial Investigators Diabetes care / 2014;37(7):1815-23 | Industry trial in a specialty journal |
| Best 2011 Weight-related quality of life, health utility, psychological well-being, and satisfaction with exenatide once weekly compared with sitagliptin or pioglitazone after 26 weeks of treatment. Best, Jennie H; Rubin, Richard R; Peyrot, Mark; Li, Yan; Yan, Ping; Malloy, Jaret; Garrison, Louis P Diabetes care / 2011;34(2):314-9 1 | Industry trial in a specialty journal |
| Billings 2018 Efficacy and Safety of IDegLira Versus Basal-Bolus Insulin Therapy in Patients With Type 2 Diabetes Uncontrolled on Metformin and Basal Insulin: The DUAL VII Randomized Clinical Trial. Billings, Liana K; Doshi, Ankur; Gouet, Didier; Oviedo, Alejandra; Rodbard, Helena W; Tentolouris, Nikolaos; Gron, Randi; Halladin, Natalie; Jodar, Esteban Diabetes care / 2018;41(5):1009-1016 | Industry trial in a specialty journal |
| Matthaei 2015 Randomized, Double-Blind Trial of Triple Therapy With Saxagliptin Add-on to Dapagliflozin Plus Metformin in Patients With Type 2 Diabetes. Matthaei, Stephan; Catrinoiu, Doina; Celinski, Aleksander; Ekholm, Ella; Cook, William; Hirshberg, Boaz; Chen, Hungta; Iqbal, Nayyar; Hansen, Lars Diabetes care / 2015;38(11):2018-24 | Industry trial in a specialty journal |
| Hermansen 2006 A 26-week, randomized, parallel, treat-to-target trial comparing insulin detemir with NPH insulin as add-on therapy to oral glucose-lowering drugs in insulin-naive people with type 2 diabetes. Hermansen, Kjeld; Davies, Melanie; Derezinski, Taudeusz; Martinez Ravn, Gabrielle; Clauson, Per; Home, Philip Diabetes care / 2006;29(6):1269-74 | Industry trial in a specialty journal |
| Buse 2016 Randomized Clinical Trial Comparing Basal Insulin Peglispro and Insulin Glargine in Patients With Type 2 Diabetes Previously Treated With Basal Insulin: IMAGINE 5. Buse, John B; Rodbard, Helena W; Trescoli Serrano, Carlos; Luo, Junxiang; Ivanyi, Tibor; Bue-Valleskey, Juliana; Hartman, Mark L; Carey, Michelle A; Chang, Annette M; IMAGINE 5 Investigators Diabetes care / 2016;39(1):92-100 | Industry trial in a specialty journal |
| Davies 2016 Efficacy and Safety of Liraglutide Versus Placebo as Add-on to Glucose-Lowering Therapy in Patients With Type 2 Diabetes and Moderate Renal Impairment (LIRA-RENAL): A Randomized Clinical Trial. Davies, Melanie J; Bain, Stephen C; Atkin, Stephen L; Rossing, Peter; Scott, David; Shamkhalova, Minara S; Bosch-Traberg, Heidrun; Syren, Annika; Umpierrez, Guillermo E Diabetes care / 2016;39(2):222-30 | Industry trial in a specialty journal |
| Davies 2013 Once-weekly exenatide versus once- or twice-daily insulin detemir: randomized, open-label, clinical trial of efficacy and safety in patients with type 2 diabetes treated with metformin alone or in combination with sulfonylureas. Davies, Melanie; Heller, Simon; Sreenan, | Industry trial in a specialty journal |

| | |
|---|---|
| Seamus; Sapin, Helene; Adetunji, Omolara; Tahbaz, Arash; Vora, Jiten Diabetes care / 2013;36(5):1368-76 | |
| Meneilly 2017 Lixisenatide Therapy in Older Patients With Type 2 Diabetes Inadequately Controlled on Their Current Antidiabetic Treatment: The GetGoal-O Randomized Trial. Meneilly, Graydon S; Roy-Duval, Christine; Alawi, Hasan; Dailey, George; Bellido, Diego; Trescoli, Carlos; Manrique Hurtado, Helard; Guo, Hailing; Pilorget, Valerie; Perfetti, Riccardo; Simpson, Hamish; GetGoal-O Trial Investigators Diabetes care / 2017;40(4):485-493 | Industry trial in a specialty journal |
| Raskin 2005 Initiating insulin therapy in type 2 Diabetes: a comparison of biphasic and basal insulin analogs. Raskin, Philip; Allen, Elsie; Hollander, Priscilla; Lewin, Andrew; Gabbay, Robert A; Hu, Peter; Bode, Bruce; Garber, Alan; INITIATE Study Group Diabetes care / 2005;28(2):260-5 | Industry trial in a specialty journal |
| Buse 2014 Contribution of liraglutide in the fixed-ratio combination of insulin degludec and liraglutide (IDegLira). Buse, John B; Vilsboll, Tina; Thurman, Jerry; Blevins, Thomas C; Langbakke, Irene H; Bottcher, Susanne G; Rodbard, Helena W; NN9068-3912 (DUAL-II) Trial Investigators Diabetes care / 2014;37(11):2926-33 | Industry trial in a specialty journal |
| Rosenstock 2013 Efficacy and safety of lixisenatide once daily versus exenatide twice daily in type 2 diabetes inadequately controlled on metformin: a 24-week, randomized, open-label, active-controlled study (GetGoal-X). Rosenstock, Julio; Raccah, Denis; Koranyi, Laszlo; Maffei, Laura; Boka, Gabor; Miossec, Patrick; Gerich, John E Diabetes care / 2013;36(10):2945-51 | Industry trial in a specialty journal |
| Vellanki 2015 Randomized controlled trial of insulin supplementation for correction of bedtime hyperglycemia in hospitalized patients with type 2 diabetes. Vellanki, Priyathama; Bean, Rachel; Oyedokun, Festus A; Pasquel, Francisco J; Smiley, Dawn; Farrokhi, Farnoosh; Newton, Christopher; Peng, Limin; Umpierrez, Guillermo E Diabetes care / 2015;38(4):568-74 2 | Industry trial in a specialty journal |
| Raz 2012 Efficacy and safety of taspoglutide monotherapy in drug-naive type 2 diabetic patients after 24 weeks of treatment: results of a randomized, double-blind, placebo-controlled phase 3 study (T-emerge 1). Raz, Itamar; Fonseca, Vivian; Kipnes, Mark; Durrwell, Laurence; Hoekstra, John; Boldrin, Mark; Balena, Raffaella Diabetes care / 2012;35(3):485-7 | Industry trial in a specialty journal |
| Heise 2011 A new-generation ultra-long-acting basal insulin with a bolus boost compared with insulin glargine in insulin-naive people with type 2 diabetes: a randomized, controlled trial. Heise, Tim; Tack, Cees J; Cuddihy, Robert; Davidson, Jaime; Gouet, Didier; Liebl, Andreas; Romero, Enrique; Mersebach, Henriette; Dykiel, Patrik; Jorde, Rolf Diabetes care / 2011;34(3):669-74 | Industry trial in a specialty journal |
| Garvey 2020 Efficacy and Safety of Liraglutide 3.0 mg in Individuals With Overweight or Obesity and Type 2 Diabetes Treated With Basal Insulin: The SCALE Insulin Randomized Controlled Trial. Garvey, W Timothy; Birkenfeld, Andreas L; Dicker, Dror; Mingrone, Geltrude; Pedersen, Sue D; Satylganova, Altynai; Skovgaard, Dorthe; Sugimoto, Danny; Jensen, Camilla; Mosenzon, Ofri Diabetes care / 2020;43(5):1085-1093 | Industry trial in a specialty journal |
| Diamant 2014 Glucagon-like peptide 1 receptor agonist or bolus insulin with optimized basal insulin in type 2 diabetes. Diamant, Michaela; Nauck, Michael A; Shaginian, Rimma; Malone, James K; Cleall, Simon; Reaney, Matthew; de Vries, Danielle; Hoogwerf, Byron J; MacConell, Leigh; Wolffenbuttel, Bruce H R; 4B Study Group Diabetes care / 2014;37(10):2763-73 | Industry trial in a specialty journal |
| Raskin 2003 Efficacy and safety of combination therapy: repaglinide plus metformin versus nateglinide plus metformin. Raskin, Philip; Klaff, Leslie; McGill, Janet; South, Stephen A; Hollander, Priscilla; Khutoryansky, Naum; Hale, Paula M; Repaglinide vs. Nateglinide Metformin Combination Study Group Diabetes care / 2003;26(7):2063-8 | Industry trial in a specialty journal |
| Bergenstal 2012 A randomized, controlled study of once-daily LY2605541, a novel long-acting basal insulin, versus insulin glargine in basal insulin-treated patients with type 2 diabetes. Bergenstal, Richard M; Rosenstock, Julio; Arakaki, Richard F; Prince, Melvin J; Qu, Yongming; Sinha, Vikram P; Howey, Daniel C; Jacober, Scott J Diabetes care / 2012;35(11):2140-7 | Industry trial in a specialty journal |
| Rosenstock 2018 Efficacy and Safety of ITCA 650, a Novel Drug-Device GLP-1 Receptor Agonist, in Type 2 Diabetes Uncontrolled With Oral Antidiabetes Drugs: The FREEDOM-1 Trial. Rosenstock, Julio; Buse, John B; Azeem, Rehan; Prabhakar, Prakash; Kjems, Lise; Huang, Holly; Baron, Michelle A Diabetes care / 2018;41(2):333-340 | Industry trial in a specialty journal |

| | |
|---|---|
| Aroda 2016 Efficacy and Safety of LixiLan, a Titratable Fixed-Ratio Combination of Insulin Glargine Plus Lixisenatide in Type 2 Diabetes Inadequately Controlled on Basal Insulin and Metformin: The LixiLan-L Randomized Trial. Aroda, Vanita R; Rosenstock, Julio; Wysham, Carol; Unger, Jeffrey; Bellido, Diego; Gonzalez-Galvez, Guillermo; Takami, Akane; Guo, Hailing; Niemoeller, Elisabeth; Souhami, Elisabeth; Bergenstal, Richard M; LixiLan-L Trial Investigators Diabetes care / 2016;39(11):1972-1980 | Industry trial in a specialty journal |
| DeFronzo 2009 The efficacy and safety of saxagliptin when added to metformin therapy in patients with inadequately controlled type 2 diabetes with metformin alone. DeFronzo, Ralph A; Hissa, Miguel N; Garber, Alan J; Luiz Gross, Jorge; Yuyan Duan, Raina; Ravichandran, Shoba; Chen, Roland S; Saxagliptin 014 Study Group Diabetes care / 2009;32(9):1649-55 | Industry trial in a specialty journal |
| Softeland 2017 Empagliflozin as Add-on Therapy in Patients With Type 2 Diabetes Inadequately Controlled With Linagliptin and Metformin: A 24-Week Randomized, Double-Blind, Parallel-Group Trial. Softeland, Eirik; Meier, Juris J; Vangen, Bente; Toorawa, Robert; Maldonado-Lutomirsky, Mario; Broedl, Uli C Diabetes care / 2017;40(2):201-209 | Industry trial in a specialty journal |
| Ziegler 2009 Treatment of symptomatic polyneuropathy with actovegin in type 2 diabetic patients. Ziegler, Dan; Movsesyan, Lusine; Mankovsky, Boris; Gurieva, Irina; Abylaiuly, Zhangentkhan; Strokov, Igor Diabetes care / 2009;32(8):1479-84 | Industry trial in a specialty journal |
| Barnett 2006 An open, randomized, parallel-group study to compare the efficacy and safety profile of inhaled human insulin (Exubera) with metformin as adjunctive therapy in patients with type 2 diabetes poorly controlled on a sulfonylurea. Barnett, Anthony H; Dreyer, Manfred; Lange, Peter; Serdarevic-Pehar, Marjana Diabetes care / 2006;29(6):1282-7 | Industry trial in a specialty journal |
| Riddle 2009 Randomized comparison of pramlintide or mealtime insulin added to basal insulin treatment for patients with type 2 diabetes. Riddle, Matthew; Pencek, Richard; Charenkavanich, Supoat; Lutz, Karen; Wilhelm, Ken; Porter, Lisa Diabetes care / 2009;32(9):1577-82 | Industry trial in a specialty journal |
| Fouqueray 2013 The efficacy and safety of imeglimin as add-on therapy in patients with type 2 diabetes inadequately controlled with metformin monotherapy. Fouqueray, Pascale; Pirags, Valdis; Inzucchi, Silvio E; Bailey, Clifford J; Schernthaner, Guntram; Diamant, Michaela; Lebovitz, Harold E Diabetes care / 2013;36(3):565-8 | Industry trial in a specialty journal |
| Russell-Jones 2012 Efficacy and safety of exenatide once weekly versus metformin, pioglitazone, and sitagliptin used as monotherapy in drug-naive patients with type 2 diabetes (DURATION-4): a 26-week double-blind study. Russell-Jones, David; Cuddihy, Robert M; Hanefeld, Markolf; Kumar, Ajay; Gonzalez, Jose G; Chan, Melanie; Wolka, Anne M; Boardman, Marilyn K; DURATION-4 Study Group Diabetes care / 2012;35(2):252-8 | Industry trial in a specialty journal |
| Saad 2004 Ragaglitazar improves glycemic control and lipid profile in type 2 diabetic subjects: a 12-week, double-blind, placebo-controlled dose-ranging study with an open pioglitazone arm. Saad, Mohammed F; Greco, Susan; Osei, Kwame; Lewin, Andrew J; Edwards, Christopher; Nunez, Margarita; Reinhardt, Rickey R; Ragaglitazar Dose-Ranging Study Group Diabetes care / 2004;27(6):1324-9 | Industry trial in a specialty journal |
| Raskin 2000 Repaglinide/troglitazone combination therapy: improved glycemic control in type 2 diabetes. Raskin, P; Jovanovic, L; Berger, S; Schwartz, S; Woo, V; Ratner, R Diabetes care / 2000;23(7):979-83 | Industry trial in a specialty journal |
| Kazda 2016 Evaluation of Efficacy and Safety of the Glucagon Receptor Antagonist LY2409021 in Patients With Type 2 Diabetes: 12- and 24-Week Phase 2 Studies. Kazda, Christof M; Ding, Ying; Kelly, Ronan P; Garhyan, Parag; Shi, Chunxue; Lim, Chay Ngee; Fu, Haoda; Watson, David E; Lewin, Andrew J; Landschulz, William H; Deeg, Mark A; Moller, David E; Hardy, Thomas A Diabetes care / 2016;39(7):1241-9 | Industry trial in a specialty journal |
| Sheu 2015 Safety and Efficacy of Omarigliptin (MK-3102), a Novel Once-Weekly DPP-4 Inhibitor for the Treatment of Patients With Type 2 Diabetes. Sheu, Wayne H-H; Gantz, Ira; Chen, Menghui; Suryawanshi, Shailaja; Mirza, Arpana; Goldstein, Barry J; Kaufman, Keith D; Engel, Samuel S Diabetes care / 2015;38(11):2106-14 | Industry trial in a specialty journal |
| Eckel 2015 Effect of Ranolazine Monotherapy on Glycemic Control in Subjects With Type 2 Diabetes. Eckel, Robert H; Henry, Robert R; Yue, Patrick; Dhalla, Arvinder; Wong, Pamela; Jochelson, Philip; Belardinelli, Luiz; Skyler, Jay S Diabetes care / 2015;38(7):1189-96 | Industry trial in a specialty journal |

| | |
|---|---|
| Nauck 2014 Efficacy and safety of dulaglutide versus sitagliptin after 52 weeks in type 2 diabetes in a randomized controlled trial (AWARD-5). Nauck, Michael; Weinstock, Ruth S; Umpierrez, Guillermo E; Guerci, Bruno; Skrivanek, Zachary; Milicevic, Zvonko Diabetes care / 2014;37(8):2149-58 | Industry trial in a specialty journal |
| Rosenstock 2012 Dose-ranging effects of canagliflozin, a sodium-glucose cotransporter 2 inhibitor, as add-on to metformin in subjects with type 2 diabetes. Rosenstock, Julio; Aggarwal, Naresh; Polidori, David; Zhao, Yue; Arbit, Deborah; Usiskin, Keith; Capuano, George; Canovatchel, William; Canagliflozin DIA 2001 Study Group Diabetes care / 2012;35(6):1232-8 | Industry trial in a specialty journal |
| Bretzel 2004 A direct efficacy and safety comparison of insulin aspart, human soluble insulin, and human premix insulin (70/30) in patients with type 2 diabetes. Bretzel, Reinhard G; Arnolds, Sabine; Medding, Jurgen; Linn, Thomas Diabetes care / 2004;27(5):1023-7 | Industry trial in a specialty journal |
| Haring 2013 Empagliflozin as add-on to metformin plus sulfonylurea in patients with type 2 diabetes: a 24-week, randomized, double-blind, placebo-controlled trial. Haring, Hans-Ulrich; Merker, Ludwig; Seewaldt-Becker, Elke; Weimer, Marc; Meinicke, Thomas; Woerle, Hans J; Broedl, Uli C; EMPA-REG METSU Trial Investigators Diabetes care / 2013;36(11):3396-404 | Industry trial in a specialty journal |
| Vilsboll 2019 Dapagliflozin Plus Saxagliptin Add-on Therapy Compared With Insulin in Patients With Type 2 Diabetes Poorly Controlled by Metformin With or Without Sulfonylurea Therapy: A Randomized Clinical Trial. Vilsboll, Tina; Ekholm, Ella; Johnsson, Eva; Dronamraju, Nalina; Jabbour, Serge; Lind, Marcus Diabetes care / 2019;42(8):1464-1472 | Industry trial in a specialty journal |
| FLAT-SUGARTrialInvestigators 2016 Glucose Variability in a 26-Week Randomized Comparison of Mealtime Treatment With Rapid-Acting Insulin Versus GLP-1 Agonist in Participants With Type 2 Diabetes at High Cardiovascular Risk. FLAT-SUGAR Trial Investigators Diabetes care / 2016;39(6):973-81 | Industry trial in a specialty journal |
| Fonseca 2008 Colesevelam HCl improves glycemic control and reduces LDL cholesterol in patients with inadequately controlled type 2 diabetes on sulfonylurea-based therapy. Fonseca, Vivian A; Rosenstock, Julio; Wang, Antonia C; Truitt, Kenneth E; Jones, Michael R Diabetes care / 2008;31(8):1479-84 | Industry trial in a specialty journal |
| DePaoli 2014 Can a selective PPARgamma modulator improve glycemic control in patients with type 2 diabetes with fewer side effects compared with pioglitazone?. DePaoli, Alex M; Higgins, Linda S; Henry, Robert R; Mantzoros, Christos; Dunn, Fredrick L; INT131-007 Study Group Diabetes care / 2014;37(7):1918-23 | Industry trial in a specialty journal |
| Haring 2014 Empagliflozin as add-on to metformin in patients with type 2 diabetes: a 24-week, randomized, double-blind, placebo-controlled trial. Haring, Hans-Ulrich; Merker, Ludwig; Seewaldt-Becker, Elke; Weimer, Marc; Meinicke, Thomas; Broedl, Uli C; Woerle, Hans J; EMPA-REG MET Trial Investigators Diabetes care / 2014;37(6):1650-9 | Industry trial in a specialty journal |
| Rosenstock 2014 Advancing basal insulin replacement in type 2 diabetes inadequately controlled with insulin glargine plus oral agents: a comparison of adding albiglutide, a weekly GLP-1 receptor agonist, versus thrice-daily prandial insulin lispro. Rosenstock, Julio; Fonseca, Vivian A; Gross, Jorge L; Ratner, Robert E; Ahren, Bo; Chow, Francis C C; Yang, Fred; Miller, Diane; Johnson, Susan L; Stewart, Murray W; Leiter, Lawrence A; Harmony 6 Study Group Diabetes care / 2014;37(8):2317-25 | Industry trial in a specialty journal |
| Ferrannini 2010 Dapagliflozin monotherapy in type 2 diabetic patients with inadequate glycemic control by diet and exercise: a randomized, double-blind, placebo-controlled, phase 3 trial. Ferrannini, Ele; Ramos, Silvia Jimenez; Salsali, Afshin; Tang, Weihua; List, James F Diabetes care / 2010;33(10):2217-24 | Industry trial in a specialty journal |
| Sandercock 2009 Gabapentin extended release for the treatment of painful diabetic peripheral neuropathy: efficacy and tolerability in a double-blind, randomized, controlled clinical trial. Sandercock, David; Cramer, Marilou; Wu, Jacqueline; Chiang, Yu-Kun; Biton, Victor; Heritier, Michelle Diabetes care / 2009;32(2):e20 | Industry trial in a specialty journal |
| Ridker 2012 Effects of interleukin-1beta inhibition with canakinumab on hemoglobin A1c, lipids, C-reactive protein, interleukin-6, and fibrinogen: a phase IIb randomized, placebo-controlled trial. Ridker, Paul M; Howard, Campbell P; Walter, Verena; Everett, Brendan; Libby, Peter; Hensen, Johannes; Thuren, Tom; CANTOS Pilot Investigative Group Circulation / 2012;126(23):2739-48 | Industry trial in a specialty journal |

| | |
|---|---|
| Sever 2005 Reduction in cardiovascular events with atorvastatin in 2,532 patients with type 2 diabetes: Anglo-Scandinavian Cardiac Outcomes Trial--lipid-lowering arm (ASCOT-LLA). Sever, Peter S; Poulter, Neil R; Dahlof, Bjorn; Wedel, Hans; Collins, Rory; Beevers, Gareth; Caulfield, Mark; Kjeldsen, Sverre E; Kristinsson, Arni; McInnes, Gordon T; Mehlsen, Jesper; Nieminen, Markku; O'Brien, Eoin; Ostergren, Jan Diabetes care / 2005;28(5):1151-7 | Industry trial in a specialty journal |
| Gerich 2005 PRESERVE-beta: two-year efficacy and safety of initial combination therapy with nateglinide or glyburide plus metformin. Gerich, John; Raskin, Philip; Jean-Louis, Lisa; Purkayastha, Das; Baron, Michelle A Diabetes care / 2005;28(9):2093-9 | Industry trial in a specialty journal |
| Charbonnel 2004 The prospective pioglitazone clinical trial in macrovascular events (PROactive): can pioglitazone reduce cardiovascular events in diabetes? Study design and baseline characteristics of 5238 patients. Charbonnel, Bernard; Dormandy, John; Erdmann, Erland; Massi-Benedetti, Massimo; Skene, Allan; PROactive Study Group Diabetes care / 2004;27(7):1647-53 | Industry trial in a specialty journal |
| Rosenstock 2015 Dual add-on therapy in type 2 diabetes poorly controlled with metformin monotherapy: a randomized double-blind trial of saxagliptin plus dapagliflozin addition versus single addition of saxagliptin or dapagliflozin to metformin. Rosenstock, Julio; Hansen, Lars; Zee, Pamela; Li, Yan; Cook, William; Hirshberg, Boaz; Iqbal, Nayyar Diabetes care / 2015;38(3):376-83 | Industry trial in a specialty journal |
| Rosenstock 2016 Initial Combination Therapy With Canagliflozin Plus Metformin Versus Each Component as Monotherapy for Drug-Naive Type 2 Diabetes. Rosenstock, Julio; Chuck, Leonard; Gonzalez-Ortiz, Manuel; Merton, Kate; Craig, Jagriti; Capuano, George; Qiu, Rong Diabetes care / 2016;39(3):353-62 | Industry trial in a specialty journal |
| DeFronzo 2015 Combination of empagliflozin and linagliptin as second-line therapy in subjects with type 2 diabetes inadequately controlled on metformin. DeFronzo, Ralph A; Lewin, Andrew; Patel, Sanjay; Liu, Dacheng; Kaste, Renee; Woerle, Hans J; Broedl, Uli C Diabetes care / 2015;38(3):384-93 | Industry trial in a specialty journal |
| ArjonaFerreira 2013 Efficacy and safety of sitagliptin versus glipizide in patients with type 2 diabetes and moderate-to-severe chronic renal insufficiency. Arjona Ferreira, Juan Camilo; Marre, Michel; Barzilai, Nir; Guo, Hua; Golm, Gregory T; Sisk, Christine McCrary; Kaufman, Keith D; Goldstein, Barry J Diabetes care / 2013;36(5):1067-73 | Industry trial in a specialty journal |
| Rosenstock 2016 Prandial Options to Advance Basal Insulin Glargine Therapy: Testing Lixisenatide Plus Basal Insulin Versus Insulin Glulisine Either as Basal-Plus or Basal-Bolus in Type 2 Diabetes: The GetGoal Duo-2 Trial. Rosenstock, Julio; Guerci, Bruno; Hanefeld, Markolf; Gentile, Sandro; Aronson, Ronnie; Tinahones, Francisco J; Roy-Duval, Christine; Souhami, Elisabeth; Wardecki, Marek; Ye, Jenny; Perfetti, Riccardo; Heller, Simon; GetGoal Duo-2 Trial Investigators Diabetes care / 2016;39(8):1318-28 | Industry trial in a specialty journal |
| Nauck 2016 Once-Daily Liraglutide Versus Lixisenatide as Add-on to Metformin in Type 2 Diabetes: A 26-Week Randomized Controlled Clinical Trial. Nauck, Michael; Rizzo, Manfredi; Johnson, Andrew; Bosch-Traberg, Heidrun; Madsen, Jesper; Cariou, Bertrand Diabetes care / 2016;39(9):1501-9 | Industry trial in a specialty journal |
| Menon 2018 Fasiglifam-Induced Liver Injury in Patients With Type 2 Diabetes: Results of a Randomized Controlled Cardiovascular Outcomes Safety Trial. Menon, Venu; Lincoff, A Michael; Nicholls, Stephen J; Jasper, Susan; Wolski, Kathy; McGuire, Darren K; Mehta, Cyrus R; Rosenstock, Julio; Lopez, Claudia; Marcinak, John; Cao, Charlie; Nissen, Steven E; GRAND 306 Investigators Diabetes care / 2018;41(12):2603-2609 | Industry trial in a specialty journal |
| Ritzel 2018 A Randomized Controlled Trial Comparing Efficacy and Safety of Insulin Glargine 300 Units/mL Versus 100 Units/mL in Older People With Type 2 Diabetes: Results From the SENIOR Study. Ritzel, Robert; Harris, Stewart B; Baron, Helen; Florez, Hermes; Roussel, Ronan; Espinasse, Melanie; Muehlen-Bartmer, Isabel; Zhang, Nianxian; Bertolini, Monica; Brulle-Wohlhueter, Claire; Munshi, Medha; Bolli, Geremia B Diabetes care / 2018;41(8):1672-1680 | Industry trial in a specialty journal |
| Miles 2002 Effect of orlistat in overweight and obese patients with type 2 diabetes treated with metformin. Miles, John M; Leiter, Lawrence; Hollander, Priscilla; Wadden, Thomas; Anderson, James W; Doyle, Michael; Foreyt, John; Aronne, Louis; Klein, Samuel Diabetes care / 2002;25(7):1123-8 | Industry trial in a specialty journal |

| | |
|---|---|
| Rosenstock 2010 Initial combination therapy with alogliptin and pioglitazone in drug-naive patients with type 2 diabetes. Rosenstock, Julio; Inzucchi, Silvio E; Seufert, Jochen; Fleck, Penny R; Wilson, Craig A; Mekki, Qais Diabetes care / 2010;33(11):2406-8 | Industry trial in a specialty journal |
| Gaziano 2010 Randomized clinical trial of quick-release bromocriptine among patients with type 2 diabetes on overall safety and cardiovascular outcomes. Gaziano, J Michael; Cincotta, Anthony H; O'Connor, Christopher M; Ezrokhi, Michael; Rutty, Dean; Ma, Z J; Scranton, Richard E Diabetes care / 2010;33(7):1503-8 | Industry trial in a specialty journal |
| Aschner 2006 Effect of the dipeptidyl peptidase-4 inhibitor sitagliptin as monotherapy on glycemic control in patients with type 2 diabetes. Aschner, Pablo; Kipnes, Mark S; Lunceford, Jared K; Sanchez, Matilde; Mickel, Carolyn; Williams-Herman, Debora E; Sitagliptin Study 021 Group Diabetes care / 2006;29(12):2632-7 | Industry trial in a specialty journal |
| Kendall 2006 Improvement of glycemic control, triglycerides, and HDL cholesterol levels with muraglitazar, a dual (alpha/gamma) peroxisome proliferator-activated receptor activator, in patients with type 2 diabetes inadequately controlled with metformin monotherapy: A Kendall, David M; Rubin, Cindy J; Mohideen, Pharis; Ledeine, Jean-Marie; Belder, Rene; Gross, Jorge; Norwood, Paul; O'Mahony, Michael; Sall, Kenneth; Sloan, Greg; Roberts, Anthony; Fiedorek, Fred T; DeFronzo, Ralph A Diabetes care / 2006;29(5):1016-23 Ref ID: 16644631 | Industry trial in a specialty journal |
| Bastyr 2000 Therapy focused on lowering postprandial glucose, not fasting glucose, may be superior for lowering HbA1c. IOEZ Study Group. Bastyr, E J 3rd; Stuart, C A; Brodows, R G; Schwartz, S; Graf, C J; Zagar, A; Robertson, K E Diabetes care / 2000;23(9):1236-41 | Industry trial in a specialty journal |
| Buse 2011 The DURAbility of Basal versus Lispro mix 75/25 insulin Efficacy (DURABLE) trial: comparing the durability of lispro mix 75/25 and glargine. Buse, John B; Wolffenbuttel, Bruce H R; Herman, William H; Hippler, Stephen; Martin, Sherry A; Jiang, Honghua H; Shenouda, Sylvia K; Fahrbach, Jessie L Diabetes care / 2011;34(2):249-55 | Industry trial in a specialty journal |
| Vilsboll 2007 Liraglutide, a long-acting human glucagon-like peptide-1 analog, given as monotherapy significantly improves glycemic control and lowers body weight without risk of hypoglycemia in patients with type 2 diabetes. Vilsboll, Tina; Zdravkovic, Milan; Le-Thi, Tu; Krarup, Thure; Schmitz, Ole; Courreges, Jean-Pierre; Verhoeven, Robert; Buganova, Ingrid; Madsbad, Sten Diabetes care / 2007;30(6):1608-10 | Industry trial in a specialty journal |
| Janka 2005 Comparison of basal insulin added to oral agents versus twice-daily premixed insulin as initial insulin therapy for type 2 diabetes. Janka, Hans U; Plewe, Gerd; Riddle, Matthew C; Kliebe-Frisch, Christine; Schweitzer, Matthias A; Yki-Jarvinen, Hannele Diabetes care / 2005;28(2):254-9 | Industry trial in a specialty journal |
| Jabbour 2014 Dapagliflozin is effective as add-on therapy to sitagliptin with or without metformin: a 24-week, multicenter, randomized, double-blind, placebo-controlled study. Jabbour, Serge A; Hardy, Elise; Sugg, Jennifer; Parikh, Shamik; Study 10 Group Diabetes care / 2014;37(3):740-50 | Industry trial in a specialty journal |
| Rodbard 2019 Oral Semaglutide Versus Empagliflozin in Patients With Type 2 Diabetes Uncontrolled on Metformin: The PIONEER 2 Trial. Rodbard, Helena W; Rosenstock, Julio; Canani, Luis H; Deerochanawong, Chaicharn; Gumprecht, Janusz; Lindberg, Soren Ostergaard; Lingvay, Ildiko; Sondergaard, Anette Luther; Treppendahl, Marianne Bach; Montanya, Eduard; PIONEER 2 Investigators Diabetes care / 2019;42(12):2272-2281 | Industry trial in a specialty journal |
| Yki-Jarvinen 2013 Effects of adding linagliptin to basal insulin regimen for inadequately controlled type 2 diabetes: a >=52-week randomized, double-blind study. Yki-Jarvinen, Hannele; Rosenstock, Julio; Duran-Garcia, Santiago; Pinnetti, Sabine; Bhattacharya, Sudipta; Thiemann, Sandra; Patel, Sanjay; Woerle, Hans-Juergen Diabetes care / 2013;36(12):3875-81 | Industry trial in a specialty journal |
| Giorgino 2015 Efficacy and Safety of Once-Weekly Dulaglutide Versus Insulin Glargine in Patients With Type 2 Diabetes on Metformin and Glimepiride (AWARD-2). Giorgino, Francesco; Benroubi, Marian; Sun, Jui-Hung; Zimmermann, Alan G; Pechtner, Valeria Diabetes care / 2015;38(12):2241-9 | Industry trial in a specialty journal |
| McGill 2013 Long-term efficacy and safety of linagliptin in patients with type 2 diabetes and severe renal impairment: a 1-year, randomized, double-blind, placebo-controlled study. McGill, Janet B; Sloan, Lance; Newman, Jennifer; Patel, Sanjay; Sauce, Christophe; von Eynatten, Maximilian; Woerle, Hans-Juergen Diabetes care / 2013;36(2):237-44 | Industry trial in a specialty journal |

| | |
|---|---|
| Zinman 2012 Insulin degludec versus insulin glargine in insulin-naive patients with type 2 diabetes: a 1-year, randomized, treat-to-target trial (BEGIN Once Long). Zinman, Bernard; Philis-Tsimikas, Athena; Cariou, Bertrand; Handelsman, Yehuda; Rodbard, Helena W; Johansen, Thue; Endahl, Lars; Mathieu, Chantal; NN1250-3579 (BEGIN Once Long) Trial Investigators Diabetes care / 2012;35(12):2464-71 | Industry trial in a specialty journal |
| Umpierrez 2014 Efficacy and safety of dulaglutide monotherapy versus metformin in type 2 diabetes in a randomized controlled trial (AWARD-3). Umpierrez, Guillermo; Tofe Povedano, Santiago; Perez Manghi, Federico; Shurzinske, Linda; Pechtner, Valeria Diabetes care / 2014;37(8):2168-76 | Industry trial in a specialty journal |
| Fulcher 2014 Comparison of insulin degludec/insulin aspart and biphasic insulin aspart 30 in uncontrolled, insulin-treated type 2 diabetes: a phase 3a, randomized, treat-to-target trial. Fulcher, Gregory R; Christiansen, Jens Sandahl; Bantwal, Ganapathi; Polaszewska-Muszynska, Miroslawa; Mersebach, Henriette; Andersen, Thomas H; Niskanen, Leo K; BOOST: Intensify Premix I Investigators Diabetes care / 2014;37(8):2084-90 | Industry trial in a specialty journal |
| Rosenstock 2018 More Similarities Than Differences Testing Insulin Glargine 300 Units/mL Versus Insulin Degludec 100 Units/mL in Insulin-Naive Type 2 Diabetes: The Randomized Head-to-Head BRIGHT Trial. Rosenstock, Julio; Cheng, Alice; Ritzel, Robert; Bosnyak, Zsolt; Devisme, Christine; Cali, Anna M G; Sieber, Jochen; Stella, Peter; Wang, Xiangling; Frias, Juan P; Roussel, Ronan; Bolli, Geremia B Diabetes care / 2018;41(10):2147-2154 | Industry trial in a specialty journal |
| Rosenstock 2012 Effects of dapagliflozin, an SGLT2 inhibitor, on HbA(1c), body weight, and hypoglycemia risk in patients with type 2 diabetes inadequately controlled on pioglitazone monotherapy. Rosenstock, Julio; Vico, Marisa; Wei, Li; Salsali, Afshin; List, James F Diabetes care / 2012;35(7):1473-8 | Industry trial in a specialty journal |
| Ferdinand 2019 Antihyperglycemic and Blood Pressure Effects of Empagliflozin in Black Patients With Type 2 Diabetes Mellitus and Hypertension. Ferdinand, Keith C; Izzo, Joseph L; Lee, Jisoo; Meng, Leslie; George, Jyothis; Salsali, Afshin; Seman, Leo Circulation / 2019;139(18):2098-2109 | Industry trial in a specialty journal |
| Umpierrez 2013 Randomized study comparing a Basal-bolus with a basal plus correction insulin regimen for the hospital management of medical and surgical patients with type 2 diabetes: basal plus trial. Umpierrez, Guillermo E; Smiley, Dawn; Hermayer, Kathie; Khan, Amna; Olson, Darin E; Newton, Christopher; Jacobs, Sol; Rizzo, Monica; Peng, Limin; Reyes, David; Pinzon, Ingrid; Fereira, Maria Eugenia; Hunt, Vicky; Gore, Ashwini; Toyoshima, Marcos T; Fonseca, Vivian A Diabetes care / 2013;36(8):2169-74 | Industry trial in a specialty journal |
| Nauck 2011 Dapagliflozin versus glipizide as add-on therapy in patients with type 2 diabetes who have inadequate glycemic control with metformin: a randomized, 52-week, double-blind, active-controlled noninferiority trial. Nauck, Michael A; Del Prato, Stefano; Meier, Juris J; Duran-Garcia, Santiago; Rohwedder, Katja; Elze, Martina; Parikh, Shamik J Diabetes care / 2011;34(9):2015-22 | Industry trial in a specialty journal |
| Giugliano 2014 Initiation and gradual intensification of premixed insulin lispro therapy versus Basal {+/-} mealtime insulin in patients with type 2 diabetes eating light breakfasts. Giugliano, Dario; Tracz, Mariusz; Shah, Sanjiv; Calle-Pascual, Alfonso; Mistodie, Cristina; Duarte, Rui; Sari, Ramazan; Woo, Vincent; Jiletcovici, Alina O; Deinhard, Jurgen; Wille, Simone A; Kiljanski, Jacek Diabetes care / 2014;37(2):372-80 | Industry trial in a specialty journal |
| Nauck 2009 Efficacy and safety comparison of liraglutide, glimepiride, and placebo, all in combination with metformin, in type 2 diabetes: the LEAD (liraglutide effect and action in diabetes)-2 study. Nauck, Michael; Frid, Anders; Hermansen, Kjeld; Shah, Nalini S; Tankova, Tsvetalina; Mitha, Ismail H; Zdravkovic, Milan; During, Maria; Matthews, David R; LEAD-2 Study Group Diabetes care / 2009;32(1):84-90 1 | Industry trial in a specialty journal |
| Matthaei 2015 Dapagliflozin improves glycemic control and reduces body weight as add-on therapy to metformin plus sulfonylurea: a 24-week randomized, double-blind clinical trial. Matthaei, Stephan; Bowering, Keith; Rohwedder, Katja; Grohl, Anke; Parikh, Shamik; Study 05 Group Diabetes care / 2015;38(3):365-72 | Industry trial in a specialty journal |
| Rosenstock 2001 Basal insulin therapy in type 2 diabetes: 28-week comparison of insulin glargine (HOE 901) and NPH insulin. Rosenstock, J; Schwartz, S L; Clark, C M Jr; Park, G D; Donley, D W; Edwards, M B Diabetes care / 2001;24(4):631-6 | Industry trial in a specialty journal |

| | |
|---|---|
| Hollander 2003 Pramlintide as an adjunct to insulin therapy improves long-term glycemic and weight control in patients with type 2 diabetes: a 1-year randomized controlled trial. Hollander, Priscilla A; Levy, Philip; Fineman, Mark S; Maggs, David G; Shen, Larry Z; Strobel, Susan A; Weyer, Christian; Kolterman, Orville G Diabetes care / 2003;26(3):784-90 | Industry trial in a specialty journal |
| Deeg 2007 Pioglitazone and rosiglitazone have different effects on serum lipoprotein particle concentrations and sizes in patients with type 2 diabetes and dyslipidemia. Deeg, Mark A; Buse, John B; Goldberg, Ronald B; Kendall, David M; Zagar, Anthony J; Jacober, Scott J; Khan, Mehmood A; Perez, Alfonzo T; Tan, Meng H; GLAI Study Investigators Diabetes care / 2007;30(10):2458-64 | Industry trial in a specialty journal |
| Nauck 2016 A Phase 2, Randomized, Dose-Finding Study of the Novel Once-Weekly Human GLP-1 Analog, Semaglutide, Compared With Placebo and Open-Label Liraglutide in Patients With Type 2 Diabetes. Nauck, Michael A; Petrie, John R; Sesti, Giorgio; Mannucci, Edoardo; Courreges, Jean-Pierre; Lindegaard, Marie L; Jensen, Christine B; Atkin, Stephen L; Study 1821 Investigators Diabetes care / 2016;39(2):231-41 | Industry trial in a specialty journal |
| Leiter 2014 Efficacy and safety of the once-weekly GLP-1 receptor agonist albiglutide versus sitagliptin in patients with type 2 diabetes and renal impairment: a randomized phase III study. Leiter, Lawrence A; Carr, Molly C; Stewart, Murray; Jones-Leone, Angela; Scott, Rhona; Yang, Fred; Handelsman, Yehuda Diabetes care / 2014;37(10):2723-30 | Industry trial in a specialty journal |
| Laakso 2015 Treatment with the dipeptidyl peptidase-4 inhibitor linagliptin or placebo followed by glimepiride in patients with type 2 diabetes with moderate to severe renal impairment: a 52-week, randomized, double-blind clinical trial. Laakso, Markku; Rosenstock, Julio; Groop, Per-Henrik; Barnett, Anthony H; Gallwitz, Baptist; Hehnke, Uwe; Tamminen, Ilkka; Patel, Sanjay; von Eynatten, Maximilian; Woerle, Hans-Juergen Diabetes care / 2015;38(2):e15-7 | Industry trial in a specialty journal |
| Meininger 2011 Effects of MK-0941, a novel glucokinase activator, on glycemic control in insulin-treated patients with type 2 diabetes. Meininger, Gary E; Scott, Russell; Alba, Maria; Shentu, Yue; Luo, Edmund; Amin, Himal; Davies, Michael J; Kaufman, Keith D; Goldstein, Barry J Diabetes care / 2011;34(12):2560-6 | Industry trial in a specialty journal |
| Riddle 2003 The treat-to-target trial: randomized addition of glargine or human NPH insulin to oral therapy of type 2 diabetic patients. Riddle, Matthew C; Rosenstock, Julio; Gerich, John; Insulin Glargine 4002 Study Investigators Diabetes care / 2003;26(11):3080-6 | Industry trial in a specialty journal |
| Hadjadj 2016 Initial Combination of Empagliflozin and Metformin in Patients With Type 2 Diabetes. Hadjadj, Samy; Rosenstock, Julio; Meinicke, Thomas; Woerle, Hans J; Broedl, Uli C Diabetes care / 2016;39(10):1718-28 | Industry trial in a specialty journal |
| Aroda 2019 PIONEER 1: Randomized Clinical Trial of the Efficacy and Safety of Oral Semaglutide Monotherapy in Comparison With Placebo in Patients With Type 2 Diabetes. Aroda, Vanita R; Rosenstock, Julio; Terauchi, Yasuo; Altuntas, Yuksel; Lalic, Nebojsa M; Morales Villegas, Enrique C; Jeppesen, Ole K; Christiansen, Erik; Hertz, Christin L; Haluzik, Martin; PIONEER 1 Investigators Diabetes care / 2019;42(9):1724-1732 | Industry trial in a specialty journal |
| Nauck 2009 Treatment with the human once-weekly glucagon-like peptide-1 analog taspoglutide in combination with metformin improves glycemic control and lowers body weight in patients with type 2 diabetes inadequately controlled with metformin alone: a double-blind p Nauck, Michael A; Ratner, Robert E; Kapitza, Christoph; Berria, Rachele; Boldrin, Mark; Balena, Raffaella Diabetes care / 2009;32(7):1237-43 | Industry trial in a specialty journal |
| Lingvay 2018 A 26-Week Randomized Controlled Trial of Semaglutide Once Daily Versus Liraglutide and Placebo in Patients With Type 2 Diabetes Suboptimally Controlled on Diet and Exercise With or Without Metformin. Lingvay, Ildiko; Desouza, Cyrus V; Lalic, Katarina S; Rose, Ludger; Hansen, Thomas; Zacho, Jeppe; Pieber, Thomas R Diabetes care / 2018;41(9):1926-1937 | Industry trial in a specialty journal |
| Rosenstock 2020 Impact of a Weekly Glucagon-Like Peptide 1 Receptor Agonist, Albiglutide, on Glycemic Control and on Reducing Prandial Insulin Use in Type 2 Diabetes Inadequately Controlled on Multiple Insulin Therapy: A Randomized Trial. Rosenstock, Julio; Nino, Antonio; Soffer, Joseph; Erskine, Lois; Acusta, Andre; Dole, Jo; Carr, Molly C; Mallory, Jason; Home, Philip Diabetes care / 2020;43(10):2509-2518 | Industry trial in a specialty journal |

| | |
|---|---|
| Ferrannini 2013 Long-term safety and efficacy of empagliflozin, sitagliptin, and metformin: an active-controlled, parallel-group, randomized, 78-week open-label extension study in patients with type 2 diabetes. Ferrannini, Ele; Berk, Andreas; Hantel, Stefan; Pinnetti, Sabine; Hach, Thomas; Woerle, Hans J; Broedl, Uli C Diabetes care / 2013;36(12):4015-21 | Industry trial in a specialty journal |
| Goldstein 2007 Effect of initial combination therapy with sitagliptin, a dipeptidyl peptidase-4 inhibitor, and metformin on glycemic control in patients with type 2 diabetes. Goldstein, Barry J; Feinglos, Mark N; Lunceford, Jared K; Johnson, Jeremy; Williams-Herman, Debora E; Sitagliptin 036 Study Group Diabetes care / 2007;30(8):1979-87 | Industry trial in a specialty journal |
| McNulty 2003 A randomized trial of sibutramine in the management of obese type 2 diabetic patients treated with metformin. McNulty, Steven J; Ur, Ehud; Williams, Gareth; Multicenter Sibutramine Study Group Diabetes care / 2003;26(1):125-31 | Industry trial in a specialty journal |
| Knopp 2006 Efficacy and safety of atorvastatin in the prevention of cardiovascular end points in subjects with type 2 diabetes: the Atorvastatin Study for Prevention of Coronary Heart Disease Endpoints in non-insulin-dependent diabetes mellitus (ASPEN). Knopp, Robert H; d'Emden, Michael; Smilde, Johan G; Pocock, Stuart J Diabetes care / 2006;29(7):1478-85 | Industry trial in a specialty journal |
| Riddle 2014 New insulin glargine 300 units/mL versus glargine 100 units/mL in people with type 2 diabetes using basal and mealtime insulin: glucose control and hypoglycemia in a 6-month randomized controlled trial (EDITION 1). Riddle, Matthew C; Bolli, Geremia B; Ziemen, Monika; Muehlen-Bartmer, Isabel; Bizet, Florence; Home, Philip D; EDITION 1 Study Investigators Diabetes care / 2014;37(10):2755-62 | Industry trial in a specialty journal |
| Rosenstock 2002 Combination therapy with nateglinide and a thiazolidinedione improves glycemic control in type 2 diabetes. Rosenstock, Julio; Shen, Sharen G; Gatlin, Marjorie R; Foley, James E Diabetes care / 2002;25(9):1529-33 | Industry trial in a specialty journal |
| DeVries 2012 Sequential intensification of metformin treatment in type 2 diabetes with liraglutide followed by randomized addition of basal insulin prompted by A1C targets. DeVries, J Hans; Bain, Stephen C; Rodbard, Helena W; Seufert, Jochen; D'Alessio, David; Thomsen, Anne B; Zychma, Marcin; Rosenstock, Julio; Liraglutide-Detemir Study Group Diabetes care / 2012;35(7):1446-54 | Industry trial in a specialty journal |
| DeFronzo 2008 Efficacy and safety of the dipeptidyl peptidase-4 inhibitor alogliptin in patients with type 2 diabetes and inadequate glycemic control: a randomized, double-blind, placebo-controlled study. DeFronzo, Ralph A; Fleck, Penny R; Wilson, Craig A; Mekki, Qais; Alogliptin Study 010 Group Diabetes care / 2008;31(12):2315-7 | Industry trial in a specialty journal |
| Zinman 2019 Efficacy, Safety, and Tolerability of Oral Semaglutide Versus Placebo Added to Insulin With or Without Metformin in Patients With Type 2 Diabetes: The PIONEER 8 Trial. Zinman, Bernard; Aroda, Vanita R; Buse, John B; Cariou, Bertrand; Harris, Stewart B; Hoff, Soren Tetens; Pedersen, Karen Boje; Tarp-Johansen, Mads Jeppe; Araki, Eiichi; PIONEER 8 Investigators Diabetes care / 2019;42(12):2262-2271 | Industry trial in a specialty journal |
| Rosenstock 2009 Potential of albiglutide, a long-acting GLP-1 receptor agonist, in type 2 diabetes: a randomized controlled trial exploring weekly, biweekly, and monthly dosing. Rosenstock, Julio; Reusch, Jane; Bush, Mark; Yang, Fred; Stewart, Murray; Albiglutide Study Group Diabetes care / 2009;32(10):1880-6 | Industry trial in a specialty journal |
| Rosenstock 2016 Efficacy and Safety of LixiLan, a Titratable Fixed-Ratio Combination of Lixisenatide and Insulin Glargine, Versus Insulin Glargine in Type 2 Diabetes Inadequately Controlled on Metformin Monotherapy: The LixiLan Proof-of-Concept Randomized Trial. Rosenstock, Julio; Diamant, Michaela; Aroda, Vanita R; Silvestre, Louise; Souhami, Elisabeth; Zhou, Tianyue; Perfetti, Riccardo; Fonseca, Vivian; LixiLan PoC Study Group Diabetes care / 2016;39(9):1579-86 | Industry trial in a specialty journal |
| Goldberg 2005 A comparison of lipid and glycemic effects of pioglitazone and rosiglitazone in patients with type 2 diabetes and dyslipidemia. Goldberg, Ronald B; Kendall, David M; Deeg, Mark A; Buse, John B; Zagar, Anthony J; Pinaire, Jane A; Tan, Meng H; Khan, Mehmood A; Perez, Alfonso T; Jacober, Scott J; GLAI Study Investigators Diabetes care / 2005;28(7):1547-54 | Industry trial in a specialty journal |
| Barnett 2006 An open, randomized, parallel-group study to compare the efficacy and safety profile of inhaled human insulin (Exubera) with glibenclamide as adjunctive therapy in patients with type 2 diabetes poorly controlled on metformin. Barnett, Anthony H; Dreyer, Manfred; Lange, Peter; Serdarevic-Pehar, Marjana Diabetes care / 2006;29(8):1818-25 Ref ID: 16873786 | Industry trial in a specialty journal |

| | |
|---|---|
| Moses 2001 Flexible meal-related dosing with repaglinide facilitates glycemic control in therapy-naive type 2 diabetes. Moses, R G; Gomis, R; Frandsen, K B; Schlienger, J L; Dedov, I Diabetes care / 2001;24(1):11-5 | Industry trial in a specialty journal |
| Blonde 2019 Switching to iGlarLixi Versus Continuing Daily or Weekly GLP-1 RA in Type 2 Diabetes Inadequately Controlled by GLP-1 RA and Oral Antihyperglycemic Therapy: The LixiLan-G Randomized Clinical Trial. Blonde, Lawrence; Rosenstock, Julio; Del Prato, Stefano; Henry, Robert; Shehadeh, Naim; Frias, Juan; Niemoeller, Elisabeth; Souhami, Elisabeth; Ji, Chen; Aroda, Vanita R Diabetes care / 2019;42(11):2108-2116 | Industry trial in a specialty journal |
| Rosenstock 2015 Inhaled Technosphere Insulin Versus Inhaled Technosphere Placebo in Insulin-Naive Subjects With Type 2 Diabetes Inadequately Controlled on Oral Antidiabetes Agents. Rosenstock, Julio; Franco, Denise; Korpachev, Vadym; Shumel, Brad; Ma, Yuhui; Baughman, Robert; Amin, Nikhil; McGill, Janet B; Affinity 2 Study Group Diabetes care / 2015;38(12):2274-81 | Industry trial in a specialty journal |
| Ahren 2013 Efficacy and safety of lixisenatide once-daily morning or evening injections in type 2 diabetes inadequately controlled on metformin (GetGoal-M). Ahren, Bo; Leguizamo Dimas, Aniceto; Miossec, Patrick; Saubadu, Stephane; Aronson, Ronnie Diabetes care / 2013;36(9):2543-50 | Industry trial in a specialty journal |
| List 2009 Sodium-glucose cotransport inhibition with dapagliflozin in type 2 diabetes. List, James F; Woo, Vincent; Morales, Enrique; Tang, Weihua; Fiedorek, Fred T Diabetes care / 2009;32(4):650-7 | Industry trial in a specialty journal |
| Jabbour 2018 Safety and Efficacy of Exenatide Once Weekly Plus Dapagliflozin Once Daily Versus Exenatide or Dapagliflozin Alone in Patients With Type 2 Diabetes Inadequately Controlled With Metformin Monotherapy: 52-Week Results of the DURATION-8 Randomized Controlled Jabbour, Serge A; Frias, Juan P; Hardy, Elise; Ahmed, Azazuddin; Wang, Hui; Ohman, Peter; Guja, Cristian Diabetes care / 2018;41(10):2136-2146 | Industry trial in a specialty journal |
| Bosi 2007 Effects of vildagliptin on glucose control over 24 weeks in patients with type 2 diabetes inadequately controlled with metformin. Bosi, Emanuele; Camisasca, Riccardo Paolo; Collober, Carole; Rochotte, Erika; Garber, Alan J Diabetes care / 2007;30(4):890-5 | Industry trial in a specialty journal |
| Viberti 2002 Microalbuminuria reduction with valsartan in patients with type 2 diabetes mellitus: a blood pressure-independent effect. Viberti, Giancarlo; Wheeldon, Nigel M; MicroAlbuminuria Reduction With VALsartan (MARVAL) Study Investigators Circulation / 2002;106(6):672-8 | Industry trial in a specialty journal |
| Rosenstock 2019 Once-Weekly Efpeglenatide Dose-Range Effects on Glycemic Control and Body Weight in Patients With Type 2 Diabetes on Metformin or Drug Naive, Referenced to Liraglutide. Rosenstock, Julio; Sorli, Christopher H; Trautmann, Michael E; Morales, Cristobal; Wendisch, Ulrich; Dailey, George; Hompesch, Marcus; Choi, In Young; Kang, Jahoon; Stewart, John; Yoon, Kun-Ho Diabetes care / 2019;42(9):1733-1741 | Industry trial in specialty journal |
| Buse 2009 DURAbility of basal versus lispro mix 75/25 insulin efficacy (DURABLE) trial 24-week results: safety and efficacy of insulin lispro mix 75/25 versus insulin glargine added to oral antihyperglycemic drugs in patients with type 2 diabetes. Buse, John B; Wolffenbuttel, Bruce H R; Herman, William H; Shemonsky, Natalie K; Jiang, Honghua H; Fahrbach, Jessie L; Scism-Bacon, Jamie L; Martin, Sherry A Diabetes care / 2009;32(6):1007-13 | Industry trial in a specialty journal |
| Fouqueray 2014 The efficacy and safety of imeglimin as add-on therapy in patients with type 2 diabetes inadequately controlled with sitagliptin monotherapy. Fouqueray, Pascale; Pirags, Valdis; Diamant, Michaela; Schernthaner, Guntram; Lebovitz, Harold E; Inzucchi, Silvio E; Bailey, Clifford J Diabetes care / 2014;37(7):1924-30 1 | Industry trial in a specialty journal |
| Rosenstock 2008 SERENADE: the Study Evaluating Rimonabant Efficacy in Drug-naive Diabetic Patients: effects of monotherapy with rimonabant, the first selective CB1 receptor antagonist, on glycemic control, body weight, and lipid profile in drug-naive type 2 diabetes. Rosenstock, Julio; Hollander, Priscilla; Chevalier, Soazig; Iranmanesh, Ali; SERENADE Study Group Diabetes care / 2008;31(11):2169-76 | Industry trial in a specialty journal |
| Horton 2000 Nateglinide alone and in combination with metformin improves glycemic control by reducing mealtime glucose levels in type 2 diabetes. Horton, E S; Clinkingbeard, C; Gatlin, M; Foley, J; Mallows, S; Shen, S Diabetes care / 2000;23(11):1660-5 United States 2000 | Industry trial in specialty journal |

| | |
|---|---|
| Jia 2015 Comparison of thrice-daily premixed insulin (insulin lispro premix) with basal-bolus (insulin glargine once-daily plus thrice-daily prandial insulin lispro) therapy in east Asian parents with type 2 diabetes insufficiently controlled with twice-daily pre. Xiao, X; Ji, Q; Ahn, K; Chuang, L; Bao, Y; Pan, C; Chen, L; Gao, F; Tu, Y; Li, P; Yang, J. The Lancet Diabetes and Endocrinology / 2015;3(4):254-62 | Industry trial in a specialty journal |
| Arnold 2014 Effects of ranolazine on quality of life among patients with diabetes mellitus and stable angina. Arnold, SV; Kosiborod, M; McGuire, DK; Li, Y; Yue, P; Ben-Yehuda, O; Spertus, JA JAMA Internal Medicine / 2014;174(8):1403-5 | Industry trial in a specialty journal |
| **Less than 100 participants** | **22** |
| Hansen 2015 The effect of metformin on glucose homeostasis during moderate exercise. Hansen, Merethe; Palsoe, Marie K; Helge, Jorn W; Dela, Flemming Diabetes care / 2015;38(2):293-301 | Less than 100 participants |
| Baba 2001 The oral insulin sensitizer, thiazolidinedione, increases plasma vascular endothelial growth factor in type 2 diabetic patients. Baba, T; Shimada, K; Neugebauer, S; Yamada, D; Hashimoto, S; Watanabe, T Diabetes care / 2001;24(5):953-4 | Less than 100 participants |
| Chen 2008 Beneficial effects of insulin on glycemic control and beta-cell function in newly diagnosed type 2 diabetes with severe hyperglycemia after short-term intensive insulin therapy. Chen, Harn-Shen; Wu, Tzu-En; Jap, Tjin-Shing; Hsiao, Li-Chuan; Lee, Shen-Hung; Lin, Hong-Da Diabetes care / 2008;31(10):1927-32 | Less than 100 participants |
| Morgan 2019 Antisense Inhibition of Glucagon Receptor by IONIS-GCGRRx Improves Type 2 Diabetes Without Increase in Hepatic Glycogen Content in Patients With Type 2 Diabetes on Stable Metformin Therapy. Morgan, Erin S; Tai, Li-Jung; Pham, Nguyen C; Overman, Julia K; Watts, Lynnetta M; Smith, Anne; Jung, Shiangtung W; Gajdosik, Martin; Krssak, Martin; Krebs, Michael; Geary, Richard S; Baker, Brenda F; Bhanot, Sanjay Diabetes care / 2019;42(4):585-593 | Less than 100 participants |
| Nellemann 2007 Simvastatin reduces plasma osteoprotegerin in type 2 diabetic patients with microalbuminuria. Nellemann, Birgitte; Gormsen, Lars C; Dollerup, Jens; Schmitz, Ole; Mogensen, Carl E; Rasmussen, Lars M; Nielsen, Soren Diabetes care / 2007;30(12):3122-4 | Less than 100 participants |
| Armstrong 2016 Liraglutide safety and efficacy in patients with non-alcoholic steatohepatitis (LEAN): a multicentre, double-blind, randomised, placebo-controlled phase 2 study. Armstrong, Matthew James; Gaunt, Piers; Aithal, Guruprasad P; Barton, Darren; Hull, Diana; Parker, Richard; Hazlehurst, Jonathan M; Guo, Kathy; LEAN trial team; Abouda, George; Aldersley, Mark A; Stocken, Deborah; Gough, Stephen C; Tomlinson, Jeremy W; Brown, Rachel M; Hubscher, Stefan G; Newsome, Philip N Lancet (London, England) / 2016;387(10019):679-690 England 2016 | Less than 100 participants |
| Papa 2006 Safety of type 2 diabetes treatment with repaglinide compared with glibenclamide in elderly people: A randomized, open-label, two-period, cross-over trial. Papa, Giuseppe; Fedele, Viviana; Rizzo, Maria Rosaria; Fioravanti, Marisa; Leotta, Carmelo; Solerte, Sebastiano Bruno; Purrello, Francesco; Paolisso, Giuseppe Diabetes care / 2006;29(8):1918-20 | Less than 100 participants |
| Peacey 2000 Does the choice of treatment for type 2 diabetes affect the physiological response to hypoglycemia?. Peacey, S R; Robinson, R; Bedford, C; Harris, N D; Macdonald, I A; Holman, R R; Heller, S R Diabetes care / 2000;23(7):1022-3 | Less than 100 participants |
| Rudovich 2015 Effect of exogenous intravenous administrations of GLP-1 and/or GIP on circulating pro-atrial natriuretic peptide in subjects with different stages of glucose tolerance. Rudovich, Natalia; Pivovarova, Olga; Gogebakan, Ozlem; Sparwasser, Andrea; Doehner, Wolfram; Anker, Stefan D; Arafat, Ayman M; Bergmann, Andreas; Nauck, Michael A; Pfeiffer, Andreas F H Diabetes care / 2015;38(1):e7-8 | Less than 100 participants |
| Striepe 2017 Effects of the Selective Sodium-Glucose Cotransporter 2 Inhibitor Empagliflozin on Vascular Function and Central Hemodynamics in Patients With Type 2 Diabetes Mellitus. Striepe, Kristina; Jumar, Agnes; Ott, Christian; Karg, Marina V; Schneider, Markus P; Kannenkeril, Dennis; Schmieder, Roland E Circulation / 2017;136(12):1167-1169 | Less than 100 participants |
| Herman 2005 A clinical trial of continuous subcutaneous insulin infusion versus multiple daily injections in older adults with type 2 diabetes. Herman, William H; Ilag, Liza L; Johnson, Susan L; Martin, Catherine L; Sinding, Joyce; Al Harthi, Abdulaziz; Plunkett, Cynthia D; LaPorte, Frankie B; Burke, Ray; Brown, Morton B; Halter, Jeffery B; Raskin, Philip Diabetes care / 2005;28(7):1568-73 | Less than 100 participants |

| | |
|---|---|
| Tai 2000 Effect of chitosan on plasma lipoprotein concentrations in type 2 diabetic subjects with hypercholesterolemia. Tai, T S; Sheu, W H; Lee, W J; Yao, H T; Chiang, M T Diabetes care / 2000;23(11):1703-4 | Less than 100 participants |
| Issa 2003 Effect of 2-week treatment with pirenzepine on fasting and postprandial glucose concentrations in individuals with type 2 diabetes. Issa, Basil G; Davies, Nichola; Hood, Kerenza; Premawardhana, Lakdasa D K E; Peters, John R; Scanlon, Maurice F Diabetes care / 2003;26(5):1636-7 | Less than 100 participants |
| Piatti 2001 Long-term oral L-arginine administration improves peripheral and hepatic insulin sensitivity in type 2 diabetic patients. Piatti, P M; Monti, L D; Valsecchi, G; Magni, F; Setola, E; Marchesi, F; Galli-Kienle, M; Pozza, G; Alberti, K G Diabetes care / 2001;24(5):875-80 | Less than 100 participants |
| Kloos 2007 Flexible intensive versus conventional insulin therapy in insulin-naive adults with type 2 diabetes: an open-label, randomized, controlled, crossover clinical trial of metabolic control and patient preference. Kloos, Christof; Samann, Alexander; Lehmann, Thomas; Braun, Anke; Heckmann, Barbara; Muller, Ulrich A Diabetes care / 2007;30(12):3031-2 | Less than 100 participants |
| Ceriello 2014 Simultaneous GLP-1 and insulin administration acutely enhances their vasodilatory, antiinflammatory, and antioxidant action in type 2 diabetes. Ceriello, Antonio; Novials, Anna; Canivell, Silvia; La Sala, Lucia; Pujadas, Gemma; Esposito, Katherine; Testa, Roberto; Bucciarelli, Loredana; Rondinelli, Maurizio; Genovese, Stefano Diabetes care / 2014;37(7):1938-43 | Less than 100 participants |
| Lovshin 2015 Liraglutide promotes natriuresis but does not increase circulating levels of atrial natriuretic peptide in hypertensive subjects with type 2 diabetes. Lovshin, Julie A; Barnie, Annette; DeAlmeida, Ariana; Logan, Alexander; Zinman, Bernard; Drucker, Daniel J Diabetes care / 2015;38(1):132-9 | Less than 100 participants |
| Mari 2007 Effects of the long-acting human glucagon-like peptide-1 analog liraglutide on beta-cell function in normal living conditions. Mari, Andrea; Degn, Kristine; Brock, Birgitte; Rungby, Joergen; Ferrannini, Ele; Schmitz, Ole Diabetes care / 2007;30(8):2032-3 | Less than 100 participants |
| Cefalu 2001 Inhaled human insulin treatment in patients with type 2 diabetes mellitus. Cefalu, W T; Skyler, J S; Kourides, I A; Landschulz, W H; Balagtas, C C; Cheng, S; Gelfand, R A; Inhaled Insulin Study Group Annals of internal medicine / 2001;134(3):203-7 | Less than 100 participants |
| Rave 2007 Coverage of postprandial blood glucose excursions with inhaled technosphere insulin in comparison to subcutaneously injected regular human insulin in subjects with type 2 diabetes. Rave, Klaus; Heise, Tim; Pfutzner, Andreas; Boss, Anders H Diabetes care / 2007;30(9):2307-8 | Less than 100 participants |
| Mangiacapra 2016 Clopidogrel Versus Ticagrelor for Antiplatelet Maintenance in Diabetic Patients Treated With Percutaneous Coronary Intervention: Results of the CLOTILDIA Study (Clopidogrel High Dose Versus Ticagrelor for Antiplatelet Maintenance in Diabetic Patients). Mangiacapra, Fabio; Panaioli, Elena; Colaiori, Iginio; Ricottini, Elisabetta; Lauria Pantano, Angelo; Pozzilli, Paolo; Barbato, Emanuele; Di Sciascio, Germano Circulation / 2016;134(11):835-7 | Less than 100 participants |
| Honisett 2003 Rosiglitazone lowers blood pressure and increases arterial compliance in postmenopausal women with type 2 diabetes. Honisett, Suzy Y; Stojanovska, Lily; Sudhir, Krishnankutty; Kingwell, Bronwyn A; Dawood, Tye; Komesaroff, Paul A Diabetes care / 2003;26(11):3194-5 | Less than 100 participants |
| **No participant breakdown by race/ethnicity** | **12** |
| ADVANCECollaborativeGroup 2008 Intensive blood glucose control and vascular outcomes in patients with type 2 diabetes. ADVANCE Collaborative Group; Patel, Anushka; MacMahon, Stephen; Chalmers, John; Neal, Bruce; Billot, Laurent; Woodward, Mark; Marre, Michel; Cooper, Mark; Glasziou, Paul; Grobbee, Diederick; Hamet, Pavel; Harrap, Stephen; Heller, Simon; Liu, Lisheng; Mancia, Giuseppe; Mogensen, Carl Erik; Pan, Changyu; Poulter, Neil; Rodgers, Anthony; Williams, Bryan; Bompoint, Severine; de Galan, Bastiaan E; Joshi, Rohina; Travert, Florence The New England journal of medicine / 2008;358(24):2560-72 | No participant breakdown by race/ethnicity |
| Bretzel 2008 Once-daily basal insulin glargine versus thrice-daily prandial insulin lispro in people with type 2 diabetes on oral hypoglycaemic agents (APOLLO): an open randomised controlled trial. Bretzel, Reinhard G; Nuber, Ulrike; Landgraf, Wolfgang; Owens, David R; Bradley, Clare; Linn, Thomas Lancet (London, England) / 2008;371(9618):1073-84 England 2008 / 1 | No participant breakdown by race/ethnicity |

| | |
|---|---|
| Haritoglou 2009 Effect of calcium dobesilate on occurrence of diabetic macular oedema (CALDIRET study): randomised, double-blind, placebo-controlled, multicentre trial. Haritoglou, Christos; Gerss, Joachim; Sauerland, Cristina; Kampik, Anselm; Ulbig, Michael W; CALDIRET study group Lancet (London, England) / 2009;373(9672):1364-71 England 2009 / | No participant breakdown by race/ethnicity |
| Patel 2007 Effects of a fixed combination of perindopril and indapamide on macrovascular and microvascular outcomes in patients with type 2 diabetes mellitus (the ADVANCE trial): a randomised controlled trial. Patel, Anushka; ADVANCE Collaborative Group; MacMahon, S; Chalmers, J; Neal, B; Woodward, M; Billot, L; Harrap, S; Poulter, N; Marre, M; Cooper, M; Glasziou, P; Grobbee, D E; Hamet, P; Heller, S; Liu, L S; Mancia, G; Mogensen, C E; Pan, C Y; Rodgers, A; Williams, B Lancet (London, England) / 2007;370(9590):829-40 England 2007 / | No participant breakdown by race/ethnicity |
| Aschner 2012 Insulin glargine versus sitagliptin in insulin-naive patients with type 2 diabetes mellitus uncontrolled on metformin (EASIE): a multicentre, randomised open-label trial. Aschner, Pablo; Chan, Juliana; Owens, David R; Picard, Sylvie; Wang, Edward; Dain, Marie-Paule; Pilorget, Valerie; Echtay, Akram; Fonseca, Vivian; EASIE investigators Lancet (London, England) / 2012;379(9833):2262-9 England 2012 / | No participant breakdown by race/ethnicity |
| Estacio 2000 Effect of blood pressure control on diabetic microvascular complications in patients with hypertension and type 2 diabetes. Estacio, R O; Jeffers, B W; Gifford, N; Schrier, R W Diabetes care / 2000;23 Suppl 2(eag, 7805975):B54-64 | No participant breakdown by race/ethnicity |
| Athyros 2002 Atorvastatin and micronized fenofibrate alone and in combination in type 2 diabetes with combined hyperlipidemia. Athyros, Vasilios G; Papageorgiou, Athanasios A; Athyrou, Valasia V; Demitriadis, Dimokritos S; Kontopoulos, Athanasios G Diabetes care / 2002;25(7):1198-202 | No participant breakdown by race/ethnicity |
| Ahren 2004 Twelve- and 52-week efficacy of the dipeptidyl peptidase IV inhibitor LAF237 in metformin-treated patients with type 2 diabetes. Ahren, Bo; Gomis, Ramon; Standl, Eberhard; Mills, David; Schweizer, Anja Diabetes care / 2004;27(12):2874-80 | No participant breakdown by race/ethnicity |
| Henry 2009 Effect of the dual peroxisome proliferator-activated receptor-alpha/gamma agonist aleglitazar on risk of cardiovascular disease in patients with type 2 diabetes (SYNCHRONY): a phase II, randomised, dose-ranging study. Henry, Robert R; Lincoff, A Michael; Mudaliar, Sunder; Rabbia, Michael; Chognot, Cathy; Herz, Matthias Lancet (London, England) / 2009;374(9684):126-35 England 2009 | No participant breakdown by race/ethnicity |
| Rosenstock 2005 Inhaled insulin improves glycemic control when substituted for or added to oral combination therapy in type 2 diabetes: a randomized controlled trial. Rosenstock, Julio; Zinman, Bernard; Murphy, Liam J; Clement, Stephen C; Moore, Paul; Bowering, C Keith; Hendler, Rosa; Lan, Shu-Ping; Cefalu, William T Annals of internal medicine / 2005;143(8):549-58 United States 2005 | No participant breakdown by race/ethnicity |
| Fritsche 2003 Glimepiride combined with morning insulin glargine, bedtime neutral protamine hagedorn insulin, or bedtime insulin glargine in patients with type 2 diabetes. A randomized, controlled trial. Fritsche, Andreas; Schweitzer, Matthias Axel; Haring, Hans-Ulrich; 4001 Study Group Annals of internal medicine / 2003;138(12):952-9 United States 2003 | No participant breakdown by race/ethnicity |
| Tofte 2020 Early detection of diabetic kidney disease by urinary proteomics and subsequent intervention with spironolactone to delay progression (PRIORITY): a prospective observational study and embedded randomised placebo-controlled trial. Tofte, Nete; Lindhardt, Morten; Adamova, Katarina; Bakker, Stephan J L; Beige, Joachim; Beulens, Joline W J; Birkenfeld, Andreas L; Currie, Gemma; Delles, Christian; Dimos, Ingo; Francova, Lidmila; Frimodt-Moller, Marie; Girman, Peter; Goke, Rudiger; Havrdova, Tereza; Heerspink, Hiddo J L; Kooy, Adriaan; Laverman, Gozewijn D; Mischak, Harald; Navis, Gerjan; Nijpels, Giel; Noutsou, Marina; Ortiz, Alberto; Parvanova, Aneliya; Persson, Frederik; Petrie, John R; Ruggenenti, Piero L; Rutters, Femke; Rychlik, Ivan; Siwy, Justyna; Spasovski, Goce; Speeckaert, Marijn; Trillini, Matias; | No participant breakdown by race/ethnicity |

| | |
|---|---|
| Zurbig, Petra; von der Leyen, Heiko; Rossing, Peter The lancet. Diabetes & endocrinology / 2020;8(4):301-312 | |
| **Unclear ethnic breakdown** | **2** |
| Rosenstock 2020 Once-Weekly Insulin for Type 2 Diabetes without Previous Insulin Treatment. Rosenstock, Julio; Bajaj, Harpreet S; Janez, Andrej; Silver, Robert; Begtrup, Kamilla; Hansen, Melissa V; Jia, Ting; Goldenberg, Ronald; NN1436-4383 Investigators<br>The New England journal of medicine / 2020;383(22):2107-2116<br>United States 2020 | Unclear ethnic breakdown |
| Reznik 2014 Insulin pump treatment compared with multiple daily injections for treatment of type 2 diabetes (OpT2mise): a randomised open-label controlled trial. Reznik, Yves; Cohen, Ohad; Aronson, Ronnie; Conget, Ignacio; Runzis, Sarah; Castaneda, Javier; Lee, Scott W; OpT2mise Study Group Lancet (London, England) / 2014;384(9950):1265-72<br>England 2014 | Unclear ethnic breakdown |
| **Not all participants had T2DM** | **17** |
| leRoux 2017 3 years of liraglutide versus placebo for type 2 diabetes risk reduction and weight management in individuals with prediabetes: a randomised, double-blind trial. le Roux, Carel W; Astrup, Arne; Fujioka, Ken; Greenway, Frank; Lau, David C W; Van Gaal, Luc; Ortiz, Rafael Violante; Wilding, John P H; Skjoth, Trine V; Manning, Linda Shapiro; Pi-Sunyer, Xavier; SCALE Obesity Prediabetes NN8022-1839 Study Group Lancet (London, England) / 2017;389(10077):1399-1409 England 2017 | Not all participants had T2DM |
| NAVIGATORStudyGroup 2010 Effect of valsartan on the incidence of diabetes and cardiovascular events. NAVIGATOR Study Group; McMurray, John J; Holman, Rury R; Haffner, Steven M; Bethel, M Angelyn; Holzhauer, Bjorn; Hua, Tsushung A; Belenkov, Yuri; Boolell, Mitradev; Buse, John B; Buckley, Brendan M; Chacra, Antonio R; Chiang, Fu-Tien; Charbonnel, Bernard; Chow, Chun-Chung; Davies, Melanie J; Deedwania, Prakash; Diem, Peter; Einhorn, Daniel; Fonseca, Vivian; Fulcher, Gregory R; Gaciong, Zbigniew; Gaztambide, Sonia; Giles, Thomas; Horton, Edward; Ilkova, Hasan; Jenssen, Trond; Kahn, Steven E; Krum, Henry; Laakso, Markku; Leiter, Lawrence A; Levitt, Naomi S; Mareev, Viacheslav; Martinez, Felipe; Masson, Chantal; Mazzone, Theodore; Meaney, Eduardo; Nesto, Richard; Pan, Changyu; Prager, Rudolf; Raptis, Sotirios A; Rutten, Guy E H M; Sandstroem, Herbert; Schaper, Frank; Scheen, Andre; Schmitz, Ole; Sinay, Isaac; Soska, Vladimir; Stender, Steen; Tamas, Gyula; Tognoni, Gianni; Tuomilehto, Jaako; Villamil, Alberto S; Vozar, Juraj; Califf, Robert M The New England journal of medicine / 2010;362(16):1477-90 | Not all participants had T2DM |
| Chiasson 2002 Acarbose for prevention of type 2 diabetes mellitus: the STOP-NIDDM randomised trial. Chiasson, Jean-Louis; Josse, Robert G; Gomis, Ramon; Hanefeld, Markolf; Karasik, Avraham; Laakso, Markku; STOP-NIDDM Trail Research Group Lancet (London, England) / 2002;359(9323):2072-7 England 2002 / | Not all participants had T2DM |
| Petrak 2015 Cognitive Behavioral Therapy Versus Sertraline in Patients With Depression and Poorly Controlled Diabetes: The Diabetes and Depression (DAD) Study: A Randomized Controlled Multicenter Trial. Petrak, Frank; Herpertz, Stephan; Albus, Christian; Hermanns, Norbert; Hiemke, Christoph; Hiller, Wolfgang; Kronfeld, Kai; Kruse, Johannes; Kulzer, Bernd; Ruckes, Christian; Zahn, Daniela; Muller, Matthias J Diabetes care / 2015;38(5):767-75 | Not all participants had T2DM |
| Bohula 2018 Effect of lorcaserin on prevention and remission of type 2 diabetes in overweight and obese patients (CAMELLIA-TIMI 61): a randomised, placebo-controlled trial. Bohula, Erin A; Scirica, Benjamin M; Inzucchi, Silvio E; McGuire, Darren K; Keech, Anthony C; Smith, Steven R; Kanevsky, Estella; Murphy, Sabina A; Leiter, Lawrence A; Dwyer, Jamie P; Corbalan, Ramon; Hamm, Christian; Kaplan, Lee; Nicolau, Jose Carlos; Ophuis, Ton Oude; Ray, Kausik K; Ruda, Mikhail; Spinar, Jindrich; Patel, Tushar; Miao, Wenfeng; Perdomo, Carlos; Francis, Bruce; Dhadda, Shobha; Bonaca, Marc P; Ruff, Christian T; Sabatine, Marc S; Wiviott, Stephen D; CAMELLIA-TIMI 61 Steering Committee Investigators Lancet (London, England) / 2018;392(10161):2269-2279 England 2018 | Not all participants had T2DM |
| Chiasson 2003 Acarbose treatment and the risk of cardiovascular disease and hypertension in patients with impaired glucose tolerance: the STOP-NIDDM trial. Chiasson, Jean-Louis; Josse, Robert G; Gomis, Ramon; Hanefeld, Markolf; Karasik, Avraham; Laakso, Markku; STOP-NIDDM Trial Research Group JAMA / 2003;290(4):486-94 | Not all participants had T2DM |

| | |
|---|---|
| ORIGINTrialInvestigators 2012 Basal insulin and cardiovascular and other outcomes in dysglycemia. ORIGIN Trial Investigators; Gerstein, Hertzel C; Bosch, Jackie; Dagenais, Gilles R; Diaz, Rafael; Jung, Hyejung; Maggioni, Aldo P; Pogue, Janice; Probstfield, Jeffrey; Ramachandran, Ambady; Riddle, Matthew C; Ryden, Lars E; Yusuf, Salim The New England journal of medicine / 2012;367(4):319-28 | Not all participants had T2DM |
| Packer 2020 Cardiovascular and Renal Outcomes with Empagliflozin in Heart Failure. Packer, Milton; Anker, Stefan D; Butler, Javed; Filippatos, Gerasimos; Pocock, Stuart J; Carson, Peter; Januzzi, James; Verma, Subodh; Tsutsui, Hiroyuki; Brueckmann, Martina; Jamal, Waheed; Kimura, Karen; Schnee, Janet; Zeller, Cordula; Cotton, Daniel; Bocchi, Edimar; Bohm, Michael; Choi, Dong-Ju; Chopra, Vijay; Chuquiure, Eduardo; Giannetti, Nadia; Janssens, Stefan; Zhang, Jian; Gonzalez Juanatey, Jose R; Kaul, Sanjay; Brunner-La Rocca, Hans-Peter; Merkely, Bela; Nicholls, Stephen J; Perrone, Sergio; Pina, Ileana; Ponikowski, Piotr; Sattar, Naveed; Senni, Michele; Seronde, Marie-France; Spinar, Jindrich; Squire, Iain; Taddei, Stefano; Wanner, Christoph; Zannad, Faiez; EMPEROR-Reduced Trial Investigators The New England journal of medicine / 2020;383(15):1413-1424 | Not all participants had T2DM |
| DiabetesPreventionProgramResearchGroup 2012 Long-term safety, tolerability, and weight loss associated with metformin in the Diabetes Prevention Program Outcomes Study. Diabetes Prevention Program Research Group Diabetes care / 2012;35(4):731-7 | Not all participants had T2DM |
| Keech 2003 Secondary prevention of cardiovascular events with long-term pravastatin in patients with diabetes or impaired fasting glucose: results from the LIPID trial. Keech, Anthony; Colquhoun, David; Best, James; Kirby, Adrienne; Simes, R John; Hunt, David; Hague, Wendy; Beller, Elaine; Arulchelvam, Manjula; Baker, Jennifer; Tonkin, Andrew; LIPID Study Group Diabetes care / 2003;26(10):2713-21 Ref ID: 14514569 | Not all participants had T2DM |
| Heerspink 2020 Dapagliflozin in Patients with Chronic Kidney Disease. Heerspink, Hiddo J L; Stefansson, Bergur V; Correa-Rotter, Ricardo; Chertow, Glenn M; Greene, Tom; Hou, Fan-Fan; Mann, Johannes F E; McMurray, John J V; Lindberg, Magnus; Rossing, Peter; Sjostrom, C David; Toto, Roberto D; Langkilde, Anna-Maria; Wheeler, David C; DAPA-CKD Trial Committees and Investigators The New England journal of medicine / 2020;383(15):1436-1446 | Not all participants had T2DM |
| Apolzan 2019 Long-Term Weight Loss With Metformin or Lifestyle Intervention in the Diabetes Prevention Program Outcomes Study. Apolzan, John W; Venditti, Elizabeth M; Edelstein, Sharon L; Knowler, William C; Dabelea, Dana; Boyko, Edward J; Pi-Sunyer, Xavier; Kalyani, Rita R; Franks, Paul W; Srikanthan, Preethi; Gadde, Kishore M; Diabetes Prevention Program Research Group Annals of internal medicine / 2019;170(10):682-690 | Not all participants had T2DM |
| Bril 2009 Ranirestat for the management of diabetic sensorimotor polyneuropathy. Bril, Vera; Hirose, Toshiyuki; Tomioka, Sasagu; Buchanan, Robert; Ranirestat Study Group Diabetes care / 2009;32(7):1256-60 | Not all participants had T2DM |
| Bril 2004 Aldose reductase inhibition by AS-3201 in sural nerve from patients with diabetic sensorimotor polyneuropathy. Bril, Vera; Buchanan, Robert A Diabetes care / 2004;27(10):2369-75 | Not all participants had T2DM |
| ORIGINTrialInvestigators 2012 n-3 fatty acids and cardiovascular outcomes in patients with dysglycemia. ORIGIN Trial Investigators; Bosch, Jackie; Gerstein, Hertzel C; Dagenais, Gilles R; Diaz, Rafael; Dyal, Leanne; Jung, Hyejung; Maggiono, Aldo P; Probstfield, Jeffrey; Ramachandran, Ambady; Riddle, Matthew C; Ryden, Lars E; Yusuf, Salim The New England journal of medicine / 2012;367(4):309-18 | Not all participants had T2DM |
| Kirkman 2006 Treating postprandial hyperglycemia does not appear to delay progression of early type 2 diabetes: the Early Diabetes Intervention Program. Kirkman, M Sue; Shankar, R Ravi; Shankar, Sudha; Shen, Changyu; Brizendine, Edward; Baron, Alain; McGill, Janet Diabetes care / 2006;29(9):2095-101 | Not all participants had T2DM |
| **Not an RCT** | **7** |
| Rosenstock 2006 Triple therapy in type 2 diabetes: insulin glargine or rosiglitazone added to combination therapy of sulfonylurea plus metformin in insulin-naive patients. Rosenstock, Julio; Sugimoto, Danny; Strange, Poul; Stewart, John A; Soltes-Rak, Erika; Dailey, George Diabetes care / 2006;29(3):554-9 | Not an RCT |
| Xie 2020 Comparative Effectiveness of SGLT2 Inhibitors, GLP-1 Receptor Agonists, DPP-4 Inhibitors, and Sulfonylureas on Risk of Kidney Outcomes: Emulation of a Target Trial Using | Not an RCT |

| | |
|---|---|
| Health Care Databases. Xie, Yan; Bowe, Benjamin; Gibson, Andrew K; McGill, Janet B; Maddukuri, Geetha; Yan, Yan; Al-Aly, Ziyad Diabetes care / 2020;43(11):2859-2869 | |
| Nissen 2005 Effect of muraglitazar on death and major adverse cardiovascular events in patients with type 2 diabetes mellitus. Nissen, Steven E; Wolski, Kathy; Topol, Eric J JAMA / 2005;294(20):2581-6 | Not an RCT |
| Ceriello 2005 Comparison of effect of pioglitazone with metformin or sulfonylurea (monotherapy and combination therapy) on postload glycemia and composite insulin sensitivity index during an oral glucose tolerance test in patients with type 2 diabetes. Ceriello, Antonio; Johns, Don; Widel, Mario; Eckland, David J; Gilmore, Kathryn J; Tan, Meng H Diabetes care / 2005;28(2):266-72 | Not an RCT |
| Hugenschmidt 2014 The cross-sectional and longitudinal associations of diabetic retinopathy with cognitive function and brain MRI findings: the Action to Control Cardiovascular Risk in Diabetes (ACCORD) trial. Hugenschmidt, Christina E; Lovato, James F; Ambrosius, Walter T; Bryan, R Nick; Gerstein, Hertzel C; Horowitz, Karen R; Launer, Lenore J; Lazar, Ronald M; Murray, Anne M; Chew, Emily Y; Danis, Ronald P; Williamson, Jeff D; Miller, Michael E; Ding, Jingzhong Diabetes care / 2014;37(12):3244-52 | Not an RCT |
| Matsagoura 2003 Carotid intima-media thickness in patients with type 2 diabetes: the significance of microalbuminuria and different risk factors for atherosclerosis. Matsagoura, Maria; Andreadis, Emanouil; Diamantopoulos, Emanouil J; Vassilopoulos, Charalambos; Tentolouris, Nicholas; Katsilambros, Nicholas Diabetes care / 2003;26(10):2966 | Not an RCT |
| Rena 2018 Repurposing Metformin for Cardiovascular Disease. Rena, Graham; Lang, Chim C Circulation / 2018;137(5):422-424 | Not an RCT |
| **Not government nor industry funded** | **8** |
| Vaccaro 2012 The TOSCA.IT trial: a study designed to evaluate the effect of pioglitazone versus sulfonylureas on cardiovascular disease in type 2 diabetes. Vaccaro, Olga; Masulli, Maria; Bonora, Enzo; Del Prato, Stefano; Nicolucci, Antonio; Rivellese, Angela A; Riccardi, Gabriele; TOSCA.IT Study Group Diabetes care / 2012;35(12):e82 | Not government nor industry funded |
| Bluher 2003 Analysis of the relationship between the Pro12Ala variant in the PPAR-gamma2 gene and the response rate to therapy with pioglitazone in patients with type 2 diabetes. Bluher, Matthias; Lubben, Georg; Paschke, Ralf Diabetes care / 2003;26(3):825-31 | Not government nor industry funded |
| Weng 2008 Effect of intensive insulin therapy on beta-cell function and glycaemic control in patients with newly diagnosed type 2 diabetes: a multicentre randomised parallel-group trial. Weng, Jianping; Li, Yanbing; Xu, Wen; Shi, Lixin; Zhang, Qiao; Zhu, Dalong; Hu, Yun; Zhou, Zhiguang; Yan, Xiang; Tian, Haoming; Ran, Xingwu; Luo, Zuojie; Xian, Jing; Yan, Li; Li, Fangping; Zeng, Longyi; Chen, Yanming; Yang, Liyong; Yan, Sunjie; Liu, Juan; Li, Ming; Fu, Zuzhi; Cheng, Hua Lancet (London, England) / 2008;371(9626):1753-60 England 2008 / | Not government nor industry funded |
| Shichiri 2000 Long-term results of the Kumamoto Study on optimal diabetes control in type 2 diabetic patients. Shichiri, M; Kishikawa, H; Ohkubo, Y; Wake, N Diabetes care / 2000;23 Suppl 2(eag, 7805975):B21-9 | Not government nor industry funded |
| Pruski 2009 Pleiotropic action of short-term metformin and fenofibrate treatment, combined with lifestyle intervention, in type 2 diabetic patients with mixed dyslipidemia. Pruski, Maciej; Krysiak, Robert; Okopien, Boguslaw Diabetes care / 2009;32(8):1421-4 1 | Not government nor industry funded |
| Sasso 2002 Irbesartan reduces the albumin excretion rate in microalbuminuric type 2 diabetic patients independently of hypertension: a randomized double-blind placebo-controlled crossover study. Sasso, Ferdinando C; Carbonara, Ornella; Persico, Marcello; Iafusco, Dario; Salvatore, Teresa; D'Ambrosio, Rosanna; Torella, Roberto; Cozzolino, Domenico Diabetes care / 2002;25(11):1909-13 | Not government nor industry funded |
| Khan 2002 A prospective, randomized comparison of the metabolic effects of pioglitazone or rosiglitazone in patients with type 2 diabetes who were previously treated with troglitazone. Khan, Mehmood A; St Peter, John V; Xue, Jay L Diabetes care / 2002;25(4):708-11 | Not government nor industry funded |

| | |
|---|---|
| **Protocol paper** | **3** |
| Nathan 2013 Rationale and design of the glycemia reduction approaches in diabetes: a comparative effectiveness study (GRADE). Nathan, David M; Buse, John B; Kahn, Steven E; Krause-Steinrauf, Heidi; Larkin, Mary E; Staten, Myrlene; Wexler, Deborah; Lachin, John M; GRADE Study Research Group Diabetes care / 2013;36(8):2254-61 1 | Protocol paper |
| FLAT-SUGARTrialInvestigators 2015 Design of FLAT-SUGAR: Randomized Trial of Prandial Insulin Versus Prandial GLP-1 Receptor Agonist Together With Basal Insulin and Metformin for High-Risk Type 2 Diabetes. FLAT-SUGAR Trial Investigators; Probstfield, Jeffrey L; Hirsch, Irl; O'Brien, Kevin; Davis, Barry; Bergenstal, Richard; Kingry, Connie; Khakpour, Dori; Pressel, Sarah; Branch, Kelley R; Riddle, Matthew Diabetes care / 2015;38(8):1558-66 | Protocol paper |
| Viberti 2002 A diabetes outcome progression trial (ADOPT): an international multicenter study of the comparative efficacy of rosiglitazone, glyburide, and metformin in recently diagnosed type 2 diabetes. Viberti, Giancarlo; Kahn, Steven E; Greene, Douglas A; Herman, William H; Zinman, Bernard; Holman, Rury R; Haffner, Steven M; Levy, Daniel; Lachin, John M; Berry, Rhona A; Heise, Mark A; Jones, Nigel P; Freed, Martin I Diabetes care / 2002;25(10):1737-43 | Protocol paper |
| **Substudy or follow-up study** | **91** |
| Bril 2006 Long-term effects of ranirestat (AS-3201) on peripheral nerve function in patients with diabetic sensorimotor polyneuropathy. Bril, Vera; Buchanan, Robert A Diabetes care / 2006;29(1):68-72 | Substudy or follow-up study |
| Miller 2014 Effects of randomization to intensive glucose control on adverse events, cardiovascular disease, and mortality in older versus younger adults in the ACCORD Trial. Miller, Michael E; Williamson, Jeff D; Gerstein, Hertzel C; Byington, Robert P; Cushman, William C; Ginsberg, Henry N; Ambrosius, Walter T; Lovato, Laura; Applegate, William B; ACCORD Investigators Diabetes care / 2014;37(3):634-43 1 | Substudy or follow-up study |
| White 2016 Cardiovascular Mortality in Patients With Type 2 Diabetes and Recent Acute Coronary Syndromes From the EXAMINE Trial. White, William B; Kupfer, Stuart; Zannad, Faiez; Mehta, Cyrus R; Wilson, Craig A; Lei, Lanyu; Bakris, George L; Nissen, Steven E; Cushman, William C; Heller, Simon R; Bergenstal, Richard M; Fleck, Penny R; Cannon, Christopher P; EXAMINE Investigators Diabetes care / 2016;39(7):1267-73 | Substudy or follow-up study |
| Solomon 2010 Erythropoietic response and outcomes in kidney disease and type 2 diabetes. Solomon, Scott D; Uno, Hajime; Lewis, Eldrin F; Eckardt, Kai-Uwe; Lin, Julie; Burdmann, Emmanuel A; de Zeeuw, Dick; Ivanovich, Peter; Levey, Andrew S; Parfrey, Patrick; Remuzzi, Giuseppe; Singh, Ajay K; Toto, Robert; Huang, Fannie; Rossert, Jerome; McMurray, John J V; Pfeffer, Marc A; Trial to Reduce Cardiovascular Events with Aranesp Therapy (TREAT) Investigators The New England journal of medicine / 2010;363(12):1146-55 | Substudy or follow-up study |
| Neil 2006 Analysis of efficacy and safety in patients aged 65-75 years at randomization: Collaborative Atorvastatin Diabetes Study (CARDS). Neil, H Andrew W; DeMicco, David A; Luo, Don; Betteridge, D John; Colhoun, Helen M; Durrington, Paul N; Livingstone, Shona J; Fuller, John H; Hitman, Graham A; CARDS Study Investigators Diabetes care / 2006;29(11):2378-84 | Substudy or follow-up study |
| Raz 2011 Post hoc subgroup analysis of the HEART2D trial demonstrates lower cardiovascular risk in older patients targeting postprandial versus fasting/premeal glycemia. Raz, Itamar; Ceriello, Antonio; Wilson, Peter W; Battioui, Chakib; Su, Eric W; Kerr, Lisa; Jones, Cate A; Milicevic, Zvonko; Jacober, Scott J Diabetes care / 2011;34(7):1511-3 | Substudy or follow-up study |
| ORIGINTrialInvestigators 2015 Predictors of nonsevere and severe hypoglycemia during glucose-lowering treatment with insulin glargine or standard drugs in the ORIGIN trial. ORIGIN Trial Investigators Diabetes care / 2015;38(1):22-8 | Substudy or follow-up study |
| Diamant 2012 Safety and efficacy of once-weekly exenatide compared with insulin glargine titrated to target in patients with type 2 diabetes over 84 weeks. Diamant, Michaela; Van Gaal, Luc; Stranks, Stephen; Guerci, Bruno; MacConell, Leigh; Haber, Harry; Scism-Bacon, Jamie; Trautmann, Michael Diabetes care / 2012;35(4):683-9 | Substudy or follow-up study |
| Woodward 2011 Does glycemic control offer similar benefits among patients with diabetes in different regions of the world? Results from the ADVANCE trial. Woodward, Mark; Patel, Anushka; Zoungas, Sophia; Liu, Lisheng; Pan, Changyu; Poulter, Neil; Januszewicz, Andrzej; | Substudy or follow-up study |

| | |
|---|---|
| Tandon, Nikhil; Joshi, Prashant; Heller, Simon; Neal, Bruce; Chalmers, John Diabetes care / 2011;34(12):2491-5 1 | |
| Tikkanen 2015 Empagliflozin reduces blood pressure in patients with type 2 diabetes and hypertension. Tikkanen, Ilkka; Narko, Kirsi; Zeller, Cordula; Green, Alexandra; Salsali, Afshin; Broedl, Uli C; Woerle, Hans J; EMPA-REG BP Investigators Diabetes care / 2015;38(3):420-8 | Substudy or follow-up study |
| Pop-Busui 2013 Impact of glycemic control strategies on the progression of diabetic peripheral neuropathy in the Bypass Angioplasty Revascularization Investigation 2 Diabetes (BARI 2D) Cohort. Pop-Busui, Rodica; Lu, Jiang; Brooks, Maria Mori; Albert, Stewart; Althouse, Andrew D; Escobedo, Jorge; Green, Jenifer; Palumbo, Pasquale; Perkins, Bruce A; Whitehouse, Fred; Jones, Teresa L Z; BARI 2D Study Group Diabetes care / 2013;36(10):3208-15 1 | Substudy or follow-up study |
| Hartman 2020 Effects of Novel Dual GIP and GLP-1 Receptor Agonist Tirzepatide on Biomarkers of Nonalcoholic Steatohepatitis in Patients With Type 2 Diabetes. Hartman, Mark L; Sanyal, Arun J; Loomba, Rohit; Wilson, Jonathan M; Nikooienejad, Amir; Bray, Ross; Karanikas, Chrisanthi A; Duffin, Kevin L; Robins, Deborah A; Haupt, Axel Diabetes care / 2020;43(6):1352-1355 | Substudy or follow-up study |
| Forsblom 2010 Effects of long-term fenofibrate treatment on markers of renal function in type 2 diabetes: the FIELD Helsinki substudy. Forsblom, Carol; Hiukka, Anne; Leinonen, Eeva S; Sundvall, Jouko; Groop, Per-Henrik; Taskinen, Marja-Riitta Diabetes care / 2010;33(2):215-20 | Substudy or follow-up study |
| Crowley 2013 Impact of baseline insulin regimen on glycemic response to a group medical clinic intervention. Crowley, Matthew J; Melnyk, Stephanie D; Coffman, Cynthia J; Jeffreys, Amy S; Edelman, David Diabetes care / 2013;36(7):1954-60 1 | Substudy or follow-up study |
| Buse 2017 Pancreatic Safety of Sitagliptin in the TECOS Study. Buse, John B; Bethel, M Angelyn; Green, Jennifer B; Stevens, Susanna R; Lokhnygina, Yuliya; Aschner, Pablo; Grado, Carlos Raffo; Tankova, Tsvetalina; Wainstein, Julio; Josse, Robert; Lachin, John M; Engel, Samuel S; Patel, Keyur; Peterson, Eric D; Holman, Rury R; TECOS Study Group Diabetes care / 2017;40(2):164-170 | Substudy or follow-up study |
| Ruggenenti 2012 Glomerular hyperfiltration and renal disease progression in type 2 diabetes. Ruggenenti, Piero; Porrini, Esteban L; Gaspari, Flavio; Motterlini, Nicola; Cannata, Antonio; Carrara, Fabiola; Cella, Claudia; Ferrari, Silvia; Stucchi, Nadia; Parvanova, Aneliya; Iliev, Ilian; Dodesini, Alessandro Roberto; Trevisan, Roberto; Bossi, Antonio; Zaletel, Jelka; Remuzzi, Giuseppe; GFR Study Investigators Diabetes care / 2012;35(10):2061-8 | Substudy or follow-up study |
| Neeland 2020 The Impact of Empagliflozin on Obstructive Sleep Apnea and Cardiovascular and Renal Outcomes: An Exploratory Analysis of the EMPA-REG OUTCOME Trial. Neeland, Ian J; Eliasson, Bjorn; Kasai, Takatoshi; Marx, Nikolaus; Zinman, Bernard; Inzucchi, Silvio E; Wanner, Christoph; Zwiener, Isabella; Wojeck, Brian S; Yaggi, Henry K; Johansen, Odd Erik; EMPA-REG OUTCOME Investigators Diabetes care / 2020;43(12):3007-3015 | Substudy or follow-up study |
| Linz 2014 Paradoxical reduction in HDL-C with fenofibrate and thiazolidinedione therapy in type 2 diabetes: the ACCORD Lipid Trial. Linz, Peter E; Lovato, Laura C; Byington, Robert P; O'Connor, Patrick J; Leiter, Lawrence A; Weiss, Daniel; Force, Rex W; Crouse, John R; Ismail-Beigi, Faramarz; Simmons, Debra L; Papademetriou, Vasilios; Ginsberg, Henry N; Elam, Marshall B Diabetes care / 2014;37(3):686-93 | Substudy or follow-up study |
| Fitchett 2019 Empagliflozin Reduced Mortality and Hospitalization for Heart Failure Across the Spectrum of Cardiovascular Risk in the EMPA-REG OUTCOME Trial. Fitchett, David; Inzucchi, Silvio E; Cannon, Christopher P; McGuire, Darren K; Scirica, Benjamin M; Johansen, Odd Erik; Sambevski, Steven; Kaspers, Stefan; Pfarr, Egon; George, Jyothis T; Zinman, Bernard Circulation / 2019;139(11):1384-1395 | Substudy or follow-up study |
| Fudim 2019 Effect of Once-Weekly Exenatide in Patients With Type 2 Diabetes Mellitus With and Without Heart Failure and Heart Failure-Related Outcomes: Insights From the EXSCEL Trial. Fudim, Marat; White, Jennifer; Pagidipati, Neha J; Lokhnygina, Yuliya; Wainstein, Julio; Murin, Jan; Iqbal, Nayyar; Ohman, Peter; Lopes, Renato D; Reicher, Barry; Holman, Rury R; Hernandez, Adrian F; Mentz, Robert J Circulation / 2019;140(20):1613-1622 | Substudy or follow-up study |
| Zinman 2009 Efficacy and safety of the human glucagon-like peptide-1 analog liraglutide in combination with metformin and thiazolidinedione in patients with type 2 diabetes (LEAD-4 Met+TZD). Zinman, Bernard; Gerich, John; Buse, John B; Lewin, Andrew; Schwartz, Sherwyn; | Substudy or follow-up study |

| | |
|---|---|
| Raskin, Philip; Hale, Paula M; Zdravkovic, Milan; Blonde, Lawrence; LEAD-4 Study Investigators Diabetes care / 2009;32(7):1224-30 | |
| ActiontoControlCardiovascularRiskinDiabetesFollow-On(ACCORDION)EyeStudyGroupandtheActiontoControlCardiovascularRiskinDiabetesFollow-On(ACCORDION)StudyGroup 2016 Persistent Effects of Intensive Glycemic Control on Retinopathy in Type 2 Diabetes in the Action to Control Cardiovascular Risk in Diabetes (ACCORD) Follow-On Study. Action to Control Cardiovascular Risk in Diabetes Follow-On (ACCORDION) Eye Study Group and the Action to Control Cardiovascular Risk in Diabetes Follow-On (ACCORDION) Study Group Diabetes care / 2016;39(7):1089-100 2 | Substudy or follow-up study |
| Cosentino 2020 Efficacy of Ertugliflozin on Heart Failure-Related Events in Patients With Type 2 Diabetes Mellitus and Established Atherosclerotic Cardiovascular Disease: Results of the VERTIS CV Trial. Cosentino, Francesco; Cannon, Christopher P; Cherney, David Z I; Masiukiewicz, Urszula; Pratley, Richard; Dagogo-Jack, Sam; Frederich, Robert; Charbonnel, Bernard; Mancuso, James; Shih, Weichung J; Terra, Steven G; Cater, Nilo B; Gantz, Ira; McGuire, Darren K; VERTIS CV Investigators Circulation / 2020;142(23):2205-2215 | Substudy or follow-up study |
| Vakkilainen 2003 Relationships between low-density lipoprotein particle size, plasma lipoproteins, and progression of coronary artery disease: the Diabetes Atherosclerosis Intervention Study (DAIS). Vakkilainen, Juha; Steiner, George; Ansquer, Jean-Claude; Aubin, Francois; Rattier, Stephanie; Foucher, Christelle; Hamsten, Anders; Taskinen, Marja-Riitta; DAIS Group Circulation / 2003;107(13):1733-7 | Substudy or follow-up study |
| Bethel 2017 Assessing the Safety of Sitagliptin in Older Participants in the Trial Evaluating Cardiovascular Outcomes with Sitagliptin (TECOS). Bethel, M Angelyn; Engel, Samuel S; Green, Jennifer B; Huang, Zhen; Josse, Robert G; Kaufman, Keith D; Standl, Eberhard; Suryawanshi, Shailaja; Van de Werf, Frans; McGuire, Darren K; Peterson, Eric D; Holman, Rury R; TECOS Study Group Diabetes care / 2017;40(4):494-501 | Substudy or follow-up study |
| Berk-Planken 2003 Atorvastatin dose-dependently decreases hepatic lipase activity in type 2 diabetes: effect of sex and the LIPC promoter variant. Berk-Planken, Ingrid I L; Hoogerbrugge, Nicoline; Stolk, Ronald P; Bootsma, Aart H; Jansen, Hans; DALI Study Group Diabetes care / 2003;26(2):427-32 | Substudy or follow-up study |
| Shepherd 2006 Effect of lowering LDL cholesterol substantially below currently recommended levels in patients with coronary heart disease and diabetes: the Treating to New Targets (TNT) study. Shepherd, James; Barter, Philip; Carmena, Rafael; Deedwania, Prakash; Fruchart, Jean-Charles; Haffner, Steven; Hsia, Judith; Breazna, Andrei; LaRosa, John; Grundy, Scott; Waters, David Diabetes care / 2006;29(6):1220-6 | Substudy or follow-up study |
| Vaur 2003 Development of congestive heart failure in type 2 diabetic patients with microalbuminuria or proteinuria: observations from the DIABHYCAR (type 2 DIABetes, Hypertension, CArdiovascular Events and Ramipril) study. Vaur, Laurent; Gueret, Pascal; Lievre, Michel; Chabaud, Sylvie; Passa, Philippe; DIABHYCAR Study Group (type 2 DIABetes, Hypertension, CARdiovascular Events and Ramipril) study Diabetes care / 2003;26(3):855-60 | Substudy or follow-up study |
| Navarro-Gonzalez 2018 Effects of Pentoxifylline on Soluble Klotho Concentrations and Renal Tubular Cell Expression in Diabetic Kidney Disease. Navarro-Gonzalez, Juan F; Sanchez-Nino, Maria Dolores; Donate-Correa, Javier; Martin-Nunez, Ernesto; Ferri, Carla; Perez-Delgado, Nayra; Gorriz, Jose Luis; Martinez-Castelao, Alberto; Ortiz, Alberto; Mora-Fernandez, Carmen Diabetes care / 2018;41(8):1817-1820 | Substudy or follow-up study |
| Leiter 2015 Efficacy and safety of saxagliptin in older participants in the SAVOR-TIMI 53 trial. Leiter, Lawrence A; Teoh, Hwee; Braunwald, Eugene; Mosenzon, Ofri; Cahn, Avivit; Kumar, K M Prasanna; Smahelova, Alena; Hirshberg, Boaz; Stahre, Christina; Frederich, Robert; Bonnici, Francois; Scirica, Benjamin M; Bhatt, Deepak L; Raz, Itamar; SAVOR-TIMI 53 Steering Committee and Investigators Diabetes care / 2015;38(6):1145-53 | Substudy or follow-up study |
| Hata 2013 Effects of visit-to-visit variability in systolic blood pressure on macrovascular and microvascular complications in patients with type 2 diabetes mellitus: the ADVANCE trial. Hata, Jun; Arima, Hisatomi; Rothwell, Peter M; Woodward, Mark; Zoungas, Sophia; Anderson, Craig; Patel, Anushka; Neal, Bruce; Glasziou, Paul; Hamet, Pavel; Mancia, Giuseppe; Poulter, Neil; Williams, Bryan; Macmahon, Stephen; Chalmers, John; ADVANCE Collaborative Group Circulation / 2013;128(12):1325-34 | Substudy or follow-up study |

| | |
|---|---|
| Wallander 2007 IGF binding protein 1 predicts cardiovascular morbidity and mortality in patients with acute myocardial infarction and type 2 diabetes. Wallander, Marit; Norhammar, Anna; Malmberg, Klas; Ohrvik, John; Ryden, Lars; Brismar, Kerstin Diabetes care / 2007;30(9):2343-8 | Substudy or follow-up study |
| Margolis 2014 Outcomes of combined cardiovascular risk factor management strategies in type 2 diabetes: the ACCORD randomized trial. Margolis, Karen L; O'Connor, Patrick J; Morgan, Timothy M; Buse, John B; Cohen, Robert M; Cushman, William C; Cutler, Jeffrey A; Evans, Gregory W; Gerstein, Hertzel C; Grimm, Richard H Jr; Lipkin, Edward W; Narayan, K M Venkat; Riddle, Matthew C Jr; Sood, Ajay; Goff, David C Jr Diabetes care / 2014;37(6):1721-8 1 | Substudy or follow-up study |
| ACCORDStudyGroup 2010 Effects of medical therapies on retinopathy progression in type 2 diabetes. ACCORD Study Group; ACCORD Eye Study Group; Chew, Emily Y; Ambrosius, Walter T; Davis, Matthew D; Danis, Ronald P; Gangaputra, Sapna; Greven, Craig M; Hubbard, Larry; Esser, Barbara A; Lovato, James F; Perdue, Letitia H; Goff, David C Jr; Cushman, William C; Ginsberg, Henry N; Elam, Marshall B; Genuth, Saul; Gerstein, Hertzel C; Schubart, Ulrich; Fine, Lawrence J The New England journal of medicine / 2010;363(3):233-44 | Substudy or follow-up study |
| Leiter 2015 Canagliflozin provides durable glycemic improvements and body weight reduction over 104 weeks versus glimepiride in patients with type 2 diabetes on metformin: a randomized, double-blind, phase 3 study. Leiter, Lawrence A; Yoon, Kun-Ho; Arias, Pablo; Langslet, Gisle; Xie, John; Balis, Dainius A; Millington, Dawn; Vercruysse, Frank; Canovatchel, William; Meininger, Gary Diabetes care / 2015;38(3):355-64 | Substudy or follow-up study |
| Zoungas 2009 Combined effects of routine blood pressure lowering and intensive glucose control on macrovascular and microvascular outcomes in patients with type 2 diabetes: New results from the ADVANCE trial. Zoungas, Sophia; de Galan, Bastiaan E; Ninomiya, Toshiharu; Grobbee, Diederick; Hamet, Pavel; Heller, Simon; MacMahon, Stephen; Marre, Michel; Neal, Bruce; Patel, Anushka; Woodward, Mark; Chalmers, John; ADVANCE Collaborative Group; Cass, Alan; Glasziou, Paul; Harrap, Stephen; Lisheng, Liu; Mancia, Guiseppe; Pillai, Avinesh; Poulter, Neil; Perkovic, Vlado; Travert, Florence Diabetes care / 2009;32(11):2068-74 | Substudy or follow-up study |
| Riddle 2010 Epidemiologic relationships between A1C and all-cause mortality during a median 3.4-year follow-up of glycemic treatment in the ACCORD trial. Riddle, Matthew C; Ambrosius, Walter T; Brillon, David J; Buse, John B; Byington, Robert P; Cohen, Robert M; Goff, David C Jr; Malozowski, Saul; Margolis, Karen L; Probstfield, Jeffrey L; Schnall, Adrian; Seaquist, Elizabeth R; Action to Control Cardiovascular Risk in Diabetes Investigators Diabetes care / 2010;33(5):983-90 | Substudy or follow-up study |
| Kahn 2010 Rosiglitazone decreases C-reactive protein to a greater extent relative to glyburide and metformin over 4 years despite greater weight gain: observations from a Diabetes Outcome Progression Trial (ADOPT). Kahn, Steven E; Haffner, Steven M; Viberti, Giancarlo; Herman, William H; Lachin, John M; Kravitz, Barbara G; Yu, Dahong; Paul, Gitanjali; Holman, Rury R; Zinman, Bernard; Diabetes Outcome Progression Trial (ADOPT) Study Group Diabetes care / 2010;33(1):177-83 | Substudy or follow-up study |
| Jabbour 2020 Efficacy and Safety Over 2 Years of Exenatide Plus Dapagliflozin in the DURATION-8 Study: A Multicenter, Double-Blind, Phase 3, Randomized Controlled Trial. Jabbour, Serge A; Frias, Juan P; Ahmed, Azazuddin; Hardy, Elise; Choi, Jasmine; Sjostrom, C David; Guja, Cristian Diabetes care / 2020;43(10):2528-2536 | Substudy or follow-up study |
| Perkovic 2020 Effects of Linagliptin on Cardiovascular and Kidney Outcomes in People With Normal and Reduced Kidney Function: Secondary Analysis of the CARMELINA Randomized Trial. Perkovic, Vlado; Toto, Robert; Cooper, Mark E; Mann, Johannes F E; Rosenstock, Julio; McGuire, Darren K; Kahn, Steven E; Marx, Nikolaus; Alexander, John H; Zinman, Bernard; Pfarr, Egon; Schnaidt, Sven; Meinicke, Thomas; von Eynatten, Maximillian; George, Jyothis T; Johansen, Odd Erik; Wanner, Christoph; CARMELINA investigators Diabetes care / 2020;43(8):1803-1812 | Substudy or follow-up study |
| Wong 2016 Long-term Benefits of Intensive Glucose Control for Preventing End-Stage Kidney Disease: ADVANCE-ON. Wong, Muh Geot; Perkovic, Vlado; Chalmers, John; Woodward, Mark; Li, Qiang; Cooper, Mark E; Hamet, Pavel; Harrap, Stephen; Heller, Simon; MacMahon, Stephen; Mancia, Giuseppe; Marre, Michel; Matthews, David; Neal, Bruce; Poulter, Neil; | Substudy or follow-up study |

| | |
|---|---|
| Rodgers, Anthony; Williams, Bryan; Zoungas, Sophia; ADVANCE-ON Collaborative Group Diabetes care / 2016;39(5):694-700 1 | |
| Ahren 2010 Changes in prandial glucagon levels after a 2-year treatment with vildagliptin or glimepiride in patients with type 2 diabetes inadequately controlled with metformin monotherapy. Ahren, Bo; Foley, James E; Ferrannini, Ele; Matthews, David R; Zinman, Bernard; Dejager, Sylvie; Fonseca, Vivian A Diabetes care / 2010;33(4):730-2 | Substudy or follow-up study |
| ACCORDStudyGroup 2010 Effects of combination lipid therapy in type 2 diabetes mellitus. ACCORD Study Group; Ginsberg, Henry N; Elam, Marshall B; Lovato, Laura C; Crouse, John R 3rd; Leiter, Lawrence A; Linz, Peter; Friedewald, William T; Buse, John B; Gerstein, Hertzel C; Probstfield, Jeffrey; Grimm, Richard H; Ismail-Beigi, Faramarz; Bigger, J Thomas; Goff, David C Jr; Cushman, William C; Simons-Morton, Denise G; Byington, Robert P The New England journal of medicine / 2010;362(17):1563-74 | Substudy or follow-up study |
| Pagidipati 2017 Secondary Prevention of Cardiovascular Disease in Patients With Type 2 Diabetes Mellitus: International Insights From the TECOS Trial (Trial Evaluating Cardiovascular Outcomes With Sitagliptin). Pagidipati, Neha J; Navar, Ann Marie; Pieper, Karen S; Green, Jennifer B; Bethel, M Angelyn; Armstrong, Paul W; Josse, Robert G; McGuire, Darren K; Lokhnygina, Yuliya; Cornel, Jan H; Halvorsen, Sigrun; Strandberg, Timo E; Delibasi, Tuncay; Holman, Rury R; Peterson, Eric D; TECOS Study Group Circulation / 2017;136(13):1193-1203 | Substudy or follow-up study |
| Bordeleau 2014 The association of basal insulin glargine and/or n-3 fatty acids with incident cancers in patients with dysglycemia. Bordeleau, Louise; Yakubovich, Natalia; Dagenais, Gilles R; Rosenstock, Julio; Probstfield, Jeffrey; Chang Yu, Pan; Ryden, Lars E; Pirags, Valdis; Spinas, Giatgen A; Birkeland, Kare I; Ratner, Robert E; Marin-Neto, Jose A; Keltai, Matyas; Riddle, Matthew C; Bosch, Jackie; Yusuf, Salim; Gerstein, Hertzel C; ORIGIN Trial Investigators Diabetes care / 2014;37(5):1360-6 | Substudy or follow-up study |
| Hegedus 2018 No Evidence of Increase in Calcitonin Concentrations or Development of C-Cell Malignancy in Response to Liraglutide for Up to 5 Years in the LEADER Trial. Hegedus, Laszlo; Sherman, Steven I; Tuttle, R Michael; von Scholten, Bernt J; Rasmussen, Soren; Karsbol, Julie D; Daniels, Gilbert H; LEADER Publication Committee on behalf of the LEADER Trial Investigators Diabetes care / 2018;41(3):620-622 | Substudy or follow-up study |
| Williams 2007 Efficacy of sertraline in prevention of depression recurrence in older versus younger adults with diabetes. Williams, Monique M; Clouse, Ray E; Nix, Billy D; Rubin, Eugene H; Sayuk, Gregory S; McGill, Janet B; Gelenberg, Alan J; Ciechanowski, Paul S; Hirsch, Irl B; Lustman, Patrick J Diabetes care / 2007;30(4):801-6 Ref ID: 17392541 | Substudy or follow-up study |
| Sobel 2011 Profibrinolytic, antithrombotic, and antiinflammatory effects of an insulin-sensitizing strategy in patients in the Bypass Angioplasty Revascularization Investigation 2 Diabetes (BARI 2D) trial. Sobel, Burton E; Hardison, Regina M; Genuth, Saul; Brooks, Maria M; McBane, Robert D 3rd; Schneider, David J; Pratley, Richard E; Huber, Kurt; Wolk, Robert; Krishnaswami, Ashok; Frye, Robert L; BARI 2D Investigators Circulation / 2011;124(6):695-703 | Substudy or follow-up study |
| Neuen 2018 Cardiovascular and Renal Outcomes With Canagliflozin According to Baseline Kidney Function. Neuen, Brendon L; Ohkuma, Toshiaki; Neal, Bruce; Matthews, David R; de Zeeuw, Dick; Mahaffey, Kenneth W; Fulcher, Greg; Desai, Mehul; Li, Qiang; Deng, Hsiaowei; Rosenthal, Norm; Jardine, Meg J; Bakris, George; Perkovic, Vlado Circulation / 2018;138(15):1537-1550 | Substudy or follow-up study |
| Cahn 2020 Efficacy and Safety of Dapagliflozin in the Elderly: Analysis From the DECLARE-TIMI 58 Study. Cahn, Avivit; Mosenzon, Ofri; Wiviott, Stephen D; Rozenberg, Aliza; Yanuv, Ilan; Goodrich, Erica L; Murphy, Sabina A; Bhatt, Deepak L; Leiter, Lawrence A; McGuire, Darren K; Wilding, John P H; Gause-Nilsson, Ingrid A M; Fredriksson, Martin; Johansson, Peter A; Langkilde, Anna Maria; Sabatine, Marc S; Raz, Itamar Diabetes care / 2020;43(2):468-475 | Substudy or follow-up study |
| Gilbert 2019 Effect of Liraglutide on Cardiovascular Outcomes in Elderly Patients: A Post Hoc Analysis of a Randomized Controlled Trial. Gilbert, Matthew P; Bain, Stephen C; Franek, Edward; Jodar-Gimeno, Esteban; Nauck, Michael A; Pratley, Richard; Rea, Rosangela Roginski; Kerr Saraiva, Jose Francisco; Rasmussen, Soren; Tornoe, Karen; von Scholten, Bernt Johan; Buse, John B; LEADER Publication Committee on behalf of the LEADER Trial Investigators Annals of internal medicine / 2019;170(6):423-426 | Substudy or follow-up study |

| | |
|---|---|
| Mann 2018 Effects of Liraglutide Versus Placebo on Cardiovascular Events in Patients With Type 2 Diabetes Mellitus and Chronic Kidney Disease. Mann, Johannes F E; Fonseca, Vivian; Mosenzon, Ofri; Raz, Itamar; Goldman, Bryan; Idorn, Thomas; von Scholten, Bernt Johan; Poulter, Neil R Circulation / 2018;138(25):2908-2918 | Substudy or follow-up study |
| vanVenrooij 2002 Aggressive lipid lowering does not improve endothelial function in type 2 diabetes: the Diabetes Atorvastatin Lipid Intervention (DALI) Study: a randomized, double-blind, placebo-controlled trial. van Venrooij, Francine V; van de Ree, Marcel A; Bots, Michiel L; Stolk, Ronald P; Huisman, Menno V; Banga, J D; DALI Study Group Diabetes care / 2002;25(7):1211-6 | Substudy or follow-up study |
| Barter 2011 Effect of torcetrapib on glucose, insulin, and hemoglobin A1c in subjects in the Investigation of Lipid Level Management to Understand its Impact in Atherosclerotic Events (ILLUMINATE) trial. Barter, Philip J; Rye, Kerry-Anne; Tardif, Jean-Claude; Waters, David D; Boekholdt, S Matthijs; Breazna, Andrei; Kastelein, John J P Circulation / 2011;124(5):555-62 | Substudy or follow-up study |
| Berl 2003 Cardiovascular outcomes in the Irbesartan Diabetic Nephropathy Trial of patients with type 2 diabetes and overt nephropathy. Berl, Tomas; Hunsicker, Lawrence G; Lewis, Julia B; Pfeffer, Marc A; Porush, Jerome G; Rouleau, Jean-Lucien; Drury, Paul L; Esmatjes, Enric; Hricik, Donald; Parikh, Chirag R; Raz, Itamar; Vanhille, Philippe; Wiegmann, Thomas B; Wolfe, Bernard M; Locatelli, Francesco; Goldhaber, Samuel Z; Lewis, Edmund J; Irbesartan Diabetic Nephropathy Trial. Collaborative Study Group Annals of internal medicine / 2003;138(7):542-9 | Substudy or follow-up study |
| Wanner 2018 Empagliflozin and Clinical Outcomes in Patients With Type 2 Diabetes Mellitus, Established Cardiovascular Disease, and Chronic Kidney Disease. Wanner, Christoph; Lachin, John M; Inzucchi, Silvio E; Fitchett, David; Mattheus, Michaela; George, Jyothis; Woerle, Hans J; Broedl, Uli C; von Eynatten, Maximilian; Zinman, Bernard; EMPA-REG OUTCOME Investigators Circulation / 2018;137(2):119-129 | Substudy or follow-up study |
| Basu 2018 Characteristics Associated With Decreased or Increased Mortality Risk From Glycemic Therapy Among Patients With Type 2 Diabetes and High Cardiovascular Risk: Machine Learning Analysis of the ACCORD Trial. Basu, Sanjay; Raghavan, Sridharan; Wexler, Deborah J; Berkowitz, Seth A Diabetes care / 2018;41(3):604-612 1 | Substudy or follow-up study |
| Udell 2015 Saxagliptin and cardiovascular outcomes in patients with type 2 diabetes and moderate or severe renal impairment: observations from the SAVOR-TIMI 53 Trial. Udell, Jacob A; Bhatt, Deepak L; Braunwald, Eugene; Cavender, Matthew A; Mosenzon, Ofri; Steg, Ph Gabriel; Davidson, Jaime A; Nicolau, Jose C; Corbalan, Ramon; Hirshberg, Boaz; Frederich, Robert; Im, KyungAh; Umez-Eronini, Amarachi A; He, Ping; McGuire, Darren K; Leiter, Lawrence A; Raz, Itamar; Scirica, Benjamin M; SAVOR-TIMI 53 Steering Committee and Investigators Diabetes care / 2015;38(4):696-705 | Substudy or follow-up study |
| Hawa 2014 LADA and CARDS: a prospective study of clinical outcome in established adult-onset autoimmune diabetes. Hawa, Mohammed Iqbal; Buchan, Ana Paula; Ola, Thomas; Wun, Chuan Chuan; DeMicco, David A; Bao, Weihang; Betteridge, D John; Durrington, Paul N; Fuller, John H; Neil, H Andrew W; Colhoun, Helen; Leslie, Richard David; Hitman, Graham A Diabetes care / 2014;37(6):1643-9 | Substudy or follow-up study |
| Waldman 2014 HDL-C and HDL-C/ApoA-I predict long-term progression of glycemia in established type 2 diabetes. Waldman, Boris; Jenkins, Alicia J; Davis, Timothy M E; Taskinen, Marja-Riitta; Scott, Russell; O'Connell, Rachel L; Gebski, Val J; Ng, Martin K C; Keech, Anthony C; FIELD Study Investigators Diabetes care / 2014;37(8):2351-8 | Substudy or follow-up study |
| vanVenrooij 2003 Common cholesteryl ester transfer protein gene polymorphisms and the effect of atorvastatin therapy in type 2 diabetes. van Venrooij, Francine V; Stolk, Ronald P; Banga, Jan-Dirk; Sijmonsma, Tjeerd P; van Tol, Arie; Erkelens, D Willem; Dallinga-Thie, Geesje M; DALI Study Group Diabetes care / 2003;26(4):1216-23 | Substudy or follow-up study |
| Raz 2014 Incidence of pancreatitis and pancreatic cancer in a randomized controlled multicenter trial (SAVOR-TIMI 53) of the dipeptidyl peptidase-4 inhibitor saxagliptin. Raz, Itamar; Bhatt, Deepak L; Hirshberg, Boaz; Mosenzon, Ofri; Scirica, Benjamin M; Umez-Eronini, Amarachi; Im, KyungAh; Stahre, Christina; Buskila, Alona; Iqbal, Nayyar; Greenberger, Norton; Lerch, Markus M Diabetes care / 2014;37(9):2435-41 | Substudy or follow-up study |

| | |
|---|---|
| Ghotbi 2013 Association of hypoglycemic treatment regimens with cardiovascular outcomes in overweight and obese subjects with type 2 diabetes: a substudy of the SCOUT trial. Ghotbi, Adam Ali; Kober, Lars; Finer, Nick; James, W Philip T; Sharma, Arya M; Caterson, Ian; Coutinho, Walmir; Van Gaal, Luc F; Torp-Pedersen, Christian; Andersson, Charlotte Diabetes care / 2013;36(11):3746-53 | Substudy or follow-up study |
| Mehler 2003 Intensive blood pressure control reduces the risk of cardiovascular events in patients with peripheral arterial disease and type 2 diabetes. Mehler, Philip S; Coll, Joseph R; Estacio, Raymond; Esler, Anne; Schrier, Robert W; Hiatt, William R Circulation / 2003;107(5):753-6 | Substudy or follow-up study |
| Gerstein 2019 Dulaglutide and renal outcomes in type 2 diabetes: an exploratory analysis of the REWIND randomised, placebo-controlled trial. Gerstein, Hertzel C; Colhoun, Helen M; Dagenais, Gilles R; Diaz, Rafael; Lakshmanan, Mark; Pais, Prem; Probstfield, Jeffrey; Botros, Fady T; Riddle, Matthew C; Ryden, Lars; Xavier, Denis; Atisso, Charles Messan; Dyal, Leanne; Hall, Stephanie; Rao-Melacini, Purnima; Wong, Gloria; Avezum, Alvaro; Basile, Jan; Chung, Namsik; Conget, Ignacio; Cushman, William C; Franek, Edward; Hancu, Nicolae; Hanefeld, Markolf; Holt, Shaun; Jansky, Petr; Keltai, Matyas; Lanas, Fernando; Leiter, Lawrence A; Lopez-Jaramillo, Patricio; Cardona Munoz, Ernesto German; Pirags, Valdis; Pogosova, Nana; Raubenheimer, Peter J; Shaw, Jonathan E; Sheu, Wayne H-H; Temelkova-Kurktschiev, Theodora; REWIND Investigators Lancet (London, England) / 2019;394(10193):131-138 England 2019 | Substudy or follow-up study |
| Figtree 2019 Effects of Canagliflozin on Heart Failure Outcomes Associated With Preserved and Reduced Ejection Fraction in Type 2 Diabetes Mellitus. Figtree, Gemma A; Radholm, Karin; Barrett, Terrance D; Perkovic, Vlado; Mahaffey, Kenneth W; de Zeeuw, Dick; Fulcher, Greg; Matthews, David R; Shaw, Wayne; Neal, Bruce Circulation / 2019;139(22):2591-2593 | Substudy or follow-up study |
| Mahaffey 2019 Canagliflozin and Cardiovascular and Renal Outcomes in Type 2 Diabetes Mellitus and Chronic Kidney Disease in Primary and Secondary Cardiovascular Prevention Groups. Mahaffey, Kenneth W; Jardine, Meg J; Bompoint, Severine; Cannon, Christopher P; Neal, Bruce; Heerspink, Hiddo J L; Charytan, David M; Edwards, Robert; Agarwal, Rajiv; Bakris, George; Bull, Scott; Capuano, George; de Zeeuw, Dick; Greene, Tom; Levin, Adeera; Pollock, Carol; Sun, Tao; Wheeler, David C; Yavin, Yshai; Zhang, Hong; Zinman, Bernard; Rosenthal, Norman; Brenner, Barry M; Perkovic, Vlado Circulation / 2019;140(9):739-750 | Substudy or follow-up study |
| Inzucchi 2018 How Does Empagliflozin Reduce Cardiovascular Mortality? Insights From a Mediation Analysis of the EMPA-REG OUTCOME Trial. Inzucchi, Silvio E; Zinman, Bernard; Fitchett, David; Wanner, Christoph; Ferrannini, Ele; Schumacher, Martin; Schmoor, Claudia; Ohneberg, Kristin; Johansen, Odd Erik; George, Jyothis T; Hantel, Stefan; Bluhmki, Erich; Lachin, John M Diabetes care / 2018;41(2):356-363 | Substudy or follow-up study |
| Winkelmayer 2006 Efficacy and safety of angiotensin II receptor blockade in elderly patients with diabetes. Winkelmayer, Wolfgang C; Zhang, Zhongxin; Shahinfar, Shahnaz; Cooper, Mark E; Avorn, Jerry; Brenner, Barry M Diabetes care / 2006;29(10):2210-7 | Substudy or follow-up study |
| Andersen 2003 Kidney function during and after withdrawal of long-term irbesartan treatment in patients with type 2 diabetes and microalbuminuria. Andersen, Steen; Brochner-Mortensen, Jens; Parving, Hans-Henrik; Irbesartan in Patients With Type 2 Diabetes and Microalbuminuria Study Group Diabetes care / 2003;26(12):3296-302 | Substudy or follow-up study |
| Kahn 2008 Rosiglitazone-associated fractures in type 2 diabetes: an Analysis from A Diabetes Outcome Progression Trial (ADOPT). Kahn, Steven E; Zinman, Bernard; Lachin, John M; Haffner, Steven M; Herman, William H; Holman, Rury R; Kravitz, Barbara G; Yu, Dahong; Heise, Mark A; Aftring, R Paul; Viberti, Giancarlo; Diabetes Outcome Progression Trial (ADOPT) Study Group Diabetes care / 2008;31(5):845-51 | Substudy or follow-up study |
| Garvey 2014 Weight-loss therapy in type 2 diabetes: effects of phentermine and topiramate extended release. Garvey, W Timothy; Ryan, Donna H; Bohannon, Nancy J V; Kushner, Robert F; Rueger, Miriam; Dvorak, Roman V; Troupin, Barbara Diabetes care / 2014;37(12):3309-16 | Substudy or follow-up study |
| Dallinga-Thie 2004 Atorvastatin decreases apolipoprotein C-III in apolipoprotein B-containing lipoprotein and HDL in type 2 diabetes: a potential mechanism to lower plasma triglycerides. Dallinga-Thie, Geesje M; Berk-Planken, Ingrid I L; Bootsma, Aart H; Jansen, Hans; Diabetes Atorvastatin Lipid intervention (DALI) Study Group Diabetes care / 2004;27(6):1358-64 | Substudy or follow-up study |

| | |
|---|---|
| Nauck 2019 Effects of Liraglutide Compared With Placebo on Events of Acute Gallbladder or Biliary Disease in Patients With Type 2 Diabetes at High Risk for Cardiovascular Events in the LEADER Randomized Trial. Nauck, Michael A; Muus Ghorbani, Marie Louise; Kreiner, Eskil; Saevereid, Hans A; Buse, John B; LEADER Publication Committee on behalf of the LEADER Trial Investigators Diabetes care / 2019;42(10):1912-1920 | Substudy or follow-up study |
| Persson 2010 Impact of baseline renal function on the efficacy and safety of aliskiren added to losartan in patients with type 2 diabetes and nephropathy. Persson, Frederik; Lewis, Julia B; Lewis, Edmund J; Rossing, Peter; Hollenberg, Norman K; Parving, Hans-Henrik; AVOID Study Investigators Diabetes care / 2010;33(11):2304-9 | Substudy or follow-up study |
| Buse 2010 Switching to once-daily liraglutide from twice-daily exenatide further improves glycemic control in patients with type 2 diabetes using oral agents. Buse, John B; Sesti, Giorgio; Schmidt, Wolfgang E; Montanya, Eduard; Chang, Cheng-Tao; Xu, Yizhen; Blonde, Lawrence; Rosenstock, Julio; Liraglutide Effect Action in Diabetes-6 Study Group Diabetes care / 2010;33(6):1300-3 1 | Substudy or follow-up study |
| Ilkun 2020 The Influence of Baseline Diastolic Blood Pressure on the Effects of Intensive Blood Pressure Lowering on Cardiovascular Outcomes and All-Cause Mortality in Type 2 Diabetes. Ilkun, Olesya L; Greene, Tom; Cheung, Alfred K; Whelton, Paul K; Wei, Guo; Boucher, Robert E; Ambrosius, Walter; Chertow, Glenn M; Beddhu, Srinivasan Diabetes care / 2020;43(8):1878-1884 2 | Substudy or follow-up study |
| Mosenzon 2015 Incidence of Fractures in Patients With Type 2 Diabetes in the SAVOR-TIMI 53 Trial. Mosenzon, Ofri; Wei, Cheryl; Davidson, Jaime; Scirica, Benjamin M; Yanuv, Ilan; Rozenberg, Aliza; Hirshberg, Boaz; Cahn, Avivit; Stahre, Christina; Strojek, Krzysztof; Bhatt, Deepak L; Raz, Itamar Diabetes care / 2015;38(11):2142-50 | Substudy or follow-up study |
| Cavender 2017 Serial Measurement of High-Sensitivity Troponin I and Cardiovascular Outcomes in Patients With Type 2 Diabetes Mellitus in the EXAMINE Trial (Examination of Cardiovascular Outcomes With Alogliptin Versus Standard of Care). Cavender, Matthew A; White, William B; Jarolim, Petr; Bakris, George L; Cushman, William C; Kupfer, Stuart; Gao, Qi; Mehta, Cyrus R; Zannad, Faiez; Cannon, Christopher P; Morrow, David A Circulation / 2017;135(20):1911-1921 | Substudy or follow-up study |
| Barzilay 2014 The impact of salsalate treatment on serum levels of advanced glycation end products in type 2 diabetes. Barzilay, Joshua I; Jablonski, Kathleen A; Fonseca, Vivian; Shoelson, Steven E; Goldfine, Allison B; Strauch, Christopher; Monnier, Vincent M; TINSAL-T2D Research Consortium Diabetes care / 2014;37(4):1083-91 | Substudy or follow-up study |
| Neal 2015 Efficacy and safety of canagliflozin, an inhibitor of sodium-glucose cotransporter 2, when used in conjunction with insulin therapy in patients with type 2 diabetes. Neal, Bruce; Perkovic, Vlado; de Zeeuw, Dick; Mahaffey, Kenneth W; Fulcher, Greg; Ways, Kirk; Desai, Mehul; Shaw, Wayne; Capuano, George; Alba, Maria; Jiang, Joel; Vercruysse, Frank; Meininger, Gary; Matthews, David; CANVAS Trial Collaborative Group Diabetes care / 2015;38(3):403-11 | Substudy or follow-up study |
| Nauck 2018 Neoplasms Reported With Liraglutide or Placebo in People With Type 2 Diabetes: Results From the LEADER Randomized Trial. Nauck, Michael A; Jensen, Thomas Jon; Rosenkilde, Carina; Calanna, Salvatore; Buse, John B; LEADER Publication Committee on behalf of the LEADER Trial Investigators Diabetes care / 2018;41(8):1663-1671 | Substudy or follow-up study |
| Koska 2013 The effect of intensive glucose lowering on lipoprotein particle profiles and inflammatory markers in the Veterans Affairs Diabetes Trial (VADT). Koska, Juraj; Saremi, Aramesh; Bahn, Gideon; Yamashita, Shizuya; Reaven, Peter D; Veterans Affairs Diabetes Trial Investigators Diabetes care / 2013;36(8):2408-14 2 | Substudy or follow-up study |
| Fonseca 2000 Long-term effects of troglitazone: open-label extension studies in type 2 diabetic patients. Fonseca, V; Foyt, H L; Shen, K; Whitcomb, R Diabetes care / 2000;23(3):354-9 | Substudy or follow-up study |
| Hanley 2010 Effect of Rosiglitazone and Ramipril on {beta}-cell function in people with impaired glucose tolerance or impaired fasting glucose: the DREAM trial. Hanley, Anthony J; Zinman, Bernard; Sheridan, Patrick; Yusuf, Salim; Gerstein, Hertzel C; Diabetes Reduction Assessment With Ramipril and Rosiglitazone Medication (DREAM) Investigators Diabetes care / 2010;33(3):608-13 | Substudy or follow-up study |

| | |
|---|---|
| Home 2007 Rosiglitazone evaluated for cardiovascular outcomes--an interim analysis. Home, Philip D; Pocock, Stuart J; Beck-Nielsen, Henning; Gomis, Ramon; Hanefeld, Markolf; Jones, Nigel P; Komajda, Michel; McMurray, John J V; RECORD Study Group The New England journal of medicine / 2007;357(1):28-38 | Substudy or follow-up study |
| Saremi 2016 A Link Between Hypoglycemia and Progression of Atherosclerosis in the Veterans Affairs Diabetes Trial (VADT). Saremi, Aramesh; Bahn, Gideon D; Reaven, Peter D; Veterans Affairs Diabetes Trial (VADT) Diabetes care / 2016;39(3):448-54 1 | Substudy or follow-up study |
| Verma 2018 Effects of Liraglutide on Cardiovascular Outcomes in Patients With Type 2 Diabetes Mellitus With or Without History of Myocardial Infarction or Stroke. Verma, Subodh; Poulter, Neil R; Bhatt, Deepak L; Bain, Stephen C; Buse, John B; Leiter, Lawrence A; Nauck, Michael A; Pratley, Richard E; Zinman, Bernard; Orsted, David D; Monk Fries, Tea; Rasmussen, Soren; Marso, Steven P Circulation / 2018;138(25):2884-2894 | Substudy or follow-up study |
| Zinman 2018 Hypoglycemia, Cardiovascular Outcomes, and Death: The LEADER Experience. Zinman, Bernard; Marso, Steven P; Christiansen, Erik; Calanna, Salvatore; Rasmussen, Soren; Buse, John B; LEADER Publication Committee on behalf of the LEADER Trial Investigators Diabetes care / 2018;41(8):1783-1791 | Substudy or follow-up study |
| Heller 2020 Redefining Hypoglycemia in Clinical Trials: Validation of Definitions Recently Adopted by the American Diabetes Association/European Association for the Study of Diabetes. Heller, Simon R; Buse, John B; Ratner, Robert; Seaquist, Elizabeth; Bardtrum, Lars; Hansen, Charlotte Thim; Tutkunkardas, Deniz; Moses, Alan C Diabetes care / 2020;43(2):398-404 | Substudy or follow-up study |
| Williamson 2014 Cognitive function and brain structure in persons with type 2 diabetes mellitus after intensive lowering of blood pressure and lipid levels: a randomized clinical trial. Williamson, Jeff D; Launer, Lenore J; Bryan, R Nick; Coker, Laura H; Lazar, Ronald M; Gerstein, Hertzel C; Murray, Anne M; Sullivan, Mark D; Horowitz, Karen R; Ding, Jingzhong; Marcovina, Santica; Lovato, Laura; Lovato, James; Margolis, Karen L; Davatzikos, Christos; Barzilay, Joshua; Ginsberg, Henry N; Linz, Peter E; Miller, Michael E JAMA internal medicine / 2014;174(3):324-33 | Substudy or follow-up study |
| **Wrong intervention** | **1** |
| Hill-Briggs 2005 Thirty-six-item short-form outcomes following a randomized controlled trial in type 2 diabetes. Hill-Briggs, Felicia; Gary, Tiffany L; Baptiste-Roberts, Kesha; Brancati, Frederick L Diabetes care / 2005;28(2):443-4 | Wrong intervention |

**ESM Fig. 1: PRISMA Flow Chart**

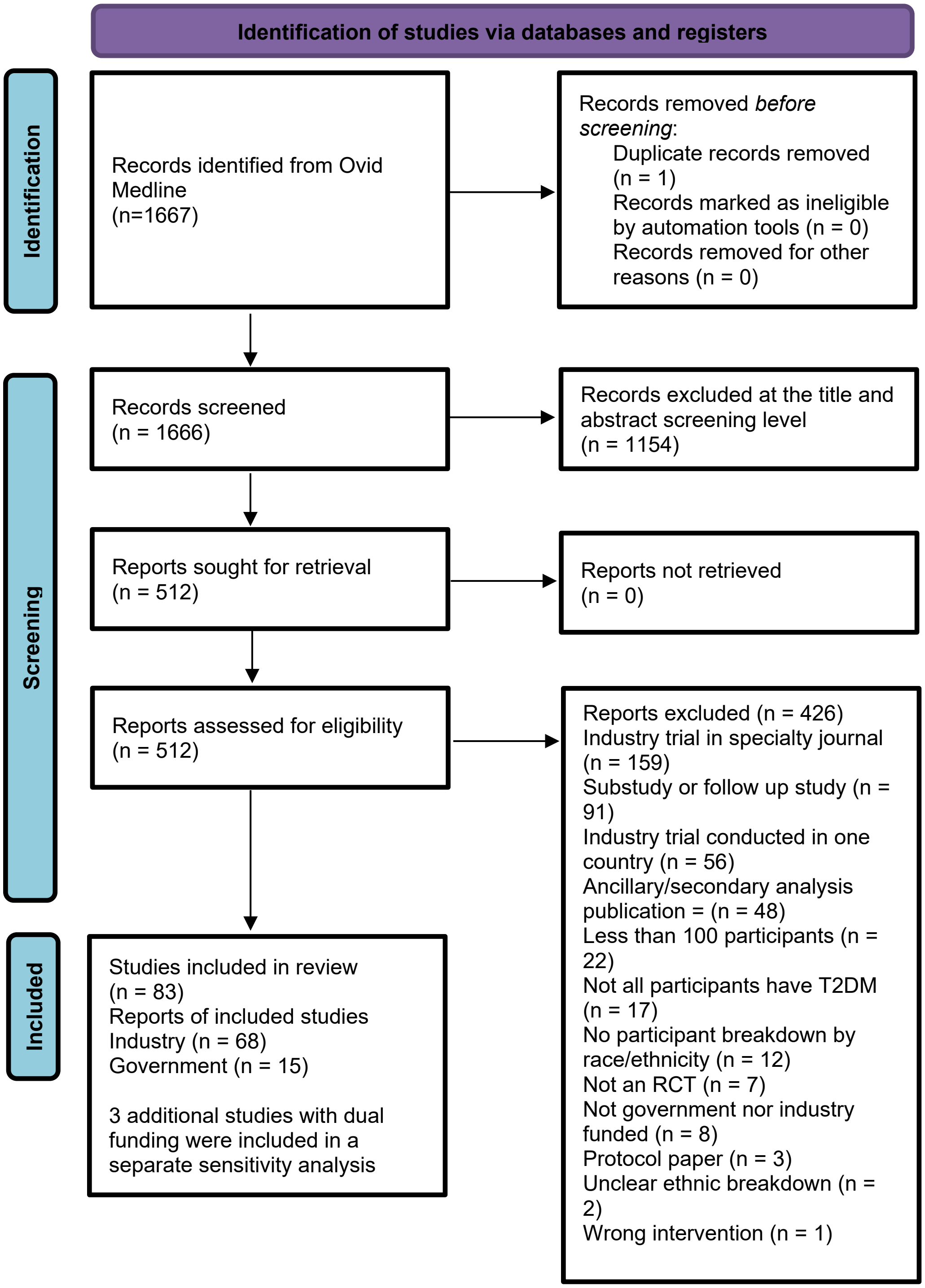

**ESM Appendix 1. Sensitivity analysis with varying worldwide population proportions**

Variation 1: Worldwide population proportion is 90% racialised and 10% white.

**ESM Fig. 2a.** Sensitivity Analysis 90/10 racialised/white comparison – White PPR Industry Trials.

| Study or Subgroup | log[Participant to Prevalence Ratio] | SE | Weight | Participant to Prevalence Ratio IV, Random, 95% CI |
|---|---|---|---|---|
| 1860-LIRA-DPP-4 2010 [25] | 0.28605035 | 0.11389498 | 1.5% | 1.33 [1.06, 1.66] |
| 4-T Study Group 2007 [26] | 0.18583238 | 0.14040374 | 1.5% | 1.20 [0.91, 1.59] |
| ADOPT 2006 [27] | 0.47110518 | 0.04737161 | 1.6% | 1.60 [1.46, 1.76] |
| AleCardio 2014 [28] | 0.318461 | 0.02495671 | 1.6% | 1.38 [1.31, 1.44] |
| ALTITUDE 2012 [29] | -0.30154 | 0.02180951 | 1.6% | 0.74 [0.71, 0.77] |
| ARTS-DN 2015 [30] | 0.09431606 | 0.09560066 | 1.5% | 1.10 [0.91, 1.33] |
| AVOID 2008 [31] | 0.28829694 | 0.12075307 | 1.5% | 1.33 [1.05, 1.69] |
| AWARD-4 2015 [32] | 0.48611608 | 0.0823548 | 1.5% | 1.63 [1.38, 1.91] |
| AWARD-6 2014 [33] | 0.57269304 | 0.11767119 | 1.5% | 1.77 [1.41, 2.23] |
| Bailey et al 2010 [34] | 0.28701563 | 0.13128156 | 1.5% | 1.33 [1.03, 1.72] |
| Barnett et al 2013 [35] | 2.86375616 | 0.35957175 | 1.0% | 17.53 [8.66, 35.46] |
| BEACON 2013 [36] | 0.469265 | 0.05125406 | 1.6% | 1.60 [1.45, 1.77] |
| BEGIN-Basal-Bolus Type 2 2012 [37] | 2.70953193 | 0.08425496 | 1.5% | 15.02 [12.74, 17.72] |
| Burant et al 2012 [38] | 0.40340909 | 0.12788441 | 1.5% | 1.50 [1.17, 1.92] |
| Buse et al 2011 [39] | 0.17620658 | 0.14905202 | 1.4% | 1.19 [0.89, 1.60] |
| CANTATA-SU 2013 [40] | 0.329979 | 0.05604587 | 1.6% | 1.39 [1.25, 1.55] |
| CANVAS 2017 [41] | 0.479519 | 0.02410063 | 1.6% | 1.62 [1.54, 1.69] |
| CARMELINA 2019 [42] | 0.045846 | 0.0300294 | 1.6% | 1.05 [0.99, 1.11] |
| CREDENCE 2019 [43] | 0.3173 | 0.03196017 | 1.6% | 1.37 [1.29, 1.46] |
| DAIS 2001 [44] | 0.22268246 | 0.2409472 | 1.3% | 1.25 [0.78, 2.00] |
| Dapagliflozin 006 2012 [45] | 0.37843644 | 0.16222142 | 1.4% | 1.46 [1.06, 2.01] |
| Davies et al 2017 [46] | 0.53764938 | 0.10610297 | 1.5% | 1.71 [1.39, 2.11] |
| DECLARE-TIMI 58 2019 [47] | 0.495166 | 0.01893114 | 1.6% | 1.64 [1.58, 1.70] |
| DETAIL 2004 [48] | 0.25056997 | 0.50404865 | 0.8% | 1.28 [0.48, 3.45] |
| DEVOTE 2017 [49] | 0.444321 | 0.0266499 | 1.6% | 1.56 [1.48, 1.64] |
| DIRECT-Protect2 2008 [50] | 0.226533 | 0.11781248 | 1.5% | 1.25 [1.00, 1.58] |
| DUAL V 2016 [51] | 0.21133466 | 0.18769887 | 1.4% | 1.24 [0.86, 1.78] |
| DURATION-1 2008 [52] | 0.34532121 | 0.14047221 | 1.5% | 1.41 [1.07, 1.86] |
| DURATION 2 2010 [53] | -0.4782629 | 0.09512285 | 1.5% | 0.62 [0.51, 0.75] |
| DURATION-3 2010 [54] | 2.71255804 | 0.12500214 | 1.5% | 15.07 [11.79, 19.25] |
| DURATION-6 2013 [55] | 2.70703698 | 0.08750506 | 1.5% | 14.98 [12.62, 17.79] |
| ELIXA 2015 [56] | 0.441584 | 0.02981229 | 1.6% | 1.56 [1.47, 1.65] |
| EMPA-REG OUTCOME 2015 [57] | -0.05656 | 0.02669347 | 1.6% | 0.95 [0.90, 1.00] |
| EUREXA 2012 [58] | 0.17791847 | 0.11474651 | 1.5% | 1.19 [0.95, 1.50] |
| EXAMINE 2013 [59] | 0.404381 | 0.03058806 | 1.6% | 1.50 [1.41, 1.59] |
| EXSCEL 2017 [60] | -0.011 | 0.01921066 | 1.6% | 0.99 [0.95, 1.03] |
| Frias et al 2018 [61] | 0.501435 | 0.14080336 | 1.5% | 1.65 [1.25, 2.18] |
| Gallwitz et al 2012 [62] | 2.73548863 | 0.07119336 | 1.5% | 15.42 [13.41, 17.73] |
| Harmony Outcomes 2018 [63] | 0.559583 | 0.02867699 | 1.6% | 1.75 [1.65, 1.85] |
| Heine et al 2005 [64] | 2.67619093 | 0.10699084 | 1.5% | 14.53 [11.78, 17.92] |
| INTERVAL 2013 [65] | 0.23378961 | 0.33886366 | 1.1% | 1.26 [0.65, 2.45] |
| IRMA-2 2001 [66] | 2.8700215 | 0.25346037 | 1.2% | 17.64 [10.73, 28.99] |
| LEAD-3 Mono 2009 [67] | 0.47724932 | 0.08860155 | 1.5% | 1.61 [1.35, 1.92] |
| LEAD-6 2009 [68] | 0.40359079 | 0.16930212 | 1.4% | 1.50 [1.07, 2.09] |
| LEADER 2016 [69] | 0.468826 | 0.02477695 | 1.6% | 1.60 [1.52, 1.68] |
| Lewis et al 2001 [70] | 2.57482455 | 0.05403071 | 1.6% | 13.13 [11.81, 14.60] |
| PERISCOPE 2008 [71] | 0.524753 | 0.11158527 | 1.5% | 1.69 [1.36, 2.10] |
| PIONEER 3 2019 [72] | 0.38172048 | 0.05106015 | 1.6% | 1.46 [1.33, 1.62] |
| PIONEER 4 2019 [73] | 0.40901919 | 0.08446961 | 1.5% | 1.51 [1.28, 1.78] |
| PIONEER 6 2019 [74] | 0.39887271 | 0.03958896 | 1.6% | 1.49 [1.38, 1.61] |
| PROactive 2005 [75] | 0.25247107 | 0.11707759 | 1.5% | 1.29 [1.02, 1.62] |
| Ray et al 2020 [76] | 0.134703 | 0.06177704 | 1.6% | 1.14 [1.01, 1.29] |
| RECORD 2009 [77] | 0.25584688 | 0.14512291 | 1.4% | 1.29 [0.97, 1.72] |
| RENAAL 2001 [78] | -0.12640234 | 0.05143634 | 1.6% | 0.88 [0.80, 0.97] |
| REWIND 2019 [79] | -0.01746 | 0.02344174 | 1.6% | 0.98 [0.94, 1.03] |
| RIO-Diabetes 2006 [80] | 2.77553622 | 0.0970279 | 1.5% | 16.05 [13.27, 19.41] |
| ROADMAP 2011 [81] | 0.26658692 | 1.41429306 | 0.2% | 1.31 [0.08, 20.87] |
| Rosenstock et al 2010 [82] | 2.50411241 | 0.08586737 | 1.5% | 12.23 [10.34, 14.47] |
| SAVOR-TIMI 53 2013 [83] | 0.43917315 | 0.01803878 | 1.6% | 1.55 [1.50, 1.61] |
| SCALE 2015 [84] | 2.71519309 | 0.09225312 | 1.5% | 15.11 [12.61, 18.10] |
| SONAR 2019 [85] | 0.17082913 | 0.03340333 | 1.6% | 1.19 [1.11, 1.27] |
| TECOS 2015 [86] | 0.336191 | 0.01767953 | 1.6% | 1.40 [1.35, 1.45] |
| THEMIS 2019 [87] | 0.38494 | 0.0159387 | 1.6% | 1.47 [1.42, 1.52] |
| TREAT 2009 [88] | 0.27194411 | 0.0327155 | 1.6% | 1.31 [1.23, 1.40] |
| VERIFY 2019 [89] | -0.23055889 | 0.04579523 | 1.6% | 0.79 [0.73, 0.87] |
| VERTIS CV 2020 [90] | 0.59368071 | 0.03364755 | 1.6% | 1.81 [1.70, 1.93] |
| Zinman et al 2007 [91] | 0.54574884 | 0.17732455 | 1.4% | 1.73 [1.22, 2.44] |
| Zinman et al 2011 [92] | 1.75296526 | 0.13714422 | 1.5% | 5.77 [4.41, 7.55] |
| **Total (95% CI)** | | | **100.0%** | **2.01 [1.78, 2.26]** |

Heterogeneity: $Tau^2 = 0.24$; $Chi^2 = 9833.45$, $df = 67$ ($P < 0.00001$); $I^2 = 99\%$

Test for overall effect: $Z = 11.25$ ($P < 0.00001$)

Participant to Prevalence Ratio IV, Random, 95% CI

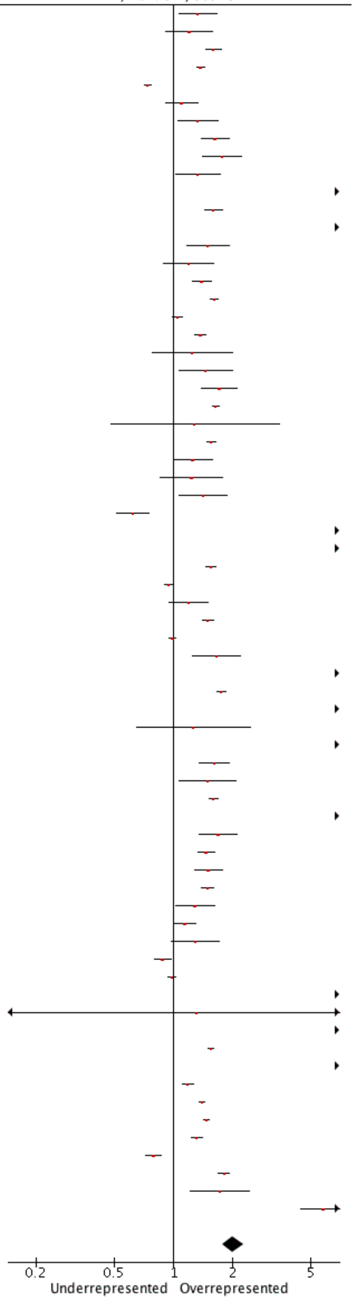

**ESM Fig. 2b.** Sensitivity Analysis 90/10 racialised/white – Racialised PPR Industry Trials.

| Study or Subgroup | log[Participant to Prevalence Ratio] | SE | Weight | Participant to Prevalence Ratio IV, Random, 95% CI |
|---|---|---|---|---|
| 1860-LIRA-DPP-4 2010 [25] | −0.95936968 | 0.11389498 | 1.5% | 0.38 [0.31, 0.48] |
| 4-T Study Group 2007 [26] | −1.10308987 | 0.14040374 | 1.5% | 0.33 [0.25, 0.44] |
| ADOPT 2006 [27] | −1.35263502 | 0.04737161 | 1.6% | 0.26 [0.24, 0.28] |
| AleCardio 2014 [28] | −0.43547148 | 0.02495671 | 1.6% | 0.65 [0.62, 0.68] |
| ALTITUDE 2012 [29] | 0.616105 | 0.02180951 | 1.6% | 1.85 [1.77, 1.93] |
| ARTS-DN 2015 [30] | −0.39096762 | 0.09560066 | 1.5% | 0.68 [0.56, 0.82] |
| AVOID 2008 [31] | −0.97403276 | 0.12075307 | 1.5% | 0.38 [0.30, 0.48] |
| AWARD-4 2015 [32] | −0.88993082 | 0.0823548 | 1.5% | 0.41 [0.35, 0.48] |
| AWARD-6 2014 [33] | −1.30102717 | 0.11767119 | 1.5% | 0.27 [0.22, 0.34] |
| Bailey et al 2010 [34] | −1.03480078 | 0.13128156 | 1.5% | 0.36 [0.27, 0.46] |
| Barnett et al 2013 [35] | −3.34861556 | 0.35957175 | 1.1% | 0.04 [0.02, 0.07] |
| BEACON 2013 [36] | −0.82951 | 0.05125406 | 1.5% | 0.44 [0.39, 0.48] |
| BEGIN-Basal-Bolus Type 2 2012 [37] | −1.70718484 | 0.08425496 | 1.5% | 0.18 [0.15, 0.21] |
| Burant et al 2012 [38] | −0.94742456 | 0.127884 | 1.5% | 0.39 [0.30, 0.50] |
| Buse et al 2011 [39] | −0.44460406 | 0.14905202 | 1.5% | 0.64 [0.48, 0.86] |
| CANTATA-SU 2013 [40] | −0.45892 | 0.05604587 | 1.5% | 0.63 [0.57, 0.71] |
| CANVAS 2017 [41] | −0.86572 | 0.02410063 | 1.6% | 0.42 [0.40, 0.44] |
| CARMELINA 2019 [42] | −0.16663 | 0.0300294 | 1.6% | 0.85 [0.80, 0.90] |
| CREDENCE 2019 [43] | −0.43315 | 0.03196017 | 1.6% | 0.65 [0.61, 0.69] |
| DAIS 2001 [44] | −1.69308864 | 0.2409472 | 1.3% | 0.18 [0.11, 0.29] |
| Dapagliflozin 006 2012 [45] | −1.94395129 | 0.16222142 | 1.4% | 0.14 [0.10, 0.20] |
| Davies et al 2017 [46] | −1.10947336 | 0.10610297 | 1.5% | 0.33 [0.27, 0.41] |
| DECLARE-TIMI 58 2019 [47] | −0.9244 | 0.01893114 | 1.6% | 0.40 [0.38, 0.41] |
| DETAIL 2004 [48] | −2.68314552 | 0.50404865 | 0.8% | 0.07 [0.03, 0.18] |
| DEVOTE 2017 [49] | −0.74794 | 0.0266499 | 1.6% | 0.47 [0.45, 0.50] |
| DIRECT-Protect2 2008 [50] | −1.78273 | 0.11781248 | 1.5% | 0.17 [0.13, 0.21] |
| DUAL V 2016 [51] | −1.46934682 | 0.18769887 | 1.4% | 0.23 [0.16, 0.33] |
| DURATION-1 2008 [52] | −0.70963839 | 0.14047221 | 1.5% | 0.49 [0.37, 0.65] |
| DURATION 2 2010 [53] | 0.38415789 | 0.09512285 | 1.5% | 1.47 [1.22, 1.77] |
| DURATION-3 2010 [54] | −1.72194756 | 0.12500214 | 1.5% | 0.18 [0.14, 0.23] |
| DURATION-6 2013 [55] | −1.69520804 | 0.08750506 | 1.5% | 0.18 [0.15, 0.22] |
| ELIXA 2015 [56] | −0.73949 | 0.02981229 | 1.6% | 0.48 [0.45, 0.51] |
| EMPA-REG OUTCOME 2015 [57] | 0.16543 | 0.02669347 | 1.6% | 1.18 [1.12, 1.24] |
| EUREXA 2012 [58] | −1.01362501 | 0.11474651 | 1.5% | 0.36 [0.29, 0.45] |
| EXAMINE 2013 [59] | −0.63333 | 0.03058806 | 1.6% | 0.53 [0.50, 0.56] |
| EXSCEL 2017 [60] | 0.035167 | 0.01921066 | 1.6% | 1.04 [1.00, 1.08] |
| Frias et al 2018 [61] | −0.94919 | 0.14080336 | 1.5% | 0.39 [0.29, 0.51] |
| Gallwitz et al 2012 [62] | −1.84317796 | 0.07119336 | 1.5% | 0.16 [0.14, 0.18] |
| Harmony Outcomes 2018 [63] | −1.2242 | 0.02867699 | 1.6% | 0.29 [0.28, 0.31] |
| Heine et al 2005 [64] | −1.56001073 | 0.10699084 | 1.5% | 0.21 [0.17, 0.26] |
| INTERVAL 2013 [65] | −1.9783755 | 0.33886366 | 1.1% | 0.14 [0.07, 0.27] |
| IRMA-2 2001 [66] | −3.55079399 | 0.25346037 | 1.3% | 0.03 [0.02, 0.05] |
| LEAD-3 Mono 2009 [67] | −0.85755777 | 0.08860155 | 1.5% | 0.42 [0.36, 0.50] |
| LEAD-6 2009 [68] | −1.55240627 | 0.16930212 | 1.4% | 0.21 [0.15, 0.30] |
| LEADER 2016 [69] | −0.82799935 | 0.02477695 | 1.6% | 0.44 [0.42, 0.46] |
| Lewis et al 2001 [70] | −1.23133314 | 0.05403071 | 1.5% | 0.29 [0.26, 0.32] |
| PERISCOPE 2008 [71] | −1.04872 | 0.11158527 | 1.5% | 0.35 [0.28, 0.44] |
| PIONEER 3 2019 [72] | −0.57549323 | 0.05106015 | 1.5% | 0.56 [0.51, 0.62] |
| PIONEER 4 2019 [73] | −0.64575943 | 0.08446961 | 1.5% | 0.52 [0.44, 0.62] |
| PIONEER 6 2019 [74] | −0.6188366 | 0.03958896 | 1.6% | 0.54 [0.50, 0.58] |
| PROactive 2005 [75] | −2.8076089 | 0.11707759 | 1.5% | 0.06 [0.05, 0.08] |
| Ray et al 2020 [76] | −0.63823 | 0.06177704 | 1.5% | 0.53 [0.47, 0.60] |
| RECORD 2009 [77] | −3.07676295 | 0.14512291 | 1.5% | 0.05 [0.03, 0.06] |
| RENAAL 2001 [78] | 0.13654033 | 0.05143634 | 1.5% | 1.15 [1.04, 1.27] |
| REWIND 2019 [79] | 0.056527 | 0.02344174 | 1.6% | 1.06 [1.01, 1.11] |
| RIO-Diabetes 2006 [80] | −2.10754059 | 0.0970279 | 1.5% | 0.12 [0.10, 0.15] |
| ROADMAP 2011 [81] | −7.64122356 | 1.41429306 | 0.2% | 0.00 [0.00, 0.01] |
| Rosenstock et al 2010 [82] | −1.06644372 | 0.08586737 | 1.5% | 0.34 [0.29, 0.41] |
| SAVOR-TIMI 53 2013 [83] | −0.73213606 | 0.01803878 | 1.6% | 0.48 [0.46, 0.50] |
| SCALE 2015 [84] | −1.73501964 | 0.09225312 | 1.5% | 0.18 [0.15, 0.21] |
| SONAR 2019 [85] | −0.19282598 | 0.03340333 | 1.6% | 0.82 [0.77, 0.88] |
| TECOS 2015 [86] | −0.47192 | 0.01767953 | 1.6% | 0.62 [0.60, 0.65] |
| THEMIS 2019 [87] | −0.58343 | 0.0159387 | 1.6% | 0.56 [0.54, 0.58] |
| TREAT 2009 [88] | −0.34843096 | 0.0327155 | 1.6% | 0.71 [0.66, 0.75] |
| VERIFY 2019 [89] | 0.51502772 | 0.04579523 | 1.6% | 1.67 [1.53, 1.83] |
| VERTIS CV 2020 [90] | −1.4403285 | 0.03364755 | 1.6% | 0.24 [0.22, 0.25] |
| Zinman et al 2007 [91] | −1.15003467 | 0.17732455 | 1.4% | 0.32 [0.22, 0.45] |
| Zinman et al 2011 [92] | −0.3265246 | 0.13714422 | 1.5% | 0.72 [0.55, 0.94] |
| **Total (95% CI)** | | | **100.0%** | **0.36 [0.32, 0.41]** |

Heterogeneity: $Tau^2 = 0.29$; $Chi^2 = 11735.52$, $df = 67$ ($P < 0.00001$); $I^2 = 99\%$
Test for overall effect: $Z = 15.03$ ($P < 0.00001$)

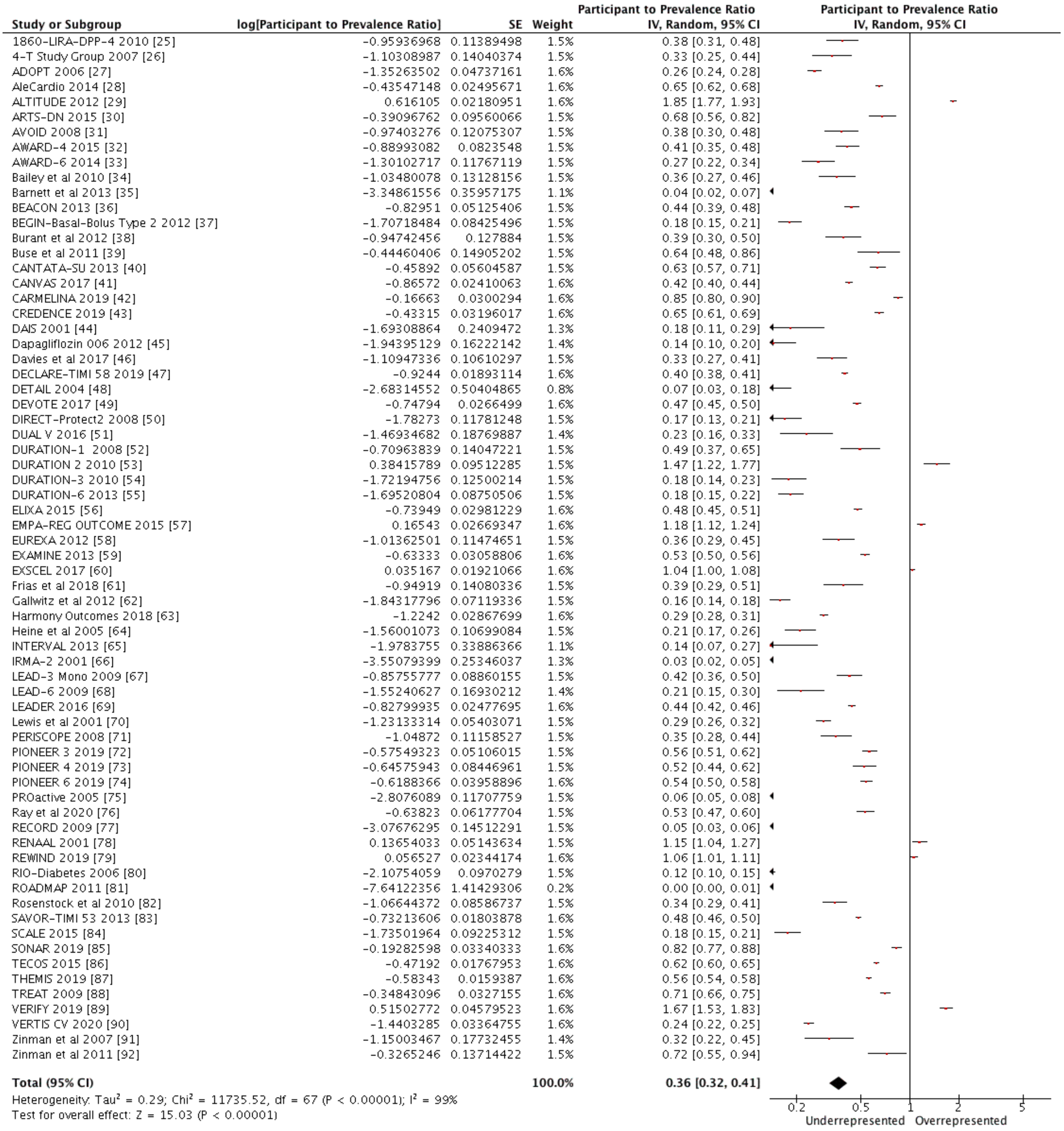

Variation 2: Worldwide population proportion is 87.5% racialised, 12.5% white

**ESM Fig. 3a.** Sensitivity Analysis 87.5/12.5 racialised/white – White PPR Industry Trials.

| Study or Subgroup | log[Participant to Prevalence Ratio] | SE | Weight | Participant to Prevalence Ratio IV, Random, 95% CI |
|---|---|---|---|---|
| 1860-LIRA-DPP-4 2010 [25] | 0.28605035 | 0.11389498 | 1.5% | 1.33 [1.06, 1.66] |
| 4-T Study Group 2007 [26] | 0.18583238 | 0.14040374 | 1.5% | 1.20 [0.91, 1.59] |
| ADOPT 2006 [27] | 0.47110518 | 0.04737161 | 1.6% | 1.60 [1.46, 1.76] |
| AleCardio 2014 [28] | 0.318461 | 0.02495671 | 1.6% | 1.38 [1.31, 1.44] |
| ALTITUDE 2012 [29] | -0.30154 | 0.02180951 | 1.6% | 0.74 [0.71, 0.77] |
| ARTS-DN 2015 [30] | 0.09431606 | 0.09560066 | 1.5% | 1.10 [0.91, 1.33] |
| AVOID 2008 [31] | 0.28829694 | 0.12075307 | 1.5% | 1.33 [1.05, 1.69] |
| AWARD-4 2015 [32] | 0.48611608 | 0.0823548 | 1.5% | 1.63 [1.38, 1.91] |
| AWARD-6 2014 [33] | 0.57269304 | 0.11767119 | 1.5% | 1.77 [1.41, 2.23] |
| Bailey et al 2010 [34] | 0.28701563 | 0.13128156 | 1.5% | 1.33 [1.03, 1.72] |
| Barnett et al 2013 [35] | 2.62807888 | 0.35957175 | 1.0% | 13.85 [6.84, 28.02] |
| BEACON 2013 [36] | 0.469265 | 0.05125406 | 1.6% | 1.60 [1.45, 1.77] |
| BEGIN-Basal-Bolus Type 2 2012 [37] | 2.47385465 | 0.08425496 | 1.5% | 11.87 [10.06, 14.00] |
| Burant et al 2012 [38] | 0.40340909 | 0.12788441 | 1.5% | 1.50 [1.17, 1.92] |
| Buse et al 2011 [39] | 0.17620658 | 0.14905202 | 1.4% | 1.19 [0.89, 1.60] |
| CANTATA-SU 2013 [40] | 0.329979 | 0.05604587 | 1.6% | 1.39 [1.25, 1.55] |
| CANVAS 2017 [41] | 0.479519 | 0.02410063 | 1.6% | 1.62 [1.54, 1.69] |
| CARMELINA 2019 [42] | 0.045846 | 0.0300294 | 1.6% | 1.05 [0.99, 1.11] |
| CREDENCE 2019 [43] | 0.3173 | 0.03196017 | 1.6% | 1.37 [1.29, 1.46] |
| DAIS 2001 [44] | 0.22268246 | 0.2409472 | 1.2% | 1.25 [0.78, 2.00] |
| Dapagliflozin 006 2012 [45] | 0.37843644 | 0.16222142 | 1.4% | 1.46 [1.06, 2.01] |
| Davies et al 2017 [46] | 0.53764938 | 0.10610297 | 1.5% | 1.71 [1.39, 2.11] |
| DECLARE-TIMI 58 2019 [47] | 0.495166 | 0.01893114 | 1.6% | 1.64 [1.58, 1.70] |
| DETAIL 2004 [48] | 0.25056997 | 0.50404865 | 0.7% | 1.28 [0.48, 3.45] |
| DEVOTE 2017 [49] | 0.444321 | 0.0266499 | 1.6% | 1.56 [1.48, 1.64] |
| DIRECT-Protect2 2008 [50] | 0.226533 | 0.11781248 | 1.5% | 1.25 [1.00, 1.58] |
| DUAL V 2016 [51] | 0.21133466 | 0.18769887 | 1.4% | 1.24 [0.86, 1.78] |
| DURATION-1 2008 [52] | 0.34532121 | 0.14047221 | 1.5% | 1.41 [1.07, 1.86] |
| DURATION 2 2010 [53] | -0.4782629 | 0.09512285 | 1.5% | 0.62 [0.51, 0.75] |
| DURATION-3 2010 [54] | 2.47688075 | 0.12500214 | 1.5% | 11.90 [9.32, 15.21] |
| DURATION-6 2013 [55] | 2.47135969 | 0.08750506 | 1.5% | 11.84 [9.97, 14.05] |
| ELIXA 2015 [56] | 0.441584 | 0.02981229 | 1.6% | 1.56 [1.47, 1.65] |
| EMPA-REG OUTCOME 2015 [57] | -0.05656 | 0.02669347 | 1.6% | 0.95 [0.90, 1.00] |
| EUREXA 2012 [58] | 0.17791847 | 0.11474651 | 1.5% | 1.19 [0.95, 1.50] |
| EXAMINE 2013 [59] | 0.404381 | 0.03058806 | 1.6% | 1.50 [1.41, 1.59] |
| EXSCEL 2017 [60] | -0.011 | 0.01921066 | 1.6% | 0.99 [0.95, 1.03] |
| Frias et al 2018 [61] | 0.501435 | 0.14080336 | 1.4% | 1.65 [1.25, 2.18] |
| Gallwitz et al 2012 [62] | 2.49981135 | 0.07119336 | 1.5% | 12.18 [10.59, 14.00] |
| Harmony Outcomes 2018 [63] | 0.559583 | 0.02867699 | 1.6% | 1.75 [1.65, 1.85] |
| Heine et al 2005 [64] | 2.44051364 | 0.10699084 | 1.5% | 11.48 [9.31, 14.16] |
| INTERVAL 2013 [65] | 0.23378961 | 0.33886366 | 1.0% | 1.26 [0.65, 2.45] |
| IRMA-2 2001 [66] | 2.63434422 | 0.25346037 | 1.2% | 13.93 [8.48, 22.90] |
| LEAD-3 Mono 2009 [67] | 0.47724932 | 0.08860155 | 1.5% | 1.61 [1.35, 1.92] |
| LEAD-6 2009 [68] | 0.40359079 | 0.16930212 | 1.4% | 1.50 [1.07, 2.09] |
| LEADER 2016 [69] | 0.468826 | 0.02477695 | 1.6% | 1.60 [1.52, 1.68] |
| Lewis et al 2001 [70] | 2.33914726 | 0.05403071 | 1.6% | 10.37 [9.33, 11.53] |
| PERISCOPE 2008 [71] | 0.524753 | 0.11158527 | 1.5% | 1.69 [1.36, 2.10] |
| PIONEER 3 2019 [72] | 0.38172048 | 0.05106015 | 1.6% | 1.46 [1.33, 1.62] |
| PIONEER 4 2019 [73] | 0.40901919 | 0.08446961 | 1.5% | 1.51 [1.28, 1.78] |
| PIONEER 6 2019 [74] | 0.39887271 | 0.03958896 | 1.6% | 1.49 [1.38, 1.61] |
| PROactive 2005 [75] | 0.25247107 | 0.11707759 | 1.5% | 1.29 [1.02, 1.62] |
| Ray et al 2020 [76] | 0.134703 | 0.06177704 | 1.6% | 1.14 [1.01, 1.29] |
| RECORD 2009 [77] | 0.25584688 | 0.14512291 | 1.4% | 1.29 [0.97, 1.72] |
| RENAAL 2001 [78] | -0.12640234 | 0.05143634 | 1.6% | 0.88 [0.80, 0.97] |
| REWIND 2019 [79] | -0.01746 | 0.02344174 | 1.6% | 0.98 [0.94, 1.03] |
| RIO-Diabetes 2006 [80] | 2.53985893 | 0.0970279 | 1.5% | 12.68 [10.48, 15.33] |
| ROADMAP 2011 [81] | 0.26658692 | 1.41429306 | 0.1% | 1.31 [0.08, 20.87] |
| Rosenstock et al 2010 [82] | 2.26843512 | 0.08586737 | 1.5% | 9.66 [8.17, 11.44] |
| SAVOR-TIMI 53 2013 [83] | 0.43917315 | 0.01803878 | 1.6% | 1.55 [1.50, 1.61] |
| SCALE 2015 [84] | 2.4795158 | 0.09225312 | 1.5% | 11.94 [9.96, 14.30] |
| SONAR 2019 [85] | 0.17082913 | 0.03340333 | 1.6% | 1.19 [1.11, 1.27] |
| TECOS 2015 [86] | 0.336191 | 0.01767953 | 1.6% | 1.40 [1.35, 1.45] |
| THEMIS 2019 [87] | 0.38494 | 0.0159387 | 1.6% | 1.47 [1.42, 1.52] |
| TREAT 2009 [88] | 0.27194411 | 0.0327155 | 1.6% | 1.31 [1.23, 1.40] |
| VERIFY 2019 [89] | -0.23055889 | 0.04579523 | 1.6% | 0.79 [0.73, 0.87] |
| VERTIS CV 2020 [90] | 0.59368071 | 0.03364755 | 1.6% | 1.81 [1.70, 1.93] |
| Zinman et al 2007 [91] | 0.54574884 | 0.17732455 | 1.4% | 1.73 [1.22, 2.44] |
| Zinman et al 2011 [92] | 1.51728797 | 0.13714422 | 1.5% | 4.56 [3.49, 5.97] |
| **Total (95% CI)** | | | **100.0%** | **1.92 [1.72, 2.15]** |

Heterogeneity: $Tau^2 = 0.21$; $Chi^2 = 8412.68$, $df = 67$ ($P < 0.00001$); $I^2 = 99\%$
Test for overall effect: $Z = 11.38$ ($P < 0.00001$)

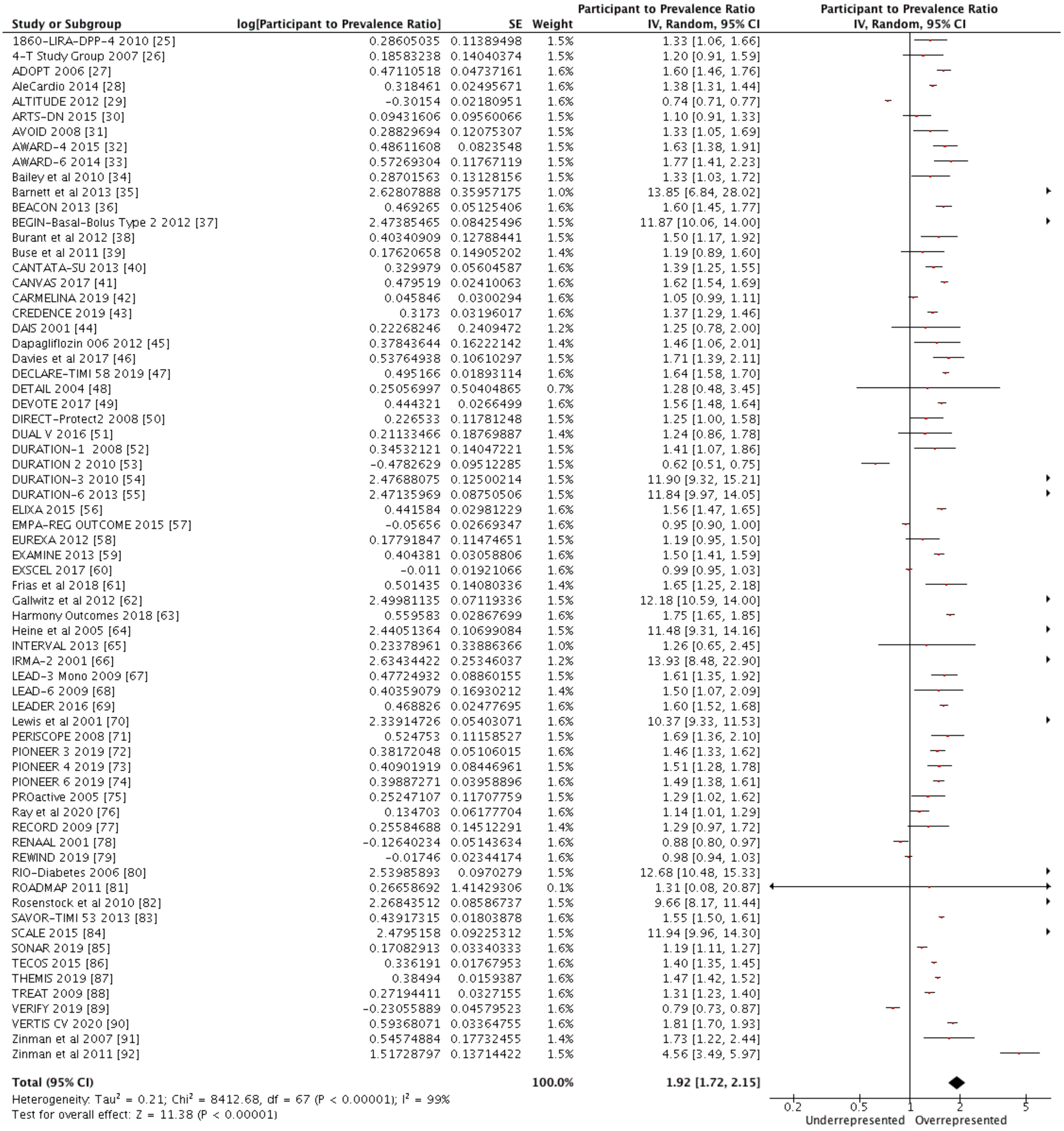

**ESM Fig. 3b.** Sensitivity Analysis 87.5/12.5 racialised/white – Racialised PPR Industry Trials

| Study or Subgroup | log[Participant to Prevalence Ratio] | SE | Weight | Participant to Prevalence Ratio IV, Random, 95% CI |
|---|---|---|---|---|
| 1860-LIRA-DPP-4 2010 [25] | -0.95936968 | 0.11389498 | 1.5% | 0.38 [0.31, 0.48] |
| 4-T Study Group 2007 [26] | -1.10308987 | 0.14040374 | 1.5% | 0.33 [0.25, 0.44] |
| ADOPT 2006 [27] | -1.35263502 | 0.04737161 | 1.6% | 0.26 [0.24, 0.28] |
| AleCardio 2014 [28] | -0.43547148 | 0.02495671 | 1.6% | 0.65 [0.62, 0.68] |
| ALTITUDE 2012 [29] | 0.616105 | 0.02180951 | 1.6% | 1.85 [1.77, 1.93] |
| ARTS-DN 2015 [30] | -0.39096762 | 0.09560066 | 1.5% | 0.68 [0.56, 0.82] |
| AVOID 2008 [31] | -0.97403276 | 0.12075307 | 1.5% | 0.38 [0.30, 0.48] |
| AWARD-4 2015 [32] | -0.88993082 | 0.0823548 | 1.5% | 0.41 [0.35, 0.48] |
| AWARD-6 2014 [33] | -1.30102717 | 0.11767119 | 1.5% | 0.27 [0.22, 0.34] |
| Bailey et al 2010 [34] | -1.03480078 | 0.13128156 | 1.5% | 0.36 [0.27, 0.46] |
| Barnett et al 2013 [35] | -3.33297842 | 0.35957175 | 1.1% | 0.04 [0.02, 0.07] |
| BEACON 2013 [36] | -0.82951 | 0.05125406 | 1.5% | 0.44 [0.39, 0.48] |
| BEGIN-Basal-Bolus Type 2 2012 [37] | -1.6915477 | 0.08425496 | 1.5% | 0.18 [0.16, 0.22] |
| Burant et al 2012 [38] | -0.94742456 | 0.127884 | 1.5% | 0.39 [0.30, 0.50] |
| Buse et al 2011 [39] | -0.44460406 | 0.14905202 | 1.5% | 0.64 [0.48, 0.86] |
| CANTATA-SU 2013 [40] | -0.45892 | 0.05604587 | 1.5% | 0.63 [0.57, 0.71] |
| CANVAS 2017 [41] | -0.86572 | 0.02410063 | 1.6% | 0.42 [0.40, 0.44] |
| CARMELINA 2019 [42] | -0.16663 | 0.0300294 | 1.6% | 0.85 [0.80, 0.90] |
| CREDENCE 2019 [43] | -0.43315 | 0.03196017 | 1.6% | 0.65 [0.61, 0.69] |
| DAIS 2001 [44] | -1.69308864 | 0.2409472 | 1.3% | 0.18 [0.11, 0.29] |
| Dapagliflozin 006 2012 [45] | -1.94395129 | 0.16222142 | 1.4% | 0.14 [0.10, 0.20] |
| Davies et al 2017 [46] | -1.10947336 | 0.10610297 | 1.5% | 0.33 [0.27, 0.41] |
| DECLARE-TIMI 58 2019 [47] | -0.9244 | 0.01893114 | 1.6% | 0.40 [0.38, 0.41] |
| DETAIL 2004 [48] | -2.68314552 | 0.50404865 | 0.8% | 0.07 [0.03, 0.18] |
| DEVOTE 2017 [49] | -0.74794 | 0.0266499 | 1.6% | 0.47 [0.45, 0.50] |
| DIRECT-Protect2 2008 [50] | -1.78273 | 0.11781248 | 1.5% | 0.17 [0.13, 0.21] |
| DUAL V 2016 [51] | -1.46934682 | 0.18769887 | 1.4% | 0.23 [0.16, 0.33] |
| DURATION-1 2008 [52] | -0.70963839 | 0.14047221 | 1.5% | 0.49 [0.37, 0.65] |
| DURATION 2 2010 [53] | 0.38415789 | 0.09512285 | 1.5% | 1.47 [1.22, 1.77] |
| DURATION-3 2010 [54] | -1.70631042 | 0.12500214 | 1.5% | 0.18 [0.14, 0.23] |
| DURATION-6 2013 [55] | -1.67957089 | 0.08750506 | 1.5% | 0.19 [0.16, 0.22] |
| ELIXA 2015 [56] | -0.73949 | 0.02981229 | 1.6% | 0.48 [0.45, 0.51] |
| EMPA-REG OUTCOME 2015 [57] | 0.16543 | 0.02669347 | 1.6% | 1.18 [1.12, 1.24] |
| EUREXA 2012 [58] | -1.01362501 | 0.11474651 | 1.5% | 0.36 [0.29, 0.45] |
| EXAMINE 2013 [59] | -0.63333 | 0.03058806 | 1.6% | 0.53 [0.50, 0.56] |
| EXSCEL 2017 [60] | 0.035167 | 0.01921066 | 1.6% | 1.04 [1.00, 1.08] |
| Frias et al 2018 [61] | -0.94919 | 0.14080336 | 1.5% | 0.39 [0.29, 0.51] |
| Gallwitz et al 2012 [62] | -1.82754082 | 0.07119336 | 1.5% | 0.16 [0.14, 0.18] |
| Harmony Outcomes 2018 [63] | -1.2242 | 0.02867699 | 1.6% | 0.29 [0.28, 0.31] |
| Heine et al 2005 [64] | -1.54437359 | 0.10699084 | 1.5% | 0.21 [0.17, 0.26] |
| INTERVAL 2013 [65] | -1.9783755 | 0.33886366 | 1.1% | 0.14 [0.07, 0.27] |
| IRMA-2 2001 [66] | -3.53515684 | 0.25346037 | 1.3% | 0.03 [0.02, 0.05] |
| LEAD-3 Mono 2009 [67] | -0.85755777 | 0.08860155 | 1.5% | 0.42 [0.36, 0.50] |
| LEAD-6 2009 [68] | -1.55240627 | 0.16930212 | 1.4% | 0.21 [0.15, 0.30] |
| LEADER 2016 [69] | -0.82799935 | 0.02477695 | 1.6% | 0.44 [0.42, 0.46] |
| Lewis et al 2001 [70] | -1.215696 | 0.05403071 | 1.5% | 0.30 [0.27, 0.33] |
| PERISCOPE 2008 [71] | -1.04872 | 0.11158527 | 1.5% | 0.35 [0.28, 0.44] |
| PIONEER 3 2019 [72] | -0.57549323 | 0.05106015 | 1.5% | 0.56 [0.51, 0.62] |
| PIONEER 4 2019 [73] | -0.64575943 | 0.08446961 | 1.5% | 0.52 [0.44, 0.62] |
| PIONEER 6 2019 [74] | -0.6188366 | 0.03958896 | 1.6% | 0.54 [0.50, 0.58] |
| PROactive 2005 [75] | -2.8076089 | 0.11707759 | 1.5% | 0.06 [0.05, 0.08] |
| Ray et al 2020 [76] | -0.63823 | 0.06177704 | 1.5% | 0.53 [0.47, 0.60] |
| RECORD 2009 [77] | -3.07676295 | 0.14512291 | 1.5% | 0.05 [0.03, 0.06] |
| RENAAL 2001 [78] | 0.13654033 | 0.05143634 | 1.5% | 1.15 [1.04, 1.27] |
| REWIND 2019 [79] | 0.056527 | 0.02344174 | 1.6% | 1.06 [1.01, 1.11] |
| RIO-Diabetes 2006 [80] | -2.09190345 | 0.0970279 | 1.5% | 0.12 [0.10, 0.15] |
| ROADMAP 2011 [81] | -7.64122356 | 1.41429306 | 0.2% | 0.00 [0.00, 0.01] |
| Rosenstock et al 2010 [82] | -1.05080658 | 0.08586737 | 1.5% | 0.35 [0.30, 0.41] |
| SAVOR-TIMI 53 2013 [83] | -0.73213606 | 0.01803878 | 1.6% | 0.48 [0.46, 0.50] |
| SCALE 2015 [84] | -1.7193825 | 0.09225312 | 1.5% | 0.18 [0.15, 0.21] |
| SONAR 2019 [85] | -0.19282598 | 0.03340333 | 1.6% | 0.82 [0.77, 0.88] |
| TECOS 2015 [86] | -0.47192 | 0.01767953 | 1.6% | 0.62 [0.60, 0.65] |
| THEMIS 2019 [87] | -0.58343 | 0.0159387 | 1.6% | 0.56 [0.54, 0.58] |
| TREAT 2009 [88] | -0.34843096 | 0.0327155 | 1.6% | 0.71 [0.66, 0.75] |
| VERIFY 2019 [89] | 0.51502772 | 0.04579523 | 1.6% | 1.67 [1.53, 1.83] |
| VERTIS CV 2020 [90] | -1.4403285 | 0.03364755 | 1.6% | 0.24 [0.22, 0.25] |
| Zinman et al 2007 [91] | -1.15003467 | 0.17732455 | 1.4% | 0.32 [0.22, 0.45] |
| Zinman et al 2011 [92] | -0.3108874 | 0.13714422 | 1.5% | 0.73 [0.56, 0.96] |
| **Total (95% CI)** | | | **100.0%** | **0.36 [0.32, 0.42]** |

Heterogeneity: $Tau^2 = 0.29$; $Chi^2 = 11691.48$, $df = 67$ ($P < 0.00001$); $I^2 = 99\%$

Test for overall effect: $Z = 15.01$ ($P < 0.00001$)

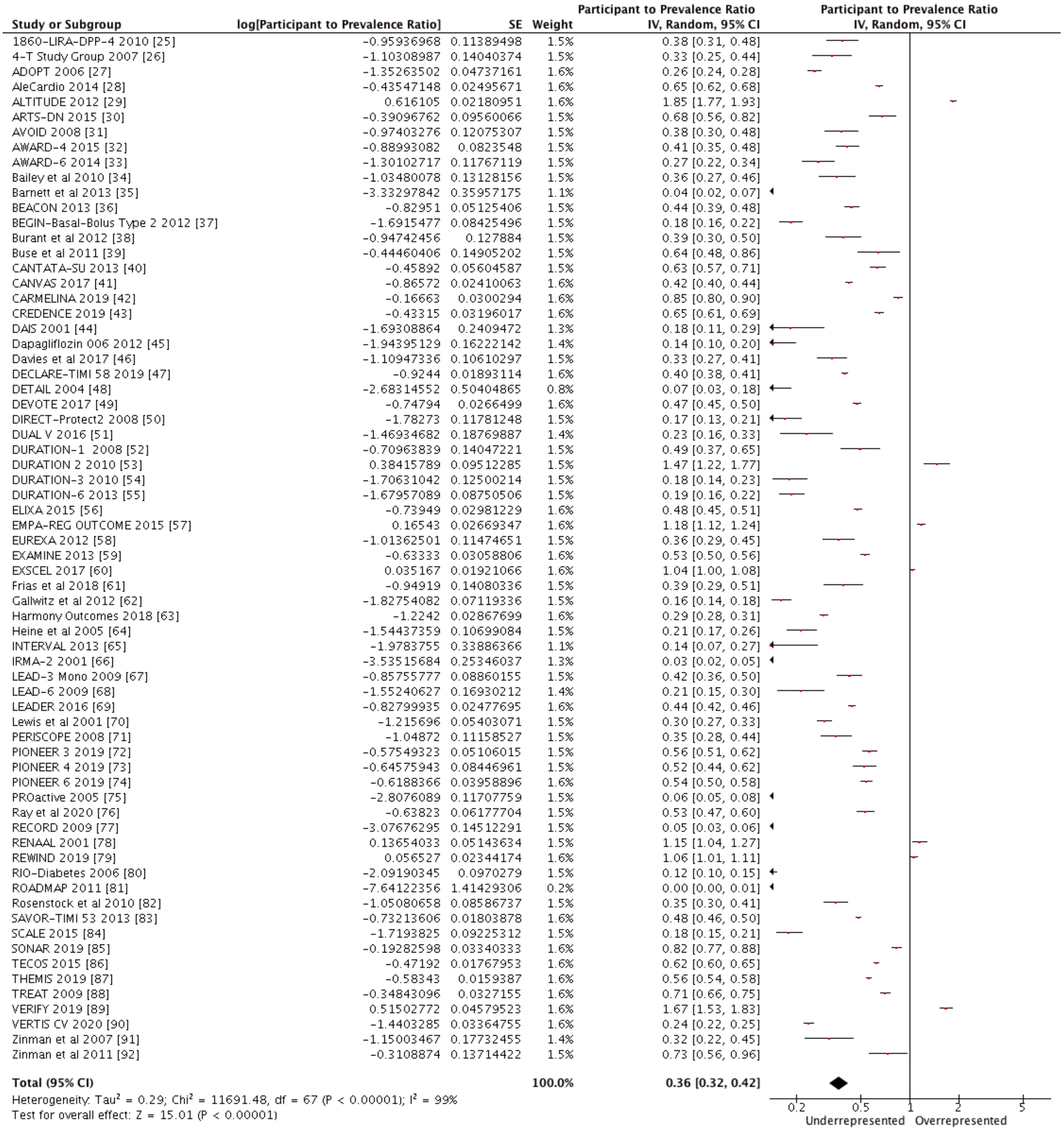

Variation 3: Worldwide population proportion is 85% racialised, 15% white

**ESM Fig. 4a.** Sensitivity Analysis 85/15 racialised/white – White PPR Industry Trials.

| Study or Subgroup | log[Participant to Prevalence Ratio] | SE | Weight | Participant to Prevalence Ratio IV, Random, 95% CI |
|---|---|---|---|---|
| 1860-LIRA-DPP-4 2010 [25] | 0.28605035 | 0.11389498 | 1.5% | 1.33 [1.06, 1.66] |
| 4-T Study Group 2007 [26] | 0.18583238 | 0.14040374 | 1.4% | 1.20 [0.91, 1.59] |
| ADOPT 2006 [27] | 0.47110518 | 0.04737161 | 1.6% | 1.60 [1.46, 1.76] |
| AleCardio 2014 [28] | 0.318461 | 0.02495671 | 1.6% | 1.38 [1.31, 1.44] |
| ALTITUDE 2012 [29] | -0.30154 | 0.02180951 | 1.6% | 0.74 [0.71, 0.77] |
| ARTS-DN 2015 [30] | 0.09431606 | 0.09560066 | 1.5% | 1.10 [0.91, 1.33] |
| AVOID 2008 [31] | 0.28829694 | 0.12075307 | 1.5% | 1.33 [1.05, 1.69] |
| AWARD-4 2015 [32] | 0.48611608 | 0.0823548 | 1.5% | 1.63 [1.38, 1.91] |
| AWARD-6 2014 [33] | 0.57269304 | 0.11767119 | 1.5% | 1.77 [1.41, 2.23] |
| Bailey et al 2010 [34] | 0.28701563 | 0.13128156 | 1.5% | 1.33 [1.03, 1.72] |
| Barnett et al 2013 [35] | 2.43306449 | 0.35957175 | 0.9% | 11.39 [5.63, 23.05] |
| BEACON 2013 [36] | 0.469265 | 0.05125406 | 1.6% | 1.60 [1.45, 1.77] |
| BEGIN-Basal-Bolus Type 2 2012 [37] | 2.27884026 | 0.08425496 | 1.5% | 9.77 [8.28, 11.52] |
| Burant et al 2012 [38] | 0.40340909 | 0.12788441 | 1.5% | 1.50 [1.17, 1.92] |
| Buse et al 2011 [39] | 0.17620658 | 0.14905202 | 1.4% | 1.19 [0.89, 1.60] |
| CANTATA-SU 2013 [40] | 0.329979 | 0.05604587 | 1.6% | 1.39 [1.25, 1.55] |
| CANVAS 2017 [41] | 0.479519 | 0.02410063 | 1.6% | 1.62 [1.54, 1.69] |
| CARMELINA 2019 [42] | 0.045846 | 0.0300294 | 1.6% | 1.05 [0.99, 1.11] |
| CREDENCE 2019 [43] | 0.3173 | 0.03196017 | 1.6% | 1.37 [1.29, 1.46] |
| DAIS 2001 [44] | 0.22268246 | 0.2409472 | 1.2% | 1.25 [0.78, 2.00] |
| Dapagliflozin 006 2012 [45] | 0.37843644 | 0.16222142 | 1.4% | 1.46 [1.06, 2.01] |
| Davies et al 2017 [46] | 0.53764938 | 0.10610297 | 1.5% | 1.71 [1.39, 2.11] |
| DECLARE-TIMI 58 2019 [47] | 0.495166 | 0.01893114 | 1.6% | 1.64 [1.58, 1.70] |
| DETAIL 2004 [48] | 0.25056997 | 0.50404865 | 0.7% | 1.28 [0.48, 3.45] |
| DEVOTE 2017 [49] | 0.444321 | 0.0266499 | 1.6% | 1.56 [1.48, 1.64] |
| DIRECT-Protect2 2008 [50] | 0.226533 | 0.11781248 | 1.5% | 1.25 [1.00, 1.58] |
| DUAL V 2016 [51] | 0.21133466 | 0.18769887 | 1.3% | 1.24 [0.86, 1.78] |
| DURATION-1 2008 [52] | 0.34532121 | 0.14047221 | 1.4% | 1.41 [1.07, 1.86] |
| DURATION 2 2010 [53] | -0.4782629 | 0.09512285 | 1.5% | 0.62 [0.51, 0.75] |
| DURATION-3 2010 [54] | 2.28186637 | 0.12500214 | 1.5% | 9.79 [7.67, 12.51] |
| DURATION-6 2013 [55] | 2.2763453 | 0.08750506 | 1.5% | 9.74 [8.21, 11.56] |
| ELIXA 2015 [56] | 0.441584 | 0.02981229 | 1.6% | 1.56 [1.47, 1.65] |
| EMPA-REG OUTCOME 2015 [57] | -0.05656 | 0.02669347 | 1.6% | 0.95 [0.90, 1.00] |
| EUREXA 2012 [58] | 0.17791847 | 0.11474651 | 1.5% | 1.19 [0.95, 1.50] |
| EXAMINE 2013 [59] | 0.404381 | 0.03058806 | 1.6% | 1.50 [1.41, 1.59] |
| EXSCEL 2017 [60] | -0.011 | 0.01921066 | 1.6% | 0.99 [0.95, 1.03] |
| Frias et al 2018 [61] | 0.501435 | 0.14080336 | 1.4% | 1.65 [1.25, 2.18] |
| Gallwitz et al 2012 [62] | 2.30479696 | 0.07119336 | 1.6% | 10.02 [8.72, 11.52] |
| Harmony Outcomes 2018 [63] | 0.559583 | 0.02867699 | 1.6% | 1.75 [1.65, 1.85] |
| Heine et al 2005 [64] | 2.24549926 | 0.10699084 | 1.5% | 9.45 [7.66, 11.65] |
| INTERVAL 2013 [65] | 0.23378961 | 0.33886366 | 1.0% | 1.26 [0.65, 2.45] |
| IRMA-2 2001 [66] | 2.43932983 | 0.25346037 | 1.2% | 11.47 [6.98, 18.84] |
| LEAD-3 Mono 2009 [67] | 0.47724932 | 0.08860155 | 1.5% | 1.61 [1.35, 1.92] |
| LEAD-6 2009 [68] | 0.40359079 | 0.16930212 | 1.4% | 1.50 [1.07, 2.09] |
| LEADER 2016 [69] | 0.468826 | 0.02477695 | 1.6% | 1.60 [1.52, 1.68] |
| Lewis et al 2001 [70] | 2.14413288 | 0.05403071 | 1.6% | 8.53 [7.68, 9.49] |
| PERISCOPE 2008 [71] | 0.524753 | 0.11158527 | 1.5% | 1.69 [1.36, 2.10] |
| PIONEER 3 2019 [72] | 0.38172048 | 0.05106015 | 1.6% | 1.46 [1.33, 1.62] |
| PIONEER 4 2019 [73] | 0.40901919 | 0.08446961 | 1.5% | 1.51 [1.28, 1.78] |
| PIONEER 6 2019 [74] | 0.39887271 | 0.03958896 | 1.6% | 1.49 [1.38, 1.61] |
| PROactive 2005 [75] | 0.25247107 | 0.11707759 | 1.5% | 1.29 [1.02, 1.62] |
| Ray et al 2020 [76] | 0.134703 | 0.06177704 | 1.6% | 1.14 [1.01, 1.29] |
| RECORD 2009 [77] | 0.25584688 | 0.14512291 | 1.4% | 1.29 [0.97, 1.72] |
| RENAAL 2001 [78] | -0.12640234 | 0.05143634 | 1.6% | 0.88 [0.80, 0.97] |
| REWIND 2019 [79] | -0.01746 | 0.02344174 | 1.6% | 0.98 [0.94, 1.03] |
| RIO-Diabetes 2006 [80] | 2.34484455 | 0.0970279 | 1.5% | 10.43 [8.63, 12.62] |
| ROADMAP 2011 [81] | 0.26658692 | 1.41429306 | 0.1% | 1.31 [0.08, 20.87] |
| Rosenstock et al 2010 [82] | 2.07342074 | 0.08586737 | 1.5% | 7.95 [6.72, 9.41] |
| SAVOR-TIMI 53 2013 [83] | 0.43917315 | 0.01803878 | 1.6% | 1.55 [1.50, 1.61] |
| SCALE 2015 [84] | 2.28450142 | 0.09225312 | 1.5% | 9.82 [8.20, 11.77] |
| SONAR 2019 [85] | 0.17082913 | 0.03340333 | 1.6% | 1.19 [1.11, 1.27] |
| TECOS 2015 [86] | 0.336191 | 0.01767953 | 1.6% | 1.40 [1.35, 1.45] |
| THEMIS 2019 [87] | 0.38494 | 0.0159387 | 1.6% | 1.47 [1.42, 1.52] |
| TREAT 2009 [88] | 0.27194411 | 0.0327155 | 1.6% | 1.31 [1.23, 1.40] |
| VERIFY 2019 [89] | -0.23055889 | 0.04579523 | 1.6% | 0.79 [0.73, 0.87] |
| VERTIS CV 2020 [90] | 0.59368071 | 0.03364755 | 1.6% | 1.81 [1.70, 1.93] |
| Zinman et al 2007 [91] | 0.54574884 | 0.17732455 | 1.4% | 1.73 [1.22, 2.44] |
| Zinman et al 2011 [92] | 1.32227359 | 0.13714422 | 1.4% | 3.75 [2.87, 4.91] |
| **Total (95% CI)** | | | **100.0%** | **1.86 [1.67, 2.06]** |

Heterogeneity: Tau² = 0.18; Chi² = 7350.61, df = 67 (P < 0.00001); I² = 99%

Test for overall effect: Z = 11.48 (P < 0.00001)

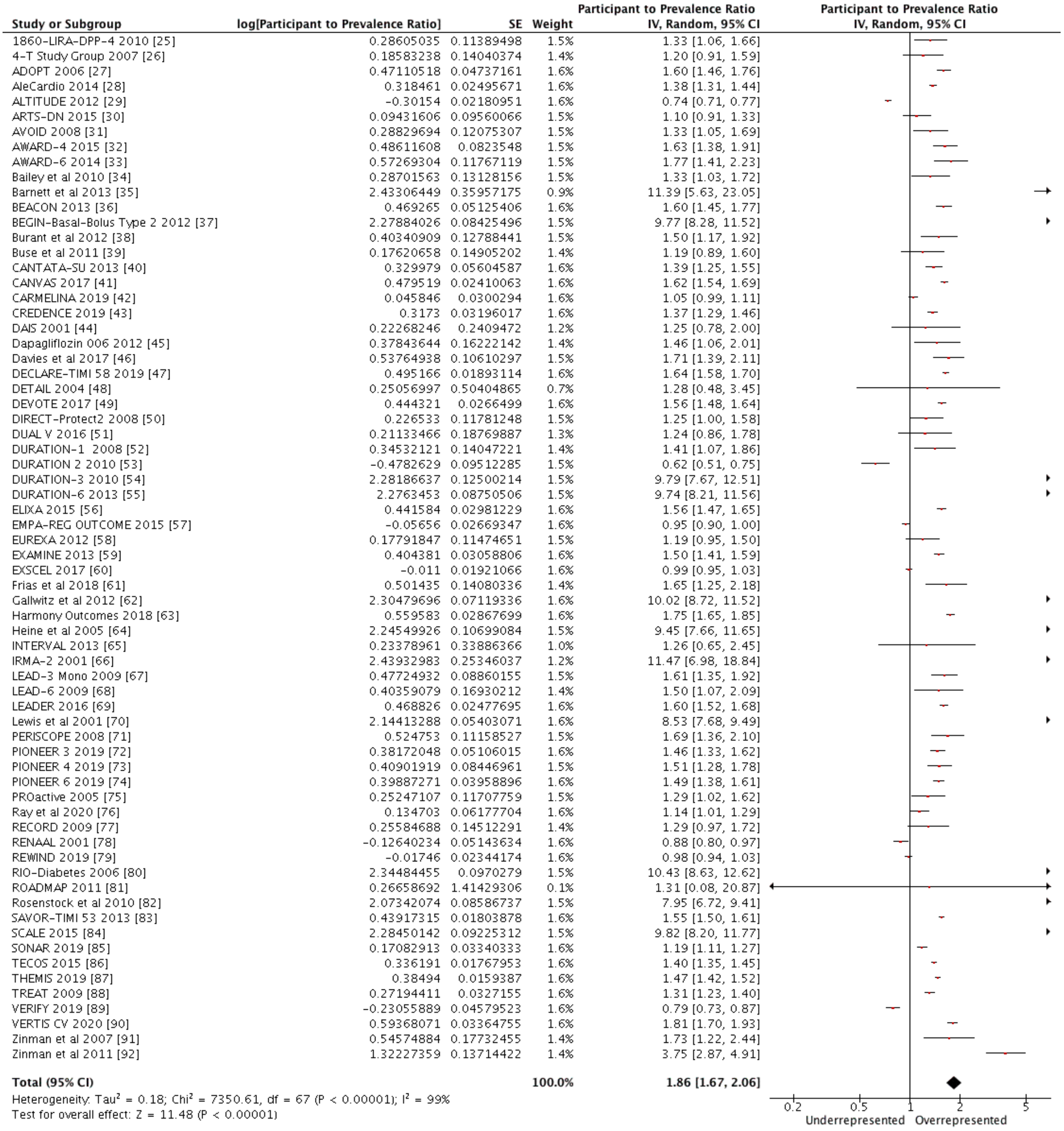

**ESM Fig. 4b.** Sensitivity Analysis 85/15 racialised/white – Racialised PPR Industry Trials.

| Study or Subgroup | log[Participant to Prevalence Ratio] | SE | Weight | Participant to Prevalence Ratio IV, Random, 95% CI |
|---|---|---|---|---|
| 1860-LIRA-DPP-4 2010 [25] | −0.95936968 | 0.11389498 | 1.5% | 0.38 [0.31, 0.48] |
| 4-T Study Group 2007 [26] | −1.10308987 | 0.14040374 | 1.5% | 0.33 [0.25, 0.44] |
| ADOPT 2006 [27] | −1.35263502 | 0.04737161 | 1.6% | 0.26 [0.24, 0.28] |
| AleCardio 2014 [28] | −0.43547148 | 0.02495671 | 1.6% | 0.65 [0.62, 0.68] |
| ALTITUDE 2012 [29] | 0.616105 | 0.02180951 | 1.6% | 1.85 [1.77, 1.93] |
| ARTS-DN 2015 [30] | −0.39096762 | 0.09560066 | 1.5% | 0.68 [0.56, 0.82] |
| AVOID 2008 [31] | −0.97403276 | 0.12075307 | 1.5% | 0.38 [0.30, 0.48] |
| AWARD-4 2015 [32] | −0.88993082 | 0.0823548 | 1.5% | 0.41 [0.35, 0.48] |
| AWARD-6 2014 [33] | −1.30102717 | 0.11767119 | 1.5% | 0.27 [0.22, 0.34] |
| Bailey et al 2010 [34] | −1.03480078 | 0.13128156 | 1.5% | 0.36 [0.27, 0.46] |
| Barnett et al 2013 [35] | −3.31668371 | 0.35957175 | 1.1% | 0.04 [0.02, 0.07] |
| BEACON 2013 [36] | −0.82951 | 0.05125406 | 1.5% | 0.44 [0.39, 0.48] |
| BEGIN-Basal-Bolus Type 2 2012 [37] | −1.67525299 | 0.08425496 | 1.5% | 0.19 [0.16, 0.22] |
| Burant et al 2012 [38] | −0.94742456 | 0.127884 | 1.5% | 0.39 [0.30, 0.50] |
| Buse et al 2011 [39] | −0.44460406 | 0.14905202 | 1.5% | 0.64 [0.48, 0.86] |
| CANTATA-SU 2013 [40] | −0.45892 | 0.05604587 | 1.5% | 0.63 [0.57, 0.71] |
| CANVAS 2017 [41] | −0.86572 | 0.02410063 | 1.6% | 0.42 [0.40, 0.44] |
| CARMELINA 2019 [42] | −0.16663 | 0.0300294 | 1.6% | 0.85 [0.80, 0.90] |
| CREDENCE 2019 [43] | −0.43315 | 0.03196017 | 1.6% | 0.65 [0.61, 0.69] |
| DAIS 2001 [44] | −1.69308864 | 0.2409472 | 1.3% | 0.18 [0.11, 0.29] |
| Dapagliflozin 006 2012 [45] | −1.94395129 | 0.16222142 | 1.4% | 0.14 [0.10, 0.20] |
| Davies et al 2017 [46] | −1.10947336 | 0.10610297 | 1.5% | 0.33 [0.27, 0.41] |
| DECLARE-TIMI 58 2019 [47] | −0.9244 | 0.01893114 | 1.6% | 0.40 [0.38, 0.41] |
| DETAIL 2004 [48] | −2.68314552 | 0.50404865 | 0.8% | 0.07 [0.03, 0.18] |
| DEVOTE 2017 [49] | −0.74794 | 0.0266499 | 1.6% | 0.47 [0.45, 0.50] |
| DIRECT-Protect2 2008 [50] | −1.78273 | 0.11781248 | 1.5% | 0.17 [0.13, 0.21] |
| DUAL V 2016 [51] | −1.46934682 | 0.18769887 | 1.4% | 0.23 [0.16, 0.33] |
| DURATION-1 2008 [52] | −0.70963839 | 0.14047221 | 1.5% | 0.49 [0.37, 0.65] |
| DURATION 2 2010 [53] | 0.38415789 | 0.09512285 | 1.5% | 1.47 [1.22, 1.77] |
| DURATION-3 2010 [54] | −1.69001571 | 0.12500214 | 1.5% | 0.18 [0.14, 0.24] |
| DURATION-6 2013 [55] | −1.66327618 | 0.08750506 | 1.5% | 0.19 [0.16, 0.22] |
| ELIXA 2015 [56] | −0.73949 | 0.02981229 | 1.6% | 0.48 [0.45, 0.51] |
| EMPA-REG OUTCOME 2015 [57] | 0.16543 | 0.02669347 | 1.6% | 1.18 [1.12, 1.24] |
| EUREXA 2012 [58] | −1.01362501 | 0.11474651 | 1.5% | 0.36 [0.29, 0.45] |
| EXAMINE 2013 [59] | −0.63333 | 0.03058806 | 1.6% | 0.53 [0.50, 0.56] |
| EXSCEL 2017 [60] | 0.035167 | 0.01921066 | 1.6% | 1.04 [1.00, 1.08] |
| Frias et al 2018 [61] | −0.94919 | 0.14080336 | 1.5% | 0.39 [0.29, 0.51] |
| Gallwitz et al 2012 [62] | −1.81124611 | 0.07119336 | 1.5% | 0.16 [0.14, 0.19] |
| Harmony Outcomes 2018 [63] | −1.2242 | 0.02867699 | 1.6% | 0.29 [0.28, 0.31] |
| Heine et al 2005 [64] | −1.52807888 | 0.10699084 | 1.5% | 0.22 [0.18, 0.27] |
| INTERVAL 2013 [65] | −1.9783755 | 0.33886366 | 1.1% | 0.14 [0.07, 0.27] |
| IRMA-2 2001 [66] | −3.51886214 | 0.25346037 | 1.3% | 0.03 [0.02, 0.05] |
| LEAD-3 Mono 2009 [67] | −0.85755777 | 0.08860155 | 1.5% | 0.42 [0.36, 0.50] |
| LEAD-6 2009 [68] | −1.55240627 | 0.16930212 | 1.4% | 0.21 [0.15, 0.30] |
| LEADER 2016 [69] | −0.82799935 | 0.02477695 | 1.6% | 0.44 [0.42, 0.46] |
| Lewis et al 2001 [70] | −1.19940129 | 0.05403071 | 1.5% | 0.30 [0.27, 0.34] |
| PERISCOPE 2008 [71] | −1.04872 | 0.11158527 | 1.5% | 0.35 [0.28, 0.44] |
| PIONEER 3 2019 [72] | −0.57549323 | 0.05106015 | 1.5% | 0.56 [0.51, 0.62] |
| PIONEER 4 2019 [73] | −0.64575943 | 0.08446961 | 1.5% | 0.52 [0.44, 0.62] |
| PIONEER 6 2019 [74] | −0.6188366 | 0.03958896 | 1.6% | 0.54 [0.50, 0.58] |
| PROactive 2005 [75] | −2.8076089 | 0.11707759 | 1.5% | 0.06 [0.05, 0.08] |
| Ray et al 2020 [76] | −0.63823 | 0.06177704 | 1.5% | 0.53 [0.47, 0.60] |
| RECORD 2009 [77] | −3.07676295 | 0.14512291 | 1.5% | 0.05 [0.03, 0.06] |
| RENAAL 2001 [78] | 0.13654033 | 0.05143634 | 1.5% | 1.15 [1.04, 1.27] |
| REWIND 2019 [79] | 0.056527 | 0.02344174 | 1.6% | 1.06 [1.01, 1.11] |
| RIO-Diabetes 2006 [80] | −2.07560874 | 0.0970279 | 1.5% | 0.13 [0.10, 0.15] |
| ROADMAP 2011 [81] | −7.64122356 | 1.41429306 | 0.2% | 0.00 [0.00, 0.01] |
| Rosenstock et al 2010 [82] | −1.03451187 | 0.08586737 | 1.5% | 0.36 [0.30, 0.42] |
| SAVOR-TIMI 53 2013 [83] | −0.73213606 | 0.01803878 | 1.6% | 0.48 [0.46, 0.50] |
| SCALE 2015 [84] | −1.70308779 | 0.09225312 | 1.5% | 0.18 [0.15, 0.22] |
| SONAR 2019 [85] | −0.19282598 | 0.03340333 | 1.6% | 0.82 [0.77, 0.88] |
| TECOS 2015 [86] | −0.47192 | 0.01767953 | 1.6% | 0.62 [0.60, 0.65] |
| THEMIS 2019 [87] | −0.58343 | 0.0159387 | 1.6% | 0.56 [0.54, 0.58] |
| TREAT 2009 [88] | −0.34843096 | 0.0327155 | 1.6% | 0.71 [0.66, 0.75] |
| VERIFY 2019 [89] | 0.51502772 | 0.04579523 | 1.6% | 1.67 [1.53, 1.83] |
| VERTIS CV 2020 [90] | −1.4403285 | 0.03364755 | 1.6% | 0.24 [0.22, 0.25] |
| Zinman et al 2007 [91] | −1.15003467 | 0.17732455 | 1.4% | 0.32 [0.22, 0.45] |
| Zinman et al 2011 [92] | −0.2945927 | 0.13714422 | 1.5% | 0.74 [0.57, 0.97] |
| **Total (95% CI)** | | | **100.0%** | **0.37 [0.32, 0.42]** |

Heterogeneity: $Tau^2 = 0.29$; $Chi^2 = 11646.30$, $df = 67$ ($P < 0.00001$); $I^2 = 99\%$
Test for overall effect: $Z = 15.00$ ($P < 0.00001$)

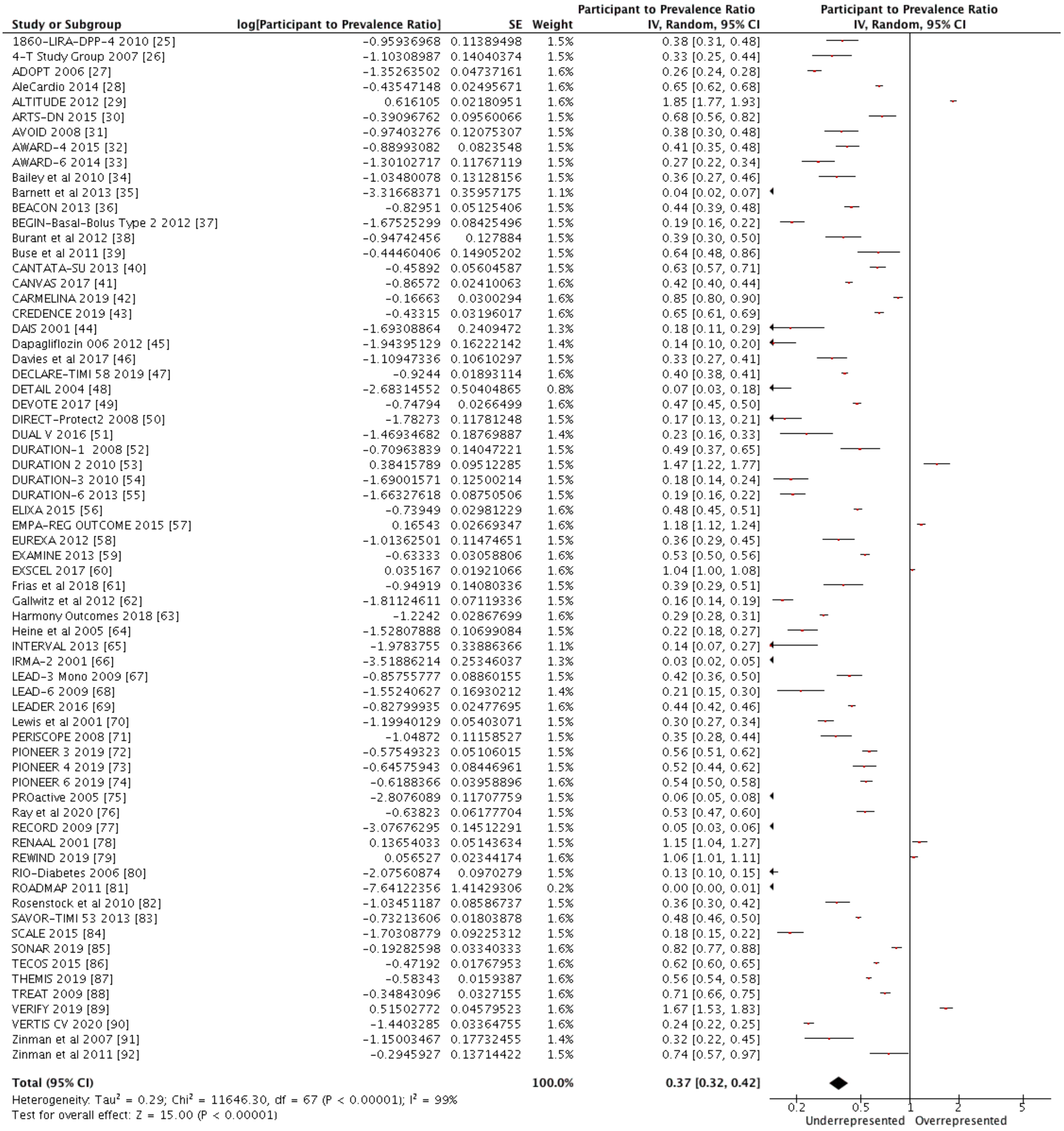

Variation 4: Worldwide population proportion is 80% racialised, 20% white

**ESM Fig. 5a.** Sensitivity Analysis 80/20 racialised/white – White PPR Industry Trials.

| Study or Subgroup | log[Participant to Prevalence Ratio] | SE | Weight | Participant to Prevalence Ratio IV, Random, 95% CI |
|---|---|---|---|---|
| 1860-LIRA-DPP-4 2010 [25] | 0.28605035 | 0.11389498 | 1.5% | 1.33 [1.06, 1.66] |
| 4-T Study Group 2007 [26] | 0.18583238 | 0.14040374 | 1.4% | 1.20 [0.91, 1.59] |
| ADOPT 2006 [27] | 0.47110518 | 0.04737161 | 1.6% | 1.60 [1.46, 1.76] |
| AleCardio 2014 [28] | 0.318461 | 0.02495671 | 1.6% | 1.38 [1.31, 1.44] |
| ALTITUDE 2012 [29] | -0.30154 | 0.02180951 | 1.6% | 0.74 [0.71, 0.77] |
| ARTS-DN 2015 [30] | 0.09431606 | 0.09560066 | 1.5% | 1.10 [0.91, 1.33] |
| AVOID 2008 [31] | 0.28829694 | 0.12075307 | 1.5% | 1.33 [1.05, 1.69] |
| AWARD-4 2015 [32] | 0.48611608 | 0.0823548 | 1.6% | 1.63 [1.38, 1.91] |
| AWARD-6 2014 [33] | 0.57269304 | 0.11767119 | 1.5% | 1.77 [1.41, 2.23] |
| Bailey et al 2010 [34] | 0.28701563 | 0.13128156 | 1.4% | 1.33 [1.03, 1.72] |
| Barnett et al 2013 [35] | 2.11950297 | 0.35957175 | 0.9% | 8.33 [4.12, 16.85] |
| BEACON 2013 [36] | 0.469265 | 0.05125406 | 1.6% | 1.60 [1.45, 1.77] |
| BEGIN-Basal-Bolus Type 2 2012 [37] | 1.96527874 | 0.08425496 | 1.5% | 7.14 [6.05, 8.42] |
| Burant et al 2012 [38] | 0.40340909 | 0.12788441 | 1.5% | 1.50 [1.17, 1.92] |
| Buse et al 2011 [39] | 0.17620658 | 0.14905202 | 1.4% | 1.19 [0.89, 1.60] |
| CANTATA-SU 2013 [40] | 0.329979 | 0.05604587 | 1.6% | 1.39 [1.25, 1.55] |
| CANVAS 2017 [41] | 0.479519 | 0.02410063 | 1.6% | 1.62 [1.54, 1.69] |
| CARMELINA 2019 [42] | 0.045846 | 0.0300294 | 1.6% | 1.05 [0.99, 1.11] |
| CREDENCE 2019 [43] | 0.3173 | 0.03196017 | 1.6% | 1.37 [1.29, 1.46] |
| DAIS 2001 [44] | 0.22268246 | 0.2409472 | 1.2% | 1.25 [0.78, 2.00] |
| Dapagliflozin 006 2012 [45] | 0.37843644 | 0.16222142 | 1.4% | 1.46 [1.06, 2.01] |
| Davies et al 2017 [46] | 0.53764938 | 0.10610297 | 1.5% | 1.71 [1.39, 2.11] |
| DECLARE-TIMI 58 2019 [47] | 0.495166 | 0.01893114 | 1.6% | 1.64 [1.58, 1.70] |
| DETAIL 2004 [48] | 0.25056997 | 0.50404865 | 0.6% | 1.28 [0.48, 3.45] |
| DEVOTE 2017 [49] | 0.444321 | 0.0266499 | 1.6% | 1.56 [1.48, 1.64] |
| DIRECT-Protect2 2008 [50] | 0.226533 | 0.11781248 | 1.5% | 1.25 [1.00, 1.58] |
| DUAL V 2016 [51] | 0.21133466 | 0.18769887 | 1.3% | 1.24 [0.86, 1.78] |
| DURATION-1 2008 [52] | 0.34532121 | 0.14047221 | 1.4% | 1.41 [1.07, 1.86] |
| DURATION 2 2010 [53] | -0.4782629 | 0.09512285 | 1.5% | 0.62 [0.51, 0.75] |
| DURATION-3 2010 [54] | 1.96830485 | 0.12500214 | 1.5% | 7.16 [5.60, 9.15] |
| DURATION-6 2013 [55] | 1.96278378 | 0.08750506 | 1.5% | 7.12 [6.00, 8.45] |
| ELIXA 2015 [56] | 0.441584 | 0.02981229 | 1.6% | 1.56 [1.47, 1.65] |
| EMPA-REG OUTCOME 2015 [57] | -0.05656 | 0.02669347 | 1.6% | 0.95 [0.90, 1.00] |
| EUREXA 2012 [58] | 0.17791847 | 0.11474651 | 1.5% | 1.19 [0.95, 1.50] |
| EXAMINE 2013 [59] | 0.404381 | 0.03058806 | 1.6% | 1.50 [1.41, 1.59] |
| EXSCEL 2017 [60] | -0.011 | 0.01921066 | 1.6% | 0.99 [0.95, 1.03] |
| Frias et al 2018 [61] | 0.501435 | 0.14080336 | 1.4% | 1.65 [1.25, 2.18] |
| Gallwitz et al 2012 [62] | 1.99123544 | 0.07119336 | 1.6% | 7.32 [6.37, 8.42] |
| Harmony Outcomes 2018 [63] | 0.559583 | 0.02867699 | 1.6% | 1.75 [1.65, 1.85] |
| Heine et al 2005 [64] | 1.93193774 | 0.10699084 | 1.5% | 6.90 [5.60, 8.51] |
| INTERVAL 2013 [65] | 0.23378961 | 0.33886366 | 0.9% | 1.26 [0.65, 2.45] |
| IRMA-2 2001 [66] | 2.12576831 | 0.25346037 | 1.1% | 8.38 [5.10, 13.77] |
| LEAD-3 Mono 2009 [67] | 0.47724932 | 0.08860155 | 1.5% | 1.61 [1.35, 1.92] |
| LEAD-6 2009 [68] | 0.40359079 | 0.16930212 | 1.4% | 1.50 [1.07, 2.09] |
| LEADER 2016 [69] | 0.468826 | 0.02477695 | 1.6% | 1.60 [1.52, 1.68] |
| Lewis et al 2001 [70] | 1.83057136 | 0.05403071 | 1.6% | 6.24 [5.61, 6.93] |
| PERISCOPE 2008 [71] | 0.524753 | 0.11158527 | 1.5% | 1.69 [1.36, 2.10] |
| PIONEER 3 2019 [72] | 0.38172048 | 0.05106015 | 1.6% | 1.46 [1.33, 1.62] |
| PIONEER 4 2019 [73] | 0.40901919 | 0.08446961 | 1.5% | 1.51 [1.28, 1.78] |
| PIONEER 6 2019 [74] | 0.39887271 | 0.03958896 | 1.6% | 1.49 [1.38, 1.61] |
| PROactive 2005 [75] | 0.25247107 | 0.11707759 | 1.5% | 1.29 [1.02, 1.62] |
| Ray et al 2020 [76] | 0.134703 | 0.06177704 | 1.6% | 1.14 [1.01, 1.29] |
| RECORD 2009 [77] | 0.25584688 | 0.14512291 | 1.4% | 1.29 [0.97, 1.72] |
| RENAAL 2001 [78] | -0.12640234 | 0.05143634 | 1.6% | 0.88 [0.80, 0.97] |
| REWIND 2019 [79] | -0.01746 | 0.02344174 | 1.6% | 0.98 [0.94, 1.03] |
| RIO-Diabetes 2006 [80] | 2.03128303 | 0.0970279 | 1.5% | 7.62 [6.30, 9.22] |
| ROADMAP 2011 [81] | 0.26658692 | 1.41429306 | 0.1% | 1.31 [0.08, 20.87] |
| Rosenstock et al 2010 [82] | 1.75985922 | 0.08586737 | 1.5% | 5.81 [4.91, 6.88] |
| SAVOR-TIMI 53 2013 [83] | 0.43917315 | 0.01803878 | 1.6% | 1.55 [1.50, 1.61] |
| SCALE 2015 [84] | 1.9709399 | 0.09225312 | 1.5% | 7.18 [5.99, 8.60] |
| SONAR 2019 [85] | 0.17082913 | 0.03340333 | 1.6% | 1.19 [1.11, 1.27] |
| TECOS 2015 [86] | 0.336191 | 0.01767953 | 1.6% | 1.40 [1.35, 1.45] |
| THEMIS 2019 [87] | 0.38494 | 0.0159387 | 1.6% | 1.47 [1.42, 1.52] |
| TREAT 2009 [88] | 0.27194411 | 0.0327155 | 1.6% | 1.31 [1.23, 1.40] |
| VERIFY 2019 [89] | -0.23055889 | 0.04579523 | 1.6% | 0.79 [0.73, 0.87] |
| VERTIS CV 2020 [90] | 0.59368071 | 0.03364755 | 1.6% | 1.81 [1.70, 1.93] |
| Zinman et al 2007 [91] | 0.54574884 | 0.17732455 | 1.3% | 1.73 [1.22, 2.44] |
| Zinman et al 2011 [92] | 1.00871207 | 0.13714422 | 1.4% | 2.74 [2.10, 3.59] |
| **Total (95% CI)** | | | **100.0%** | **1.75 [1.60, 1.93]** |

Heterogeneity: $Tau^2 = 0.14$; $Chi^2 = 5858.51$, $df = 67$ ($P < 0.00001$); $I^2 = 99\%$
Test for overall effect: $Z = 11.62$ ($P < 0.00001$)

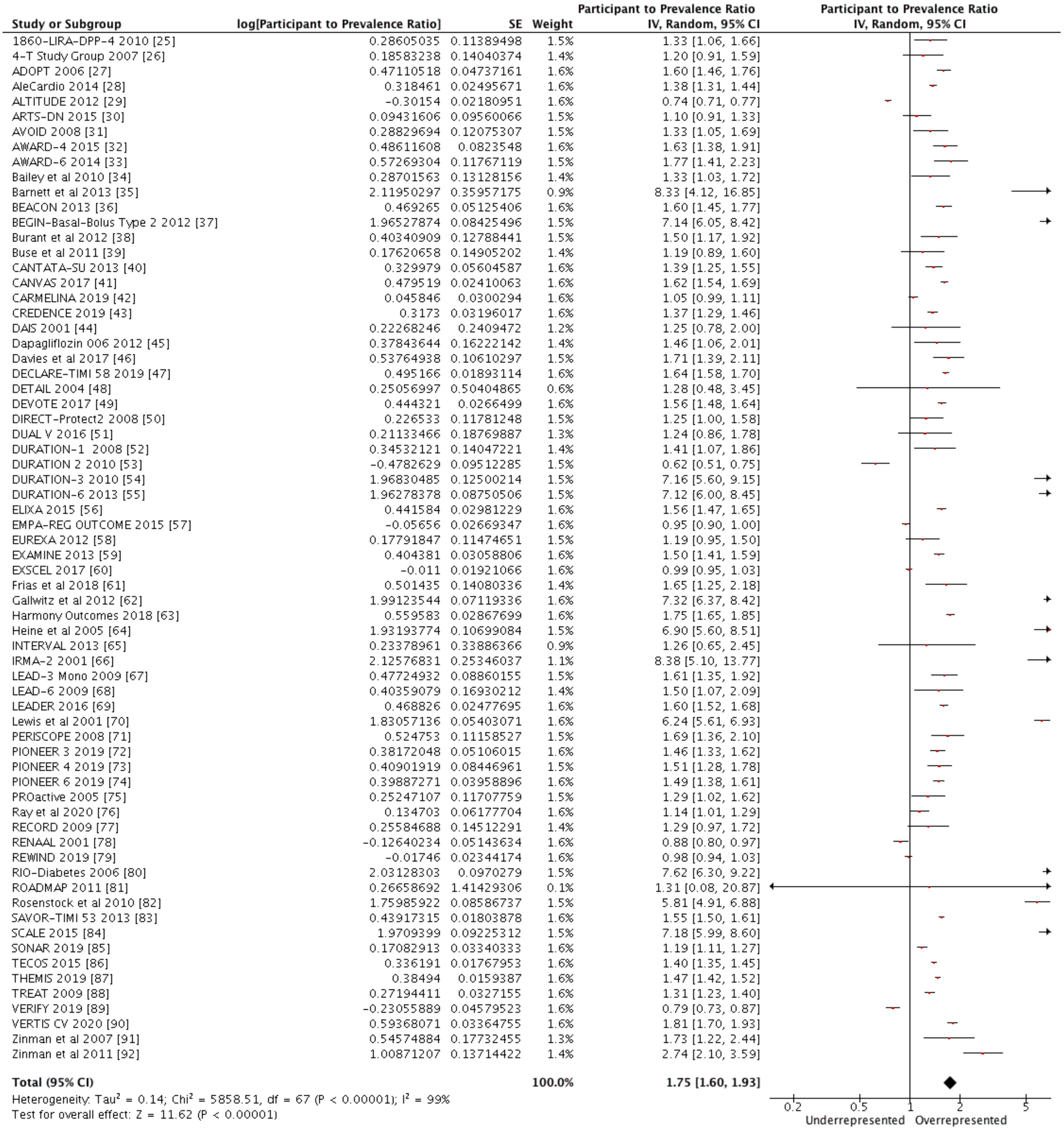

**ESM Fig. 5b.** Sensitivity Analysis 80/20 racialised/white – Racialised PPR Industry Trials.

| Study or Subgroup | log[Participant to Prevalence Ratio] | SE | Weight | Participant to Prevalence Ratio IV, Random, 95% CI |
|---|---|---|---|---|
| 1860-LIRA-DPP-4 2010 [25] | -0.95936968 | 0.11389498 | 1.5% | 0.38 [0.31, 0.48] |
| 4-T Study Group 2007 [26] | -1.10308987 | 0.14040374 | 1.5% | 0.33 [0.25, 0.44] |
| ADOPT 2006 [27] | -1.35263502 | 0.04737161 | 1.6% | 0.26 [0.24, 0.28] |
| AleCardio 2014 [28] | -0.43547148 | 0.02495671 | 1.6% | 0.65 [0.62, 0.68] |
| ALTITUDE 2012 [29] | 0.616105 | 0.02180951 | 1.6% | 1.85 [1.77, 1.93] |
| ARTS-DN 2015 [30] | -0.39096762 | 0.09560066 | 1.5% | 0.68 [0.56, 0.82] |
| AVOID 2008 [31] | -0.97403276 | 0.12075307 | 1.5% | 0.38 [0.30, 0.48] |
| AWARD-4 2015 [32] | -0.88993082 | 0.0823548 | 1.5% | 0.41 [0.35, 0.48] |
| AWARD-6 2014 [33] | -1.30102717 | 0.11767119 | 1.5% | 0.27 [0.22, 0.34] |
| Bailey et al 2010 [34] | -1.03480078 | 0.13128156 | 1.5% | 0.36 [0.27, 0.46] |
| Barnett et al 2013 [35] | -3.28193854 | 0.35957175 | 1.1% | 0.04 [0.02, 0.08] |
| BEACON 2013 [36] | -0.82951 | 0.05125406 | 1.5% | 0.44 [0.39, 0.48] |
| BEGIN-Basal-Bolus Type 2 2012 [37] | -1.64050782 | 0.08425496 | 1.5% | 0.19 [0.16, 0.23] |
| Burant et al 2012 [38] | -0.94742456 | 0.127884 | 1.5% | 0.39 [0.30, 0.50] |
| Buse et al 2011 [39] | -0.44460406 | 0.14905202 | 1.5% | 0.64 [0.48, 0.86] |
| CANTATA-SU 2013 [40] | -0.45892 | 0.05604587 | 1.5% | 0.63 [0.57, 0.71] |
| CANVAS 2017 [41] | -0.86572 | 0.02410063 | 1.6% | 0.42 [0.40, 0.44] |
| CARMELINA 2019 [42] | -0.16663 | 0.0300294 | 1.6% | 0.85 [0.80, 0.90] |
| CREDENCE 2019 [43] | -0.43315 | 0.03196017 | 1.6% | 0.65 [0.61, 0.69] |
| DAIS 2001 [44] | -1.69308864 | 0.2409472 | 1.3% | 0.18 [0.11, 0.29] |
| Dapagliflozin 006 2012 [45] | -1.94395129 | 0.16222142 | 1.4% | 0.14 [0.10, 0.20] |
| Davies et al 2017 [46] | -1.10947336 | 0.10610297 | 1.5% | 0.33 [0.27, 0.41] |
| DECLARE-TIMI 58 2019 [47] | -0.9244 | 0.01893114 | 1.6% | 0.40 [0.38, 0.41] |
| DETAIL 2004 [48] | -2.68314552 | 0.50404865 | 0.8% | 0.07 [0.03, 0.18] |
| DEVOTE 2017 [49] | -0.74794 | 0.0266499 | 1.6% | 0.47 [0.45, 0.50] |
| DIRECT-Protect2 2008 [50] | -1.78273 | 0.11781248 | 1.5% | 0.17 [0.13, 0.21] |
| DUAL V 2016 [51] | -1.46934682 | 0.18769887 | 1.4% | 0.23 [0.16, 0.33] |
| DURATION-1 2008 [52] | -0.70963839 | 0.14047221 | 1.5% | 0.49 [0.37, 0.65] |
| DURATION 2 2010 [53] | 0.38415789 | 0.09512285 | 1.5% | 1.47 [1.22, 1.77] |
| DURATION-3 2010 [54] | -1.65527053 | 0.12500214 | 1.5% | 0.19 [0.15, 0.24] |
| DURATION-6 2013 [55] | -1.62853101 | 0.08750506 | 1.5% | 0.20 [0.17, 0.23] |
| ELIXA 2015 [56] | -0.73949 | 0.02981229 | 1.6% | 0.48 [0.45, 0.51] |
| EMPA-REG OUTCOME 2015 [57] | 0.16543 | 0.02669347 | 1.6% | 1.18 [1.12, 1.24] |
| EUREXA 2012 [58] | -1.01362501 | 0.11474651 | 1.5% | 0.36 [0.29, 0.45] |
| EXAMINE 2013 [59] | -0.63333 | 0.03058806 | 1.6% | 0.53 [0.50, 0.56] |
| EXSCEL 2017 [60] | 0.035167 | 0.01921066 | 1.6% | 1.04 [1.00, 1.08] |
| Frias et al 2018 [61] | -0.94919 | 0.14080336 | 1.5% | 0.39 [0.29, 0.51] |
| Gallwitz et al 2012 [62] | -1.77650094 | 0.07119336 | 1.5% | 0.17 [0.15, 0.19] |
| Harmony Outcomes 2018 [63] | -1.2242 | 0.02867699 | 1.6% | 0.29 [0.28, 0.31] |
| Heine et al 2005 [64] | -1.49333371 | 0.10699084 | 1.5% | 0.22 [0.18, 0.28] |
| INTERVAL 2013 [65] | -1.9783755 | 0.33886366 | 1.1% | 0.14 [0.07, 0.27] |
| IRMA-2 2001 [66] | -3.48411696 | 0.25346037 | 1.3% | 0.03 [0.02, 0.05] |
| LEAD-3 Mono 2009 [67] | -0.85755777 | 0.08860155 | 1.5% | 0.42 [0.36, 0.50] |
| LEAD-6 2009 [68] | -1.55240627 | 0.16930212 | 1.4% | 0.21 [0.15, 0.30] |
| LEADER 2016 [69] | -0.82799935 | 0.02477695 | 1.6% | 0.44 [0.42, 0.46] |
| Lewis et al 2001 [70] | -1.16465612 | 0.05403071 | 1.5% | 0.31 [0.28, 0.35] |
| PERISCOPE 2008 [71] | -1.04872 | 0.11158527 | 1.5% | 0.35 [0.28, 0.44] |
| PIONEER 3 2019 [72] | -0.57549323 | 0.05106015 | 1.5% | 0.56 [0.51, 0.62] |
| PIONEER 4 2019 [73] | -0.64575943 | 0.08446961 | 1.5% | 0.52 [0.44, 0.62] |
| PIONEER 6 2019 [74] | -0.6188366 | 0.03958896 | 1.6% | 0.54 [0.50, 0.58] |
| PROactive 2005 [75] | -2.8076089 | 0.11707759 | 1.5% | 0.06 [0.05, 0.08] |
| Ray et al 2020 [76] | -0.63823 | 0.06177704 | 1.5% | 0.53 [0.47, 0.60] |
| RECORD 2009 [77] | -3.07676295 | 0.14512291 | 1.5% | 0.05 [0.03, 0.06] |
| RENAAL 2001 [78] | 0.13654033 | 0.05143634 | 1.5% | 1.15 [1.04, 1.27] |
| REWIND 2019 [79] | 0.056527 | 0.02344174 | 1.6% | 1.06 [1.01, 1.11] |
| RIO-Diabetes 2006 [80] | -2.04086357 | 0.0970279 | 1.5% | 0.13 [0.11, 0.16] |
| ROADMAP 2011 [81] | -7.64122356 | 1.41429306 | 0.2% | 0.00 [0.00, 0.01] |
| Rosenstock et al 2010 [82] | -0.9997667 | 0.08586737 | 1.5% | 0.37 [0.31, 0.44] |
| SAVOR-TIMI 53 2013 [83] | -0.73213606 | 0.01803878 | 1.6% | 0.48 [0.46, 0.50] |
| SCALE 2015 [84] | -1.66834262 | 0.09225312 | 1.5% | 0.19 [0.16, 0.23] |
| SONAR 2019 [85] | -0.19282598 | 0.03340333 | 1.6% | 0.82 [0.77, 0.88] |
| TECOS 2015 [86] | -0.47192 | 0.01767953 | 1.6% | 0.62 [0.60, 0.65] |
| THEMIS 2019 [87] | -0.58343 | 0.0159387 | 1.6% | 0.56 [0.54, 0.58] |
| TREAT 2009 [88] | -0.34843096 | 0.0327155 | 1.6% | 0.71 [0.66, 0.75] |
| VERIFY 2019 [89] | 0.51502772 | 0.04579523 | 1.6% | 1.67 [1.53, 1.83] |
| VERTIS CV 2020 [90] | -1.4403285 | 0.03364755 | 1.6% | 0.24 [0.22, 0.25] |
| Zinman et al 2007 [91] | -1.15003467 | 0.17732455 | 1.4% | 0.32 [0.22, 0.45] |
| Zinman et al 2011 [92] | -0.25984755 | 0.13714422 | 1.5% | 0.77 [0.59, 1.01] |
| **Total (95% CI)** | | | **100.0%** | **0.37 [0.32, 0.42]** |

Heterogeneity: $Tau^2 = 0.29$; $Chi^2 = 11552.37$, $df = 67$ ($P < 0.00001$); $I^2 = 99\%$

Test for overall effect: $Z = 14.96$ ($P < 0.00001$)

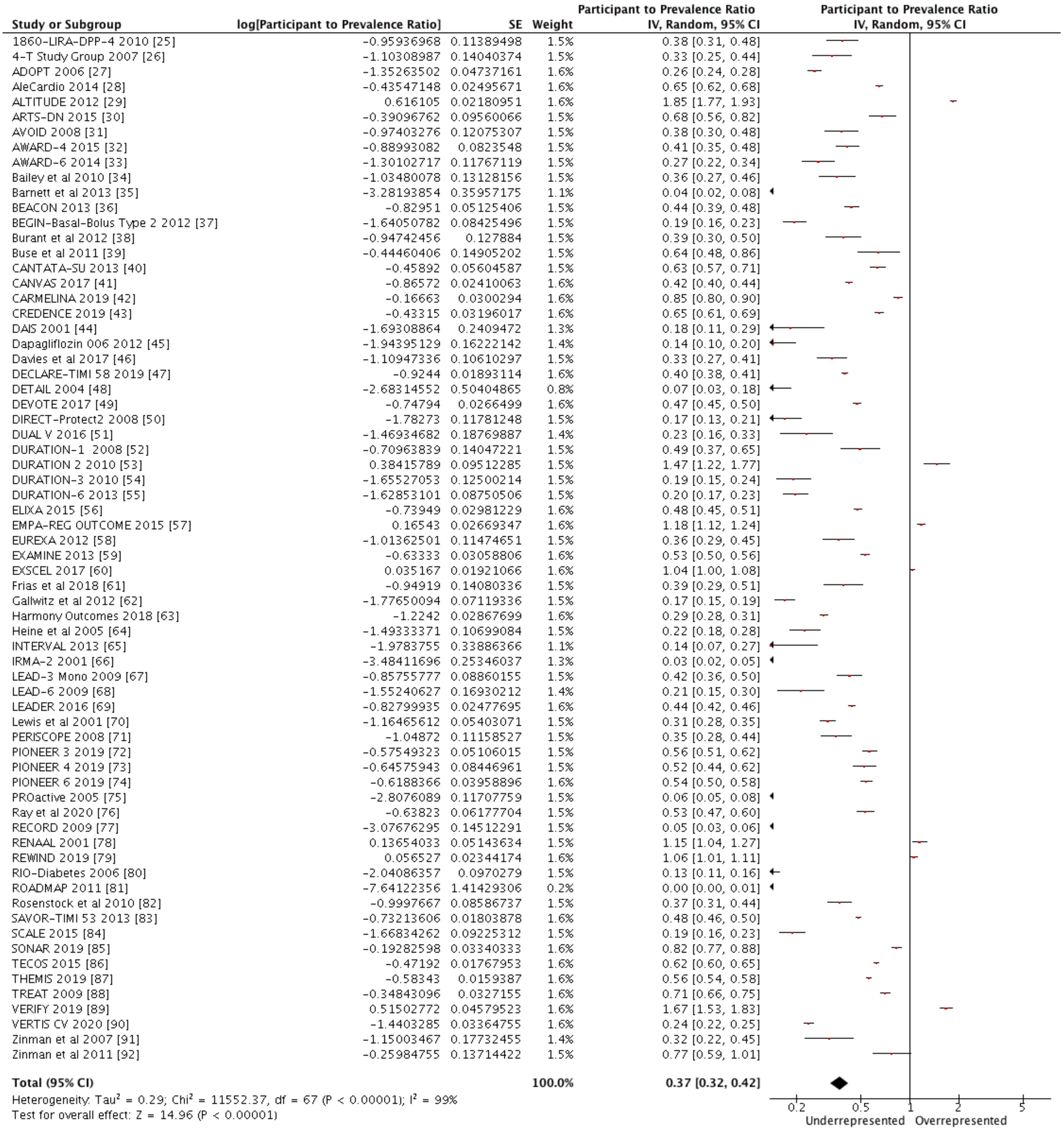

## Appendix 2. Sensitivity analysis including three trials with dual funding

**ESM Fig. 6a.** PPR White – Industry Trials including Dailey et al (2004), Fayfman et al (2019), and Zhu et al (2018).

| Study or Subgroup | log[Participant to Prevalence Ratio] | SE | Weight | Participant to Prevalence Ratio IV, Random, 95% CI |
|---|---|---|---|---|
| 1860-LIRA-DPP-4 2010 [25] | 0.28605035 | 0.11389498 | 1.5% | 1.33 [1.06, 1.66] |
| 4-T Study Group 2007 [26] | 0.18583238 | 0.14040374 | 1.4% | 1.20 [0.91, 1.59] |
| ADOPT 2006 [27] | 0.47110518 | 0.04737161 | 1.5% | 1.60 [1.46, 1.76] |
| AleCardio 2014 [28] | 0.318461 | 0.02495671 | 1.5% | 1.38 [1.31, 1.44] |
| ALTITUDE 2012 [29] | -0.30154 | 0.02180951 | 1.5% | 0.74 [0.71, 0.77] |
| ARTS-DN 2015 [30] | 0.09431606 | 0.09560066 | 1.5% | 1.10 [0.91, 1.33] |
| AVOID 2008 [31] | 0.28829694 | 0.12075307 | 1.4% | 1.33 [1.05, 1.69] |
| AWARD-4 2015 [32] | 0.48611608 | 0.0823548 | 1.5% | 1.63 [1.38, 1.91] |
| AWARD-6 2014 [33] | 0.57269304 | 0.11767119 | 1.4% | 1.77 [1.41, 2.23] |
| Bailey et al 2010 [34] | 0.28701563 | 0.13128156 | 1.4% | 1.33 [1.03, 1.72] |
| Barnett et al 2013 [35] | 2.69824659 | 0.35957175 | 1.0% | 14.85 [7.34, 30.05] |
| BEACON 2013 [36] | 0.469265 | 0.05125406 | 1.5% | 1.60 [1.45, 1.77] |
| BEGIN-Basal-Bolus Type 2 2012 [37] | 2.54402336 | 0.08425496 | 1.5% | 12.73 [10.79, 15.02] |
| Burant et al 2012 [38] | 0.40340909 | 0.12788441 | 1.4% | 1.50 [1.17, 1.92] |
| Buse et al 2011 [39] | 0.17620658 | 0.14905202 | 1.4% | 1.19 [0.89, 1.60] |
| CANTATA-SU 2013 [40] | 0.329979 | 0.05604587 | 1.5% | 1.39 [1.25, 1.55] |
| CANVAS 2017 [41] | 0.479519 | 0.02410063 | 1.5% | 1.62 [1.54, 1.69] |
| CARMELINA 2019 [42] | 0.045846 | 0.0300294 | 1.5% | 1.05 [0.99, 1.11] |
| CREDENCE 2019 [43] | 0.3173 | 0.03196017 | 1.5% | 1.37 [1.29, 1.46] |
| Dailey et al 2004 [94] | 0.23285145 | 0.09565249 | 1.5% | 1.26 [1.05, 1.52] |
| DAIS 2001 [44] | 0.22268246 | 0.2409472 | 1.2% | 1.25 [0.78, 2.00] |
| Dapagliflozin 006 2012 [45] | 0.37843644 | 0.16222142 | 1.4% | 1.46 [1.06, 2.01] |
| Davies et al 2017 [46] | 0.53764938 | 0.10610297 | 1.5% | 1.71 [1.39, 2.11] |
| DECLARE-TIMI 58 2019 [47] | 0.495166 | 0.01893114 | 1.5% | 1.64 [1.58, 1.70] |
| DETAIL 2004 [48] | 0.25056997 | 0.50404865 | 0.7% | 1.28 [0.48, 3.45] |
| DEVOTE 2017 [49] | 0.444321 | 0.0266499 | 1.5% | 1.56 [1.48, 1.64] |
| DIRECT-Protect2 2008 [50] | 0.226533 | 0.11781248 | 1.4% | 1.25 [1.00, 1.58] |
| DUAL V 2016 [51] | 0.21133466 | 0.18769887 | 1.3% | 1.24 [0.86, 1.78] |
| DURATION-1 2008 [52] | 0.34532121 | 0.14047221 | 1.4% | 1.41 [1.07, 1.86] |
| DURATION 2 2010 [53] | -0.4782629 | 0.09512285 | 1.5% | 0.62 [0.51, 0.75] |
| DURATION-3 2010 [54] | 2.54704847 | 0.12500214 | 1.4% | 12.77 [9.99, 16.31] |
| DURATION-6 2013 [55] | 2.5415274 | 0.08750506 | 1.5% | 12.70 [10.70, 15.07] |
| ELIXA 2015 [56] | 0.441584 | 0.02981229 | 1.5% | 1.56 [1.47, 1.65] |
| EMPA-REG OUTCOME 2015 [57] | -0.05656 | 0.02669347 | 1.5% | 0.95 [0.90, 1.00] |
| EUREXA 2012 [58] | 0.17791847 | 0.11474651 | 1.4% | 1.19 [0.95, 1.50] |
| EXAMINE 2013 [59] | 0.404381 | 0.03058806 | 1.5% | 1.50 [1.41, 1.59] |
| EXSCEL 2017 [60] | -0.011 | 0.01921066 | 1.5% | 0.99 [0.95, 1.03] |
| Fayfman et al 2019 [93] | -0.57327549 | 0.18320632 | 1.3% | 0.56 [0.39, 0.81] |
| Frias et al 2018 [61] | 0.501435 | 0.14080336 | 1.4% | 1.65 [1.25, 2.18] |
| Gallwitz et al 2012 [62] | 2.56997906 | 0.07119336 | 1.5% | 13.07 [11.36, 15.02] |
| Harmony Outcomes 2018 [63] | 0.559583 | 0.02867699 | 1.5% | 1.75 [1.65, 1.85] |
| Heine et al 2005 [64] | 2.51068136 | 0.10699084 | 1.5% | 12.31 [9.98, 15.19] |
| INTERVAL 2013 [65] | 0.23378961 | 0.33886366 | 1.0% | 1.26 [0.65, 2.45] |
| IRMA-2 2001 [66] | 2.70451193 | 0.25346037 | 1.2% | 14.95 [9.10, 24.56] |
| LEAD-3 Mono 2009 [67] | 0.47724932 | 0.08860155 | 1.5% | 1.61 [1.35, 1.92] |
| LEAD-6 2009 [68] | 0.40359079 | 0.16930212 | 1.4% | 1.50 [1.07, 2.09] |
| LEADER 2016 [69] | 0.468826 | 0.02477695 | 1.5% | 1.60 [1.52, 1.68] |
| Lewis et al 2001 [70] | 2.40931498 | 0.05403071 | 1.5% | 11.13 [10.01, 12.37] |
| PERISCOPE 2008 [71] | 0.524753 | 0.11158527 | 1.5% | 1.69 [1.36, 2.10] |
| PIONEER 3 2019 [72] | 0.38172048 | 0.05106015 | 1.5% | 1.46 [1.33, 1.62] |
| PIONEER 4 2019 [73] | 0.40901919 | 0.08446961 | 1.5% | 1.51 [1.28, 1.78] |
| PIONEER 6 2019 [74] | 0.39887271 | 0.03958896 | 1.5% | 1.49 [1.38, 1.61] |
| PROactive 2005 [75] | 0.25247107 | 0.11707759 | 1.4% | 1.29 [1.02, 1.62] |
| Ray et al 2020 [76] | 0.134703 | 0.06177704 | 1.5% | 1.14 [1.01, 1.29] |
| RECORD 2009 [77] | 0.25584688 | 0.14512291 | 1.4% | 1.29 [0.97, 1.72] |
| RENAAL 2001 [78] | -0.12640234 | 0.05143634 | 1.5% | 0.88 [0.80, 0.97] |
| REWIND 2019 [79] | -0.01746 | 0.02344174 | 1.5% | 0.98 [0.94, 1.03] |
| RIO-Diabetes 2006 [80] | 2.61002665 | 0.0970279 | 1.5% | 13.60 [11.24, 16.45] |
| ROADMAP 2011 [81] | 0.26658692 | 1.41429306 | 0.2% | 1.31 [0.08, 20.87] |
| Rosenstock et al 2010 [82] | 2.33860284 | 0.08586737 | 1.5% | 10.37 [8.76, 12.27] |
| SAVOR-TIMI 53 2013 [83] | 0.43917315 | 0.01803878 | 1.5% | 1.55 [1.50, 1.61] |
| SCALE 2015 [84] | 2.54968352 | 0.09225312 | 1.5% | 12.80 [10.69, 15.34] |
| SONAR 2019 [85] | 0.17082913 | 0.03340333 | 1.5% | 1.19 [1.11, 1.27] |
| TECOS 2015 [86] | 0.336191 | 0.01767953 | 1.5% | 1.40 [1.35, 1.45] |
| THEMIS 2019 [87] | 0.38494 | 0.0159387 | 1.5% | 1.47 [1.42, 1.52] |
| TREAT 2009 [88] | 0.27194411 | 0.0327155 | 1.5% | 1.31 [1.23, 1.40] |
| VERIFY 2019 [89] | -0.23055889 | 0.04579523 | 1.5% | 0.79 [0.73, 0.87] |
| VERTIS CV 2020 [90] | 0.59368071 | 0.03364755 | 1.5% | 1.81 [1.70, 1.93] |
| Zhu et al 2018 [95] | -2.00168122 | 1.41559937 | 0.2% | 0.14 [0.01, 2.17] |
| Zinman et al 2007 [91] | 0.54574884 | 0.17732455 | 1.3% | 1.73 [1.22, 2.44] |
| Zinman et al 2011 [92] | 1.58745569 | 0.13714422 | 1.4% | 4.89 [3.74, 6.40] |
| **Total (95% CI)** | | | **100.0%** | **1.90 [1.69, 2.12]** |

Heterogeneity: $Tau^2 = 0.22$; $Chi^2 = 8850.02$, df = 70 ($P < 0.00001$); $I^2 = 99\%$
Test for overall effect: Z = 11.05 ($P < 0.00001$)

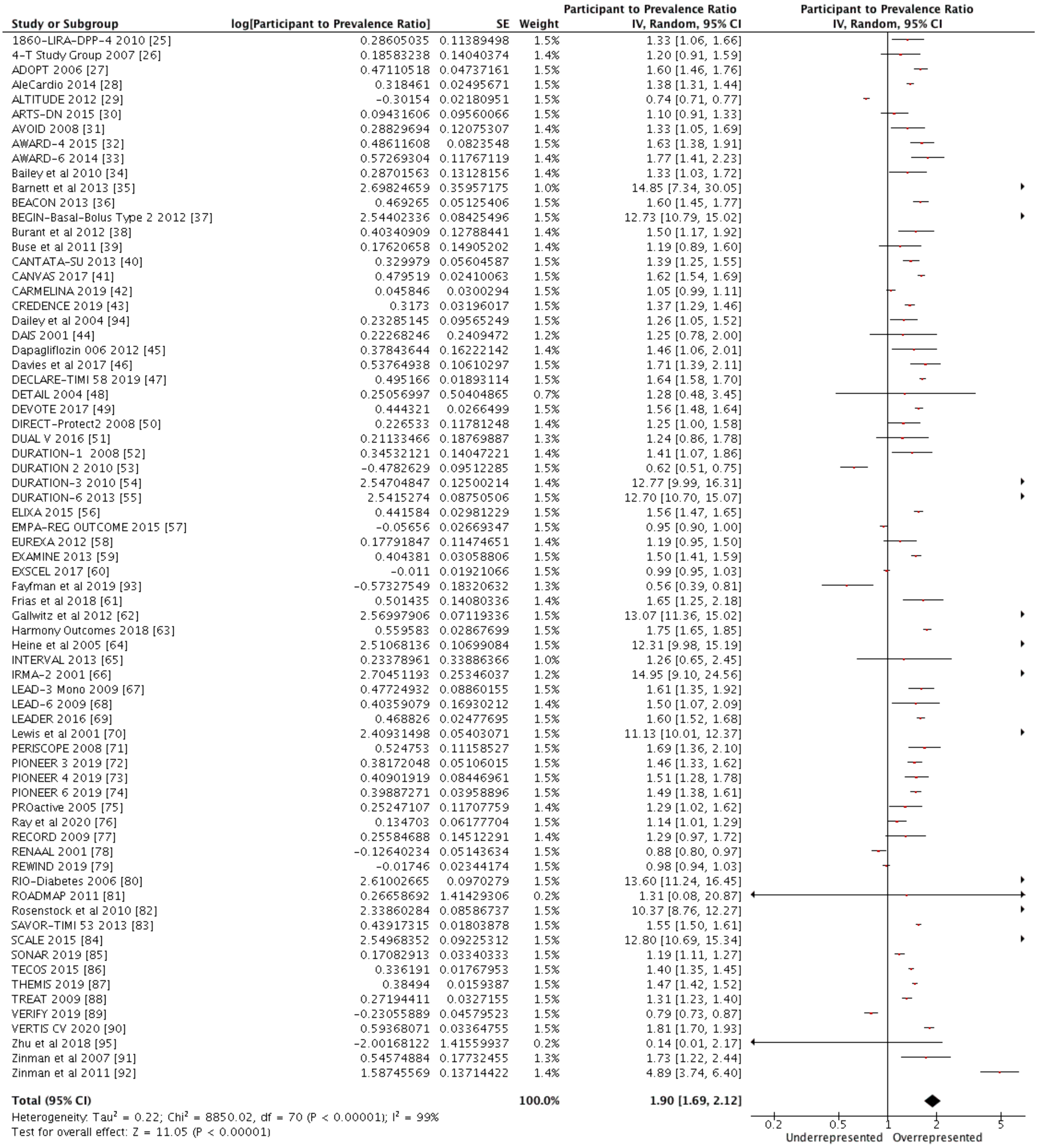

**ESM Fig. 6b.** PPR Racialised – Industry Trials including Dailey et al (2004), Fayfman et al (2019), and Zhu et al (2018).

| Study or Subgroup | log[Participant to Prevalence Ratio] | SE | Weight | Participant to Prevalence Ratio IV, Random, 95% CI |
|---|---|---|---|---|
| 1860-LIRA-DPP-4 2010 [25] | -0.95936968 | 0.11389498 | 1.5% | 0.38 [0.31, 0.48] |
| 4-T Study Group 2007 [26] | -1.10308987 | 0.14040374 | 1.4% | 0.33 [0.25, 0.44] |
| ADOPT 2006 [27] | -1.35263502 | 0.04737161 | 1.5% | 0.26 [0.24, 0.28] |
| AleCardio 2014 [28] | -0.43547148 | 0.02495671 | 1.5% | 0.65 [0.62, 0.68] |
| ALTITUDE 2012 [29] | 0.616105 | 0.02180951 | 1.5% | 1.85 [1.77, 1.93] |
| ARTS-DN 2015 [30] | -0.39096762 | 0.09560066 | 1.5% | 0.68 [0.56, 0.82] |
| AVOID 2008 [31] | -0.97403276 | 0.12075307 | 1.4% | 0.38 [0.30, 0.48] |
| AWARD-4 2015 [32] | -0.88993082 | 0.0823548 | 1.5% | 0.41 [0.35, 0.48] |
| AWARD-6 2014 [33] | -1.30102717 | 0.11767119 | 1.4% | 0.27 [0.22, 0.34] |
| Bailey et al 2010 [34] | -1.03480078 | 0.13128156 | 1.4% | 0.36 [0.27, 0.46] |
| Barnett et al 2013 [35] | -3.33805182 | 0.35957175 | 1.0% | 0.04 [0.02, 0.07] |
| BEACON 2013 [36] | -0.82951 | 0.05125406 | 1.5% | 0.44 [0.39, 0.48] |
| BEGIN-Basal-Bolus Type 2 2012 [37] | -1.6966211 | 0.08425496 | 1.5% | 0.18 [0.16, 0.22] |
| Burant et al 2012 [38] | -0.94742456 | 0.127884 | 1.4% | 0.39 [0.30, 0.50] |
| Buse et al 2011 [39] | -0.44460406 | 0.14905202 | 1.4% | 0.64 [0.48, 0.86] |
| CANTATA-SU 2013 [40] | -0.45892 | 0.05604587 | 1.5% | 0.63 [0.57, 0.71] |
| CANVAS 2017 [41] | -0.86572 | 0.02410063 | 1.5% | 0.42 [0.40, 0.44] |
| CARMELINA 2019 [42] | -0.16663 | 0.0300294 | 1.5% | 0.85 [0.80, 0.90] |
| CREDENCE 2019 [43] | -0.43315 | 0.03196017 | 1.5% | 0.65 [0.61, 0.69] |
| Dailey et al 2004 [94] | -0.79476237 | 0.09565249 | 1.5% | 0.45 [0.37, 0.54] |
| DAIS 2001 [44] | -1.69308864 | 0.2409472 | 1.3% | 0.18 [0.11, 0.29] |
| Dapagliflozin 006 2012 [45] | -1.94395129 | 0.16222142 | 1.4% | 0.14 [0.10, 0.20] |
| Davies et al 2017 [46] | -1.10947336 | 0.10610297 | 1.5% | 0.33 [0.27, 0.41] |
| DECLARE-TIMI 58 2019 [47] | -0.9244 | 0.01893114 | 1.5% | 0.40 [0.38, 0.41] |
| DETAIL 2004 [48] | -2.68314552 | 0.50404865 | 0.8% | 0.07 [0.03, 0.18] |
| DEVOTE 2017 [49] | -0.74794 | 0.0266499 | 1.5% | 0.47 [0.45, 0.50] |
| DIRECT-Protect2 2008 [50] | -1.78273 | 0.11781248 | 1.4% | 0.17 [0.13, 0.21] |
| DUAL V 2016 [51] | -1.46934682 | 0.18769887 | 1.4% | 0.23 [0.16, 0.33] |
| DURATION-1 2008 [52] | -0.70963839 | 0.14047221 | 1.4% | 0.49 [0.37, 0.65] |
| DURATION 2 2010 [53] | 0.38415789 | 0.09512285 | 1.5% | 1.47 [1.22, 1.77] |
| DURATION-3 2010 [54] | -1.71138382 | 0.12500214 | 1.4% | 0.18 [0.14, 0.23] |
| DURATION-6 2013 [55] | -1.6846443 | 0.08750506 | 1.5% | 0.19 [0.16, 0.22] |
| ELIXA 2015 [56] | -0.73949 | 0.02981229 | 1.5% | 0.48 [0.45, 0.51] |
| EMPA-REG OUTCOME 2015 [57] | 0.16543 | 0.02669347 | 1.5% | 1.18 [1.12, 1.24] |
| EUREXA 2012 [58] | -1.01362501 | 0.11474651 | 1.4% | 0.36 [0.29, 0.45] |
| EXAMINE 2013 [59] | -0.63333 | 0.03058806 | 1.5% | 0.53 [0.50, 0.56] |
| EXSCEL 2017 [60] | 0.035167 | 0.01921066 | 1.5% | 1.04 [1.00, 1.08] |
| Fayfman et al 2019 [93] | 0.34413021 | 0.18320632 | 1.4% | 1.41 [0.99, 2.02] |
| Frias et al 2018 [61] | -0.94919 | 0.14080336 | 1.4% | 0.39 [0.29, 0.51] |
| Gallwitz et al 2012 [62] | -1.83261422 | 0.07119336 | 1.5% | 0.16 [0.14, 0.18] |
| Harmony Outcomes 2018 [63] | -1.2242 | 0.02867699 | 1.5% | 0.29 [0.28, 0.31] |
| Heine et al 2005 [64] | -1.54944699 | 0.10699084 | 1.5% | 0.21 [0.17, 0.26] |
| INTERVAL 2013 [65] | -1.9783755 | 0.33886366 | 1.1% | 0.14 [0.07, 0.27] |
| IRMA-2 2001 [66] | -3.54023025 | 0.25346037 | 1.2% | 0.03 [0.02, 0.05] |
| LEAD-3 Mono 2009 [67] | -0.85755777 | 0.08860155 | 1.5% | 0.42 [0.36, 0.50] |
| LEAD-6 2009 [68] | -1.55240627 | 0.16930212 | 1.4% | 0.21 [0.15, 0.30] |
| LEADER 2016 [69] | -0.82799935 | 0.02477695 | 1.5% | 0.44 [0.42, 0.46] |
| Lewis et al 2001 [70] | -1.2207694 | 0.05403071 | 1.5% | 0.30 [0.27, 0.33] |
| PERISCOPE 2008 [71] | -1.04872 | 0.11158527 | 1.5% | 0.35 [0.28, 0.44] |
| PIONEER 3 2019 [72] | -0.57549323 | 0.05106015 | 1.5% | 0.56 [0.51, 0.62] |
| PIONEER 4 2019 [73] | -0.64575943 | 0.08446961 | 1.5% | 0.52 [0.44, 0.62] |
| PIONEER 6 2019 [74] | -0.6188366 | 0.03958896 | 1.5% | 0.54 [0.50, 0.58] |
| PROactive 2005 [75] | -2.8076089 | 0.11707759 | 1.4% | 0.06 [0.05, 0.08] |
| Ray et al 2020 [76] | -0.63823 | 0.06177704 | 1.5% | 0.53 [0.47, 0.60] |
| RECORD 2009 [77] | -3.07676295 | 0.14512291 | 1.4% | 0.05 [0.03, 0.06] |
| RENAAL 2001 [78] | 0.13654033 | 0.05143634 | 1.5% | 1.15 [1.04, 1.27] |
| REWIND 2019 [79] | 0.056527 | 0.02344174 | 1.5% | 1.06 [1.01, 1.11] |
| RIO-Diabetes 2006 [80] | -2.09697685 | 0.0970279 | 1.5% | 0.12 [0.10, 0.15] |
| ROADMAP 2011 [81] | -7.64122356 | 1.41429306 | 0.2% | 0.00 [0.00, 0.01] |
| Rosenstock et al 2010 [82] | -1.05587998 | 0.08586737 | 1.5% | 0.35 [0.29, 0.41] |
| SAVOR-TIMI 53 2013 [83] | -0.73213606 | 0.01803878 | 1.5% | 0.48 [0.46, 0.50] |
| SCALE 2015 [84] | -1.7244559 | 0.09225312 | 1.5% | 0.18 [0.15, 0.21] |
| SONAR 2019 [85] | -0.19282598 | 0.03340333 | 1.5% | 0.82 [0.77, 0.88] |
| TECOS 2015 [86] | -0.47192 | 0.01767953 | 1.5% | 0.62 [0.60, 0.65] |
| THEMIS 2019 [87] | -0.58343 | 0.0159387 | 1.5% | 0.56 [0.54, 0.58] |
| TREAT 2009 [88] | -0.34843096 | 0.0327155 | 1.5% | 0.71 [0.66, 0.75] |
| VERIFY 2019 [89] | 0.51502772 | 0.04579523 | 1.5% | 1.67 [1.53, 1.83] |
| VERTIS CV 2020 [90] | -1.4403285 | 0.03364755 | 1.5% | 0.24 [0.22, 0.25] |
| Zhu et al 2018 [95] | 0.01263138 | 1.41559937 | 0.2% | 1.01 [0.06, 16.23] |
| Zinman et al 2007 [91] | -1.15003467 | 0.17732455 | 1.4% | 0.32 [0.22, 0.45] |
| Zinman et al 2011 [92] | -0.31596083 | 0.13714422 | 1.4% | 0.73 [0.56, 0.95] |
| **Total (95% CI)** | | | **100.0%** | **0.37 [0.33, 0.42]** |

Heterogeneity: $Tau^2 = 0.29$; $Chi^2 = 11736.26$, df = 70 ($P < 0.00001$); $I^2 = 99\%$
Test for overall effect: $Z = 14.90$ ($P < 0.00001$)

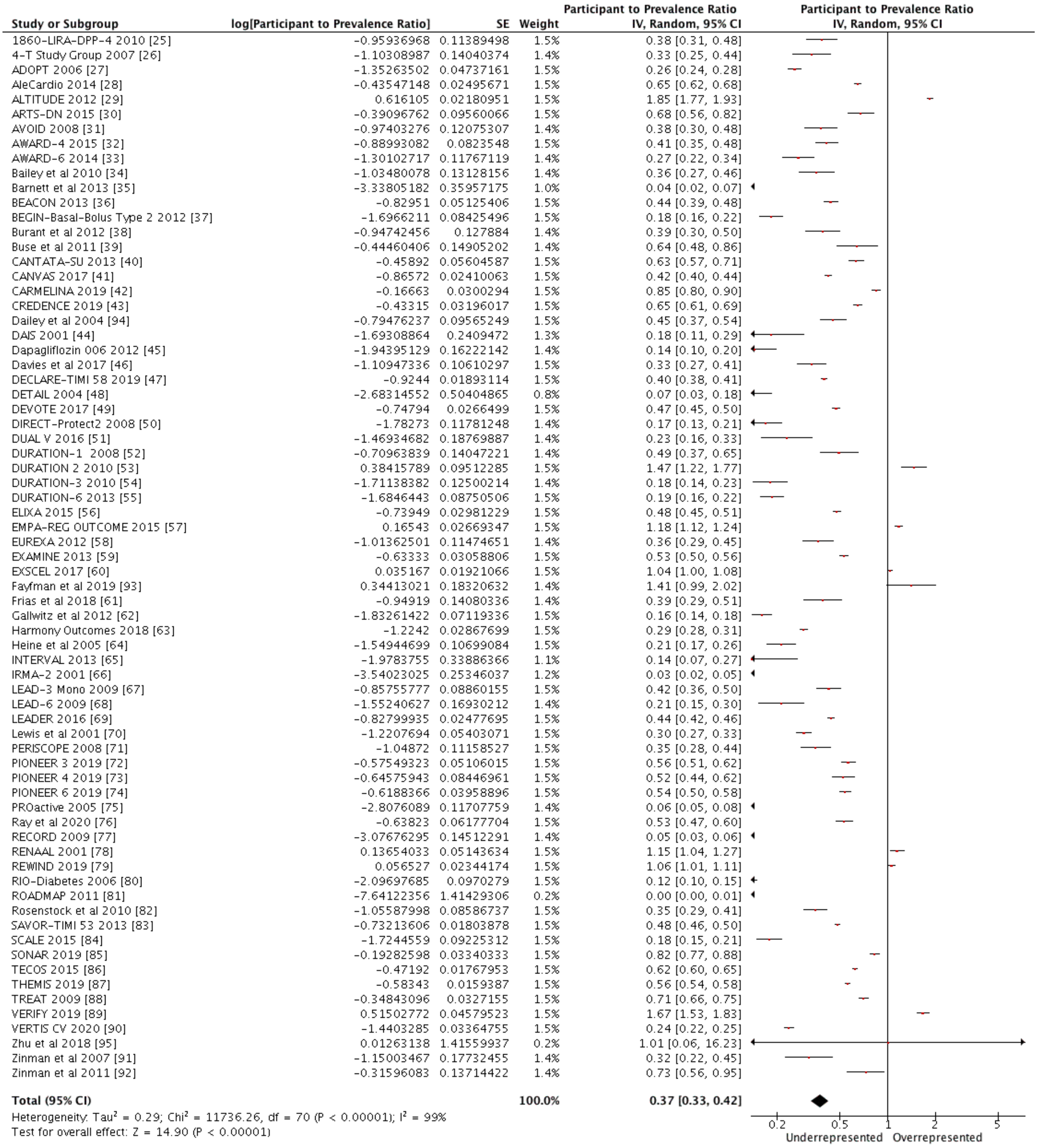

**ESM Fig. 7a.** PPR White – Government Trials including Dailey et al (2004), Fayfman et al (2019), and Zhu et al (2018).

| Study or Subgroup | log[Participant to Prevalence Ratio] | SE | Weight | Participant to Prevalence Ratio IV, Random, 95% CI |
|---|---|---|---|---|
| ACCORD 2008 [10] | 0.284088 | 0.02063059 | 8.8% | 1.33 [1.28, 1.38] |
| BARI 2D 2009 [11] | 0.30706866 | 0.04335634 | 8.5% | 1.36 [1.25, 1.48] |
| CARDS 2004 [12] | 0.13977786 | 0.08091064 | 7.5% | 1.15 [0.98, 1.35] |
| Dailey et al 2004 [94] | 0.23285145 | 0.09565249 | 7.0% | 1.26 [1.05, 1.52] |
| Fayfman et al 2019 [93] | −0.57327549 | 0.18320632 | 4.5% | 0.56 [0.39, 0.81] |
| FIELD 2005 [13] | 0.11145231 | 0.03917239 | 8.5% | 1.12 [1.04, 1.21] |
| GRADE 2019 [14] | 0.30314965 | 0.02964429 | 8.7% | 1.35 [1.28, 1.44] |
| Kadoglou et al 2007 [15] | 0.1793224 | 1.41774469 | 0.1% | 1.20 [0.07, 19.26] |
| Levin et al 2000 [16] | 0.28846966 | 0.16917307 | 4.9% | 1.33 [0.96, 1.86] |
| Meyer et al 2010 [17] | 0.44348577 | 0.17343648 | 4.8% | 1.56 [1.11, 2.19] |
| MiTy 2020 [18] | −0.72539254 | 0.09713954 | 7.0% | 0.48 [0.40, 0.59] |
| SPREAD-DIMCAD 2013 [19] | −2.17712989 | 1.41537609 | 0.2% | 0.11 [0.01, 1.82] |
| The Qatar Study 2017 [20] | −0.54756745 | 0.25913541 | 3.0% | 0.58 [0.35, 0.96] |
| TINSAL-T2D 2010 [21] | 0.04898969 | 0.1924831 | 4.3% | 1.05 [0.72, 1.53] |
| TINSAL-T2D-II 2013 [22] | 0.08507576 | 0.11844798 | 6.3% | 1.09 [0.86, 1.37] |
| UKPDS 57 2002 [23] | −0.03646335 | 0.08550871 | 7.4% | 0.96 [0.82, 1.14] |
| VADT 2009 [24] | 0.24627412 | 0.04868961 | 8.3% | 1.28 [1.16, 1.41] |
| Zhu et al 2018 [95] | −2.00168122 | 1.41559937 | 0.2% | 0.14 [0.01, 2.17] |
| **Total (95% CI)** | | | **100.0%** | **1.08 [0.97, 1.20]** |

Heterogeneity: $Tau^2 = 0.03$; $Chi^2 = 170.67$, df = 17 ($P < 0.00001$); $I^2 = 90\%$
Test for overall effect: Z = 1.36 (P = 0.18)

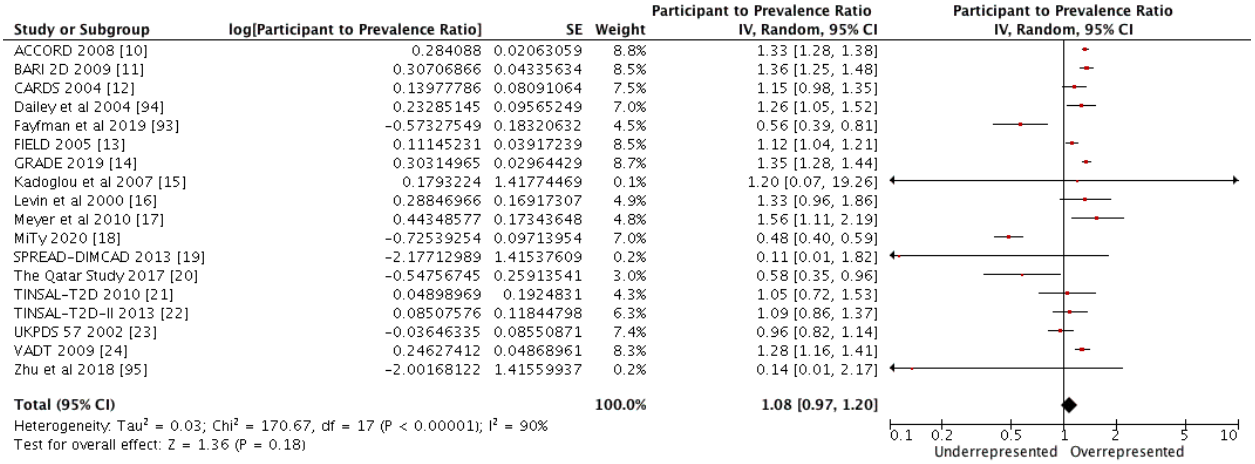


**ESM Fig. 7b.** PPR Racialised – Government Trials including Dailey et al (2004), Fayfman et al (2019), and Zhu et al (2018).

| Study or Subgroup | log[Participant to Prevalence Ratio] | SE | Weight | Participant to Prevalence Ratio IV, Random, 95% CI |
|---|---|---|---|---|
| ACCORD 2008 [10] | −0.37005273 | 0.02063059 | 7.6% | 0.69 [0.66, 0.72] |
| BARI 2D 2009 [11] | −0.4130597 | 0.04335634 | 7.4% | 0.66 [0.61, 0.72] |
| CARDS 2004 [12] | −1.1489308 | 0.08091064 | 7.1% | 0.32 [0.27, 0.37] |
| Dailey et al 2004 [94] | −0.79476237 | 0.09565249 | 6.9% | 0.45 [0.37, 0.54] |
| Fayfman et al 2019 [93] | 0.34413021 | 0.18320632 | 5.6% | 1.41 [0.99, 2.02] |
| FIELD 2005 [13] | −0.8612428 | 0.03917239 | 7.5% | 0.42 [0.39, 0.46] |
| GRADE 2019 [14] | −0.40552237 | 0.02964429 | 7.5% | 0.67 [0.63, 0.71] |
| Kadoglou et al 2007 [15] | −3.52142848 | 1.41774469 | 0.3% | 0.03 [0.00, 0.48] |
| Levin et al 2000 [16] | −0.37803625 | 0.16917307 | 5.8% | 0.69 [0.49, 0.95] |
| Meyer et al 2010 [17] | −0.74534959 | 0.17343648 | 5.7% | 0.47 [0.34, 0.67] |
| MiTy 2020 [18] | 0.62129093 | 0.09713954 | 6.9% | 1.86 [1.54, 2.25] |
| SPREAD-DIMCAD 2013 [19] | 0.01294686 | 1.41537609 | 0.3% | 1.01 [0.06, 16.23] |
| The Qatar Study 2017 [20] | 0.05578154 | 0.25913541 | 4.4% | 1.06 [0.64, 1.76] |
| TINSAL-T2D 2010 [21] | −0.04842169 | 0.1924831 | 5.4% | 0.95 [0.65, 1.39] |
| TINSAL-T2D-II 2013 [22] | −0.0872994 | 0.11844798 | 6.6% | 0.92 [0.73, 1.16] |
| UKPDS 57 2002 [23] | 0.15102455 | 0.08550871 | 7.0% | 1.16 [0.98, 1.38] |
| VADT 2009 [24] | −0.30501898 | 0.04868961 | 7.4% | 0.74 [0.67, 0.81] |
| Zhu et al 2018 [95] | 0.01263138 | 1.41559937 | 0.3% | 1.01 [0.06, 16.23] |
| **Total (95% CI)** | | | **100.0%** | **0.72 [0.61, 0.85]** |

Heterogeneity: $Tau^2 = 0.09$; $Chi^2 = 435.51$, df = 17 ($P < 0.00001$); $I^2 = 96\%$
Test for overall effect: Z = 3.83 (P = 0.0001)

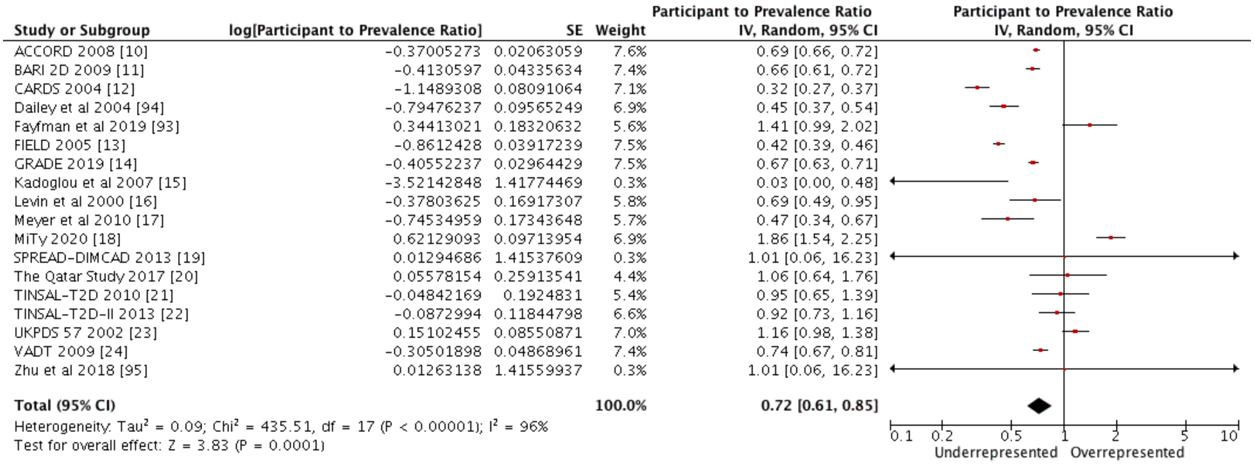

Supplement: Supplementary file 1 — Supplementary file1 (PDF 1.28 MB) [file 125_2023_6052_MOESM1_ESM.pdf]
